# Comprehensive reanalysis for CNVs in ES data from unsolved rare disease cases results in new diagnoses

**DOI:** 10.1038/s41525-024-00436-6

**Published:** 2024-10-26

**Authors:** German Demidov, Burcu Yaldiz, José Garcia-Pelaez, Elke de Boer, Nika Schuermans, Liedewei Van de Vondel, Ida Paramonov, Lennart F. Johansson, Francesco Musacchia, Elisa Benetti, Gemma Bullich, Karolis Sablauskas, Sergi Beltran, Christian Gilissen, Alexander Hoischen, Stephan Ossowski, Richarda de Voer, Katja Lohmann, Carla Oliveira, Ana Topf, Lisenka E. L. M. Vissers, Solve-RD Consortium, German Demidov, Burcu Yaldiz, José Garcia-Pelaez, Nika Schuermans, Ida Paramonov, Lennart F. Johansson, Gemma Bullich, Karolis Sablauskas, Sergi Beltran, Christian Gilissen, Alexander Hoischen, Stephan Ossowski, Katja Lohmann, Carla Oliveira, Ana Topf, Lisenka E. L. M. Vissers, Olaf Riess, Tobias B. Haack, Holm Graessner, Birte Zurek, Kornelia Ellwanger, Marc Sturm, Joohyun Park, Leon Schütz, Julia M. Schulze-Hentrich, Rebecca Schüle, Jishu Xu, Melanie Kellner, Baptist Resch, Ingrid Kolen, Matthis Synofzik, Carlo Wilke, Andreas Traschütz, Danique Beijer, Peter Heutink, Ludger Schöls, Holger Hengel, Holger Lerche, Christian Boßelmann, Josua Kegele, Robert Lauerer-Braun, Stephan Lauxmann, Han Brunner, Hans Scheffer, Nicoline Hoogerbrugge, Peter A. C. ’t Hoen, Wouter Steyaert, Richarda de Voer, Erik-Jan Kamsteeg, Bart van de Warrenburg, Nienke van Os, Iris te Paske, Erik Janssen, Elke de Boer, Marloes Steehouwer, Kornelia Neveling, Bart van der Sanden, Lydia Sagath, Tjitske Kleefstra, Anthony J. Brookes, Spencer Gibson, Umar Riaz, Greg Warren, Sai Anuhya Nalagandla, Yunze Patrick Wang, Deepthi Sukumaran, Sadegh Abadijou, Volker Straub, Chiara Marini Bettolo, Jordi Diaz Manera, Sophie Hambleton, Karin Engelhardt, Jill Clayton-Smith, Siddharth Banka, Elizabeth Alexander, Adam Jackson, Laurence Faivre, Christel Thauvin, Antonio Vitobello, Anne-Sophie Denommé-Pichon, Yannis Duffourd, Ange-Line Bruel, Victor Couturier, Ivo Glynne Gut, Davide Piscia, Leslie Matalonga, Anastasios Papakonstantinou, Alberto Corvo, Marcos Fernandez-Callejo, Carles Hernández, Daniel Picó, Anna Esteve Codina, Marc Dabad, Marta Gut, Emanuele Raineri, Gulcin Gumus, Virginie Bros-Facer, Ana Rath, Marc Hanauer, David Lagorce, Oscar Hongnat, Maroua Chahdil, Caterina Lucano, Emeline Lebreton, Giovanni Stevanin, Alexandra Durr, Claire-Sophie Davoine, Léna Guillot-Noel, Anna Heinzmann, Giulia Coarelli, Gisèle Bonne, Teresinha Evangelista, Valérie Allamand, Isabelle Nelson, Rabah Ben Yaou, Corinne Metay, Bruno Eymard, Enzo Cohen, Antonio Atalaia, Tanya Stojkovic, Milan Macek, Marek Turnovec, Dana Thomasová, Radka Pourová Kremliková, Vera Franková, Markéta Havlovicová, Lukáš Ryba, Petra Lišková, Pavla Doležalová, Alice Krebsová, Helen Parkinson, Thomas Keane, Mallory Freeberg, Coline Thomas, Dylan Spalding, Peter Robinson, Daniel Danis, Glenn Robert, Alessia Costa, Mike Hanna, Henry Houlden, Mary Reilly, Jana Vandrovcova, Stephanie Efthymiou, Heba Morsy, Elisa Cali, Francesca Magrinelli, Sanjay M. Sisodiya, Ravishankara Bellampalli, Patrick Moloney, Jonathan Rohrer, Francesco Muntoni, Irina Zaharieva, Anna Sarkozy, Luke Perry, Veronica Pini, Juliane Müller, Vincent Timmerman, Jonathan Baets, Geert de Vries, Jonathan De Winter, Peter de Jonghe, Liedewei Van de Vondel, Willem De Ridder, Sarah Weckhuysen, Hannah Stamberger, Charissa Millevert, Noor Smal, Vincenzo Nigro, Manuela Morleo, Michele Pinelli, Sandro Banfi, Annalaura Torella, Roberta Zeuli, Mariateresa Zanobio, Giulio Piluso, Alessandra Ferlini, Rita Selvatici, Francesca Gualandi, Stefania Bigoni, Marcella Neri, Stefan Aretz, Isabel Spier, Anna Katharina Sommer, Sophia Peters, Rita Barbosa-Matos, Celina São José, Marta Ferreira, Irene Gullo, Susana Fernandes, Luzia Garrido, Pedro Ferreira, Fátima Carneiro, Morris A. Swertz, Joeri K. van der Velde, Gerben van der Vries, Pieter B. Neerincx, Dieuwke Roelofs-Prins, David Ruvolo, Marielle van Gijn, Kristin M. Abbott, Wilhemina S. Kerstjens Frederikse, Eveline Zonneveld-Huijssoon, Sebastian Köhler, Alison Metcalfe, Richard Moore, Alain Verloes, Séverine Drunat, Delphine Heron, Cyril Mignot, Boris Keren, Jean-Madeleine de Sainte Agathe, Rami Abou Jamra, Marc Abramowicz, Özge Aksel Kiliçarslan, Nicholas Allen, Francisco Javier Alonso García de la Rosa, Simona Balestrini, Peter Balicza, Tobias Bartolomaeus, Ayşe Nazl Başak, Laura Batlle Masó, David Beeson, Valerie Benoit, Katherine Benson, Eva Bermejo Sánchez, Emilia K. Bijlsma, Elke Bogaert, Mara Bourbouli, Kaan Boztug, Sylvain Brohée, Susan Byrne, Andrés Caballero Garcia de Oteyza, Gabriel Capella, Evelina Carpancea, Gianpiero Cavalleri, Ana Cazurro-Gutiérrez, Patrick F. Chinnery, Maria-Roberta Cilio, Andrea Ciolfi, Kristl Claeys, Roger Colobran, Isabell Cordts, Judith Cossins, Karin Dahan, Bruno Dallapiccola, Norman Delanty, Christel Depienne, Chantal Depondt, Bart Dermaut, Marcus Deschauer, Julie Desir, Anne Destrée, Minas Drakos, Sarah Duerinckx, Berta Estevez, Athanasios Evangeliou, Chiara Fallerini, Marco Ferilli, Simone Furini, Julien Gagneur, Hamidah Ghani, Marie Greally, Bodo Grimbacher, Renzo Guerrini, Peter Hackman, Matthias Haimel, Eva Hammar Bouveret, Dimitri Hemelsoet, Rebecca Herzog, Mariette J. V. Hoffer, Elke Holinski-Feder, Rita Horvath, Manon Huibers, Michele Iacomino, Mridul Johari, Elisabeth Kapaki, Deniz Karadurmus, Mert Karakaya, Evgenia Kokosali, Christian Korff, Leon Krass, Didier Lacombe, Andreas Laner, Helen Leavis, Damien Lederer, Elsa Leitão, Hanns Lochmüller, Estrella López Martín, Rebeka Luknárová, Alfons Macaya, Sivasankar Malaichamy, Anna Marcé-Grau, Beatriz Martínez Delgado, Sandrine Mary, Frédéric Masclaux, Lambros Mathioudakis, Ales Maver, Patrick May, Isabelle Maystadt, Davide Mei, Christian Mertes, Colombine Meunier, Maria Judit Molnar, Olivier Monestier, Stéphanie Moortgat, Alexander Münchau, Francina Munell, Andrés Nascimento Osorio, Daniel Natera de Benito, Mary O. Reghan, Catarina Olimpio, Elena Parrini, Martje Pauly, Belén Pérez-Dueñas, Borut Peterlin, Konrad Platzer, Kiran Polavarapu, Bruce Poppe, Manuel Posada De la Paz, Flavia Privitera, Francesca Clementina Radio, Thiloka Ratnaike, Alessandra Renieri, Antonella Riva, Caroline Rooryck, Andreas Roos, Claudia A. L. Ruivenkamp, Andreas Rump, Gijs W. E. Santen, Marco Savarese, Marcello Scala, Katherine Schon, Evelin Schröck, Paolo Scudieri, Martha Spilioti, Verena Steinke-Lange, Pasquale Striano, Yves Sznajer, Marco Tartaglia, Rachel Thompson, Aurelien Trimouille, Bjarne Udd, Paolo Uva, Laura Valle, Lars van der Veken, Roxane van Heurck, Joris van Montfrans, Erika Van Nieuwenhove, Hannah Verdin, David Webb, Brunhilde Wirth, Vicente A. Yépez, Ioannis Zaganas, Federico Zara, Kristina Zguro, Steven Laurie, Steven Laurie

**Affiliations:** 1 Institute of Medical Genetics and Applied Genomics, University of Tübingen, Tübingen, Germany; 2 Institute for Bioinformatics and Medical Informatics (IBMI), University of Tübingen, Tübingen, Germany; 3 Department of Human Genetics, Radboud University Medical Center, Nijmegen, The Netherlands; 4 Department of Clinical Genetics, Maastricht University Medical Center, Maastricht, The Netherlands; 5 i3S - Instituto de Investigação e Inovação em Saúde, Rua Alfredo Allen, 208, 4200-135 Porto, Portugal; 6 IPATIMUP - Institute of Molecular Pathology and Immunology, University of Porto, Porto, Portugal; 7 Faculty of Medicine, University of Porto, Porto, Portugal; 8 Donders Institute for Brain, Cognition and Behaviour, Radboud University, Nijmegen, The Netherlands; 9 Department of Clinical Genetics, Erasmus MC University Medical Center, Rotterdam, The Netherlands; 10 Center for Medical Genetics, Ghent University Hospital, Ghent, Belgium; 11 Translational Neurosciences, Faculty of Medicine and Health Sciences, University of Antwerp, Antwerp, Belgium; 12 Laboratory of Neuromuscular Pathology, Institute Born-Bunge, University of Antwerp, Antwerp, Belgium; 13 Centro Nacional de Análisis Genómico (CNAG), C/Baldiri Reixac 4, 08028 Barcelona, Spain; 14 Universitat de Barcelona (UB), Barcelona, Spain; 15 University of Groningen, University Medical Center Groningen, Department of Genetics, Groningen, The Netherlands; 16 Center for Human Technologies, Italian Institute of Technology (IIT), Genova, Italy; 17 Telethon Institute for Genetics and Medicine, 80078 Pozzuoli (Napoli), Italy; 18 Department of Medical Biotechnologies, Med Biotech Hub and Competence Center, University of Siena, 53100 Siena, Italy; 19 Institute of Data Science and Digital Technologies, Vilnius University, Vilnius, Lithuania; 20 Departament de Genètica, Microbiologia i Estadística, Facultat de Biologia, Universitat de Barcelona (UB), Barcelona, Spain; 21 Radboud Institute for Molecular Life Sciences, Nijmegen, The Netherlands; 22 Department of Internal Medicine and Radboud Center for Infectious Diseases (RCI), Radboud University Medical Center, Nijmegen, The Netherlands; 23 Research Institute for Medical Innovation, Radboud University Medical Center, Nijmegen, The Netherlands; 24 Institute of Neurogenetics, University of Lübeck, Ratzeburger Allee 160, 23562 Lübeck, Germany; 25 John Walton Muscular Dystrophy Research Centre, Translational and Clinical Research Institute, Newcastle University and Newcastle Hospitals NHS Foundation Trust, Newcastle upon Tyne, UK; 26 Centre for Rare Diseases, University of Tübingen, Tübingen, Germany; 27 Department of Genetics/Epigenetics, Faculty NT, Saarland University, Saarbrücken, Germany; 28 Department of Neurodegeneration, Hertie Institute for Clinical Brain Research (HIH), University of Tübingen, Tübingen, Germany; 29 Division of Neurodegenerative Diseases and Movement Disorders, Department of Neurology, University of Heidelberg, Heidelberg, Germany; 30 German Center for Neurodegenerative Diseases (DZNE), Tübingen, Germany; 31 Division Translational Genomics of Neurodegenerative Diseases, Hertie-Institute for Clinical Brain Research and Center of Neurology, University of Tübingen, Tübingen, Germany; 32 Department of Neurology and Epileptology, Hertie Institute for Clinical Brain Research (HIH), University of Tübingen, Tübingen, Germany; 33 Center for Molecular and Biomolecular Informatics, Radboud University Medical Center, Nijmegen, The Netherlands; 34 Department of Neurology, Radboud University Medical Center, Nijmegen, The Netherlands; 35 Department of Genetics and Genome Biology, University of Leicester, Leicester, UK; 36 Primary Immunodeficiency Group, Translational and Clinical Research Institute, Newcastle University and Newcastle upon Tyne Hospitals NHS Foundation Trust, Newcastle upon Tyne, UK; 37 Division of Evolution, Infection and Genomics, School of Biological Sciences, Faculty of Biology, Medicine and Health, University of Manchester, Manchester, M13 9WL UK; 38 Manchester Centre for Genomic Medicine, St Mary’s Hospital, Manchester University Hospitals NHS Foundation Trust, Health Innovation Manchester, Manchester, M13 9WL UK; 39 Dijon University Hospital, Genetics Department, Dijon, France; 40 Dijon University Hospital, Centre of Reference for Rare Diseases: Development disorders and malformation syndromes, Dijon, France; 41 Inserm - University of Burgundy-Franche Comté, UMR1231 GAD Dijon, France; 42 Dijon University Hospital, FHU-TRANSLAD, Dijon, France; 43 Dijon University Hospital, GIMI institute, Dijon, France; 44 Universitat Pompeu Fabra (UPF), Barcelona, Spain; 45 EURORDIS-Rare Diseases Europe, Sant Antoni Maria Claret, 167 - 08025 Barcelona, Spain; 46 EURORDIS-Rare Diseases Europe, Plateforme Maladies Rares, 75014 Paris, France; 47 INSERM, US14 - Orphanet, Plateforme Maladies Rares, 75014 Paris, France; 48 Institut du Cerveau, INSERM U1127, CNRS UMR7225, Sorbonne university, Paris, France; 49 INCIA, EPHE, CNRS UMR5287, Bordeaux university, Bordeaux, France; 50 Hôpital de la Pitié-Salpêtrière, Assistance Publique-Hôpitaux de Paris (AP-HP), Paris, France; 51 Sorbonne Université, Inserm, Institut de Myologie, Centre de Recherche en Myologie, F-75013 Paris, France; 52 AP-HP, Centre de Référence de Pathologie Neuromusculaire Nord, Est, Ile-de-France, Institut de Myologie, G.H. Pitié-Salpêtrière, F-75013 Paris, France; 53 Institut de Myologie, Equipe Bases de données, G.H. Pitié-Salpêtrière, F-75013 Paris, France; 54 AP-HP, Unité Fonctionnelle de Cardiogénétique et Myogénétique Moléculaire et Cellulaire, G.H. Pitié-Salpêtrière, F-75013 Paris, France; 55 Department of Biology and Medical Genetics, Charles University Prague-2nd Faculty of Medicine and University Hospital Motol, Prague, Czech Republic; 56 Department of Paediatrics and Inherited Metabolic Disorders, First Faculty of Medicine, Charles University and General University Hospital in Prague, Prague, Czech Republic; 57 Department of Ophthalmology, First Faculty of Medicine, Charles University and General University Hospital in Prague, Prague, Czech Republic; 58 Department of Paediatrics and Inherited Metabolic Disorders, 1st Faculty of Medicine, Centre for Paediatric Rheumatology and Autoinflammatory Diseases, Charles University and General University Hospital in Prague, Prague, Czech Republic; 59 Department of Cardiology-Institute of Clinical and Experimental Medicine and Department of Biology and Medical Genetics-2nd Faculty of Medicine Charles University, Prague, Czech Republic; 60 European Bioinformatics Institute, European Molecular Biology Laboratory, Wellcome Genome Campus, Hinxton, Cambridge, UK; 61 CSC–IT Center for Science, 02101 Espoo, Finland; 62 Jackson Laboratory for Genomic Medicine, Farmington, CT 06032 USA; 63 Berlin Institute of Health at Charité–Universitätsmedizin Berlin, Charitéplatz 1, 10117 Berlin, Germany; 64 Florence Nightingale Faculty of Nursing, Midwifery & Palliative Care, King’s College, London, UK; 65 Society and Ethics Research, Connecting Science, Wellcome Genome Campus, Hinxton, UK; 66 MRC Centre for Neuromuscular Diseases and National Hospital for Neurology and Neurosurgery, UCL Queen Square Institute of Neurology, London, UK; 67 Department of Neuromuscular Diseases, UCL Queen Square Institute of Neurology, London, UK; 68 Department of Human Genetics, Medical Research Institute, Alexandria University, Alexandria, Egypt; 69 Department of Clinical and Movement Neurosciences, UCL Queen Square Institute of Neurology, University College London, WC1N 3BG London, UK; 70 Department of Clinical and Experimental Epilepsy, UCL Queen Square Institute of Neurology, London, UK; 71 Dementia Research Centre, Department of Neurodegenerative Disease, UCL Queen Square Institute of Neurology, London, UK; 72 Dubowitz Neuromuscular Centre, UCL Great Ormond Street Hospital, London, UK; 73 NIHR Great Ormond Street Hospital Biomedical Research Centre, London, UK; 74 Peripheral Neuropathy Research Group, University of Antwerp, Antwerp, Belgium; 75 Neuromuscular Reference Centre, Department of Neurology, Antwerp University Hospital, Antwerpen, Belgium; 76 VIB-CMN, Applied and Translational Neurogenomics Group, Antwerpen, Belgium; 77 Dipartimento di Medicina di Precisione, Università degli Studi della Campania “Luigi Vanvitelli”, Napoli, Italy; 78 Unit of Medical Genetics, Department of Medical Sciences, University of Ferrara, Ferrara, Italy; 79 Institute of Human Genetics, Medical Faculty, University of Bonn, Bonn, Germany; 80 Center for Hereditary Tumor Syndromes, University Hospital Bonn, Bonn, Germany; 81 CHUSJ, Centro Hospitalar e Universitário de São João, Porto, Portugal; 82 Departament of Genetics, Faculty of Medicine, University of Porto, Porto, Portugal; 83 Faculty of Sciences, University of Porto, Porto, Portugal; 84 Department of Genetics, University Medical Center Groningen, University of Groningen, Groningen, The Netherlands; 85 Department of Human Genetics, Amsterdam UMC, University of Amsterdam, Amsterdam, The Netherlands; 86 Ada Health GmbH, Karl-Liebknecht-Str. 1, 10178 Berlin, Germany; 87 College of Health, Well-being and Life-Sciences, Sheffield Hallam University, Sheffield, UK; 88 Advanced Wellbeing Research Centre, Sheffield Hallam University, Olympic Legacy Park, 2 Old Hall Road, Sheffield, S9 3TU UK; 89 Dept of Genetics, Assistance Publique-Hôpitaux de Paris-Université de Paris, Robert DEBRE University Hospital, 48 bd SERURIER, Paris, France; 90 INSERM UMR 1141 “NeuroDiderot”, Hôpital Robert DEBRE, Paris, France; 91 Department of Genetics, Assistance Publique-Hôpitaux de Paris - Sorbonne Université, Pitié-Salpêtrière University Hospital, 83 Boulevard de l’Hôpital, Paris, France; 92 Reference Center of Rare Diseases “Intellectuel Disability of Rare Causes”, Paris, France; 93 Institut du Cerveau (ICM), UMR S 1127, Inserm U1127, CNRS UMR 7225, Sorbonne Université, 75013 Paris, France; 94 Institute of Human Genetics, University of Leipzig Medical Center, Leipzig, Germany; 95 Genetic Medicine Division, University Hospitals and University of Geneva, Geneva, Switzerland; 96 Genetics & Development, Faculty of Medicine, University of Geneva, Geneva, Switzerland; 97 Children’s Hospital of Eastern Ontario Research Institute, University of Ottawa, Ottawa, Canada; 98 Paediatric Neurology, University Hospital Galway, Galway, Ireland; 99 Institute of Rare Diseases Research, Spanish Undiagnosed Rare Diseases Cases Program (SpainUDP) & Undiagnosed Diseases Network International (UDNI), Instituto de Salud Carlos III, Madrid, Spain; 100 Neuroscience Department, Children’s Hospital A. Meyer-University of Florence, 50139 Florence, Italy; 101 Institute of Genomic Medicine and Rare Diseases, Semmelweis University, Budapest, Hungary; 102 Koç Universıty, School of Medicine, Translational Medicine Research Center, KUTTAM-NDAL, Istanbul, Turkey; 103 Infection in Immunocompromised Pediatric Patients Research Group, Vall d’Hebron Research Institute (VHIR), Barcelona, Spain; 104 Pediatric Infectious Diseases and Immunodeficiencies Unit, Vall d’Hebron University Hospital (HUVH), Barcelona, Spain; 105 Nuffield Department of Clinical Neurosciences, University of Oxford, Oxford, UK; 106 Centre de Génétique Humaine, Institut de Pathologie et de Génétique, Gosselies, Belgium; 107 School of Pharmacy and Bimolecular Sciences, RCSI, Dublin, Ireland; 108 Department of Clinical Genetics, Leiden University Medical Center, Leiden, The Netherlands; 109 Neurology/Neurogenetics Laboratory University of Crete, Heraklion, Crete, Greece; 110 Ludwig Boltzmann Institute for Rare and Undiagnosed Diseases, Vienna, Austria; 111 St. Anna Children’s Cancer Research Institute (CCRI), Vienna, Austria; 112 CeMM Research Center for Molecular Medicine of the Austrian Academy of Sciences, Vienna, Austria; 113 Department of Pediatrics and Adolescent Medicine, Medical University of Vienna, Vienna, Austria; 114 St. Anna Children’s Hospital, Department of Pediatrics and Adolescent Medicine, Medical University of Vienna, Vienna, Austria; 115 SFI FutureNeuro Research Centre, Dublin, Ireland; 116 Department of Paediatrics, RCSI, Dublin, Ireland; 117 Department of Paediatrics Neurology, CHI, Dublin, Ireland; 118 Institute for Immunodeficiency, Center for Chronic Immunodeficiency (CCI), Medical Center, Faculty of Medicine, Albert-Ludwigs-University of Freiburg, Freiburg, Germany; 119 RESIST – Cluster of Excellence 2155 to Hanover Medical School, Satellite Center Freiburg, Freiburg, Germany; 120 Bellvitge Biomedical Research Institute (IDIBELL), Barcelona, Spain; 121 Catalan Institute of Oncology (IROCA), Barcelona, Spain; 122 Pediatric Neurology Department, Saint-Luc University Hospital, Université Catholique de Louvain, Brussels, Belgium; 123 SFI Centre for Research Training in Genomics Data Science, Dublin, Ireland; 124 Pediatric Neurology Research Group, Vall d’Hebron Research Institute, Universitat Autònoma de Barcelona, Barcelona, Spain; 125 Department of Clinical Neurosciences, University of Cambridge, Cambridge, UK; 126 Medical Research Council Mitochondrial Biology Unit, University of Cambridge, Cambridge, UK; 127 Molecular Genetics and Functional Genomics, Ospedale Pediatrico Bambino Gesù, IRCCS, Rome, Italy; 128 Department of Neurology, University Hospitals Leuven, Leuven, Belgium; 129 Laboratory for Muscle Diseases and Neuropathies, Department of Neurosciences, and Leuven Brain Institute (LBI), KU Leuven-University of Leuven, Leuven, Belgium; 130 Diagnostic Immunology Research Group, Vall d’Hebron Research Institute (VHIR), Barcelona, Spain; 131 Immunology Division, Genetics Department, Vall d’Hebron University Hospital (HUVH), Barcelona, Spain; 132 Immunology Unit, Department of Cell Biology, Physiology and Immunology. Autonomous University of Barcelona (UAB), Bellaterra, Spain; 133 Department of Neurology, Klinikum rechts der Isar, Technical University Munich, Munich, Germany; 134 Département de néphrologie, Cliniques Universitaires Saint-Luc, Bruxelles, Belgium; 135 Department of Neurology, Beaumont Hospital, Dublin, Ireland; 136 Institute of Human Genetics, University Hospital Essen, University Duisburg-Essen, Essen, Germany; 137 Institut du Cerveau et de la Moelle épinière (ICM), Sorbonne Université, UMR S 1127, Inserm U1127, CNRS UMR 7225, F-75013 Paris, France; 138 Department of Neurology, CUB Erasme Hospital, Hôpital Universitaire de Bruxelles, Université Libre de Bruxelles, Brussels, Belgium; 139 Program for Undiagnosed Rare Diseases (UD-PrOZA), Ghent University Hospital, Ghent, Belgium; 140 Department of Biomolecular Medicine, Faculty of Medicine and Health Sciences, Ghent University, Ghent, Belgium; 141 Neuromuscular Disorders Unit, Department of Pediatric Neurology. Hospital Sant Joan de Déu, Barcelona, Spain; 142 Laboratory of Neurogenetics and Molecular Medicine - IPER, Institut de Recerca Sant Joan de Déu, Barcelona, Spain; 143 Saint Luke Hospital, Division of Child Neurology, Thessaloniki, Greece; 144 Medical Genetics, University of Siena, Siena, Italy; 145 School of Computation, Information and Technology, Technical University of Munich, Garching, Germany; 146 Institute of Human Genetics, School of Medicine, Technical University of Munich, Munich, Germany; 147 Computational Health Center, Helmholtz Center Munich, Neuherberg, Germany; 148 Department of Clinical Genetics, CHI, Dublin, Ireland; 149 Clinic of Rheumatology and Clinical Immunology, Center for Chronic Immunodeficiency (CCI), Medical Center, Faculty of Medicine, Albert-Ludwigs-University of Freiburg, Freiburg, Germany; 150 DZIF–German Center for Infection Research, Satellite Center Freiburg, Freiburg, Germany; 151 CIBSS–Centre for Integrative Biological Signalling Studies, Albert-Ludwigs-University of Freiburg, Freiburg, Germany; 152 Neurofarba Department, University of Florence, Florence, Italy; 153 Folkhälsan Research Centre and Medicum, University of Helsinki, Helsinki, Finland; 154 Department of Neurology, Ghent University Hospital, Ghent, Belgium; 155 Institute of Systems Motor Science, University of Lübeck, Ratzeburger Allee 160, 23562 Lübeck, Germany; 156 Department of Neurology, University Hospital Schleswig Holstein, Ratzeburger Allee 160, 23538 Lübeck, Germany; 157 Medizinische Klinik und Poliklinik IV–Campus Innenstadt, Klinikum der Universität München, Munich, Germany; 158 Department of Genetics, Division Laboratories, Pharmacy and Biomedical Genetics, University Medical Center Utrecht, Utrecht University, Utrecht, The Netherlands; 159 Unit of Medical Genetics, IRCCS Istituto Giannina Gaslini, Genoa, Italy; 160 Clinical Bioinformatics, IRCCS Istituto Giannina Gaslini, Genoa, Italy; 161 Neurochemistry and Biomarker Unit, 1st Department of Neurology, School of Medicine, National and Kapodistrian University of Athens, Eginition Hospital, Athens, Greece; 162 Institute of Human Genetics, University Hospital of Cologne, University Cologne, Kerpener Str. 34, 50931 Cologne, Germany; 163 Center for Molecular Medicine Cologne, University of Cologne, 50931 Cologne, Germany; 164 Institute for Genetics, University of Cologne, 50674 Cologne, Germany; 165 Center for Rare Diseases Cologne, University Hospital Cologne, 50937 Cologne, Germany; 166 Pediatric Neurology Unit, University Hospitals, Geneva, Switzerland; 167 Univ. Bordeaux, MRGM INSERM U1211, CHU de Bordeaux, Service de Génétique Médicale, F-33000 Bordeaux, France; 168 MGZ - Medical Genetics Center, Munich, Germany; 169 Department of Rheumatology & Clinical Immunology, University Medical Center Utrecht, Utrecht University, Utrecht, The Netherlands; 170 Institute of Pathology and Genetics, Charleroi, Belgium; 171 Department of Neuropediatrics and Muscle Disorders, Medical Center, Faculty of Medicine, University of Freiburg, Freiburg, Germany; 172 Centro Nacional de Análisis Genómico (CNAG-CRG), Center for Genomic Regulation, Barcelona Institute of Science and Technology (BIST), Barcelona, Spain; 173 Institut de Neurociències, Universitat Autònoma de Barcelona, Barcelona, Spain; 174 Clinical Institute of Genomic Medicine, University Medical Centre Ljubljana, Ljubljana, Slovenia; 175 Luxembourg Centre for Systems Biomedicine, University of Luxembourg, Esch-sur-Alzette, Luxembourg; 176 Département de Médecine, Université de Namur (Unamur), Namur, Belgium; 177 Center for Rare Diseases, University Hospital Schleswig-Holstein, Ratzeburger Allee 160, 23562 Lübeck, Germany; 178 Applied Research in Neuromuscular Diseases, Institut de Recerca Sant Joan de Déu, Barcelona, Spain; 179 Center for Biomedical Research Network on Rare Diseases (CIBERER), ISCIII, Barcelona, Spain; 180 East Anglian Medical Genetics Service, Cambridge University Hospitals NHS Foundation Trust, Cambridge, UK; 181 Department of Paediatrics, University of Cambridge, Cambridge, UK; 182 Genetica Medica, Azienda Ospedaliero-Universitaria Senese, Senese, Italy; 183 Department of Neurosciences, Rehabilitation, Ophthalmology, Genetics, Maternal and Child Health, University of Genoa, Genoa, Italy; 184 Department of Pediatric Neurology, Developmental Neurology and Social Pediatrics, Children’s Hospital University of Essen, Essen, Germany; 185 Institute for Clinical Genetics, Faculty of Medicine Carl Gustav Carus, Technical University Dresden, Dresden, Germany; 186 Center for Personalized Oncology, University Hospital Carl Gustav Carus, Technical University Dresden, Dresden, Germany; 187 Pediatric Neurology and Muscular Disease Unit, IRCCS Istituto Giannina Gaslini, Genoa, Italy; 188 1sts Department of Neurology, Aristotle University of Thessaloniki, University General Hospital of Thessaloniki, AHEPA, Thessaloniki, Greece; 189 IRCCS Istituto Giannina Gaslini, Genoa, Italy; 190 Human Genetics Department, Saint-Luc University Hospital, Université Catholique de Louvain, Brussels, Belgium; 191 Laboratoire de Génétique Moléculaire, Service de Génétique Médicale, CHU Bordeaux – Hôpital Pellegrin, Place Amélie Raba Léon, 33076 Bordeaux Cedex, France; 192 Tampere Neuromuscular Center, Tampere, Finland; 193 Vasa Central Hospital, Vaasa, Finland; 194 Department of Pediatric Immunology and Infectious Diseases, University Medical Center Utrecht, Utrecht University, Utrecht, The Netherlands

**Keywords:** Molecular medicine, Genetics research

## Abstract

We report the results of a comprehensive copy number variant (CNV) reanalysis of 9171 exome sequencing datasets from 5757 families affected by a rare disease (RD). The data reanalysed was extremely heterogeneous, having been generated using 28 different enrichment kits by 42 different research groups across Europe partnering in the Solve-RD project. Each research group had previously undertaken their own analysis of the data but failed to identify disease-causing variants. We applied three CNV calling algorithms to maximise sensitivity, and rare CNVs overlapping genes of interest, provided by four partner European Reference Networks, were taken forward for interpretation by clinical experts. This reanalysis has resulted in a molecular diagnosis being provided to 51 families in this sample, with ClinCNV performing the best of the three algorithms. We also identified partially explanatory pathogenic CNVs in a further 34 individuals. This work illustrates the value of reanalysing ES cold cases for CNVs.

## Introduction

Rare diseases (RD) are defined in Europe as conditions that affect <1 in 2000 individuals. Nevertheless, it is estimated that more than 30 million people across the European Union are affected by one of ~6000–8000 different RDs^1,2^. As 80% of RD are expected to have a genetic aetiology, massively parallel sequencing approaches, in particular exome sequencing (ES), have been widely applied over the last decade to identify variants in DNA that cause RD. However, despite many advances in technology during this period, more than half of all individuals affected by an RD remain without a molecular diagnosis following such analyses, thus extending their diagnostic odyssey. While the accurate detection of single nucleotide variants (SNV) and short (<50nt) insertions and deletions (InDels) from ES data has become relatively robust in recent years^3^, the reliable detection of larger variants, including copy number variants (CNVs), remains a challenge, and it is likely that undetected pathogenic CNVs account for a proportion of undiagnosed individuals.

CNVs comprise losses, which may be heterozygous or homozygous in autosomes, or hemizygous in gonosomes, and gains of genetic material, which we refer to here as *deletions* and *duplications*, respectively. Identification of CNVs from short-read ES data (i.e. 100–150nt paired-end reads) is complicated by several factors, the most important of which being that read length is usually shorter than variant length, and that the boundaries of the CNV, referred to as breakpoints, are unlikely to be captured directly by the enrichment targets, since they represent only ~1–2% of the genome. An exacerbating factor is a marked variability in the enrichment process, in which targets for ~200,000 exons undergo DNA hybridisation and PCR amplification prior to sequencing, both between kits and between experiments. Many methods have been developed for CNV detection from ES data, most of which use the comparison of depth of coverage (DoC) between the observed number of reads covering a particular exon/target in a sample of interest and the normalised coverage for the same exon/target in a large reference batch of matched experimental samples^4–9^. For such methods to be successful, the sequencing data needs to be as homogenous as possible, particularly with respect to the evenness of coverage^10^, which is the key factor in CNV detection since it directly affects the signal-to-noise ratio.

As reviewed recently in Gordeeva et al.^11^, these methods differ from each other primarily in terms of the approach taken for read count normalisation, assumptions regarding read-depth distribution, and the segmentation process, i.e. identification of the boundaries of a variant. Despite the application of sophisticated normalisation techniques, the correct separation of the signal of true CNVs from background noise remains challenging, particularly for short CNVs that only impact one or a few exons. This is illustrated by numerous cross-tool comparisons in which the intersection of CNVs detected by different methods is limited, ranging from ~1–20% concordance when three or more tools are compared across samples^12–14^. Indeed, a recent benchmarking initiative involving sixteen tools showed that the number of raw CNVs called on a single ES sample ranged from just two to over a thousand^11^, reflecting differing optimisation of algorithms for specificity or sensitivity. Therefore, following identification of a list of potential CNVs, subsequent filtering steps are required, including determining which CNVs are technically valid (i.e. bona fide biological events), and whether any of the valid CNVs are of clinical relevance with respect to the phenotype of the affected individual. Hence, both technical expertise and expert clinical knowledge are required if disease-causing CNVs are to be correctly identified.

This complexity may explain why the detection of CNVs has often been omitted from diagnostic ES workflows, with array comparative genome hybridisation (aCGH) continuing to be the preferred method in the clinic over the last decade, despite limitations in its sensitivity and resolution, particularly with respect to short CNVs. However, recent studies have indicated that ES may be a suitable replacement as a first-tier diagnostic test^15–17^, with the added benefit that SNVs and InDels are detected simultaneously.

A key goal of the EU Horizon 2020 Solve-RD project is to raise the diagnostic rate of individuals with an RD for whom ES analysis and variant interpretation have previously been undertaken, but without a conclusive diagnosis having been reached. This is being achieved by undertaking massive pan-European data collation and complete reanalysis of raw data, followed by expert technical and clinical interpretation and validation of variants^18^. The CNV analysis conducted here, was an integral part of a larger re-analysis effort undertaken on the same dataset, covering most other variant types (Laurie et al.^19^). Here we describe the workflow applied in a comprehensive reanalysis of this heterogeneous sample of ES data from 9171 individuals pertaining to 5757 families, including 6143 individuals affected by an RD, to identify (likely) pathogenic CNVs. The ES data was generated using 28 different enrichment kits in multiple sequencing centres. Hence, to maximise the accuracy and sensitivity of CNV detection we applied three different algorithms, ClinCNV, Conifer, and ExomeDepth, and analysed experiments in 28 different batches, comprising data generated using the same enrichment kit. We filtered the raw call set, initially consisting of over two million CNV calls (average of ~300 per individual), to a manageable number of 0–2 potentially pathogenic rare CNVs per affected individual requiring interpretation by the clinical experts who submitted the cases to Solve-RD. This extensive endeavour has led to the closure of many diagnostic odysseys, some of which had been ongoing for decades, of which we provide some illustrative examples.

## Results

### Technical results

**Fig. 1:**
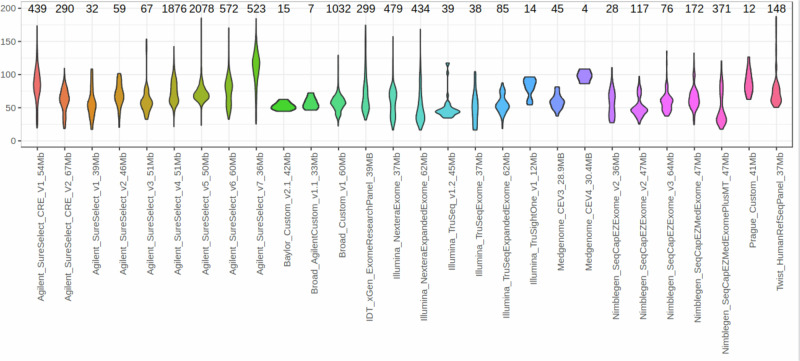
Violin plot of the median depth of coverage by kit for 9351 ES experiments pertaining to 28 different enrichment kits. The number of experiments pertaining to each kit is shown above the plots. Coverage is shown on the *Y*-axis. Thickness of the plotted shape indicates the proportion of experiments that have a particular coverage.

Prior to the initiation of CNV calling, minimal quality control was undertaken, which took the form of requiring that data from each submitted family included at least one affected individual with accompanying Human Phenotype Ontology (HPO) terms. Furthermore, following the alignment of sequencing reads, it was required that at least 70% of the target region of the enrichment kit had a depth of coverage (DoC) of ten reads. After the removal of 143 experiments that did not meet these criteria, CNV calling was undertaken on data from a total of 9171 individuals from 5757 families, of whom 6143 had a rare condition. Initial investigations indicated the presence of a large variance in sequencing depth both within and between the 28 enrichment kit batches, reflecting the heterogeneity of the sequencing data submitted to Solve-RD (Fig. 1).

Following the identification and removal of likely false positive calls based upon tool-specific QC metrics, the removal of commonly observed events, and restriction to events overlapping genes in the custom gene lists from the corresponding European Reference Network (ERN), a total of 7849 calls in 3436 affected individuals from 3300 families remained for interpretation (Table 1). The number of probands with at least one CNV call to be interpreted by clinical specialists from the ERN ranged from 113 for GENTURIS (33% of families) to 1239 for ITHACA (69% of families) (Supplementary Table 3). No CNV of interest was detected in 2707 affected individuals from the remaining 2457 families. In addition, a further 393 pairs of potential CNV-SNV *double-hit* compound heterozygous variants in 226 affected individuals were returned to clinical experts for interpretation. Overall, a mean of 1.3 CNVs per proband was returned for interpretation. However, as CNVs of potential interest were only identified in 55% of probands, this equated to 2.4 variants per proband that required interpretation.

**Table 1:** Table showing overall number of CNV calls submitted for clinical interpretation following filtering, separated by type and caller used

		Copy number						
Tool	Long	0	1	2	3	4	>4	Total
ClinCNV	248 (68)	283 (206)	1,203 (64)	99 (99)	776 (29)	145 (1)	28 (2)	2,782 (469)
Conifer	526 (14)	5 (4)	65 (0)	20 (20)	246 (5)	0 (0)	0 (0)	862 (43)
ExomeDepth	502 (31)	218 (28)	1342 (90)	38 (38)	1948 (64)	134 (4)	23 (9)	4,205 (264)
Total	1276 (113)	506 (238)	2610 (154)	157 (157)	2970 (98)	279 (5)	51 (11)	7849 (776)
% of Events	16.26	6.45	33.25	2.00	37.84	3.55	0.65	100

Numbers in brackets denote the subset of calls detected on sex chromosomes.

**Fig. 2:**
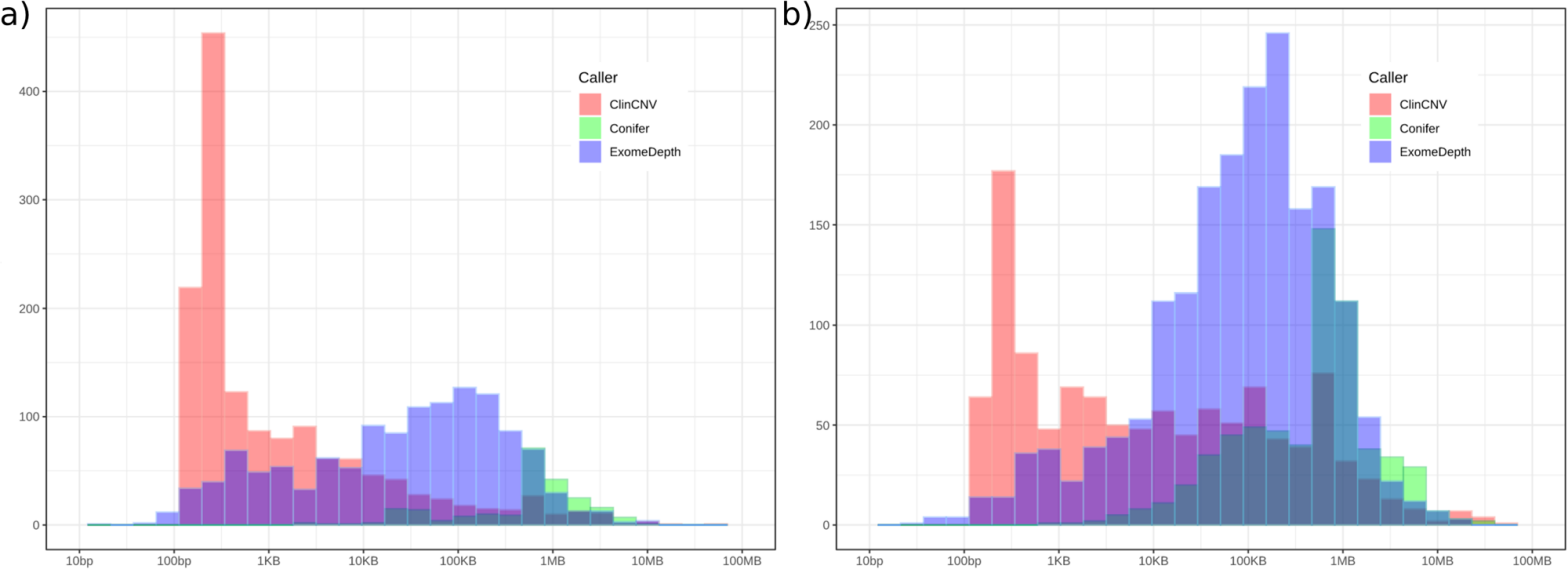
Distribution of lengths of 7849 CNV calls detected in 3436 affected individuals, separated into deletions (Panel a) and duplications (Panel b). The *x*-axis represents the length of calls identified (log_10_ scale), and the *y*-axis the number of events observed. Note that the *y*-axis scale is different in panel **a** from panel **b**.

The total number of CNV calls in affected individuals returned for interpretation was highest for ExomeDepth (*n* = 4205), while ClinCNV called about two-thirds of this number (2782), and Conifer approximately one-fifth (862), reflecting different predilections of the underlying algorithms with respect to sensitivity and specificity of CNV detection. While Conifer and ExomeDepth showed a significant bias toward calling duplications, the reverse pattern was observed for ClinCNV, which identified more deletions (*p* < 0.00001 in all cases, Fisher exact test; Supplementary Table 4). We assessed the distribution of the length of CNVs returned for interpretation as identified by each tool. Notably, the average length of CNVs detected by Conifer was approximately an order of magnitude larger than that of ExomeDepth, which in turn was longer than that of ClinCNV. This pattern held for both duplications and deletions and again reflects differences in the way the tools identify and segment CNVs (Fig. 2, Supplementary Table 5).

### Diagnostic results

**Fig. 3:**
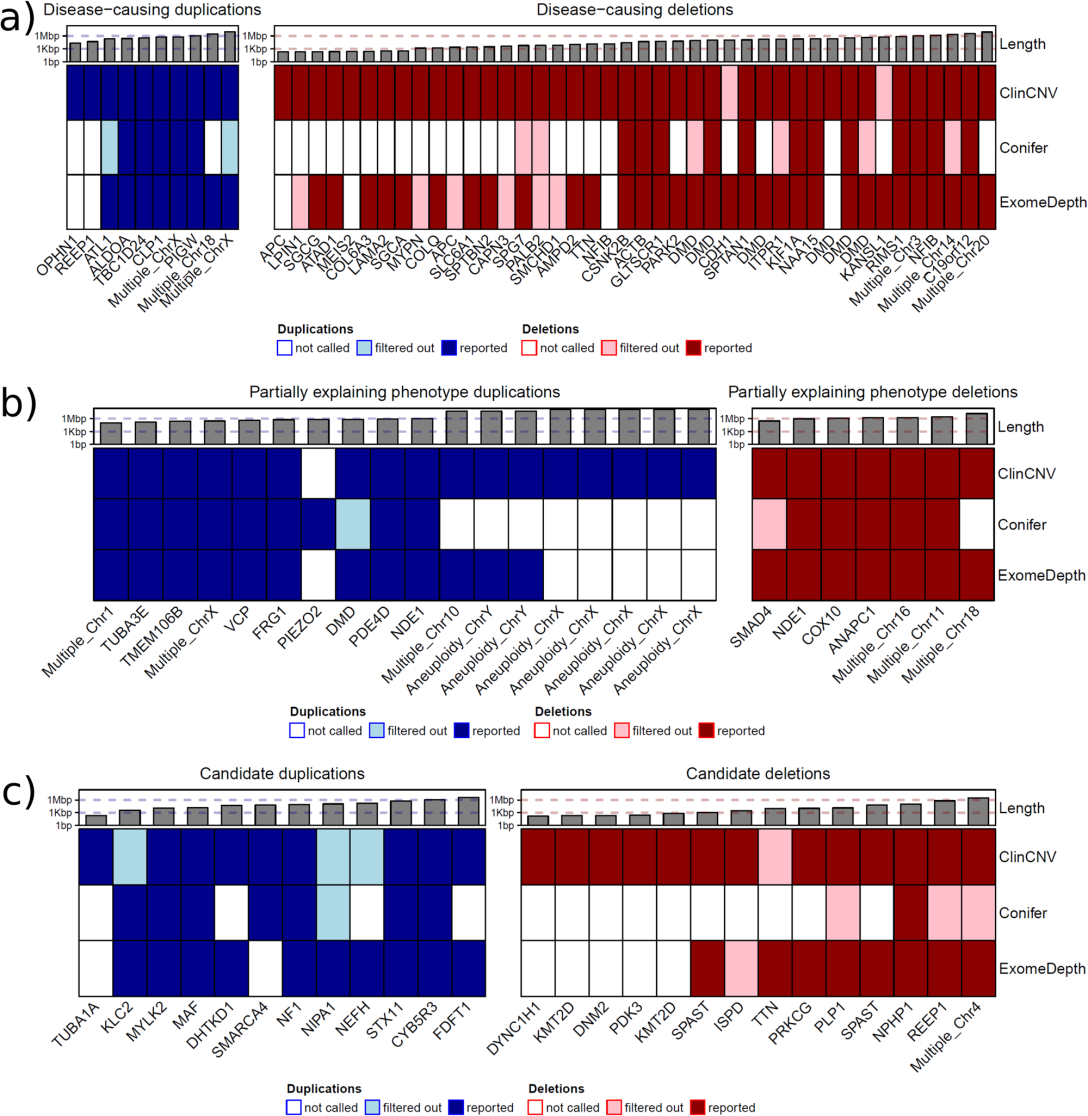
Heat maps illustrating the length of confirmed disease-causing CNVs (Panel a), partially explanatory disease-causing CNVs (Panel b) and candidate disease-causing CNVs (Panel c) identified in this study. Duplications are shown in blue, and deletions in red. Cyan and pink, represent duplication and deletion calls, respectively, which were initially QC filtered in the workflow for the respective tool, and identified post hoc. The approximate length of the event is indicated in the top layer using a log_10_ scale. The affected gene is indicated along the bottom. Where more than one gene was unaffected, it is shown as multiple, with the affected chromosome indicated.

Following expert interpretation, 105 potentially pathogenic CNVs of interest in 103 affected probands were identified, of which 52 have been confirmed as disease-causing in 51 individuals (Table 2). The disease-causing CNVs included three “double-hit” instances where an SNV and CNV affecting different alleles of the same gene were identified, resulting in a compound heterozygous diagnosis and one instance where two CNVs affecting different genes provided a dual genetic diagnosis for a complex phenotype. Parent–child trios account for 18 out of the 51 solved cases (35%), and 13 of these cases are caused by de novo CNVs. A further 25 CNVs are regarded as pathogenic by the clinical experts but not sufficient to explain the full phenotype observed in the affected individual, including seven complete gonosomal aneuploidies (“Partially explanatory” in Tables 2 and 3). A further 26 potentially pathogenic CNVs were identified for which further validation is not logistically possible due to lack of access to DNA and/or the patient (referred to as candidates below). While 81% (42 of 52) of confirmed disease-causing CNVs are deletions, only 39% (7 of 18) of the partially explanatory pathogenic CNVs are deletions, even when disregarding the gonosomal duplications. Of the 26 candidate CNVs, 54% (14) are deletions (Fig. 3 and Table 2).

**Table 2:** Table listing the 105 potentially pathogenic CNVs discovered in this study

Individual_ID	Family_ID	CaseID	Status	CNV/SNV	Variant_zygosity	Type	CN	Coordinates	Length	Gene(s)	Ensembl75_Gene_ID	ERN	Variant_in_HGVS_nomenclature	Clinvar ID	ClinVar_Position_HGVS	Number of P/LP CNVs in ClinVar that intersect with these coordinates [18.04]
P0002153	FAM0009777	3	Candidate	CNV	Heterozygous	DUP	3	10_12110981_12162938_DUP	51,957	DHTKD1	ENSG00000181192	EURO-NMD	NC_000010.10:g.(?_12110981)_(12162938_?)dup	154187	NC_000010.10:g.(?_100026)_(13247916_?)dup	>10
P0002506	FAM0009943	4	Candidate	CNV	Heterozygous	DEL	1	X_24521392_24521679_DEL	287	PDK3	ENSG00000067992	EURO-NMD	NC_000023.10:g.(?_24521392)_(24521679_?)del	146764	NC_000023.10:g.(?_60001)_(155260560_?)del	–
P0003100	FAM0010323	7	Candidate	CNV	Heterozygous	DUP	3	20_30409190_30421651_DUP	12,461	MYLK2	ENSG00000101306	EURO-NMD	NC_000020.10:g.(?_30409190)_(30421651_?)dup	146032	NC_000020.10:g.(?_29842786)_(32060886_?)dup	>10
P0003888	FAM0008256	9	Candidate	CNV	Heterozygous	DEL	1	2_85922037_86565199_DEL	643,162	REEP1	ENSG00000068615	EURO-NMD	NC_000002.11:g.(?_85922037)_(86565199_?)del	442633	NC_000002.11:g.(?_74365484)_(89129064_?)del	7
P0004077	FAM0008231	11	Candidate	CNV	Homozygous	DEL	0	19_54376781_54387499_DEL	10,718	PRKCG	ENSG00000126583	EURO-NMD	NC_000019.9:g.(?_54376781)_(54387499_?)[0]	253434	NC_000019.9:g.(?_54280799)_(54635178_?)del	1
P0005362	FAM0001210	14	Candidate	CNV	Heterozygous	DEL	1	2_179536648_179546515_DEL	9867	TTN	ENSG00000155657	EURO-NMD	NC_000002.11:g.(?_179536648)_(179546515_?)del	60248	NC_000002.11:g.(?_164821892)_(183059789_?)del	>10
P0005726	FAM0001326	16	Candidate	CNV	Heterozygous	DEL	1	7_16128729_16131473_DEL	2744	ISPD/CRPPA	ENSG00000214960	EURO-NMD	NC_000007.13:g.(?_16128729)_(16131473_?)del	148859	NC_000007.13:g.(?_15573437)_(24891051_?)del	>10
P0007842	FAM0007698	28	Candidate	CNV	Heterozygous	DUP	3	17_29421297_29509695_DUP	88,398	NF1	ENSG00000196712	ITHACA	NC_000017.10:g.(?_29421297)_(29509695_?)dup	58136	NC_000017.10:g.(?_28957752)_(30415399_?)dup	>10
P0009051	FAM0002446	30	Candidate	CNV	Heterozygous	DEL	1	12_49444872_49445072_DEL	200	KMT2D	ENSG00000167548	RND	NC_000012.11:g.(?_49444872)_(49445072_?)del	–	–	1
P0009060	FAM0002452	31	Candidate	CNV	Heterozygous	DEL	1	12_49445280_49445945_DEL	665	KMT2D	ENSG00000167548	RND	NC_000012.11:g.(?_49445280)_(49445945_?)del	–	–	1
P0010706	FAM0003484	41	Candidate	CNV	Heterozygous	DEL	1	19_10934412_10934626_DEL	214	DNM2	ENSG00000079805	EURO-NMD	NC_000019.9:g.(?_10934412)_(10934626_?)del	146077	NC_000019.9:g.(?_9846119)_(11338677_?)del	5
P0011100	FAM0003747	44	Candidate	CNV	Heterozygous	DEL	1	2_32312464_32372390_DEL	59,926	SPAST	ENSG00000021574	RND	NC_000002.11:g.(?_32312464)_(32372390_?)del	60107	NC_000002.11:g.(?_31591498)_(32312698_?)del	>10
P0011134	FAM0003776	45	Candidate	CNV	Heterozygous	DEL	1	2_32339721_32340871_DEL	1150	SPAST	ENSG00000021574	RND	NC_000002.11:g.(?_32339721)_(32340871_?)del	60107	NC_000002.11:g.(?_31591498)_(32312698_?)del	10
P0012337	FAM0004586	49	Candidate	CNV	Heterozygous	DUP	4	19_11039687_11105889_DUP	66,202	SMARCA4	ENSG00000127616	ITHACA	NC_000019.9:g.(?_11039687)_(11105889_?)[4]	59110	NC_000019.9:g.(?_8941823)_(13442041_?)dup	5
P0012447	FAM0004642	50	Candidate	CNV	Heterozygous	DUP	3	12_49580141_49580343_DUP	202	TUBA1A	ENSG00000167552	RND	NC_000012.11:g.(?_49580141)_(49580343_?)dup	150740	NC_000012.11:g.(?_282465)_(133773393_?)dup	6
P0015656	FAM0008419	70	Candidate	CNV	Heterozygous	DUP	3	11_66031070_66034990_DUP	3920	KLC2	ENSG00000174996	RND	NC_000011.9:g.(?_66031070)_(66034990_?)dup	59757	NC_000011.9:g.(?_65960973)_(67658241_?)dup	8
P0015855	FAM0008489	73	Candidate	CNV	Heterozygous	DUP	3	16_79619708_79633852_DUP	14,144	MAF	ENSG00000178573	RND	NC_000016.9:g.(?_79619708)_(79633852_?)dup	154511	NC_000016.9:g.(?_64423281)_(90148393_?)dup	>10
P0017644	FAM0007321	84	Candidate	CNV	Heterozygous	DUP	3	6_144612964_145161968_DUP	549,004	STX11	ENSG00000135604	ITHACA	NC_000006.11:g.(?_144612964)_(145161968_?)dup	147665	NC_000006.11:g.(?_135679288)_(155776251_?)dup	>10
P0019474	FAM0009178	88	Candidate	CNV	Homozygous	DEL	0	2_110855123_110962791_DEL	107,668	NPHP1	ENSG00000144061	RND	NC_000002.11:g.(?_110855123)_(110962791_?)[0]	57545	NC_000002.11:g.(?_110783236)_(111128847_?)del	>10
P0019717	FAM0009787	89	Candidate	CNV	Heterozygous	DEL	1	14_102478650_102478806_DEL	156	DYNC1H1	ENSG00000197102	EURO-NMD	NC_000014.8:g.(?_102478650)_(102478806_?)del	146793	NC_000014.8:g.(?_95990744)_(107287708_?)del	>10
P0021122	FAM0011359	93	Candidate	CNV	Heterozygous	DUP	3	8_8098277_11725590_DUP	3,627,313	FDFT1	ENSG00000079459	ITHACA	NC_000008.10:g.(?_8098277)_(11725590_?)dup	152873	NC_000008.10:g.(?_11298481)_(11841842_?)dup	>10
P0021571	FAM0011780	94	Candidate	CNV	Heterozygous	DUP	3	22_42781153_43870829_DUP	1,089,676	CYB5R3	ENSG00000100243	RND	NC_000022.10:g.(?_42781153)_(43870829_?)dup	57944	NC_000022.10:g.(?_42995763)_(51163669_?)dup	>10
P0021588	FAM0011796	95	Candidate	CNV	Heterozygous	DUP	3	15_23021145_23140413_DUP	119,268	NIPA1	ENSG00000170113	RND	NC_000015.9:g.(?_23021145)_(23140413_?)dup	58531	NC_000015.9:g.(?_20085002)_(29210720_?)dup	>10
P0021625	FAM0011830	97	Candidate	CNV	Heterozygous	DEL	1	X_103031767_103045531_DEL	13,764	PLP1	ENSG00000123560	RND	NC_000023.10:g.(?_103031767)_(103045531_?)del	146764	NC_000023.10:g.(?_60001)_(155260560_?)del	–
P0021628	FAM0011833	98	Candidate	CNV	Heterozygous	DEL	1	4_103553260_106891654_DEL	3,338,394	MANBA;CISD2;PPA2	Multiple	RND	NC_000004.11:g.(?_103553260)_(106891654_?)del	–	–	6
P0022254	FAM0012220	103	Candidate	CNV	Heterozygous	DUP	3	22_29696076_29876767_DUP	180,691	NEFH	ENSG00000100285	EURO-NMD	NC_000022.10:g.(?_29696076)_(29876767_?)dup	145336	NC_000022.10:g.(?_17397633)_(51178213_?)dup	>10
P0000914	FAM0006341	1	Disease-causing	CNV	Heterozygous	DEL	1	9_131295791_131419128_DEL	123,337	SPTAN1	ENSG00000197694	EURO-NMD	NC_000009.11:g.(?_131295791)_(131419128_?)del	148721	NC_000009.11:g.(?_127818144)_(131400225_?)del	8
P0001253	FAM0009337	2	Disease-causing	CNV	Hemizygous	DEL	0	X_31947661_32053731_DEL	106,070	DMD	ENSG00000198947	EURO-NMD	NC_000023.10:g.(?_31947661)_(32053731_?)[0]	147348	NC_000023.10:g.(?_30112028)_(34078784_?)del	–
P0002519	FAM0000188	5	Disease-causing	CNV	Heterozygous	DEL	1	15_42681074_42684971_DEL	3897	CAPN3	ENSG00000092529	EURO-NMD	NC_000015.9:g.(?_42681074)_(42684971_?)del	2579221	NC_000015.9:g.(?_42641534)_(42671130_?)del	2
P0002690	FAM0010002	6	Disease-causing	CNV	Heterozygous	DEL	1	X_31893253_32053731_DEL	160,478	DMD	ENSG00000198947	EURO-NMD	NC_000023.10:g.(?_31893253)_(32053731_?)del	160983	NC_000023.10:g.(?_60679)_(155242832_?)del	–
P0003633	FAM0000707	8	Disease-causing	CNV	Heterozygous	DEL	1	X_32429817_32867988_DEL	438,171	DMD	ENSG00000198947	EURO-NMD	NC_000023.10:g.(?_32429817)_(32867988_?)del	160983	NC_000023.10:g.(?_60679)_(155242832_?)del	–
P0003891	FAM0001526	10	Disease-causing	CNV	Homozygous	DEL	0	1_110163633_110173775_DEL	10,142	AMPD2	ENSG00000116337	EURO-NMD	NC_000001.10:g.(?_110163633)_(110173775_?)[0]	60005	NC_000001.10:g.(?_97876158)_(111213132_?)del	8
P0004907	FAM0010435	13	Disease-causing	CNV	Heterozygous	DEL	1	X_32632368_32867988_DEL	235,620	DMD	ENSG00000198947	EURO-NMD	NC_000023.10:g.(?_32632368)_(32867988_?)del	160983	NC_000023.10:g.(?_60679)_(155242832_?)del	–
P0005481	FAM0001265	15	Disease-causing	CNV	Homozygous	DEL	0	13_23853446_23853668_DEL	222	SGCG	ENSG00000102683	EURO-NMD	NC_000013.10:g.(?_23853446)_(23853668_?)[0]	146305	NC_000013.10:g.(?_19020001)_(115085141_?)del	8
P0005861	FAM0010571	18	Disease-causing	CNV	Heterozygous	DEL	1	18_2795896_2802599_DEL	6703	SMCHD1	ENSG00000101596	EURO-NMD	NC_000018.9:g.(?_2795896)_(2802599_?)del	153128	NC_000018.9:g.(?_48782)_(14978075_?)del	>10
P0005947	FAM0010587	20	Disease-causing	CNV	Heterozygous	DEL	1	6_129674257_129674553_DEL	296	LAMA2	ENSG00000196569	EURO-NMD	NC_000006.11:g.(?_129674257)_(129674553_?)del	57486	NC_000006.11:g.(?_109266102)_(132388860_?)del	7
P0006025	FAM0001383	21	Disease-causing	CNV	Homozygous	DEL	0	17_48247452_48247763_DEL	311	SGCA	ENSG00000108823	EURO-NMD	NC_000017.10:g.(?_48247452)_(48247763_?)[0]	59589	NC_000017.10:g.(?_47215226)_(50225170_?)del	4
P0006355	FAM0001683	22	Disease-causing	CNV	Homozygous	DEL	0	3_15529661_15531195_DEL	1534	COLQ	ENSG00000206561	EURO-NMD	NC_000003.11:g.(?_15529661)_(15531195_?)[0]	–	–	1
P0006523	FAM0010645	23	Disease-causing	CNV	Homozygous	DEL	0	2_11959558_11959775_DEL	217	LPIN1	ENSG00000134324	EURO-NMD	NC_000002.11:g.(?_11959558)_(11959775_?)[0]	60105	NC_000002.11:g.(?_6671304)_(16243921_?)del	2
P0007326	FAM0007602	25	Disease-causing	CNV	Homozygous	DEL	0	10_89549991_89550223_DEL	232	ATAD1	ENSG00000138138	ITHACA	NC_000010.10:g.(?_89549991)_(89550223_?)[0]	661198	NC_000010.10:g.(?_88514773)_(89725239_?)del	7
P0008231	FAM0002064	29	Disease-causing	CNV	Heterozygous	DEL	1	X_32456306_32536299_DEL	79,993	DMD	ENSG00000198947	EURO-NMD	NC_000023.10:g.(?_32456306)_(32536299_?)del	160983	NC_000023.10:g.(?_60679)_(155242832_?)del	–
P0009136	FAM0002525	34	Disease-causing	CNV	Heterozygous	DEL	1	5_112173249_112173448_DEL	199	APC	ENSG00000134982	GENTURIS	NC_000005.9:g.(?_112173249)_(112173448_?)del	495348	NC_000005.9:g.(?_112036100)_(?_112045850)del	>10
P0009225	FAM0002614	35	Disease-causing	CNV	Heterozygous	DEL	1	5_112175002_112177352_DEL	2350	APC	ENSG00000134982	GENTURIS	NC_000005.9:g.(?_112175002)_(112177352_?)del	495348	NC_000005.9:g.(?_112036100)_(?_112045850)del	>10
P0009735	FAM0002877	39	Disease-causing	CNV	Homozygous	DEL	0	16_89611055_89617017_DEL	5962	SPG7	ENSG00000197912	RND	NC_000016.9:g.(?_89611055)_(89617017_?)[0]	59538	NC_000016.9:g.(?_88706524)_(89596883_?)del	2
P0010944	FAM0003678	42	Disease-causing	CNV	Heterozygous	DEL	1	2_241737061_241932645_DEL	195,584	KIF1A	ENSG00000130294	RND	NC_000002.11:g.(?_241737061)_(241932645_?)del	161051	NC_000002.11:g.(?_232634989)_(243059659_?)del	>10
P0011003	FAM0003709	43	Disease-causing	CNV	Heterozygous	DUP	3	2_86459682_86509481_DUP	49,799	REEP1	ENSG00000068615	RND	NC_000002.11:g.(?_86459682)_(86509481_?)dup	145403	NC_000002.11:g.(?_77252342)_(91619262_?)dup	5
P0011213	FAM0003850	46	Disease-causing	CNV	Heterozygous	DEL	1	11_66472691_66475714_DEL	3023	SPTBN2	ENSG00000173898	RND	NC_000011.9:g.(?_66472691)_(66475714_?)del	154814	NC_000011.9:g.(?_65508902)_(67473140_?)del	1
P0011479	FAM0004055	47	Disease-causing	CNV	Hemizygous	DEL	0	X_31697440_32053731_DEL	356,291	DMD	ENSG00000198947	EURO-NMD	NC_000023.10:g.(?_31697440)_(32053731_?)[0]	147348	NC_000023.10:g.(?_30112028)_(34078784_?)del	–
P0011750	FAM0004194	48	Disease-causing	CNV	Heterozygous	DUP	4	14_50911699_51132124_DUP	220,425	ATL1	ENSG00000198513	RND	NC_000014.8:g.(?_50911699)_(51132124_?)[4]	152063	NC_000014.8:g.(?_39665376)_(57181179_?)dup	7
P0012480	FAM0004812	51	Disease-causing	CNV-DNM	Heterozygous	DUP	4	16_2229815_2582030_DUP	352,215	TBC1D24	ENSG00000162065	ITHACA	NC_000016.9:g.(?_2229815)_(2582030_?)[4]	58594	NC_000016.9:g.(?_73141)_(11390552_?)dup	>10
P0012545	FAM0004885	52	Disease-causing	CNV-DNM	Heterozygous	DEL	1	9_13927869_15424029_DEL	1496160	NFIB	ENSG00000147862	ITHACA	NC_000009.11:g.(?_13927869)_(15424029_?)del	60415	NC_000009.11:g.(?_111216)_(14650760_?)del	>10
P0012573	FAM0004950	54	Disease-causing	CNV-DNM	Heterozygous	DUP	4	18_158412_2960886_DUP	2,802,474	TGIF1;LAMA1;NDUFV2;PIEZO2;AFG3L2	Multiple	ITHACA	NC_000018.9:g.(?_158412)_(2960886_?)[4]	155367	NC_000018.9:g.(?_136226)_(15198990_?)dup	>10
P0012635	FAM0005017	55	Disease-causing	CNV	Heterozygous	DUP	3	X_154124335_154736815_DUP	612,480	RAB39B;TMLHE;CLIC2	Multiple	ITHACA	NC_000023.10:g.(?_154124335)_(154736815_?)dup	160897	NC_000023.10:g.(?_60679)_(155251871_?)dup	–
P0012660	FAM0005044	56	Disease-causing	CNV	Heterozygous	DEL	1	3_9974258_11078781_DEL	1,104,523	FANCD2;SLC6A1	Multiple	ITHACA	NC_000003.11:g.(?_9974258)_(11078781_?)del	153284	NC_000003.11:g.(?_61891)_(11263288_?)del	>10
P0012662	FAM0005046	57	Disease-causing	CNV	Heterozygous	DUP	3	11_57003258_57596656_DUP	593,398	CLP1	ENSG00000172409	ITHACA	NC_000011.9:g.(?_57003258)_(57596656_?)dup	154690	NC_000011.9:g.(?_55086995)_(58766250_?)dup	7
P0012737	FAM0005123	59	Disease-causing	CNV	Heterozygous	DUP	4	X_46626489_56455293_DUP	9,828,804	RBM10;SYN1;FTSJ1;PORCN;EBP;HDAC6;PQBP1;SLC35A2;OTUD5;TFE3;WDR45;SYP;CCDC22;USP27X;SHROOM4;KDM5C;IQSEC2;SMC1A;HSD17B10;HUWE1;PHF8;FGD1	Multiple	ITHACA	NC_000023.10:g.(?_46626489)_(56455293_?)[4]	144172	NC_000023.10:g.(?_60679)_(155252491_?)dup	–
P0012861	FAM0005243	60	Disease-causing	CNV-DNM	Heterozygous	DEL	1	19_48185250_48245216_DEL	59,966	GLTSCR1/BICRA	ENSG00000063169	ITHACA	NC_000019.9:g.(?_48185250)_(48245216_?)del	60103	NC_000019.9:g.(?_46961379)_(48186836_?)del	3
P0012931	FAM0005537	61	Disease-causing	CNV-DNM	Heterozygous	DEL	1	6_31630124_31657924_DEL	27,800	CSNK2B	ENSG00000204435	ITHACA	NC_000006.11:g.(?_31630124)_(31657924_?)del	–	–	0
P0013033	FAM0005409	63	Disease-causing	CNV1of2	Heterozygous	DUP	3	16_29624260_29874118_DUP	249,858	ALDOA	ENSG00000149925	ITHACA	NC_000016.9:g.(?_29624260)_(29874118_?)dup	60446	NC_000016.9:g.(?_28377432)_(30194753_?)dup	>10
P0013033	FAM0005409	63	Disease-causing	CNV1of2	Heterozygous	DUP	3	17_34842442_36065085_DUP	1,222,643	PIGW	ENSG00000277161	ITHACA	NC_000017.10:g.(?_34842442)_(36065085_?)dup	58166	NC_000017.9:g.(?_31335111)_(33373530_?)dup	>10
P0013051	FAM0005425	64	Disease-causing	CNV-DNM	Heterozygous	DEL	1	19_29567062_32902357_DEL	3,335,295	C19orf12	ENSG00000131943	ITHACA	NC_000019.9:g.(?_29567062)_(32902357_?)del	153639	NC_000019.9:g.(?_29542795)_(32458502_?)del	5
P0013071	FAM0005602	66	Disease-causing	CNV-DNM	Heterozygous	DEL	1	6_71998625_72678833_DEL	680,208	RIMS1	ENSG00000079841	ITHACA	NC_000006.11:g.(?_71998625)_(72678833_?)del	154460	NC_000006.11:g.(?_65259548)_(84136510_?)del	6
P0014615	FAM0004765	67	Disease-causing	CNV	Heterozygous	DEL	1	16_68846035_68961985_DEL	115,950	CDH1	ENSG00000039068	GENTURIS	NC_000016.9:g.(?_68846035)_(68961985_?)del	417390	NC_000016.9:g.(?_68771195)_(68772314_?)del	2
P0015418	FAM0006080	68	Disease-causing	CNV	Heterozygous	DEL	1	2_179448320_179462531_DEL	14,211	TTN	ENSG00000155657	EURO-NMD	NC_000002.11:g.(?_179448320)_(179462531_?)del	60248	NC_000002.11:g.(?_164821892)_(183059789_?)del	>10
P0015586	FAM0006143	69	Disease-causing	CNV	Heterozygous	DEL	1	3_4669445_4859925_DEL	190,480	ITPR1	ENSG00000150995	RND	NC_000003.11:g.(?_4669445)_(4859925_?)del	153284	NC_000003.11:g.(?_61891)_(11263288_?)del	>10
P0015673	FAM0008430	71	Disease-causing	CNV	Heterozygous	DEL	1	3_11076181_11078707_DEL	2526	SLC6A1	ENSG00000157103	RND	NC_000003.11:g.(?_11076181)_(11078707_?)del	153284	NC_000003.11:g.(?_61891)_(11263288_?)del	>10
P0015720	FAM0008452	72	Disease-causing	CNV	Heterozygous	DEL	1	4_140187697_140394334_DEL	206,637	NAA15	ENSG00000164134	RND	NC_000004.11:g.(?_140187697)_(140394334_?)del	59479	NC_000004.11:g.(?_117552018)_(146351052_?)del	6
P0016422	FAM0006790	74	Disease-causing	CNV	Heterozygous	DEL	1	20_5454270_13610745_DEL	8,156,475	PLCB1;SNAP25;MKKS	Multiple	RND	NC_000020.10:g.(?_5454270)_(13610745_?)del	57236	NC_000020.10:g.(?_6317254)_(8558193_?)del	>10
P0016927	FAM0007063	78	Disease-causing	CNV	Heterozygous	DEL	1	16_23619233_23625407_DEL	6174	PALB2	ENSG00000083093	GENTURIS	NC_000016.9:g.(?_23619233)_(23625407_?)del	58734	NC_000016.9:g.(?_21612313)_(28334665_?)del	6
P0017508	FAM0007186	83	Disease-causing	CNV-DNM	Heterozygous	DEL	1	7_5521357_5569119_DEL	47,762	ACTB	ENSG00000075624	ITHACA	NC_000007.13:g.(?_5521357)_(5569119_?)del	58492	NC_000007.13:g.(?_45130)_(5920006_?)del	10
P0017993	FAM0010911	85	Disease-causing	CNV	Homozygous	DEL	0	2_238234151_238234418_DEL	267	COL6A3	ENSG00000163359	EURO-NMD	NC_000002.11:g.(?_238234151)_238234418_?)[0]	161051	NC_000002.11:g.(?_232634989)_(243059659_?)del	>10
P0018002	FAM0010913	86	Disease-causing	CNV	Homozygous	DEL	0	10_69933771_69935269_DEL	1498	MYPN	ENSG00000138347	EURO-NMD	NC_000010.10:g.(?_69933771)_69935269_?)[0]	147477	NC_000010.10:g.(?_65162339)_(77055857_?)del	6
P0019280	FAM0009101	87	Disease-causing	CNV-DNM	Heterozygous	DEL	1	9_14088188_14102587_DEL	14,399	NFIB	ENSG00000147862	ITHACA	NC_000009.11:g.(?_14088188)_(14102587_?)del	60415	NC_000009.11:g.(?_111216)_(14650760_?)del	>10
P0021613	FAM0011818	96	Disease-causing	CNV	Heterozygous	DEL	1	6_162622080_162683770_DEL	61,690	PARK2/PRKN	ENSG00000185345	RND	NC_000006.11:g.(?_162622080)_(162683770_?)del	536459	NC_000006.11:g.(?_162206784)_(162394469_?)del	>10
P0021951	FAM0012039	99	Disease-causing	CNV	Heterozygous	DEL	1	15_37188738_37188988_DEL	250	MEIS2	ENSG00000134138	ITHACA	NC_000015.9:g.(?_37188738)_(37188988_?)del	146702	NC_000015.9:g.(?_22698522)_(38381783_?)del	10
P0021980	FAM0012053	100	Disease-causing	CNV	Heterozygous	DEL	1	14_54866611_57272174_DEL	2,405,563	GCH1;OTX2	Multiple	RND	NC_000014.8:(?_54866611)_(57272174_?)del	146623	NC_000014.8:g.(?_52011564)_(55787316_?)del	>10
P0021982	FAM0012054	101	Disease-causing	CNV	Hemizygous	DUP	2	X_67433703_67454430_DUP	20,727	OPHN1	ENSG00000079482	RND	NC_000023.10:g.(?_67433703)_(67454430_?)dup	147683	NC_000023.10:g.(?_2700316)_(154785891_?)dup	–
P0021987	FAM0012059	102	Disease-causing	CNV	Heterozygous	DEL	1	17_44248221_44772028_DEL	523,807	KANSL1	ENSG00000120071	RND	NC_000017.10:g.(?_44248221)_(44772028_?)del	148960	NC_000017.10:g.(?_43593476)_(44224221_?)del	>10
P0002519	FAM0000188	5	NA	SNV	Heterozygous	NA	NA	15_42703181_42703180_-/TC	NA	CAPN3	ENSG00000092529	EURO-NMD	NC_000015.9:g.42703181_42703180insTC			–
P0005726	FAM0001326	16	NA	SNV	Heterozygous	NA	NA	7_16415796_16415796_G/A	NA	ISPD/CRPPA	ENSG00000214960	EURO-NMD	NC_000007.13:g.16415796G>A			–
P0005947	FAM0010587	20	NA	SNV	Heterozygous	NA	NA	6_129609205_129609204_-/T	NA	LAMA2	ENSG00000196569	EURO-NMD	NC_000006.11:g.129609205_129609204insT			–
P0007185	FAM0007805	24	NA	SNV	Heterozygous	NA	NA	7_70233042_70233041_-/CTA	NA	AUTS2	ENSG00000158321	ITHACA	NC_000007.13:g.70233042_70233041insCTA			–
P0010706	FAM0003484	41	NA	SNV	Heterozygous	NA	NA	19_10908150_10908149_-/G	NA	DNM2	ENSG00000079805	EURO-NMD	NC_000019.9:g.10908150_10908149insG	–	–	–
P0012708	FAM0005093	58	NA	SNV-DNM	Heterozygous	NA	NA	16_2815056_2815057_TC/-	NA	SRRM2	ENSG00000167978	ITHACA	NC_000016.9:g.2815056_2815057del			–
P0017358	FAM0007141	81	NA	SNV	Heterozygous	NA	NA	3_9495428_9495428_G/T	NA	SETD5	ENSG00000168137	ITHACA	NC_000003.11:g.9495428G>T			–
P0021613	FAM0011818	96	NA	SNV	Heterozygous	NA	NA	6_162864411_162864412_CT/-	NA	PARK2/PRKN	ENSG00000185345	RND	NC_000006.11:g.162864411_162864412del			–
P0004123	FAM0008287	12	Partially explanatory	CNV	Heterozygous	DUP	3	10_81196342_135267946_DUP	54,071,604	ANXA11;LDB3;ANKRD1;ALDH18A1;ENTPD1;ZFYVE27;COX15;ERLIN1;CWF19L1;C10orf2/TWNK;GBF1;NT5C2;RBM20;BAG3;NKX6-2	Multiple	EURO-NMD	NC_000010.10:g.(?_81196342)_(135267946_?)dup			>10
P0005756	FAM0001477	17	Partially explanatory	CNV	Heterozygous	DEL	1	18_48889_14852528_DEL	14,803,639	SMCHD1;PIEZO2;AFG3L2	Multiple	EURO-NMD	NC_000018.9:g.(?_48889)_(14852528_?)del			>10
P0005942	FAM0010582	19	Partially explanatory	CNV	Heterozygous	DUP	3	9_34729354_35107435_DUP	378,081	VCP	ENSG00000165280	EURO-NMD	NC_000009.11:g.(?_34729354)_(35107435_?)dup			>10
P0007185	FAM0007805	24	Partially explanatory	CNV	Heterozygous	DEL	1	16_15489788_16410082_DEL	920,294	NDE1	ENSG00000072864	ITHACA	NC_000016.9:g.(?_15489788)_(16410082_?)del			>10
P0007761	FAM0007818	26	Partially explanatory	Aneuploidy	Aneuploid	DUP	3	47,XXX	NA	Multiple	Multiple	ITHACA	NC_000023.10:g.pter_qter[3]			–
P0007820	FAM0007610	27	Partially explanatory	CNV	Heterozygous	DUP	3	16_15248224_16349639_DUP	1,101,415	NDE1	ENSG00000072864	ITHACA	NC_000016.9:g.(?_15248224)_(16349639_?)dup			>10
P0009070	FAM0008031	32	Partially explanatory	CNV	Heterozygous	DUP	3	5_57750426_58513073_DUP	762,647	PDE4D	ENSG00000113448	RND	NC_000005.9:g.(?_57750426)_(58513073_?)dup			5
P0009495	FAM0002734	37	Partially Explanatory	CNV	Heterozygous	DUP	3	4_190396051_190963305_DUP	567,254	FRG1	ENSG00000109536	ITHACA	NC_000004.11:g.(?_190396051)_(190963305_?)dup			>10
P0009589	FAM0002767	38	Partially explanatory	CNV	Heterozygous	DUP	3	18_11065984_11655038_DUP	589,054	PIEZO2	ENSG00000154864	ITHACA	NC_000018.9:g.(?_11065984)_(11655038_?)dup			>10
P0009943	FAM0007904	40	Partially explanatory	CNV	Heterozygous	DEL	1	11_44125177_46644454_DEL	2,519,277	ALX4;EXT2;PHF21A;SLC35C1;PEX16	Multiple	ITHACA	NC_000011.9:g.(?_44125177)_(46644454_?)del			10
P0012547	FAM0004889	53	Partially explanatory	CNV-DNM	Heterozygous	DEL	1	16_28426101_30199851_DEL	1,773,750	PRRT2;ALDOA	Multiple	ITHACA	NC_000016.9:g.(?_28426101)_(30199851_?)del			>10
P0012708	FAM0005093	58	Partially explanatory	CNV	Heterozygous	DUP	3	1_145414683_145515896_DUP	101,213	POLR3GL;RBM8A	Multiple	ITHACA	NC_000001.10:g.(?_145414683)_(145515896_?)dup			>10
P0012932	FAM0005312	62	Partially explanatory	Aneuploidy	Aneuploid	DUP	2	47,XYY	NA	Multiple	Multiple	ITHACA	NC_000024.9:g.pter_qter[2]			–
P0013060	FAM0005678	65	Partially explanatory	Aneuploidy	Aneuploid	DUP	2	47,XXY	NA	Multiple	Multiple	ITHACA	NC_000023.10:g.pter_qter[2]			–
P0016495	FAM0006844	75	Partially explanatory	CNV	Hemizygous	DUP	2	X_153170463_153453587_DUP	283,124	AVPR2;HCFC1;MECP2;NAA10	Multiple	ITHACA	NC_000023.10:g.(?_153170463)_(153453587_?)dup			–
P0016555	FAM0006885	76	Partially explanatory	CNV	Heterozygous	DUP	3	2_130897038_131132287_DUP	235,249	TMEM106B	ENSG00000106460	ITHACA	NC_000007.13:g.(?_130897038)_(131132287_?)dup			5
P0016555	FAM0006885	76	Partially explanatory	CNV	Heterozygous	DUP	4	7_12269989_12433449_DUP	163,460	TUBA3E	ENSG00000152086	ITHACA	NC_000002.11:g.(?_12269989)_(12433449_?)[4]			>10
P0016613	FAM0006927	77	Partially explanatory	CNV	Heterozygous	DEL	1	2_111395546_113157372_DEL	1,761,826	ANAPC1	ENSG00000153107	ITHACA	NC_000002.11:g.(?_111395546)_(113157372_?)del			>10
P0016996	FAM0007304	79	Partially explanatory	Aneuploidy	Aneuploid	DUP	3	47,XXX	NA	Multiple	Multiple	ITHACA	NC_000023.10:g.pter_qter[3]			–
P0017021	FAM0007286	80	Partially explanatory	Aneuploidy	Aneuploid	DUP	2	47,XXY	NA	Multiple	Multiple	ITHACA	NC_000023.10:g.pter_qter[2]			–
P0017358	FAM0007141	81	Partially explanatory	CNV-DNM	Heterozygous	DEL	1	17_14095281_15477522_DEL	1,382,241	COX10	ENSG00000006695	ITHACA	NC_000017.10:g.(?_14095281)_(15477522_?)del			>10
P0017437	FAM0007167	82	Partially explanatory	Aneuploidy	Aneuploid	DUP	2	47,XYY	NA	Multiple	Multiple	ITHACA	NC_000024.9:g.pter_qter[2]			–
P0020413	FAM0010991	90	Partially Explanatory	CNV	Heterozygous	DEL	1	18_48434432_48723514_DEL	289,082	SMAD4	ENSG00000141646	RND	NC_000018.9:g.(?_48434432)_(48723514_?)del			5
P0020456	FAM0011036	91	Partially explanatory	CNV	Heterozygous	DUP	3	X_32404426_33038317_DUP	633891	DMD	ENSG00000198947	EURO-NMD	NC_000023.10:g.(?_32404426)_(33038317_?)dup			–
P0021091	FAM0011328	92	Partially explanatory	Aneuploidy	Aneuploid	DUP	2	47,XXY	NA	Multiple	Multiple	ITHACA	NC_000023.10:g.pter_qter[2]			–

52 of the CNVs have been confirmed as disease causing, 25 as pathogenic, but not sufficient to explain the full phenotype of the affected individual, and 26 CNVs which are potentially pathogenic but have not been validated are considered as candidates. The table also includes the co-ordinates of 8 SNVs, which form a compound-heterozygote pairing with a CNV in the same gene from the same individual resulting in disease. *CN* Copy Number, *ERN* European Reference Network, *P/LP* Pathogenic/Likely Pathogenic

**Table 3:** Table showing success rates in identification of pathogenic CNVs from each of the four ERNs (European reference Networks)

ERN	Solved	Partially solved (families)		Candidates	Total	Pathogenic CNV	Solved families
	Families	Sex chromosome aneuploidies	Other	Families	Families	%	%
EURO-NMD	18	0	4	10	1.461	1.5	1.2
GENTURIS	4	0	0	0	340	1.2	1.2
RND	13	0	2	12	2.168	0.7	0.6
ITHACA	16	7	12	4	1.788	2.0	0.9
Totals	51	7	18	26	5.757	1.3	0.9

The table shows the number and proportion of families found to have disease-causing variants which fully or partially explain the affected individual's phenotype, and how many have candidate CNVs requiring further invetigation.

Of the 77 confirmed pathogenic CNVs, 40 (52%) were initially identified by all three callers (Fig. 3 and Table 2). However, in the case of ten of the 40, the Conifer call was subsequently discarded due to it being below the applied SV-RPKM threshold, and one of the ten was also discarded by the ExomeDepth workflow due to a low BF. Of the remaining 37 pathogenic CNVs, 36 (97%) were identified by ClinCNV, two of which subsequently failed ClinCNV quality control thresholds, while 25 (68%) were identified by ExomeDepth, five of which were subsequently discarded due to a low BF. Interestingly one of the 37, a duplication in *PIEZO2* was identified by Conifer alone.

Below we provide an example of an RD case solved through the analysis of CNVs undertaken here, from each of the four ERN partners in Solve-RD.

### Example of successful new diagnosis from ERN EURO-NMD

This male in his thirties first came to clinical attention in his adolescence, affected by poor balance, recurrent falls, and difficulty rising from the floor. Prior to this, he had been able to run and play sports normally. His symptoms worsened slowly over time, and he is currently unable to walk or stand without assistance. He also has mild facial weakness and mildly elevated serum creatine kinase. His family history is negative, having several unaffected siblings. Muscle biopsy showed clear features of muscular dystrophy, and immunohistochemical analysis suggested reduced expression of dystrophin. Exome sequencing was initially undertaken in 2017, but no diagnosis was reached at that point.

As a result of reanalysis of the ES data undertaken here, a three-exon deletion affecting exons 45 through 47 of the *DMD* gene (NC_000023.10:g.(?_31947661)_(32053731_?)[0]) was detected by both ExomeDepth and ClinCNV, consistent with the suspected diagnosis of Becker Muscular Dystrophy. This hemizygous deletion was subsequently confirmed using multiplex ligation-dependent probe amplification (MLPA). Confirmation of the molecular diagnosis in this individual has enabled enhanced genetic counselling, as any future daughter he may have would be an obligate, and possibly manifesting, carrier of the CNV, thus requiring clinical management.

### Example of successful new diagnosis from ERN GENTURIS

This family first came to clinical attention in 2003, meeting the criteria for hereditary diffuse gastric cancer (HDGC)^20^, as several family members had developed diffuse gastric cancers prior to 30 years of age. HDGC typically results from *CDH1* loss of function^21,22^. However, Sanger sequencing of *CDH1* performed proved negative, as did a subsequent investigation in the form of MLPA, and ES, at which point no potentially explanatory SNVs, InDels, or CNVs were identified in *CDH1*, nor other candidate genes^23^.

**Fig. 4:**
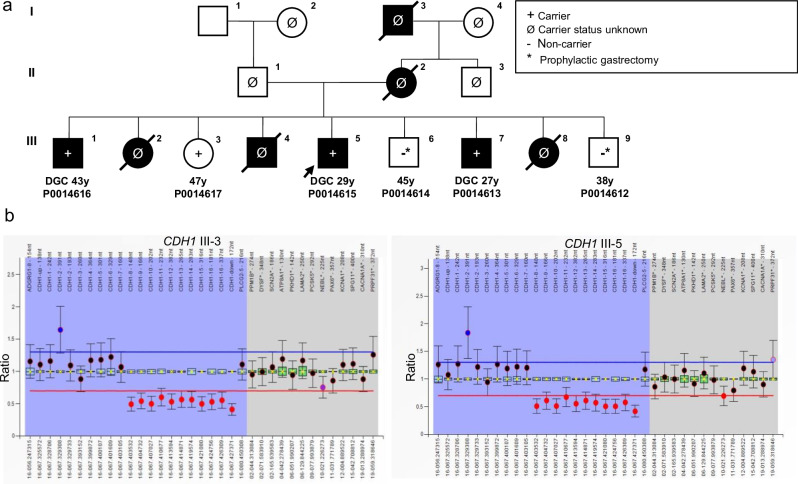
Family pedigree and MLPA confirmation results for a Mexican family extensively affected by Hereditary Gastric Cancer. **a** Family tree of the family of proband P0014615 (represented by an arrow). Exome Sequencing data from six individuals of the family was submitted to Solve-RD for re-analysis, following prior analysis in 2015 for both SNVs and CNVs, which did not identify any variants of interest. Three of the sequenced family members were affected by diffuse gastric cancer (DGC, black symbols: P0014616, P0014615, P0014613), while the other three were unaffected (P0014617, P0014614, P0014612). Individual III-3 (P0014617) is currently a healthy carrier, perhaps due to incomplete penetrance previously reported for *CHD1*. The age shown below affected individuals indicates the age of disease onset, while that below healthy individuals represents their current age. **b** MLPA validation results using SALSA MLPA-Probemix P083 *CDH1* (MRC Holland) in the healthy-carrier III-3, and in the proband, III-5. A ratio above the blue line indicates an elevated number of copies, while a ratio below the red line indicates a decrease in copy number. The shaded blue area represents the position of probes for *CDH1* and two neighbouring genes, while the grey area represents reference probes.

Following these negative findings, the ES data was submitted to Solve-RD for two affected, and four unaffected siblings. The comprehensive reanalysis of the ES data resulted in the identification of a ~116 kb heterozygous deletion impacting half of the *CDH1* gene (from intron 7 forwards) and the start of the downstream gene *TANGO6* (as far as intron 14) on chromosome 16 (NC_000016.9:g.(?_68846035)_(68961985_?)del) in four of the six siblings (Fig. 4). The CNV was detected by both ClinCNV and ExomeDepth and further supported by split-reads and abnormally paired reads observed in data from one of the affected individuals. Visualisation in IGV and subsequent MLPA validated this large event. Of note, one of the unaffected siblings, a female carrier in her 40s, has not developed gastric cancer to date, in accordance with previously reported incomplete penetrance among *CDH1* mutation carriers^24^. Another of the unaffected siblings was a carrier but never developed gastric cancer as a result of having undergone prophylactic total gastrectomy due to the high incidence of cancer in the family. The remaining unaffected siblings were found not to harbour the deletion, but unfortunately, both have also already undergone prophylactic gastrectomy. Nevertheless, as a result of their inclusion in Solve-RD, the family has since been recontacted and enroled in a clinical pathway of care, and their 20-year diagnostic odyssey has now come to an end. Importantly, targeted genetic testing has now been made available to their offspring to avoid unnecessary prophylactic gastrectomy in subsequent generations. The functional analysis and clinical implications of this CNV are described in more detail in São José et al.^25^.

### Example of successful new diagnosis from ERN ITHACA

This girl was first referred to paediatric neurology in her first year of life, presenting with generalised tonic-clonic seizures. During her infancy, mild global developmental delay became evident, with delays in speech and language acquirement and in gross-motor skill acquisition. Seizures were controlled with lamotrigine monotherapy, which could be discontinued during childhood following prolonged seizure-free periods. Apart from polyhydramnios, pregnancy and delivery were uncomplicated. Medical history comprised constipation and eczema, and family history was unremarkable. Physical examination revealed no additional phenotypic features, i.e. no congenital anomalies, no facial dysmorphisms, and no growth abnormalities. Investigations, including cerebral MRI and general metabolic screening were negative. Singleton ES was performed, followed by trio ES, which revealed a heterozygous de novo SNV of uncertain significance (VUS) in *STIP1* (*STIP1*; chr11(GRCh37):g.63961718C>T; NM_001282652.1:c.418C>T; p.(Arg140*)). Within this diagnostic trajectory, no analysis dedicated to CNV detection was performed.

The systematic reanalysis of ES data reported here led to the identification of a heterozygous 27 kb deletion on chromosome 6p21 (NC_000006.11:g.(?_31630124)_(31657924_?)del) in the proband. This deletion was detected by all three tools, and visual inspection of sequence alignment files in IGV clearly indicated the presence of the variant in the affected daughter, and its absence in both parents, thus confirming that it is a de novo deletion. The deletion fully removes *CSNK2B, LY6G5B* and *LY6G5C*, and its breakpoints affect *GPANK1* and *ABHD16A*. *GPANK1, LY6G5B* and *LY6G5C* currently have no disease association, and while *ABHD16A* is associated with autosomal recessive spastic paraplegia-86 (MIM#619735), there is no apparent second hit in *ABHD16A*, and the phenotype of the proband does not comprise spastic paraplegia. *CSNK2B*, on the other hand, has recently been shown to be associated with autosomal dominant Poirier-Bienvenu neurodevelopmental syndrome (POBINDS; MIM#618732), in which truncating variants in *CSNK2B* result in haploinsufficiency, leading to early-onset seizures and highly variable impairments of intellectual functioning^26–28^. As the de novo deletion observed in this proband results in haploinsufficiency of *CSNK2B*, and her phenotypic description fits within the *CSNK2B*-associated phenotypic spectrum, this 27 kb deletion on chromosome 6p21 is regarded as explanatory for her rare condition, thus ending a seven-year diagnostic odyssey for this family.

### Example of successful new diagnosis from ERN RND

This teenage female was first evaluated in paediatric neurology as a child, presenting with global developmental delay and behavioural and learning problems. Retrospectively, the first symptoms had become apparent in her infancy, consisting of mild delayed development of fine and gross motor skills. Additionally, she had delays in language and speech development and was diagnosed with attention deficit disorder, for which she is being treated with methylphenidate and responding well. No obvious dysmorphic features were observed upon physical examination, but mild hypertonia of the triceps surae, hyperreflexia, kinetic tremor, mirror hand movement, and a tiptoeing gait were observed. Subsequent cerebral MRI showed ventriculomegaly, corpus callosum hypoplasia, prominent cerebellar folia, and thin middle cerebellar peduncles. Genetic testing, consisting of aCGH (median resolution 180 kb), targeted testing for Fragile X syndrome, and ES did not pinpoint a molecular cause.

Systematic reanalysis of the ES data undertaken here led to the identification of a heterozygous deletion of ~200 kb at chromosome 4q31.1 (NC_000004.11:g.(?_140187697)_(140394334_?)del), encompassing part of the *MGARP* gene (not known to be associated with disease), and the entire *NAA15* gene, which encodes the catalytic subunit in the N-terminal acetyltransferase A complex (MIM: 608000). The deletion was identified by all three tools and subsequently validated using high-resolution aCGH (median resolution 60 kb). Following the review of the prior results, the absence of recall of the variant in the initial aCGH analysis was attributed to its limited resolution. The patient’s mother, who had had similar learning problems and has mild cognitive disability, was subsequently also found to be positive for the deletion. No further family testing was possible. Echocardiography was normal in both cases. Loss-of-function variants in *NAA15* and heterozygous deletion of this gene and nearby genes are associated with ‘Intellectual developmental disorder, autosomal dominant 50, with behavioural abnormalities’ (MIM: 617787)^20,29^. This disorder has the features of a wide spectrum of neurodevelopmental severity and variable association of congenital anomalies, thus confirming that the observed CNV was causative in this case, and ending this family’s seven-year diagnostic odyssey.

## Discussion

Rigorous detection of CNVs from ES requires sequencing data that has been generated as uniformly as possible, in order that the test experiment can be compared against a similarly generated batch of matched control samples. However, the ES data submitted to Solve-RD had been generated using 28 different enrichment kits and sequenced with different short-read technologies to different depths of coverage in multiple sequencing centres across Europe. Hence the primary challenge encountered during this analysis was data heterogeneity. Similarly, from the perspective of diagnosis, it is essential to have a clear clinical description of the affected individual to be able to determine which genes and variants, if encountered, may explain the observed phenotype. This was achieved here firstly through the use of the HPO to capture a deep phenotypic description of affected individuals from the referring clinicians, and secondly using the curated set of genes of interest provided by each ERN. Together these significantly reduced the search space for potentially disease-causing CNVs.

The interpretation of raw CNV calls is challenging due to the initial high number of calls most tools report. We applied a robust filtering strategy to remove calls that were clearly unlikely to be of relevance for RD and benefited from the curated lists of genes of interest provided by each ERN. Nevertheless, visual inspection of the affected region using IGV was key for assessing the technical validity of calls, prior to, or in parallel with, their biological interpretation. For interpretation purposes, we routinely provided the following images: (1) Image of normalised coverage across the whole genome, (2) Close-up images of apparent breakpoints, and (3) Image of the variant itself and the surrounding neighbourhood. It is likely that this is an aspect where an AI-based tool for automated IGV-image analysis would be of significant benefit, potentially saving many hours of human expert review time. We believe that a Machine Learning/AI tool could be trained to discriminate between whether a variant called by one of the algorithms is clearly a false positive or likely to be a bona fide biological event, in the same manner that the human eye can, when presented with the same images.

The clinical researchers representing each ERN applied their own prioritisation strategy when interpreting CNV calls according to the specific pathologic and phenotypic characteristics of their patients. When used as a first-tier analysis, CNV detection from ES has been reported to result in diagnostic yields as high as 7–19%^30–32^. The overall rate of novel diagnoses reached here through reanalysis was 0.9%, ranging from 0.6% for RND and 0.9% for ITHACA to 1.2% for GENTURIS and EURO-NMD. Notably, nine of the sixteen CNVs established as being disease-causing in ITHACA cases could be confirmed as de novo mutations due to ES data being available from the proband’s parents. While our values are lower than those of prior reports, where yield from reanalysis efforts, have resulted in increases in diagnostic yield with respect to CNVs in the range of 1.6–2.0%^24,33,34^ in those studies, the prior CNV analyses had largely consisted of only chromosomal microarray (CMA) analyses, which lack sensitivity for short CNV events, which were hence identified in the subsequent ES-based CNV analyses. Our results reflect several factors: the likelihood that detailed CNV analysis of the ES data had been undertaken prior to submission to Solve-RD; the role that CNVs are likely to play in the respective class of disease; the time passed since the initial analysis, which would affect the number of genes known to be associated with a particular class of disease. Interestingly, the number of genes of interest in each of the custom ERN gene lists does not appear to be a factor, given that GENTURIS had by far the shortest list, and RND and ITHACA the longest.

There was a clear bias towards deletions vis-à-vis duplications being identified as pathogenic, with 49 of 77 (64%) confirmed pathogenic CNVs being deletions and 42 of 52 (81%) disease-causing CNVs. This reflects the fact that duplications are more challenging to detect, and even when detected by ES, with DoC data alone it is invariably unclear as to whether they are tandem duplications, possibly inverted, or inserted elsewhere in the genome, each of these scenarios being likely to result in a different biological consequence, making interpretation challenging. Furthermore, long duplications appear to be under less evolutionary constraint than similarly sized deletions^35^, suggesting that they are less likely to result in disease. Accordingly, the ACMG guidelines for the interpretation of constitutional CNVs^36^, require more supporting evidence for a duplication to be confirmed as pathogenic than is required for a deletion.

It is noteworthy that, in comparison with the other two tools, Conifer called very few CNVs under 20 kb in length, and indeed failed to successfully identify 18 of 20 deletions <20 kb that were determined to be disease-causing, and the remaining two fell below the calling threshold. Notably, Conifer also failed to identify duplications over 1 Mb in length, including seven sex-chromosome aneuploidies, a limitation mentioned in the original paper^4^. It is this failure at the two extremes of CNV length that largely contributes to the inferior performance of Conifer. It should also be highlighted that we required a *Z*-score in excess of ±1.75 for a CNV called by Conifer to be returned for interpretation, whereas had we used ±1.5, Conifer would have successfully identified a further eight events of the disease-causing CNVs, all but two of which were over 20 kb in length. ClinCNV performed best of the three callers with this highly heterogeneous dataset, which is likely due to its more adaptive DoC calculation whereby it subsegments target regions into overlapping 120 bp tiles, significantly improving resolution, particularly for short CNVs, most of which were also detected by ExomeDepth but some of which fell below the minimal calling threshold. Indeed, only ClinCNV was sensitive enough to be able to identify the three events affecting only one or two exons in APC, MEIS2, and NFIB, respectively.

In addition to cases of de novo dominant inheritance resolved by an individual CNV, we also identified eight cases where an SNV and CNV were affecting different alleles of the same gene, potentially forming a disease-causing compound heterozygote. Two of these have been confirmed as being explanatory for the individuals’ conditions, with the remaining six requiring further validation. These findings underline the importance of having all data relevant to the interpretation of an affected individual’s condition readily at hand, as had the SNV and CNV analyses been undertaken independently, these individuals would have been unlikely to have received a diagnosis. Furthermore, in one affected individual, we identified two pathogenic CNVs affecting different genes, each of which explains unique features of the individual's complex phenotype, i.e. a dual diagnosis^37^. We are confident that many of the CNVs that we currently classify as candidates are likely pathogenic in the affected individuals, but complete follow-up has not yet been possible. The complete expert-curated dataset of deletions and duplications, together with the detailed phenotypes and pedigrees and the aligned sequence files (BAM/CRAMs), are available to the entire RD community via the European Genome–Phenome Archive (EGA)^38^, allowing for new discoveries (see Data Availability section, below).

There are many reasons why a pathogenic CNV identified here may not have been found in prior analysis of the ES data. Firstly, there may have been no attempt to identify CNVs by the respective clinical research team, due to a lack of resources or expertise. However, we know that some form of prior CNV analysis had been undertaken for the majority of affected individuals analysed here. Secondly, the tool(s) applied previously for CNV detection may not have identified the relevant CNV, or though identified, it may have been discarded due to local quality control parameters applied, e.g. ~10% of all the experiments submitted to Solve-RD were of sufficiently poor quality such that one of the centres involved in the reanalysis undertaken here would have routinely QC-failed the sample in their diagnostic workflow and thus not attempted to identify CNVs. Thirdly, while the CNV may have been identified, there may not have been any known association between the affected gene(s) and the clinical presentation of the patient at the time of the initial analysis, resulting in, at best, classification of the CNV as a variant of uncertain significance (VUS), and the individual remaining undiagnosed.

We would emphasise that any observations of potential tendencies in the results presented here must be interpreted prudently since this was an extremely heterogeneous dataset both in terms of the breadth and the quality of the data and in terms of the time and expertise that had been applied to the interpretation of the ES data in analyses undertaken prior to submission to Solve-RD. As we gather more information about the role of CNVs in RD through projects that share data widely, such as Solve-RD, hopefully, the accuracy of CNV detection will improve, and the entire process of identification and interpretation of this important class of variants, from sequencing data to identification of pathogenic variants can be automated, resulting in families affected by RD receiving a diagnosis sooner rather than later.

The work presented here has several clear limitations vis-a-vis reaching a diagnosis for individuals affected by an RD. Firstly, given that the data was from ES and that we only considered events affecting one of between 230 and 1944 genes of interest identified by each of the ERNs, we will obviously miss any non-exonic events or CNVs affecting genes not in the list of genes of interest. However, undertaking this work without using gene lists would result in a currently insurmountable load of data for interpretation, and novel gene discovery was not the goal of the work undertaken here. However, such discoveries are enabled by the sharing of data with the wider RD community via the EGA, which we hope will enable more cases to be solved. Different approaches in interpretation undertaken by the ERN experts may have resulted in some biologically relevant events being discarded as uninteresting, which may be particularly true for duplications, for which evidence of biological relevance in RD is currently relatively scarce. It is also possible that the application of other tools designed to find CNVs affecting only single exons, such as VarGenius-HZD^39^, may have allowed the identification of shorter events missed by the tools applied. With the future adoption of long-read genome sequencing technologies such as those provided by Oxford Nanopore Technologies and Pacific Biosciences, it is likely that the accuracy of CNV detection, and hence ease of interpretation, will improve markedly.

Despite these limitations, we have successfully provided diagnoses to at least 51 families who had previously undergone extensive genetic testing and, in many cases, multiple hospital visits over many years, some even decades, without having been provided with a diagnosis. Within the larger Solve-RD reanalysis of all variant types, these 51 CNVs were the second most common type of disease-causing variant identified, after SNVs/InDels, contributing to ~9% of the successful diagnoses (Laurie et al.^19^). The ending of a diagnostic odyssey has many benefits to patients and their families, beyond changes in medical management and genetic counselling of relatives. It also allows a better understanding of disease progression, access to disease-specific online communities, and psychological closure, amongst other benefits^40^. The work undertaken here indicates the value of comprehensive (re)-analysis of copy number variants in undiagnosed RD cases, even from historic ES data, and has resulted in patients and their families being given an accurate diagnosis, finally ending their diagnostic odysseys.

Based upon our findings, we suggest the following recommendations for future (re)-analyses of ES data with respect to the identification of disease-causing CNVs.

1. Know your enrichment kit. Investigate how well and how evenly it captures your genes of interest.
2. Choose your tools wisely. While Conifer has been shown to work with homogenous datasets, e.g., thousands of ES datasets generated using the same kit in the same sequencing centre, it does not perform with the heterogeneous dataset analysed here. Furthermore, it identified very few CNVs <20 kb in length, missing many disease-causing variants.
3. Identifying regions that are commonly copy-number variants. In this way any CNVs observed in such regions can be excluded from being potentially disease-causing.
4. Use an in silico candidate gene list when possible. This will greatly accelerate the process of interpretation. If the list is very short, then any signal of a CNV in a gene of interest should be examined further, since the sensitivity of tools remains low, and the prior probability of the gene being variant is high. However, as lists grow longer, this probability reduces, and calls will have to be filtered by quality thresholds.
5. Visualisation of CNV calls using a tool such as IGV is essential to assure that they are likely to be real biological events, prior to expending time and effort on further interpretation, investigation, and/or confirmation using orthologous techniques.

## Methods

### Data collation

The ES data reanalysed here comprises previously inconclusive ES experiments submitted for reanalysis as part of the Solve-RD project by 42 different research groups based in 12 countries across Europe and Canada (range of 1–2111 experiments submitted per group). Each experiment was submitted via one of four European Reference Networks (ERN) partnering in Solve-RD, each focusing on a particular group of RD: EURO-NMD (rare neuromuscular diseases); GENTURIS (rare genetic tumour risk syndromes); ITHACA (rare malformation syndromes, intellectual and other neurodevelopmental disorders); RND (rare neurological diseases).

A total of 9351 ES experiments from 9314 individuals (6224 affected individuals and 3090 unaffected relatives) were initially submitted for reanalysis. After the removal of samples sequenced with enrichment kits for which the available control cohort was <30 and thus not large enough to allow accurate CNV identification, data from 9171 individuals from 5757 families were analysed (see Technical Results). While 1320 of 1788 (74%) families from ITHACA were composed of parent–child trios, facilitating identification of de novo mutations, only 239 of the remaining 3969 (6%) probands from other ERNs were trios. ES had been performed using 28 different enrichment kits (range of 4–2078 experiments per kit), and each of the 42 research groups had followed their own DNA library preparation, target enrichment, and short-read sequencing protocol in their local labs, or via external DNA-sequencing providers. Furthermore, each group had previously undertaken its own historic analysis and interpretation of the resulting ES data to identify disease-causing variants, which had proven inconclusive. The date at which the initial ES analysis and interpretation had been undertaken ranged from 6 months to 8 years prior to the experimental data being submitted to Solve-RD for reanalysis; however, this information was not collected systematically for individual data sets.

In addition to sequencing data, a phenotypic description for each affected individual was recorded in the PhenoStore module of the RD-Connect GPAP^41^, consisting of a minimum of five Human Phenotype Ontology terms (HPO^42^) wherever possible, and disease classification using Orphanet Rare Disease Ontology (ORDO) ORPHA codes (http://www.orphadata.org/cgi-bin/index.php), and/or OMIM identifiers^43^ (https://www.omim.org/) where appropriate, together with family pedigrees. A detailed description of this data set can be found in Laurie et al.^19^.

### Ethics statement

The Ethics committee of the Eberhard Karl University of Tubingen gave ethical approval for this work. Written informed consent for data sharing within Europe for the purpose of research was obtained from all recruited individuals or their parents/legal guardians where appropriate. The responsibility of checking the data is suitable for submission to the RD-Connect GPAP and Solve-RD, including informed consent, lies within the data submitter as required by their Code of Conduct and Data Sharing Policy, respectively. In some cases, individuals had to be re-consented prior to data submission. This study adheres to the principles set out in the Declaration of Helsinki.

### Alignment and definition of capture regions of interest

Sequencing data was submitted in BAM, CRAM, or FastQ format. Where data was submitted in BAM or CRAM format, it was reconverted to FastQs at read-group level prior to being realigned to the hs37d5 human genome reference version, as used in phase 2 of the 1000 genomes project^44^ with BWA-MEM^45^ (v0.7.8-r455). As GC-rich enrichment targets are known to amplify poorly, resulting in unreliable CNV calling^46^, the GC-content for each target in each enrichment kit was calculated, and any targets in which the GC-content was >80% were removed from the corresponding target BED file prior to CNV calling. This resulted in the removal of <0.5% of target regions per kit. Ensembl version 75 was used for gene and transcript definition.

With the goal of maximising the probability of detecting potentially disease-causing CNVs, three different algorithms which identify CNVs based on DoC were applied. Two of these, Conifer^4^, and ExomeDepth^6^, have been widely applied to ES data with success previously, while the third, ClinCNV, was developed recently by a Solve-RD partner^47^. Each of these tools offers the practical advantage of separating the DoC calculation for each individual experiment from the CNV calling step, and thus CNVs were subsequently called in batches by enrichment kit. The processing took on average 1 CPU hour per experiment per tool, e.g. a batch of 500 samples was processed in around 32 h on a machine with 16 cores. Furthermore, each algorithm provides an estimate of the likelihood that calls produced are biologically real, and the most likely false positive calls were excluded based on these metrics. As primary filters, in the case of Conifer, a value in excess of ±1.75 SV-RPKM was required for a CNV call to be taken forward for biological interpretation, while for ExomeDepth a Bayes factor (BF) > 15 was required, and for ClinCNV, a minimum log likelihood estimation of twenty was applied.

### CNV call filtering and visualisation

As the focus of Solve-RD is diagnosing RD cases, through the identification of rare variants that are potentially disease-causing, any apparent CNV call observed in a region where more than 1% of individuals in the whole sample had a similar type of call (i.e. a deletion or duplication) were discarded as being too common to be clinically relevant with respect to RD. Furthermore, CNVs returned for interpretation by clinical experts were restricted to those that overlapped with at least part of a gene in a predefined list of curated genes of interest provided by the respective ERN: EURO-NMD (*n* = 615), GENTURIS (230), ITHACA (1944), RND (1820). The full list of ERN curated genes is provided in Supplementary Table 1 and details as to how these lists were determined in Laurie et al.^19^. Potential CNVs of interest were subsequently categorised into six non-redundant classes to aid interpretation: Long CNVs (>500 kb in length); Homozygous deletions; Heterozygous CNVs affecting genes known to cause disorders with an autosomal dominant mode of inheritance; Regions with apparent copy numbers of four or more; Gonosomal CNVs; Potential compound-heterozygous *double-hits* in the form of a CNV affecting the second allele of a gene in which biallelic variants are known to be disease-causing, and in which a potentially pathogenic SNV has been previously identified in Solve-RD. For each class recommendations were provided for interpretation, for example, computationally detected consanguinity status was used for prioritising short homozygous deletions (<500 bp) and short regions with copy number four or more, which would otherwise have been filtered due to the minimum size threshold. To provide support for the interpretation of the technical validity of CNV calls, images of regions containing CNV calls were generated automatically using the Integrative Genomics Viewer (IGV)^48^. A variety of custom tracks, including call tracks for each of the three algorithms, BAM DoC, and gene tracks for ERN genes of interest, were incorporated, among others.

### ClinCNV Workflow

Analysis was performed separately for experiments generated by different exome enrichment kits. Initially, ClinCNV calculates the average read coverage of targeted regions of the enrichment kit divided into 120 bp windows. As the first step of preprocessing, coverage is corrected for GC-content and library size for each sample individually. Following normalisation, systematically poorly covered regions (i.e. where 90% of samples had a normalised coverage < 0.3) were excluded, followed by the application of variance stabilisation of read counts (square root transformation). To ameliorate the potential impact of batch effects on coverage calculation, samples were further clustered based on their global coverage profiles. In generating these clusters, target regions in the top and bottom quintiles for a variance were excluded to minimise the potential impact of polymorphic regions on cluster generation and coverage profiles were smoothed using the rolling median. Uniform manifold approximation and projection (UMAP)^35^ was performed for the mapping of smoothed coverage profiles. Samples were clustered into subgroups with a minimum size of 15 using dbscan^49^. Finally, the coverage of each 120 bp window was normalised using the median of coverages within the cluster. Different potential copy numbers are modelled using the theoretically expected value and estimated variance, and the log likelihood of normalised coverage under different expected copy-number models is calculated for each window. Calling is performed analogously to Circular Binary Segmentation^50^ using a Maximum Subarray Sum algorithm^38^, i.e. the segment with the highest evidence supporting an alternative copy-number to that of the model is identified at each step of the segmentation, rather than the segment with the largest difference in mean.

Resulting CNV calls were filtered according to measures of within-kit allele frequency of the CNV and the noisiness of the coverage at the CNV site, requiring a minimum log likelihood ratio of 20 to be considered worthy of biological interpretation. A robust regression model is fitted, taking the 75% percentile rank of the per-chromosome number of CNVs as a response variable, and median read depth, enrichment kit, and predicted ancestry determined using SampleAncestry (https://github.com/imgag/ngs-bits/blob/master/doc/tools/SampleAncestry) as predictors. A sample was assessed as QC failed if the response variable was outwith the 99.5% prediction interval of the regression. The 75% percentile of the per-chromosome number of CNVs was chosen to overcome cases where long CNVs may have been segmented into many separate calls, and thus, an otherwise good sample could be falsely identified as QC failed if only the total number of CNV calls was used as a response. Where parents of a case were available (i.e. family trios), copy-number information from the parents was also provided to assist in interpretation and to confirm if CNVs represented de novo events.

### Conifer workflow

Conifer^4^ (http://conifer.sourceforge.net/) uses singular value decomposition (SVD) to identify rare CNVs from exome sequencing data. Samples with similar read lengths were analysed in the same batch, and sex-specific sample pools were created to generate accurate X-Chromosome calls. Reads Per Kilobase per Million mapped reads (RPKM) values were calculated independently by enrichment kit for all corresponding targets. Following SVD to identify biases in coverage introduced by batch effects, 3–15 components were removed from each group based on manual inspection of the inflection points of scree plots generated by the programme.

Within each analysis batch, if all experiments had <30 calls, the results were considered ready for further filtering. On the contrary, where any experiment in a batch had more than 30 calls, then if the median number of calls per experiment in the batch was less than 10, any experiment with more than 30 calls was discarded as failing QC, and the results from the remaining experiments were considered ready for filtering. However, if the median number of calls within the batch was more than 10 per experiment, then the SVD value was increased, and the batch analysis was rerun, until either all experiments had <30 calls or the median number of calls was <10, at which point any experiment with more than 30 calls was discarded as described above. CNVs with an SVD-ZRPKM value >1.75 or less than −1.75 were considered bona fide duplication or deletion calls, respectively, worthy of biological interpretation. Conifer does not provide any guidance as to the exact copy number identified at a particular locus and provides no further indicators of the quality of a detected event other than the SVD-RPKM metric.

### ExomeDepth workflow

ExomeDepth^6^ applies a beta-binomial model to the genome-wide distribution of read-depth data, aiming to compare a test sample to a similar reference set selected by the tool. For the implementation of the ExomeDepth workflow, the generation of read count data was separated from that of identifying candidate CNVs. Thus, for each experiment, read depth was initially calculated for all targets of the respective capture kit and stored as a Bioconductor iRanges object^44^^,45^^,51^. In the second step, all iRanges objects from experiments generated using the same enrichment kit were analysed as a batch to generate raw CNV call sets. In this second step, ExomeDepth automatically identifies an independent background reference set for each test sample by selecting the most closely correlated samples in terms of coverage from within the batch. Copy-number prediction is provided by the ratio of observed/expected reads over a set of targets. We interpreted these ratios in diploid chromosomes as follows:

- O/E ratio <0.10—likely homozygous deletion i.e. copy number (CN) = 0
- 0.10 < O/E ratio <0.75—likely heterozygous deletion; CN = 1
- 0.75 < O/E ratio <1.25—likely copy number neutral; CN = 2, i.e. No CNV to report
- 1.25 < O/E ratio <1.75—likely heterozygous duplication; CN = 3
- 1.75 < O/E ratio <2.25—CN = 4
- O/E ratio >2.25—CN OTHER

ExomeDepth provides two indicators of quality. The first is a sample-level indicator of the correlation between the test sample and the background reference, which should be >0.97 for the results to be regarded as reliable. Secondly, regarding call quality, ExomeDepth provides a Bayes factor (BF) based on the ratio of observed/expected reads over a set of apparently copy-number variant targets. Experiments with a correlation <0.97 were considered failing QC, and any calls with a BF < 0.15 were discarded as being unreliable.

### CNV classes

To aid downstream interpretation, each CNV call was categorised into one of six classes.

1. Putative CNVs longer than 500 kb in length were initially identified regardless of the presence or absence of genes of interest in the ERN gene lists. The recent release of large CNVs catalogues, such as DECIPHER, as well as the presence of a large number of case reports with chromosomal changes of this size and larger, allowed us to hypothesise that such variants could be interpreted successfully, even if the reported phenotypes of the patients exhibiting such variants may differ from the phenotypes expected for affected genes.
2. Homozygous deletions are generally rare, and the presence of a homozygous deletion needs to be interpreted very cautiously due to potentially incorrect enrichment kit reporting, or poor-quality library preparation. An important indicator that a putative homozygous deletion call is likely to be bona fide is the consanguinity status of the patient.
3. Heterozygous CNVs occurring in genes with a described autosomal-dominant mode of inheritance reported in OMIM.
4. Duplications with apparent copy number > 3. These may represent cases where alleles on both chromosomes are duplicated or cases where only the allele on one chromosome has been duplicated multiple times.
5. *Gonosomal CNVs*: As gonosomal CNVs require a mixed workflow depending on the sex of the participant, a separate set of calls was generated for CNV calls on chromosomes X and Y. In the case of the Y-Chromosome, only “Long” CNVs that would fall into category 1 above were reported for interpretation since there were no genes of interest on the Y-Chromosome on any of the ERN gene lists.
6. Potential compound heterozygote SNV/CNV “double-hits”. For a short list of experiments in which a single candidate SNV had been identified by the Solve-RD SNV working group, which was either listed in ClinVar as Pathogenic/Likely Pathogenic or predicted to have a high impact in a gene of interest, affecting an individual where the mode of inheritance was suspected to be recessive, (see Laurie et al.^19^) we investigated whether a potentially pathogenic CNV affecting the second allele of the same gene could explain the case as a compound heterozygote.

### Call filtering and visualisation

As the focus of Solve-RD is diagnosing RD cases, through the identification of rare variants that are potentially disease-causing, any apparent CNV call observed in a region where more than 1% of individuals in the whole sample had a similar type of call (*i.e*. a deletion or duplication) were discarded as being too common to be clinically relevant with respect to RD. Furthermore, CNVs returned for interpretation by clinical experts were restricted to those that overlapped with at least part of a gene in a predefined list of curated genes of interest provided by the respective ERN: EURO-NMD (*n* = 615), GENTURIS (230), ITHACA (1944), RND (1820). The full list of ERN curated genes is provided in Supplementary Table 1, and details as to how these lists were determined is described in Laurie et al.^19^. Potential CNVs of interest were subsequently categorised into six non-redundant classes to aid interpretation: Long CNVs (>500 kb in length); Homozygous deletions; Heterozygous CNVs affecting genes known to cause disorders with an autosomal dominant mode of inheritance; Regions with apparent copy numbers of four or more; Gonosomal CNVs; Potential compound-heterozygous *double-hits* in the form of a CNV affecting the second allele of a gene in which biallelic variants are known to be disease-causing, and in which a potentially pathogenic SNV has been previously identified in Solve-RD. For each class, we gave recommendations for interpretation; for example, computationally detected consanguinity status was used for prioritising small homozygous deletions (<500 bp) and small regions with copy number four or more, which would otherwise have been filtered due to the minimum size threshold.

**Fig. 5:**
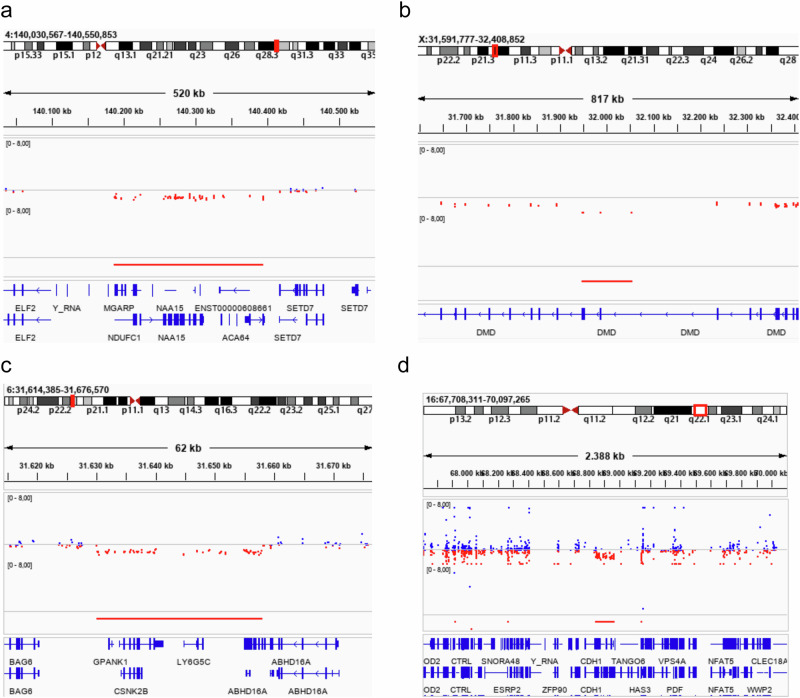
IGV screenshots correspond to the four illustrative newly diagnosed individuals described in the main text, one from each ERN. **a** RND: Heterozygous deletion spanning *NAA15*, in an individual with intellectual disability, which was found to be inherited from her paucisymptomatic mother. **b** EURO-NMD: Hemizygous deletion of exons 45-47 of *DMD* resulting in Becker Muscular Dystrophy. **c** ITHACA: Heterozygous de novo deletion spanning *CSNK2B*, resulting in POBINDS. **d** GENTURIS: Inherited heterozygous deletion affecting *CDH1* and *TANGO6*, resulting in autosomal dominant HDGC. Images show customised coverage tracks and the position of the identified CNV (red bar). Blue dots above the midline indicate elevated coverage, while red dots below the line indicate reduced coverage. The position of genes is indicated at the bottom of the image, while the chromosomal position is indicated at the top of the image.

To provide support for interpretation of the technical validity of CNV calls, screenshots for regions containing CNV calls were generated automatically using the Integrative Genomics Viewer^48^ (IGV), incorporating a variety of custom-built tracks (see Fig. 5). These included call tracks for each of the three callers in SEG format, normalised coverage tracks for ClinCNV and Conifer, beta-allele frequency, BAM DoC, Institute of Medical Genetics and Applied Genomics (Tübingen) in-house polymorphic CNV regions, and gene tracks from RefSeq genes, ERN candidate genes, and DECIPHER microdeletion and duplication syndromes^52^.

For each CNV returned for interpretation, we generated IGV screenshots of both the whole sample (chr1-22 and chrX/Y) to allow evaluation of overall sample quality, and the region around the individual CNV (±10 kb). Specifically in the case of long CNVs, the observation of clear deviations from the expected ratio of 50/50 in beta-allele frequencies provided strong additional support of variant validity. For rare cases in which a signal of unusual read pairing was observed, suggesting that a breakpoint may have been captured, a screenshot was generated, including the suspected breakpoint.

### Clinical interpretation

**Fig. 6:**
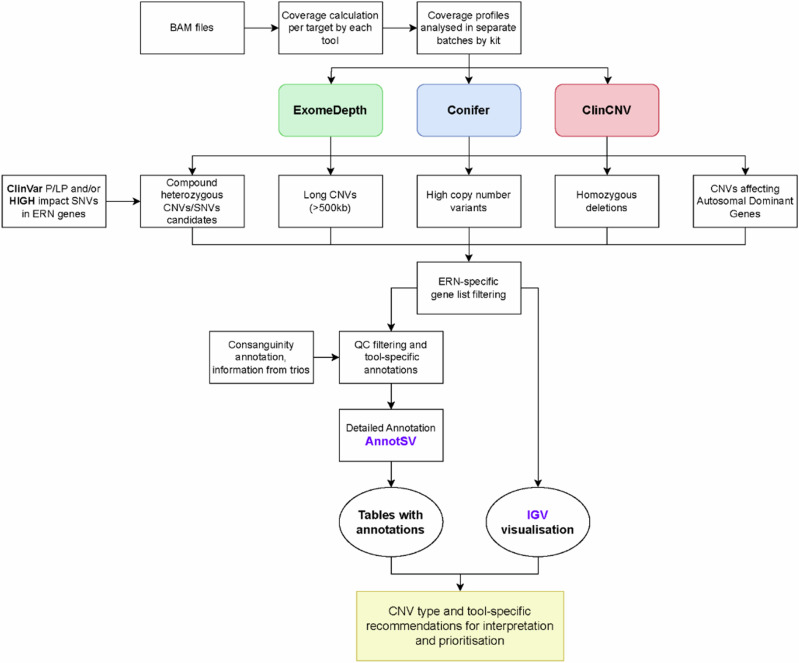
Workflow used for CNV calling, filtering, and annotation prior to returning calls to clinical experts for interpretation. The first line shows the pre-processing generation of coverage profiles for each experiment, prior to these profiles being passed to the 3 algorithms for CNV calling. The third line indicates the collation of CNVs of diferent types which were then annotatd and filtered appropriately before being passed to the respective ERNs for prioritisation.

Further annotations to aid interpretation (Supplementary Table 2) were added to the results using AnnotSV^53^ (Version 3.0.7), and fully annotated CNV call sets generated for all tools together with accompanying customised IGV visualisations were distributed to clinical experts in each ERN for diagnostic interpretation. Some annotations, such as that of the ENCODE blacklist for high-signal regions, were used to quickly discard overlapping CNVs by all ERNs, whereas other information, such as evidence of consanguinity, provided further support that homozygous deletions were likely to be relevant in affected cases. For the interpretation of heterozygous deletions, pLI scores from GnomAD^54^ and haploinsufficiency gene lists from the DDD project^55^, aided interpretation. Each ERN prioritised calls for further investigation based on their expert knowledge of underlying disease mechanisms in their respective patients. The full workflow is illustrated in Fig. 6. On average the clinical experts spent 5 min on interpretation per CNV with less than two CNVs of interest on average per sample. Many CNV calls could be rapidly discarded based upon a lack of match between the gene potentially affected and the phenotype of the affected individual, and/or segregation patterns within the family. Others were rejected when visual inspection of the IGV tracks indicated that they were likely false-positive calls, and thus unlikely to be bona fide biological events. Where deemed necessary and when feasible, CNVs believed to be diagnostically relevant were validated at local centres using orthologous approaches. The final decision as to whether a CNV was determined to be pathogenic or not was taken by the respective clinical experts from the ERN (see below for further details).

### The filtering strategy of ERN EURO-NMD

The filtering strategy undertaken by EURO-NMD was determined per analysis (see the section “Call filtering and visualisation” above). In general, a balance had to be upheld whereby clinical researchers would interpret as many CNVs as possible while maintaining a feasible interpretation load. Thus the following analyses were shared directly given the relative number of CNVs to be analysed: homozygous deletions, high copy number duplications, gonosomal CNVs, and potential compound heterozygote second hits, whereas heterozygous CNVs were split between CNVs of copy number one (CN1, i.e. deletions) and those of copy number three (CN3 i.e. duplications).

For CN1, CNVs for genes with DDD Haploinsuffiency scores > 90 or a GnomAD pLi < 0.1 were discarded, as these indicate that the gene is likely tolerant of heterozygous deletions. For both CN1 and CN3, CNVs identified through ClinCNV with a log likelihood <30 were discarded, as these are likely false positives. CNVs identified in genes only known to have recessive inheritance patterns were discarded, as were CNVs reported in Conrad et al.^56^. For long CNVs, CNVs found in the Encode blacklist were discarded. Following these filtering steps, experts from the submitting groups applied a phenotype-first approach. If the phenotype could potentially match with the gene affected by the CNV call, IGV tracks were checked to evaluate the likelihood of the called CNV being a true CNV.

### The filtering strategy of ERN GENTURIS

Due to the small size of the ERN GENTURIS cohort, and the short gene list, only limited further filtering of calls was necessary. No additional filters were applied to call sets from Conifer. In the case of heterozygote deletions and duplications, specific filtering criteria were applied separately for ClinCNV and ExomeDepth. For ClinCNV, we first interpreted all events identified by more than one tool, independent of the ClinCNV log likelihood value. After this, we proceeded to analyse all events called only by ClinCNV with a log likelihood of at least 20. For ExomeDepth, we first interpreted all events called by more than one tool, independently of the Bayes factor (BF), and subsequently considered events called only by ExomeDepth with a BF of at least 15. For long CNVs, we first discarded all those events found in the encode blacklist and analysed the rest. For all datasets, following IGV visualisation, only CNVs observed to be rare in control populations were considered for further interpretation.

### The filtering strategy of ERN ITHACA

For ERN ITHACA, as a first step, we discarded variants that were annotated to have low QC, had been previously annotated as benign, or occurred in regions on the Encode Blacklist, as provided by the AnnotSV annotation. Additionally, to reduce the proportion of false positives, we discarded deletions shorter than 10 kb and duplications shorter than 20 kb in length, with the exception of homozygous deletion calls and variants in parent-offspring trios identified as being de novo by ClinCNV. Following this, a visual inspection of each of the remaining CNV calls in IGV images was undertaken to assess technical validity, using reads and coverage supporting the call and B-allele frequency. Based on this visual assessment, apparently, real biological CNVs were defined. For detailed clinical interpretation, prioritisation was subsequently guided by genes present on the ERN ITHACA gene list with a disease-association validity score ≥3, see Laurie et al.^19^, consistent with the expected mode of inheritance. Of note, CNVs ≥200 kb were also investigated regardless of the presence or absence of a gene on the ERN ITHACA gene list, given the prior knowledge of large CNVs being involved in ITHACA-associated phenotypes. All CNVs passing the above criteria were returned to the submitting groups from ERN-ITHACA, for diagnostic interpretation based on the clinical relevance to the phenotype observed in the affected individual.

### The filtering strategy of ERN RND

The filtering strategy of ERN RND was predominantly based on tool-specific metrics. In general, the goal was to exclude calls with a high likelihood of being false positives. For ClinCNV, we discarded all calls with a log likelihood <30 and fist prioritised calls with a log likelihood > 200. As Conifer provides no metrics for filtering, all Conifer calls were analysed. For ExomeDepth, we discarded all calls affecting less than three targets and those with a Bayes factor <30, unless there was an overlapping CNV identified by one of the other tools. Following these filtering steps, the clinical researchers who submitted the case applied a phenotype-first approach. If the phenotype could potentially match that of the called CNV, IGV tracks were checked visually to evaluate the likelihood that the called CNV was bona fide.

## Supplementary information

Supplementary Information

## Data Availability

All raw and processed data files are deposited at the EGA (Datasets EGAD00001009767, EGAD00001009768, EGAD00001009769, and EGAD00001009770, under Solve-RD study EGAS00001003851) and can be made available upon approval by the Data Access Committee (EGAC00001001319). The family (FAM) and participant (P) identifiers used in this manuscript are pseudonymized and known only to the researchers involved In Solve-RD.

## Code availability

All the software tools used in this paper are open-source and freely available online at https://github.com/imgag/ClinCNV (ClinCNV 1.16.6), https://github.com/vplagnol/ExomeDepth (ExomeDepth 1.1.15), https://conifer.sourceforge.net/ (CoNIFER 0.2.2), https://github.com/lgmgeo/AnnotSV (AnnotSV v.3.0.7). Genome-Phenome Analysis Platform used for the metadata collection is available on https://platform.rd-connect.eu/.
