# Supplementary material for: Comprehensive reanalysis for CNVs in ES data from unsolved rare disease cases results in new diagnoses
Source: NPJ Genom Med. 2024 Oct 26;9:49. doi: 10.1038/s41525-024-00436-6 (PMC11513043; doi:10.1038/s41525-024-00436-6)
Supplement: Supplementary file 1 — Supplementary Information [file 41525_2024_436_MOESM1_ESM.pdf]

**Supplementary Table 1. Curated ERN gene lists.** The full expert-curated gene list from all four ERNs is provided. The gene symbol and identifier from Ensembl version 75 is used for consistency throughout this paper. We also provide the up-to-date HGNC gene symbol as of September, 2022 for reference and the given gene name. The gene list column indicates in which ERN list(s) each gene was included.
**Supplementary Table 2. Annotations added to CNV calls to aid variant interpretation.** The source column indicates the origin of the annotation, which are provided by either one of the three CNV calling tools, AnnotSV, or from the Solve-RD project in the case of phenotypic or family level data and candidate gene information.
**Supplementary Table 3. Number of families and affected individuals analysed per ERN, and number of families and affected individuals with at least one CNV requiring interpretation.** *Numbers reported for ERN ITHACA include 21 families (25 affected and 40 unaffected individuals) from the Spanish Undiagnosed Disease Program (UDP-Spain).
**Supplementary Table 4. Summary statistics regarding 7,849 CNVs initially returned for interpretation.**
**Supplementary Table 5. Summary statistics regarding the length of 3,487 duplications and 4,362 deletions returned for interpretation.**

Supplementary Table 1

Ensembl75_Gene_Symbol	Current_HGNC_Gene_Symbol	Gene_Name	ERN_Gene_List(s)
A2ML1	A2ML1	alpha-2-macroglobulin like 1	GENTURIS_ITHACA
AAAS	AAAS	aladin WD repeat nucleoporin	ITHACA_RND
AACS	AACS	acetoacetyl-CoA synthetase	ITHACA
AARS	AARS1	alanyl-tRNA synthetase 1	ITHACA_NMD_RND
AARS2	AARS2	alanyl-tRNA synthetase 2, mitochondrial	ITHACA_NMD_RND
AASS	AASS	aminoadipate-semialdehyde synthase	ITHACA_RND
ABAT	ABAT	4-aminobutyrate aminotransferase	ITHACA_RND
ABCA1	ABCA1	ATP binding cassette subfamily A member 1	RND
ABCA13	ABCA13	ATP binding cassette subfamily A member 13	ITHACA
ABCA2	ABCA2	ATP binding cassette subfamily A member 2	ITHACA
ABCA5	ABCA5	ATP binding cassette subfamily A member 5	ITHACA
ABCB11	ABCB11	ATP binding cassette subfamily B member 11	RND
ABCB4	ABCB4	ATP binding cassette subfamily B member 4	RND
ABCB7	ABCB7	ATP binding cassette subfamily B member 7	ITHACA_RND
ABCC8	ABCC8	ATP binding cassette subfamily C member 8	ITHACA
ABCC9	ABCC9	ATP binding cassette subfamily C member 9	ITHACA_NMD_RND
ABCD1	ABCD1	ATP binding cassette subfamily D member 1	ITHACA_RND
ABCD4	ABCD4	ATP binding cassette subfamily D member 4	ITHACA_RND
ABCG5	ABCG5	ATP binding cassette subfamily G member 5	RND
ABCG8	ABCG8	ATP binding cassette subfamily G member 8	RND
ABHD12	ABHD12	abhydrolase domain containing 12, lysophospholipase	RND
ABHD5	ABHD5	abhydrolase domain containing 5, lysophosphatidic acid acyltransferase	ITHACA_NMD_RND
ABHD6	ABHD6	abhydrolase domain containing 6, acylglycerol lipase	ITHACA
ABI2	ABI2	abl interactor 2	ITHACA
ABL1	ABL1	ABL proto-oncogene 1, non-receptor tyrosine kinase	ITHACA
ACACA	ACACA	acetyl-CoA carboxylase alpha	ITHACA
ACACB	ACACB	acetyl-CoA carboxylase beta	ITHACA
ACAD8	ACAD8	acyl-CoA dehydrogenase family member 8	RND
ACAD9	ACAD9	acyl-CoA dehydrogenase family member 9	ITHACA_NMD_RND
ACADM	ACADM	acyl-CoA dehydrogenase medium chain	ITHACA_RND
ACADS	ACADS	acyl-CoA dehydrogenase short chain	ITHACA_RND
ACADSB	ACADSB	acyl-CoA dehydrogenase short/branched chain	ITHACA_RND
ACADVL	ACADVL	acyl-CoA dehydrogenase very long chain	ITHACA_NMD_RND
ACAT1	ACAT1	acetyl-CoA acetyltransferase 1	ITHACA_RND
ACAT2	ACAT2	acetyl-CoA acetyltransferase 2	ITHACA
ACBD5	ACBD5	acyl-CoA binding domain containing 5	ITHACA

ACBD6	ACBD6	acyl-CoA binding domain containing 6	ITHACA
ACD	ACD	ACD shelterin complex subunit and telomerase recruitment factor	GENTURIS_ITHACA
ACER3	ACER3	alkaline ceramidase 3	ITHACA_NMD
ACHE	ACHE	acetylcholinesterase (Cartwright blood group)	ITHACA
ACO2	ACO2	aconitase 2	ITHACA_RND
ACOX1	ACOX1	acyl-CoA oxidase 1	ITHACA_RND
ACOX2	ACOX2	acyl-CoA oxidase 2	ITHACA
ACP5	ACP5	acid phosphatase 5, tartrate resistant	ITHACA_RND
ACSF3	ACSF3	acyl-CoA synthetase family member 3	ITHACA_RND
ACSL4	ACSL4	acyl-CoA synthetase long chain family member 4	ITHACA_RND
ACTA1	ACTA1	actin alpha 1, skeletal muscle	ITHACA_NMD_RND
ACTB	ACTB	actin beta	ITHACA_RND
ACTC1	ACTC1	actin alpha cardiac muscle 1	NMD
ACTG1	ACTG1	actin gamma 1	ITHACA_RND
ACTL6A	ACTL6A	actin like 6A	ITHACA_RND
ACTL6B	ACTL6B	actin like 6B	ITHACA_RND
ACTN2	ACTN2	actinin alpha 2	ITHACA_NMD_RND
ACVR1	ACVR1	activin A receptor type 1	ITHACA_NMD
ACY1	ACY1	aminoacylase 1	ITHACA_RND
ACY3	ACY3	aminoacylase 3	ITHACA
ADA	ADA	adenosine deaminase	ITHACA_RND
ADAM22	ADAM22	ADAM metallopeptidase domain 22	ITHACA
ADAM28	ADAM28	ADAM metallopeptidase domain 28	ITHACA
ADAM33	ADAM33	ADAM metallopeptidase domain 33	ITHACA
ADAMTS10	ADAMTS10	ADAM metallopeptidase with thrombospondin type 1 motif 10	ITHACA
ADAMTS2	ADAMTS2	ADAM metallopeptidase with thrombospondin type 1 motif 2	ITHACA
ADAMTS9	ADAMTS9	ADAM metallopeptidase with thrombospondin type 1 motif 9	ITHACA
ADAMTSL2	ADAMTSL2	ADAMTS like 2	ITHACA
ADAR	ADAR	adenosine deaminase RNA specific	ITHACA_RND
ADARB1	ADARB1	adenosine deaminase RNA specific B1	ITHACA
ADAT3	ADAT3	adenosine deaminase tRNA specific 3	ITHACA_RND
ADCK3	COQ8A	coenzyme Q8A	ITHACA_NMD_RND
ADCK4	COQ8B	coenzyme Q8B	RND
ADCY3	ADCY3	adenylate cyclase 3	ITHACA
ADCY5	ADCY5	adenylate cyclase 5	ITHACA_RND
ADCY6	ADCY6	adenylate cyclase 6	NMD
ADD3	ADD3	adducin 3	ITHACA
ADH5	ADH5	alcohol dehydrogenase 5 (class III), chi polypeptide	ITHACA
ADIPOR1	ADIPOR1	adiponectin receptor 1	ITHACA
ADK	ADK	adenosine kinase	ITHACA_RND
ADM	ADM	adrenomedullin	GENTURIS
ADNP	ADNP	activity dependent neuroprotector homeobox	ITHACA_RND
ADPRHL2	ADPRS	ADP-ribosylserine hydrolase	ITHACA_RND

ADRA2B	ADRA2B	adrenoceptor alpha 2B	ITHACA
ADSL	ADSL	adenylosuccinate lyase	ITHACA_RND
ADSSL1	ADSS1	adenylosuccinate synthase 1	NMD_RND
AFF2	AFF2	ALF transcription elongation factor 2	ITHACA_RND
AFF3	AFF3	ALF transcription elongation factor 3	ITHACA_RND
AFF4	AFF4	ALF transcription elongation factor 4	ITHACA_RND
AFG3L2	AFG3L2	AFG3 like matrix AAA peptidase subunit 2	ITHACA_NMD_RND
AGA	AGA	aspartylglucosaminidase	ITHACA_RND
AGAP1	AGAP1	ArfGAP with GTPase domain, ankyrin repeat and PH domain 1	ITHACA
AGAP2	AGAP2	ArfGAP with GTPase domain, ankyrin repeat and PH domain 2	ITHACA
AGBL2	AGBL2	AGBL carboxypeptidase 2	ITHACA
AGK	AGK	acylglycerol kinase	ITHACA_RND
AGL	AGL	amylo-alpha-1, 6-glucosidase, 4-alpha-glucanotransferase	NMD_RND
AGMO	AGMO	alkylglycerol monooxygenase	ITHACA
AGO1	AGO1	argonaute RISC component 1	ITHACA
AGO2	AGO2	argonaute RISC catalytic component 2	ITHACA
AGO3	AGO3	argonaute RISC catalytic component 3	ITHACA
AGO4	AGO4	argonaute RISC component 4	ITHACA
AGPAT2	AGPAT2	1-acylglycerol-3-phosphate O-acyltransferase 2	ITHACA
AGPS	AGPS	alkylglycerone phosphate synthase	ITHACA_RND
AGRN	AGRN	agrin	NMD_RND
AGTPBP1	AGTPBP1	ATP/GTP binding carboxypeptidase 1	ITHACA_NMD
AGTR2	AGTR2	angiotensin II receptor type 2	ITHACA
AGXT	AGXT	alanine--glyoxylate and serine--pyruvate aminotransferase	ITHACA_RND
AHCY	AHCY	adenosylhomocysteinase	ITHACA
AHDC1	AHDC1	AT-hook DNA binding motif containing 1	ITHACA_RND
AHI1	AHI1	Abelson helper integration site 1	ITHACA_RND
AHNAK	AHNAK	AHNAK nucleoprotein	ITHACA
AHNAK2	AHNAK2	AHNAK nucleoprotein 2	NMD
AHSG	AHSG	alpha 2-HS glycoprotein	ITHACA
AIFM1	AIFM1	apoptosis inducing factor mitochondria associated 1	ITHACA_NMD_RND
AIMP1	AIMP1	aminoacyl tRNA synthetase complex interacting multifunctional protein 1	ITHACA_RND
AIMP2	AIMP2	aminoacyl tRNA synthetase complex interacting multifunctional protein 2	ITHACA
AIP	AIP	aryl hydrocarbon receptor interacting protein	GENTURIS
AIPL1	AIPL1	aryl hydrocarbon receptor interacting protein like 1	ITHACA
AIRE	AIRE	autoimmune regulator	ITHACA
AK1	AK1	adenylate kinase 1	ITHACA
AK2	AK2	adenylate kinase 2	ITHACA
AKAP6	AKAP6	A-kinase anchoring protein 6	ITHACA
AKAP9	AKAP9	A-kinase anchoring protein 9	NMD
AKR1D1	AKR1D1	aldo-keto reductase family 1 member D1	ITHACA_RND

AKT1	AKT1	AKT serine/threonine kinase 1	GENTURIS_ITHACA_RND
AKT3	AKT3	AKT serine/threonine kinase 3	ITHACA_RND
ALAD	ALAD	aminolevulinate dehydratase	ITHACA_RND
ALAS2	ALAS2	5'-aminolevulinate synthase 2	RND
ALDH18A1	ALDH18A1	aldehyde dehydrogenase 18 family member A1	ITHACA_NMD_RND
ALDH1A3	ALDH1A3	aldehyde dehydrogenase 1 family member A3	ITHACA
ALDH3A2	ALDH3A2	aldehyde dehydrogenase 3 family member A2	ITHACA_NMD_RND
ALDH4A1	ALDH4A1	aldehyde dehydrogenase 4 family member A1	ITHACA_RND
ALDH5A1	ALDH5A1	aldehyde dehydrogenase 5 family member A1	ITHACA_RND
ALDH6A1	ALDH6A1	aldehyde dehydrogenase 6 family member A1	ITHACA_RND
ALDH7A1	ALDH7A1	aldehyde dehydrogenase 7 family member A1	ITHACA_RND
ALDOA	ALDOA	aldolase, fructose-bisphosphate A	ITHACA_RND
ALDOB	ALDOB	aldolase, fructose-bisphosphate B	ITHACA_RND
ALG1	ALG1	ALG1 chitobiosyldiphosphodolichol beta-mannosyltransferase	ITHACA_RND
ALG10	ALG10	ALG10 alpha-1,2-glucosyltransferase	ITHACA
ALG11	ALG11	ALG11 alpha-1,2-mannosyltransferase	ITHACA_RND
ALG12	ALG12	ALG12 alpha-1,6-mannosyltransferase	ITHACA_RND
ALG13	ALG13	ALG13 UDP-N-acetylglucosaminyltransferase subunit	ITHACA_NMD_RND
ALG14	ALG14	ALG14 UDP-N-acetylglucosaminyltransferase subunit	ITHACA_NMD_RND
ALG2	ALG2	ALG2 alpha-1,3/1,6-mannosyltransferase	ITHACA_NMD_RND
ALG3	ALG3	ALG3 alpha-1,3- mannosyltransferase	ITHACA_RND
ALG6	ALG6	ALG6 alpha-1,3-glucosyltransferase	ITHACA_RND
ALG8	ALG8	ALG8 alpha-1,3-glucosyltransferase	ITHACA_RND
ALG9	ALG9	ALG9 alpha-1,2-mannosyltransferase	ITHACA_RND
ALK	ALK	ALK receptor tyrosine kinase	GENTURIS
ALKBH8	ALKBH8	alkB homolog 8, tRNA methyltransferase	ITHACA_RND
ALMS1	ALMS1	ALMS1 centrosome and basal body associated protein	ITHACA_RND
ALOX12B	ALOX12B	arachidonate 12-lipoxygenase, 12R type	ITHACA
ALOXE3	ALOXE3	arachidonate lipoxygenase 3	ITHACA
ALPK1	ALPK1	alpha kinase 1	ITHACA
ALPK3	ALPK3	alpha kinase 3	NMD
ALPL	ALPL	alkaline phosphatase, biomineralization associated	ITHACA_RND
ALS2	ALS2	alsin Rho guanine nucleotide exchange factor ALS2	ITHACA_NMD_RND
ALX1	ALX1	ALX homeobox 1	ITHACA
ALX3	ALX3	ALX homeobox 3	ITHACA
ALX4	ALX4	ALX homeobox 4	ITHACA
AMACR	AMACR	alpha-methylacyl-CoA racemase	RND
AMBRA1	AMBRA1	autophagy and beclin 1 regulator 1	ITHACA
AMER1	AMER1	APC membrane recruitment protein 1	ITHACA_RND
AMER2	AMER2	APC membrane recruitment protein 2	ITHACA
AMHR2	AMHR2	anti-Mullerian hormone receptor type 2	ITHACA

AMMECR1	AMMECR1	AMMECR nuclear protein 1	ITHACA
AMN	AMN	amnion associated transmembrane protein	RND
AMPD2	AMPD2	adenosine monophosphate deaminase 2	ITHACA_NMD_RND
AMT	AMT	aminomethyltransferase	ITHACA_RND
AMZ2	AMZ2	archaelysin family metallopeptidase 2	ITHACA
ANAPC1	ANAPC1	anaphase promoting complex subunit 1	ITHACA
ANAPC2	ANAPC2	anaphase promoting complex subunit 2	ITHACA
ANG	ANG	angiogenin	NMD_RND
ANK2	ANK2	ankyrin 2	ITHACA_NMD
ANK3	ANK3	ankyrin 3	ITHACA
ANKH	ANKH	ANKH inorganic pyrophosphate transport regulator	ITHACA_RND
ANKHD1	ANKHD1	ankyrin repeat and KH domain containing 1	ITHACA
ANKLE2	ANKLE2	ankyrin repeat and LEM domain containing 2	ITHACA
ANKRD1	ANKRD1	ankyrin repeat domain 1	NMD
ANKRD11	ANKRD11	ankyrin repeat domain containing 11	ITHACA_RND
ANKRD26	ANKRD26	ankyrin repeat domain containing 26	GENTURIS
ANKS1B	ANKS1B	ankyrin repeat and sterile alpha motif domain containing 1B	ITHACA
ANO1	ANO1	anoctamin 1	ITHACA
ANO10	ANO10	anoctamin 10	ITHACA_NMD_RND
ANO3	ANO3	anoctamin 3	ITHACA_RND
ANO5	ANO5	anoctamin 5	ITHACA_NMD_RND
ANTXR1	ANTXR1	ANTXR cell adhesion molecule 1	ITHACA
ANXA11	ANXA11	annexin A11	NMD_RND
AP1B1	AP1B1	adaptor related protein complex 1 subunit beta 1	ITHACA
AP1S1	AP1S1	adaptor related protein complex 1 subunit sigma 1	ITHACA_RND
AP1S2	AP1S2	adaptor related protein complex 1 subunit sigma 2	ITHACA_RND
AP2M1	AP2M1	adaptor related protein complex 2 subunit mu 1	ITHACA_RND
AP2S1	AP2S1	adaptor related protein complex 2 subunit sigma 1	ITHACA
AP3B1	AP3B1	adaptor related protein complex 3 subunit beta 1	ITHACA_RND
AP3B2	AP3B2	adaptor related protein complex 3 subunit beta 2	ITHACA_RND
AP3D1	AP3D1	adaptor related protein complex 3 subunit delta 1	ITHACA
AP3S2	AP3S2	adaptor related protein complex 3 subunit sigma 2	ITHACA
AP4B1	AP4B1	adaptor related protein complex 4 subunit beta 1	ITHACA_NMD_RND
AP4E1	AP4E1	adaptor related protein complex 4 subunit epsilon 1	ITHACA_NMD_RND
AP4M1	AP4M1	adaptor related protein complex 4 subunit mu 1	ITHACA_NMD_RND
AP4S1	AP4S1	adaptor related protein complex 4 subunit sigma 1	ITHACA_NMD_RND
AP5Z1	AP5Z1	adaptor related protein complex 5 subunit zeta 1	ITHACA_NMD_RND

APBA2	APBA2	amyloid beta precursor protein binding family A member 2	ITHACA
APC	APC	APC regulator of WNT signaling pathway	GENTURIS
APC2	APC2	APC regulator of WNT signaling pathway 2	ITHACA_RND
APOA1	APOA1	apolipoprotein A1	RND
APOA1BP	NAXE	NAD(P)HX epimerase	ITHACA_RND
APOA5	APOA5	apolipoprotein A5	RND
APOB	APOB	apolipoprotein B	RND
APOC2	APOC2	apolipoprotein C2	RND
APOE	APOE	apolipoprotein E	RND
APOO	APOO	apolipoprotein O	ITHACA
APOPT1	COA8	cytochrome c oxidase assembly factor 8	ITHACA_RND
APP	APP	amyloid beta precursor protein	ITHACA_RND
APPL2	APPL2	adaptor protein, phosphotyrosine interacting with PH domain and leucine zipper 2	ITHACA
APRT	APRT	adenine phosphoribosyltransferase	RND
APTX	APTX	aprataxin	ITHACA_NMD_RND
AQP10	AQP10	aquaporin 10	ITHACA
AQP2	AQP2	aquaporin 2	ITHACA
AQP4	AQP4	aquaporin 4	ITHACA
AR	AR	androgen receptor	NMD_RND
ARCN1	ARCN1	archain 1	ITHACA_RND
AREL1	AREL1	apoptosis resistant E3 ubiquitin protein ligase 1	ITHACA
ARF1	ARF1	ADP ribosylation factor 1	ITHACA
ARFGEF2	ARFGEF2	ADP ribosylation factor guanine nucleotide exchange factor 2	ITHACA_RND
ARG1	ARG1	arginase 1	ITHACA_RND
ARHGAP15	ARHGAP15	Rho GTPase activating protein 15	ITHACA
ARHGAP21	ARHGAP21	Rho GTPase activating protein 21	ITHACA
ARHGAP31	ARHGAP31	Rho GTPase activating protein 31	ITHACA
ARHGAP32	ARHGAP32	Rho GTPase activating protein 32	ITHACA
ARHGAP33	ARHGAP33	Rho GTPase activating protein 33	ITHACA
ARHGAP35	ARHGAP35	Rho GTPase activating protein 35	ITHACA
ARHGDIA	ARHGDIA	Rho GDP dissociation inhibitor alpha	ITHACA
ARHGEF10	ARHGEF10	Rho guanine nucleotide exchange factor 10	NMD
ARHGEF2	ARHGEF2	Rho/Rac guanine nucleotide exchange factor 2	ITHACA
ARHGEF6	ARHGEF6	Rac/Cdc42 guanine nucleotide exchange factor 6	ITHACA
ARHGEF9	ARHGEF9	Cdc42 guanine nucleotide exchange factor 9	ITHACA_RND
ARID1A	ARID1A	AT-rich interaction domain 1A	ITHACA_RND
ARID1B	ARID1B	AT-rich interaction domain 1B	ITHACA_RND
ARID2	ARID2	AT-rich interaction domain 2	ITHACA_RND
ARIH1	ARIH1	ariadne RBR E3 ubiquitin protein ligase 1	ITHACA
ARL13B	ARL13B	ADP ribosylation factor like GTPase 13B	ITHACA_RND
ARL14EP	ARL14EP	ADP ribosylation factor like GTPase 14 effector protein	ITHACA
ARL3	ARL3	ADP ribosylation factor like GTPase 3	ITHACA
ARL6	ARL6	ADP ribosylation factor like GTPase 6	ITHACA_RND
ARL6IP1	ARL6IP1	ADP ribosylation factor like GTPase 6 interacting protein 1	ITHACA_NMD_RND
ARMC4	ODAD2	outer dynein arm docking complex subunit 2	ITHACA

ARMC5	ARMC5	armadillo repeat containing 5	GENTURIS
ARMC9	ARMC9	armadillo repeat containing 9	ITHACA_RND
ARNT2	ARNT2	aryl hydrocarbon receptor nuclear translocator 2	ITHACA
ARSA	ARSA	arylsulfatase A	ITHACA_RND
ARSB	ARSB	arylsulfatase B	ITHACA_RND
ARSE	ARSL	arylsulfatase L	ITHACA_RND
ARV1	ARV1	ARV1 homolog, fatty acid homeostasis modulator	ITHACA_RND
ARX	ARX	aristaless related homeobox	ITHACA_RND
ASAH1	ASAH1	N-acylsphingosine amidohydrolase 1	ITHACA_NMD_RND
ASCC1	ASCC1	activating signal cointegrator 1 complex subunit 1	ITHACA_NMD
ASCC3	ASCC3	activating signal cointegrator 1 complex subunit 3	ITHACA
ASCL1	ASCL1	achaete-scute family bHLH transcription factor 1	ITHACA
ASH1L	ASH1L	ASH1 like histone lysine methyltransferase	ITHACA_RND
ASH2L	ASH2L	ASH2 like, histone lysine methyltransferase complex subunit	ITHACA
ASL	ASL	argininosuccinate lyase	ITHACA_RND
ASNS	ASNS	asparagine synthetase (glutamine-hydrolyzing)	ITHACA_RND
ASPA	ASPA	aspartoacylase	ITHACA_RND
ASPM	ASPM	assembly factor for spindle microtubules	ITHACA_RND
ASS1	ASS1	argininosuccinate synthase 1	ITHACA_RND
ASTN1	ASTN1	astrotactin 1	ITHACA
ASTN2	ASTN2	astrotactin 2	ITHACA
ASUN	INTS13	integrator complex subunit 13	ITHACA
ASXL1	ASXL1	ASXL transcriptional regulator 1	GENTURIS_ITHACA_RND
ASXL2	ASXL2	ASXL transcriptional regulator 2	ITHACA_RND
ASXL3	ASXL3	ASXL transcriptional regulator 3	ITHACA_RND
ATAD1	ATAD1	ATPase family AAA domain containing 1	ITHACA_RND
ATAD3A	ATAD3A	ATPase family AAA domain containing 3A	ITHACA_RND
ATCAY	ATCAY	ATCAY kinesin light chain interacting caytaxin	ITHACA_RND
ATF7IP	ATF7IP	activating transcription factor 7 interacting protein	ITHACA
ATG5	ATG5	autophagy related 5	ITHACA_NMD
ATG7	ATG7	autophagy related 7	RND
ATIC	ATIC	5-aminoimidazole-4-carboxamide ribonucleotide formyltransferase/IMP cyclohydrolase	ITHACA_RND
ATL1	ATL1	atlastin GTPase 1	ITHACA_NMD_RND
ATL3	ATL3	atlastin GTPase 3	NMD
ATM	ATM	ATM serine/threonine kinase	GENTURIS_ITHACA_NMD_RND
ATN1	ATN1	atrophin 1	ITHACA_RND
ATOH1	ATOH1	atonal bHLH transcription factor 1	ITHACA
ATP10D	ATP10D	ATPase phospholipid transporting 10D (putative)	ITHACA
ATP13A1	ATP13A1	ATPase 13A1	ITHACA
ATP13A2	ATP13A2	ATPase cation transporting 13A2	ITHACA_NMD_RND
ATP1A1	ATP1A1	ATPase Na+/K+ transporting subunit alpha 1	ITHACA_NMD_RND

ATP1A2	ATP1A2	ATPase Na+/K+ transporting subunit alpha 2	ITHACA_NMD_RND
ATP1A3	ATP1A3	ATPase Na+/K+ transporting subunit alpha 3	ITHACA_RND
ATP1B1	ATP1B1	ATPase Na+/K+ transporting subunit beta 1	ITHACA
ATP2A1	ATP2A1	ATPase sarcoplasmic/endoplasmic reticulum Ca2+ transporting 1	ITHACA_NMD_RND
ATP2A2	ATP2A2	ATPase sarcoplasmic/endoplasmic reticulum Ca2+ transporting 2	ITHACA
ATP2B1	ATP2B1	ATPase plasma membrane Ca2+ transporting 1	ITHACA
ATP2B3	ATP2B3	ATPase plasma membrane Ca2+ transporting 3	ITHACA
ATP2C2	ATP2C2	ATPase secretory pathway Ca2+ transporting 2	ITHACA
ATP5A1	ATP5F1A	ATP synthase F1 subunit alpha	ITHACA
ATP5D	ATP5F1D	ATP synthase F1 subunit delta	ITHACA_RND
ATP5E	ATP5F1E	ATP synthase F1 subunit epsilon	ITHACA
ATP6AP1	ATP6AP1	ATPase H+ transporting accessory protein 1	ITHACA_RND
ATP6AP2	ATP6AP2	ATPase H+ transporting accessory protein 2	ITHACA_RND
ATP6V0A1	ATP6V0A1	ATPase H+ transporting V0 subunit a1	ITHACA
ATP6V0A2	ATP6V0A2	ATPase H+ transporting V0 subunit a2	ITHACA_RND
ATP6V0C	ATP6V0C	ATPase H+ transporting V0 subunit c	ITHACA
ATP6V1A	ATP6V1A	ATPase H+ transporting V1 subunit A	ITHACA_RND
ATP6V1B2	ATP6V1B2	ATPase H+ transporting V1 subunit B2	ITHACA_RND
ATP7A	ATP7A	ATPase copper transporting alpha	ITHACA_NMD_RND
ATP7B	ATP7B	ATPase copper transporting beta	RND
ATP8A2	ATP8A2	ATPase phospholipid transporting 8A2	ITHACA_RND
ATP8B1	ATP8B1	ATPase phospholipid transporting 8B1	RND
ATPAF2	ATPAF2	ATP synthase mitochondrial F1 complex assembly factor 2	ITHACA_RND
ATR	ATR	ATR serine/threonine kinase	GENTURIS_ITHACA_RND
ATRN	ATRN	attractin	ITHACA
ATRX	ATRX	ATRX chromatin remodeler	ITHACA_RND
ATXN1	ATXN1	ataxin 1	NMD
ATXN10	ATXN10	ataxin 10	NMD
ATXN2	ATXN2	ataxin 2	NMD
ATXN2L	ATXN2L	ataxin 2 like	ITHACA
ATXN3	ATXN3	ataxin 3	NMD
ATXN7	ATXN7	ataxin 7	NMD
ATXN8OS	ATXN8OS	ATXN8 opposite strand lncRNA	NMD
AUH	AUH	AU RNA binding methylglutaconyl-CoA hydratase	ITHACA_RND
AUTS2	AUTS2	activator of transcription and developmental regulator AUTS2	ITHACA_RND
AVPR2	AVPR2	arginine vasopressin receptor 2	ITHACA
AXIN1	AXIN1	axin 1	GENTURIS
AXIN2	AXIN2	axin 2	GENTURIS
AZI1	CEP131	centrosomal protein 131	ITHACA
B3GALNT2	B3GALNT2	beta-1,3-N-acetylgalactosaminyltransferase 2	ITHACA_NMD_RND
B3GALT6	B3GALT6	beta-1,3-galactosyltransferase 6	ITHACA_RND
B3GALTL	B3GLCT	beta 3-glucosyltransferase	ITHACA_RND
B3GAT3	B3GAT3	beta-1,3-glucuronyltransferase 3	ITHACA_RND

B3GNT1	B4GAT1	beta-1,4-glucuronyltransferase 1	ITHACA_NMD
B3GNT6	B3GNT6	UDP-GlcNAc:betaGal beta-1,3-N-acetylglucosaminyltransferase 6	RND
B4GALNT1	B4GALNT1	beta-1,4-N-acetyl-galactosaminyltransferase 1	ITHACA_NMD_RND
B4GALT1	B4GALT1	beta-1,4-galactosyltransferase 1	ITHACA_RND
B4GALT7	B4GALT7	beta-1,4-galactosyltransferase 7	ITHACA_RND
B9D1	B9D1	B9 domain containing 1	ITHACA
B9D2	B9D2	B9 domain containing 2	ITHACA_RND
BAAT	BAAT	bile acid-CoA:amino acid N-acyltransferase	RND
BAG3	BAG3	BAG cochaperone 3	NMD_RND
BAI3	ADGRB3	adhesion G protein-coupled receptor B3	ITHACA
BAP1	BAP1	BRCA1 associated protein 1	GENTURIS
BARD1	BARD1	BRCA1 associated RING domain 1	GENTURIS
BAZ1A	BAZ1A	bromodomain adjacent to zinc finger domain 1A	ITHACA
BAZ1B	BAZ1B	bromodomain adjacent to zinc finger domain 1B	ITHACA
BAZ2B	BAZ2B	bromodomain adjacent to zinc finger domain 2B	ITHACA
BBIP1	BBIP1	BBSome interacting protein 1	ITHACA
BBS1	BBS1	Bardet-Biedl syndrome 1	ITHACA_RND
BBS10	BBS10	Bardet-Biedl syndrome 10	ITHACA_RND
BBS12	BBS12	Bardet-Biedl syndrome 12	ITHACA_RND
BBS2	BBS2	Bardet-Biedl syndrome 2	ITHACA_RND
BBS4	BBS4	Bardet-Biedl syndrome 4	ITHACA_RND
BBS5	BBS5	Bardet-Biedl syndrome 5	ITHACA_RND
BBS7	BBS7	Bardet-Biedl syndrome 7	ITHACA_RND
BBS9	BBS9	Bardet-Biedl syndrome 9	ITHACA_RND
BCAP31	BCAP31	B cell receptor associated protein 31	ITHACA_RND
BCAS3	BCAS3	BCAS3 microtubule associated cell migration factor	ITHACA_RND
BCAT2	BCAT2	branched chain amino acid transaminase 2	ITHACA_RND
BCHE	BCHE	butyrylcholinesterase	ITHACA
BCKDHA	BCKDHA	branched chain keto acid dehydrogenase E1 subunit alpha	ITHACA_RND
BCKDHB	BCKDHB	branched chain keto acid dehydrogenase E1 subunit beta	ITHACA_RND
BCKDK	BCKDK	branched chain keto acid dehydrogenase kinase	ITHACA_RND
BCL11A	BCL11A	BAF chromatin remodeling complex subunit BCL11A	ITHACA_RND
BCL11B	BCL11B	BAF chromatin remodeling complex subunit BCL11B	ITHACA_RND
BCOR	BCOR	BCL6 corepressor	ITHACA_RND
BCORL1	BCORL1	BCL6 corepressor like 1	ITHACA
BCS1L	BCS1L	BCS1 homolog, ubiquinol-cytochrome c reductase complex chaperone	ITHACA_RND
BDH1	BDH1	3-hydroxybutyrate dehydrogenase 1	ITHACA
BDNF	BDNF	brain derived neurotrophic factor	ITHACA
BEAN1	BEAN1	brain expressed associated with NEDD4 1	NMD_RND
BICD2	BICD2	BICD cargo adaptor 2	ITHACA_NMD_RND
BIN1	BIN1	bridging integrator 1	ITHACA_NMD_RND

BLM	BLM	BLM RecQ like helicase	GENTURIS_ITHACA_RND
BMP1	BMP1	bone morphogenetic protein 1	ITHACA
BMP4	BMP4	bone morphogenetic protein 4	ITHACA_RND
BMPER	BMPER	BMP binding endothelial regulator	ITHACA
BMPR1A	BMPR1A	bone morphogenetic protein receptor type 1A	GENTURIS
BMPR1B	BMPR1B	bone morphogenetic protein receptor type 1B	ITHACA
BOD1	BOD1	biorientation of chromosomes in cell division 1	ITHACA
BOLA3	BOLA3	bolA family member 3	ITHACA_RND
BPIFB6	BPIFB6	BPI fold containing family B member 6	ITHACA
BPTF	BPTF	bromodomain PHD finger transcription factor	ITHACA_RND
BRAF	BRAF	B-Raf proto-oncogene, serine/threonine kinase	GENTURIS_ITHACA_RND
BRAT1	BRAT1	BRCA1 associated ATM activator 1	ITHACA_RND
BRCA1	BRCA1	BRCA1 DNA repair associated	GENTURIS
BRCA2	BRCA2	BRCA2 DNA repair associated	GENTURIS
BRD3	BRD3	bromodomain containing 3	ITHACA
BRD4	BRD4	bromodomain containing 4	ITHACA_RND
BRF1	BRF1	BRF1 RNA polymerase III transcription initiation factor subunit	ITHACA_RND
BRIP1	BRIP1	BRCA1 interacting helicase 1	GENTURIS_ITHACA
BRPF1	BRPF1	bromodomain and PHD finger containing 1	ITHACA_RND
BRSK2	BRSK2	BR serine/threonine kinase 2	ITHACA_RND
BRWD1	BRWD1	bromodomain and WD repeat domain containing 1	ITHACA
BRWD3	BRWD3	bromodomain and WD repeat domain containing 3	ITHACA_RND
BSCL2	BSCL2	BSCL2 lipid droplet biogenesis associated, seipin	ITHACA_NMD_RND
BSN	BSN	bassoon presynaptic cytomatrix protein	ITHACA
BSND	BSND	barttin CLCNK type accessory subunit beta	ITHACA
BTD	BTD	biotinidase	ITHACA_RND
BTF3	BTF3	basic transcription factor 3	ITHACA
BTK	BTK	Bruton tyrosine kinase	ITHACA
BTN1A1	BTN1A1	butyrophilin subfamily 1 member A1	ITHACA
BTN2A2	BTN2A2	butyrophilin subfamily 2 member A2	ITHACA
BTN3A2	BTN3A2	butyrophilin subfamily 3 member A2	ITHACA
BUB1	BUB1	BUB1 mitotic checkpoint serine/threonine kinase	GENTURIS
BUB1B	BUB1B	BUB1 mitotic checkpoint serine/threonine kinase B	GENTURIS_ITHACA_RND
BUB3	BUB3	BUB3 mitotic checkpoint protein	GENTURIS
BVES	BVES	blood vessel epicardial substance	NMD
BZRAP1	TSPOAP1	TSPO associated protein 1	ITHACA
C10orf118	CCDC186	coiled-coil domain containing 186	ITHACA
C10orf2	TWNK	twinkle mtDNA helicase	ITHACA_NMD_RND
C11orf73	HIKESHI	heat shock protein nuclear import factor hikeshi	ITHACA
C11orf83	UQCC3	ubiquinol-cytochrome c reductase complex assembly factor 3	ITHACA
C12orf4	C12orf4	chromosome 12 open reading frame 4	ITHACA_RND

C12orf57	C12orf57	chromosome 12 open reading frame 57	ITHACA_RND
C12orf65	MTRFR	mitochondrial translation release factor in rescue	ITHACA_NMD_RND
C14orf80	TEDC1	tubulin epsilon and delta complex 1	ITHACA
C15orf57	CCDC32	coiled-coil domain containing 32	ITHACA
C16orf62	VPS35L	VPS35 endosomal protein sorting factor like	ITHACA
C16orf90	C16orf90	chromosome 16 open reading frame 90	ITHACA
C17orf89	NDUFAF8	NADH:ubiquinone oxidoreductase complex assembly factor 8	ITHACA_RND
C19orf12	C19orf12	chromosome 19 open reading frame 12	ITHACA_NMD_RND
C19orf70	MICOS13	mitochondrial contact site and cristae organizing system subunit 13	ITHACA_RND
C1orf194	CFAP276	cilia and flagella associated protein 276	NMD
C1QBP	C1QBP	complement C1q binding protein	ITHACA_NMD_RND
C22orf31	C22orf31	chromosome 22 open reading frame 31	ITHACA
C2CD3	C2CD3	C2 domain containing 3 centriole elongation regulator	ITHACA_RND
C2orf42	C2orf42	chromosome 2 open reading frame 42	ITHACA
C3orf17	NEPRO	nucleolus and neural progenitor protein	ITHACA
C5orf42	CPLANE1	ciliogenesis and planar polarity effector complex subunit 1	ITHACA_RND
C7orf43	TRAPPC14	trafficking protein particle complex subunit 14	ITHACA
C8orf37	CFAP418	cilia and flagella associated protein 418	ITHACA
C9orf114	SPOUT1	SPOUT domain containing methyltransferase 1	ITHACA
C9orf41	CARNMT1	carnosine N-methyltransferase 1	ITHACA
C9orf72	C9orf72	C9orf72-SMCR8 complex subunit	ITHACA_NMD_RND
CA2	CA2	carbonic anhydrase 2	ITHACA_RND
CA5A	CA5A	carbonic anhydrase 5A	ITHACA_RND
CA8	CA8	carbonic anhydrase 8	ITHACA_RND
CABP7	CABP7	calcium binding protein 7	ITHACA
CACNA1A	CACNA1A	calcium voltage-gated channel subunit alpha1 A	ITHACA_NMD_RND
CACNA1B	CACNA1B	calcium voltage-gated channel subunit alpha1 B	ITHACA_RND
CACNA1C	CACNA1C	calcium voltage-gated channel subunit alpha1 C	ITHACA_NMD_RND
CACNA1D	CACNA1D	calcium voltage-gated channel subunit alpha1 D	ITHACA_RND
CACNA1E	CACNA1E	calcium voltage-gated channel subunit alpha1 E	ITHACA_RND
CACNA1G	CACNA1G	calcium voltage-gated channel subunit alpha1 G	ITHACA_NMD_RND
CACNA1H	CACNA1H	calcium voltage-gated channel subunit alpha1 H	ITHACA_NMD
CACNA1I	CACNA1I	calcium voltage-gated channel subunit alpha1 I	ITHACA
CACNA1S	CACNA1S	calcium voltage-gated channel subunit alpha1 S	NMD_RND
CACNA2D1	CACNA2D1	calcium voltage-gated channel auxiliary subunit alpha2delta 1	ITHACA
CACNA2D2	CACNA2D2	calcium voltage-gated channel auxiliary subunit alpha2delta 2	ITHACA_RND
CACNA2D3	CACNA2D3	calcium voltage-gated channel auxiliary subunit alpha2delta 3	ITHACA

CACNB2	CACNB2	calcium voltage-gated channel auxiliary subunit beta 2	ITHACA_NMD
CACNB4	CACNB4	calcium voltage-gated channel auxiliary subunit beta 4	ITHACA_NMD_RND
CACNG2	CACNG2	calcium voltage-gated channel auxiliary subunit gamma 2	ITHACA
CACNG7	CACNG7	calcium voltage-gated channel auxiliary subunit gamma 7	ITHACA
CAD	CAD	carbamoyl-phosphate synthetase 2, aspartate transcarbamylase, and dihydroorotase	ITHACA_RND
CADPS	CADPS	calcium dependent secretion activator	ITHACA
CADPS2	CADPS2	calcium dependent secretion activator 2	ITHACA
CALM1	CALM1	calmodulin 1	NMD
CALM2	CALM2	calmodulin 2	NMD
CALR3	CALR3	calreticulin 3	NMD
CAMK1D	CAMK1D	calcium/calmodulin dependent protein kinase ID	ITHACA
CAMK2A	CAMK2A	calcium/calmodulin dependent protein kinase II alpha	ITHACA_RND
CAMK2B	CAMK2B	calcium/calmodulin dependent protein kinase II beta	ITHACA_RND
CAMK2G	CAMK2G	calcium/calmodulin dependent protein kinase II gamma	ITHACA
CAMK4	CAMK4	calcium/calmodulin dependent protein kinase IV	ITHACA
CAMTA1	CAMTA1	calmodulin binding transcription activator 1	ITHACA_RND
CANT1	CANT1	calcium activated nucleotidase 1	ITHACA
CAPG	CAPG	capping actin protein, gelsolin like	ITHACA
CAPN1	CAPN1	calpain 1	NMD_RND
CAPN10	CAPN10	calpain 10	ITHACA
CAPN12	CAPN12	calpain 12	ITHACA
CAPN15	CAPN15	calpain 15	ITHACA
CAPN3	CAPN3	calpain 3	ITHACA_NMD_RND
CAPN9	CAPN9	calpain 9	ITHACA
CAPRIN1	CAPRIN1	cell cycle associated protein 1	ITHACA
CAPS	CAPS	calcyphosine	ITHACA
CAPS2	CAPS2	calcyphosine 2	ITHACA
CAPZA2	CAPZA2	capping actin protein of muscle Z-line subunit alpha 2	ITHACA
CARKD	NAXD	NAD(P)HX dehydratase	ITHACA
CARS	CARS1	cysteinyl-tRNA synthetase 1	ITHACA_RND
CARS2	CARS2	cysteinyl-tRNA synthetase 2, mitochondrial	ITHACA_RND
CASC5	KNL1	kinetochore scaffold 1	ITHACA_RND
CASK	CASK	calcium/calmodulin dependent serine protein kinase	ITHACA_RND
CASP2	CASP2	caspase 2	ITHACA
CASQ1	CASQ1	calsequestrin 1	NMD
CASQ2	CASQ2	calsequestrin 2	NMD
CASR	CASR	calcium sensing receptor	ITHACA
CASZ1	CASZ1	castor zinc finger 1	ITHACA
CAT	CAT	catalase	RND
CAV3	CAV3	caveolin 3	NMD_RND

CBL	CBL	Cbl proto-oncogene	GENTURIS_ITHACA_RND
CBS	CBS	cystathionine beta-synthase	ITHACA_RND
CBX4	CBX4	chromobox 4	ITHACA
CBY1	CBY1	chibby family member 1, beta catenin antagonist	ITHACA
CC2D1A	CC2D1A	coiled-coil and C2 domain containing 1A	ITHACA_RND
CC2D2A	CC2D2A	coiled-coil and C2 domain containing 2A	ITHACA_RND
CCAR2	CCAR2	cell cycle and apoptosis regulator 2	ITHACA
CCBE1	CCBE1	collagen and calcium binding EGF domains 1	ITHACA_RND
CCDC115	CCDC115	coiled-coil domain containing 115	ITHACA_RND
CCDC174	CCDC174	coiled-coil domain containing 174	ITHACA
CCDC22	CCDC22	coiled-coil domain containing 22	ITHACA_RND
CCDC23	SVBP	small vasohibin binding protein	ITHACA_RND
CCDC28B	CCDC28B	coiled-coil domain containing 28B	ITHACA
CCDC41	CEP83	centrosomal protein 83	ITHACA_RND
CCDC47	CCDC47	coiled-coil domain containing 47	ITHACA_RND
CCDC78	CCDC78	coiled-coil domain containing 78	ITHACA_NMD_RND
CCDC8	CCDC8	coiled-coil domain containing 8	ITHACA
CCDC82	CCDC82	coiled-coil domain containing 82	ITHACA
CCDC88A	CCDC88A	coiled-coil domain containing 88A	ITHACA
CCDC88C	CCDC88C	coiled-coil domain containing 88C	ITHACA_NMD_RND
CCNA2	CCNA2	cyclin A2	ITHACA
CCND2	CCND2	cyclin D2	ITHACA_RND
CCNF	CCNF	cyclin F	RND
CCNH	CCNH	cyclin H	ITHACA
CCNK	CCNK	cyclin K	ITHACA
CCNL2	CCNL2	cyclin L2	ITHACA
CCNT2	CCNT2	cyclin T2	ITHACA
CCT5	CCT5	chaperonin containing TCP1 subunit 5	NMD
CD163L1	CD163L1	CD163 molecule like 1	ITHACA
CD96	CD96	CD96 molecule	ITHACA
CDC42	CDC42	cell division cycle 42	ITHACA_RND
CDC42BPB	CDC42BPB	CDC42 binding protein kinase beta	ITHACA
CDC45	CDC45	cell division cycle 45	ITHACA
CDC6	CDC6	cell division cycle 6	ITHACA_RND
CDC73	CDC73	cell division cycle 73	GENTURIS
CDCA7	CDCA7	cell division cycle associated 7	ITHACA
CDH1	CDH1	cadherin 1	GENTURIS
CDH11	CDH11	cadherin 11	ITHACA_RND
CDH15	CDH15	cadherin 15	ITHACA_RND
CDH2	CDH2	cadherin 2	ITHACA_RND
CDH4	CDH4	cadherin 4	ITHACA
CDK10	CDK10	cyclin dependent kinase 10	ITHACA_RND
CDK13	CDK13	cyclin dependent kinase 13	ITHACA_RND
CDK16	CDK16	cyclin dependent kinase 16	ITHACA
CDK19	CDK19	cyclin dependent kinase 19	ITHACA
CDK4	CDK4	cyclin dependent kinase 4	GENTURIS
CDK5	CDK5	cyclin dependent kinase 5	ITHACA
CDK5R1	CDK5R1	cyclin dependent kinase 5 regulatory subunit 1	ITHACA

CDK5RAP2	CDK5RAP2	CDK5 regulatory subunit associated protein 2	ITHACA_RND
CDK6	CDK6	cyclin dependent kinase 6	ITHACA
CDK8	CDK8	cyclin dependent kinase 8	ITHACA_RND
CDK9	CDK9	cyclin dependent kinase 9	ITHACA
CDKL1	CDKL1	cyclin dependent kinase like 1	ITHACA
CDKL5	CDKL5	cyclin dependent kinase like 5	ITHACA_RND
CDKN1A	CDKN1A	cyclin dependent kinase inhibitor 1A	GENTURIS
CDKN1B	CDKN1B	cyclin dependent kinase inhibitor 1B	GENTURIS
CDKN1C	CDKN1C	cyclin dependent kinase inhibitor 1C	GENTURIS_ITHACA
CDKN2A	CDKN2A	cyclin dependent kinase inhibitor 2A	GENTURIS
CDKN2AIP	CDKN2AIP	CDKN2A interacting protein	ITHACA
CDKN2B	CDKN2B	cyclin dependent kinase inhibitor 2B	GENTURIS
CDKN2C	CDKN2C	cyclin dependent kinase inhibitor 2C	GENTURIS
CDON	CDON	cell adhesion associated, oncogene regulated	ITHACA_RND
CDT1	CDT1	chromatin licensing and DNA replication factor 1	ITHACA
CEBPA	CEBPA	CCAAT enhancer binding protein alpha	GENTURIS
CELF2	CELF2	CUGBP Elav-like family member 2	ITHACA
CELSR2	CELSR2	cadherin EGF LAG seven-pass G-type receptor 2	ITHACA
CENPE	CENPE	centromere protein E	ITHACA
CENPF	CENPF	centromere protein F	ITHACA_RND
CENPJ	CENPJ	centromere protein J	ITHACA_RND
CENPT	CENPT	centromere protein T	ITHACA
CEP104	CEP104	centrosomal protein 104	ITHACA_RND
CEP120	CEP120	centrosomal protein 120	ITHACA
CEP135	CEP135	centrosomal protein 135	ITHACA_RND
CEP152	CEP152	centrosomal protein 152	ITHACA_RND
CEP164	CEP164	centrosomal protein 164	ITHACA
CEP19	CEP19	centrosomal protein 19	ITHACA
CEP290	CEP290	centrosomal protein 290	ITHACA_RND
CEP41	CEP41	centrosomal protein 41	ITHACA_RND
CEP55	CEP55	centrosomal protein 55	ITHACA
CEP57	CEP57	centrosomal protein 57	ITHACA_RND
CEP63	CEP63	centrosomal protein 63	ITHACA
CEP76	CEP76	centrosomal protein 76	ITHACA
CEP85	CEP85	centrosomal protein 85	ITHACA
CEP85L	CEP85L	centrosomal protein 85 like	ITHACA
CEP89	CEP89	centrosomal protein 89	ITHACA
CEP97	CEP97	centrosomal protein 97	ITHACA
CERS1	CERS1	ceramide synthase 1	ITHACA
CFL2	CFL2	cofilin 2	ITHACA_NMD_RND
CHAF1B	CHAF1B	chromatin assembly factor 1 subunit B	ITHACA
CHAMP1	CHAMP1	chromosome alignment maintaining phosphoprotein 1	ITHACA_RND
CHAT	CHAT	choline O-acetyltransferase	NMD_RND
CHCHD10	CHCHD10	coiled-coil-helix-coiled-coil-helix domain containing 10	NMD_RND
CHCHD2	CHCHD2	coiled-coil-helix-coiled-coil-helix domain containing 2	RND
CHD1	CHD1	chromodomain helicase DNA binding protein 1	ITHACA

CHD1L	CHD1L	chromodomain helicase DNA binding protein 1 like	ITHACA
CHD2	CHD2	chromodomain helicase DNA binding protein 2	ITHACA_RND
CHD3	CHD3	chromodomain helicase DNA binding protein 3	ITHACA_RND
CHD4	CHD4	chromodomain helicase DNA binding protein 4	ITHACA_RND
CHD5	CHD5	chromodomain helicase DNA binding protein 5	ITHACA
CHD6	CHD6	chromodomain helicase DNA binding protein 6	ITHACA
CHD7	CHD7	chromodomain helicase DNA binding protein 7	ITHACA_RND
CHD8	CHD8	chromodomain helicase DNA binding protein 8	ITHACA_RND
CHD9	CHD9	chromodomain helicase DNA binding protein 9	ITHACA
CHEK1	CHEK1	checkpoint kinase 1	GENTURIS
CHEK2	CHEK2	checkpoint kinase 2	GENTURIS
CHKB	CHKB	choline kinase beta	ITHACA_NMD_RND
CHL1	CHL1	cell adhesion molecule L1 like	ITHACA
CHMP1A	CHMP1A	charged multivesicular body protein 1A	ITHACA_RND
CHMP2A	CHMP2A	charged multivesicular body protein 2A	ITHACA
CHMP2B	CHMP2B	charged multivesicular body protein 2B	NMD_RND
CHP1	CHP1	calcineurin like EF-hand protein 1	ITHACA_NMD
CHRM3	CHRM3	cholinergic receptor muscarinic 3	ITHACA
CHRNA1	CHRNA1	cholinergic receptor nicotinic alpha 1 subunit	ITHACA_NMD_RND
CHRNA2	CHRNA2	cholinergic receptor nicotinic alpha 2 subunit	RND
CHRNA4	CHRNA4	cholinergic receptor nicotinic alpha 4 subunit	ITHACA_RND
CHRNA7	CHRNA7	cholinergic receptor nicotinic alpha 7 subunit	ITHACA
CHRNB1	CHRNB1	cholinergic receptor nicotinic beta 1 subunit	NMD_RND
CHRNB2	CHRNB2	cholinergic receptor nicotinic beta 2 subunit	ITHACA_RND
CHRND	CHRND	cholinergic receptor nicotinic delta subunit	NMD_RND
CHRNE	CHRNE	cholinergic receptor nicotinic epsilon subunit	ITHACA_NMD_RND
CHRNG	CHRNG	cholinergic receptor nicotinic gamma subunit	NMD_RND
CHST14	CHST14	carbohydrate sulfotransferase 14	ITHACA_RND
CHST3	CHST3	carbohydrate sulfotransferase 3	ITHACA_RND
CHST5	CHST5	carbohydrate sulfotransferase 5	ITHACA
CHST6	CHST6	carbohydrate sulfotransferase 6	RND
CHSY1	CHSY1	chondroitin sulfate synthase 1	ITHACA_RND
CIB2	CIB2	calcium and integrin binding family member 2	ITHACA
CIC	CIC	capicua transcriptional repressor	ITHACA_RND
CINP	CINP	cyclin dependent kinase 2 interacting protein	ITHACA
CISD2	CISD2	CDGSH iron sulfur domain 2	RND
CIT	CIT	citron rho-interacting serine/threonine kinase	ITHACA_RND
CKAP2L	CKAP2L	cytoskeleton associated protein 2 like	ITHACA_RND
CKAP5	CKAP5	cytoskeleton associated protein 5	ITHACA
CLASP1	CLASP1	cytoplasmic linker associated protein 1	ITHACA
CLCN1	CLCN1	chloride voltage-gated channel 1	NMD
CLCN2	CLCN2	chloride voltage-gated channel 2	ITHACA_RND
CLCN4	CLCN4	chloride voltage-gated channel 4	ITHACA_RND
CLCN6	CLCN6	chloride voltage-gated channel 6	ITHACA
CLCN7	CLCN7	chloride voltage-gated channel 7	ITHACA
CLCNKA	CLCNKA	chloride voltage-gated channel Ka	ITHACA
CLCNKB	CLCNKB	chloride voltage-gated channel Kb	ITHACA

CLDN16	CLDN16	claudin 16	RND
CLDN19	CLDN19	claudin 19	RND
CLHC1	CLHC1	clathrin heavy chain linker domain containing 1	ITHACA
CLIC2	CLIC2	chloride intracellular channel 2	ITHACA
CLIP1	CLIP1	CAP-Gly domain containing linker protein 1	ITHACA
CLMN	CLMN	calmin	ITHACA
CLN3	CLN3	CLN3 lysosomal/endosomal transmembrane protein, battenin	ITHACA_NMD_RND
CLN5	CLN5	CLN5 intracellular trafficking protein	ITHACA_RND
CLN6	CLN6	CLN6 transmembrane ER protein	ITHACA_RND
CLN8	CLN8	CLN8 transmembrane ER and ERGIC protein	ITHACA_RND
CLP1	CLP1	cleavage factor polyribonucleotide kinase subunit 1	ITHACA_RND
CLPB	CLPB	caseinolytic mitochondrial matrix peptidase chaperone subunit B	ITHACA_RND
CLPP	CLPP	caseinolytic mitochondrial matrix peptidase proteolytic subunit	ITHACA_RND
CLPTM1	CLPTM1	CLPTM1 regulator of GABA type A receptor forward trafficking	ITHACA
CLSTN2	CLSTN2	calsyntenin 2	ITHACA
CLTC	CLTC	clathrin heavy chain	ITHACA_RND
CLTCL1	CLTCL1	clathrin heavy chain like 1	ITHACA_NMD
CMAS	CMAS	cytidine monophosphate N-acetylneuraminic acid synthetase	ITHACA
CNBP	CNBP	CCHC-type zinc finger nucleic acid binding protein	NMD_RND
CNKSR1	CNKSR1	connector enhancer of kinase suppressor of Ras 1	ITHACA
CNKSR2	CNKSR2	connector enhancer of kinase suppressor of Ras 2	ITHACA_RND
CNNM2	CNNM2	cyclin and CBS domain divalent metal cation transport mediator 2	ITHACA_RND
CNOT1	CNOT1	CCR4-NOT transcription complex subunit 1	ITHACA_RND
CNOT2	CNOT2	CCR4-NOT transcription complex subunit 2	ITHACA_RND
CNOT3	CNOT3	CCR4-NOT transcription complex subunit 3	ITHACA_RND
CNOT4	CNOT4	CCR4-NOT transcription complex subunit 4	ITHACA
CNPY3	CNPY3	canopy FGF signaling regulator 3	ITHACA_RND
CNTN1	CNTN1	contactin 1	NMD
CNTN3	CNTN3	contactin 3	ITHACA
CNTN4	CNTN4	contactin 4	ITHACA
CNTN5	CNTN5	contactin 5	ITHACA
CNTNAP1	CNTNAP1	contactin associated protein 1	ITHACA_NMD_RND
CNTNAP2	CNTNAP2	contactin associated protein 2	ITHACA_RND
CNTNAP4	CNTNAP4	contactin associated protein family member 4	ITHACA
COA3	COA3	cytochrome c oxidase assembly factor 3	ITHACA
COA6	COA6	cytochrome c oxidase assembly factor 6	ITHACA_RND
COA7	COA7	cytochrome c oxidase assembly factor 7	ITHACA_NMD_RND
COASY	COASY	Coenzyme A synthase	ITHACA_RND
COG1	COG1	component of oligomeric golgi complex 1	ITHACA_RND
COG2	COG2	component of oligomeric golgi complex 2	ITHACA
COG4	COG4	component of oligomeric golgi complex 4	ITHACA_RND
COG5	COG5	component of oligomeric golgi complex 5	ITHACA_RND

COG6	COG6	component of oligomeric golgi complex 6	ITHACA_RND
COG7	COG7	component of oligomeric golgi complex 7	ITHACA_RND
COG8	COG8	component of oligomeric golgi complex 8	ITHACA_RND
COL12A1	COL12A1	collagen type XII alpha 1 chain	ITHACA_NMD_RND
COL13A1	COL13A1	collagen type XIII alpha 1 chain	ITHACA_NMD_RND
COL18A1	COL18A1	collagen type XVIII alpha 1 chain	ITHACA_RND
COL1A1	COL1A1	collagen type I alpha 1 chain	ITHACA
COL1A2	COL1A2	collagen type I alpha 2 chain	ITHACA
COL25A1	COL25A1	collagen type XXV alpha 1 chain	NMD
COL27A1	COL27A1	collagen type XXVII alpha 1 chain	ITHACA
COL2A1	COL2A1	collagen type II alpha 1 chain	ITHACA
COL3A1	COL3A1	collagen type III alpha 1 chain	ITHACA
COL4A1	COL4A1	collagen type IV alpha 1 chain	ITHACA_RND
COL4A2	COL4A2	collagen type IV alpha 2 chain	ITHACA_RND
COL4A3BP	CERT1	ceramide transporter 1	ITHACA_RND
COL5A1	COL5A1	collagen type V alpha 1 chain	NMD
COL6A1	COL6A1	collagen type VI alpha 1 chain	ITHACA_NMD_RND
COL6A2	COL6A2	collagen type VI alpha 2 chain	ITHACA_NMD_RND
COL6A3	COL6A3	collagen type VI alpha 3 chain	ITHACA_NMD_RND
COL9A3	COL9A3	collagen type IX alpha 3 chain	ITHACA
COLEC10	COLEC10	collectin subfamily member 10	ITHACA
COLEC11	COLEC11	collectin subfamily member 11	ITHACA_RND
COLGALT1	COLGALT1	collagen beta(1-O)galactosyltransferase 1	ITHACA
COLQ	COLQ	collagen like tail subunit of asymmetric acetylcholinesterase	NMD_RND
COPB1	COPB1	COPI coat complex subunit beta 1	ITHACA
COPB2	COPB2	COPI coat complex subunit beta 2	ITHACA
COQ2	COQ2	coenzyme Q2, polyprenyltransferase	ITHACA_NMD_RND
COQ4	COQ4	coenzyme Q4	ITHACA_NMD_RND
COQ5	COQ5	coenzyme Q5, methyltransferase	ITHACA
COQ6	COQ6	coenzyme Q6, monooxygenase	NMD_RND
COQ7	COQ7	coenzyme Q7, hydroxylase	ITHACA_NMD_RND
COQ9	COQ9	coenzyme Q9	ITHACA_NMD_RND
CORO1A	CORO1A	coronin 1A	ITHACA
COX10	COX10	cytochrome c oxidase assembly factor heme A:farnesyltransferase COX10	ITHACA_RND
COX14	COX14	cytochrome c oxidase assembly factor COX14	ITHACA_RND
COX15	COX15	cytochrome c oxidase assembly homolog COX15	ITHACA_NMD_RND
COX16	COX16	cytochrome c oxidase assembly factor COX16	ITHACA
COX20	COX20	cytochrome c oxidase assembly factor COX20	ITHACA_RND
COX4I1	COX4I1	cytochrome c oxidase subunit 4I1	ITHACA

COX4I2	COX4I2	cytochrome c oxidase subunit 4I2	ITHACA
COX5A	COX5A	cytochrome c oxidase subunit 5A	ITHACA
COX6A1	COX6A1	cytochrome c oxidase subunit 6A1	NMD_RND
COX6A2	COX6A2	cytochrome c oxidase subunit 6A2	NMD
COX6B1	COX6B1	cytochrome c oxidase subunit 6B1	ITHACA_RND
COX7B	COX7B	cytochrome c oxidase subunit 7B	ITHACA_RND
COX8A	COX8A	cytochrome c oxidase subunit 8A	ITHACA
CP	CP	ceruloplasmin	RND
CPA6	CPA6	carboxypeptidase A6	RND
CPE	CPE	carboxypeptidase E	ITHACA
CPLX1	CPLX1	complexin 1	ITHACA
CPNE6	CPNE6	copine 6	ITHACA
CPOX	CPOX	coproporphyrinogen oxidase	RND
CPS1	CPS1	carbamoyl-phosphate synthase 1	ITHACA_RND
CPT1A	CPT1A	carnitine palmitoyltransferase 1A	ITHACA_RND
CPT1C	CPT1C	carnitine palmitoyltransferase 1C	NMD_RND
CPT2	CPT2	carnitine palmitoyltransferase 2	NMD_RND
CRADD	CRADD	CASP2 and RIPK1 domain containing adaptor with death domain	ITHACA_RND
CRAT	CRAT	carnitine O-acetyltransferase	ITHACA
CRB2	CRB2	crumbs cell polarity complex component 2	ITHACA_RND
CRBN	CRBN	cereblon	ITHACA
CREBBP	CREBBP	CREB binding protein	GENTURIS_ITHACA_RND
CRIPT	CRIPT	CXXC repeat containing interactor of PDZ3 domain	ITHACA
CRLF1	CRLF1	cytokine receptor like factor 1	ITHACA
CRYAB	CRYAB	crystallin alpha B	NMD_RND
CRYL1	CRYL1	crystallin lambda 1	ITHACA
CSDE1	CSDE1	cold shock domain containing E1	ITHACA_RND
CSF1R	CSF1R	colony stimulating factor 1 receptor	ITHACA_RND
CSGALNACT1	CSGALNACT1	chondroitin sulfate N-acetylgalactosaminyltransferase 1	ITHACA
CSNK1D	CSNK1D	casein kinase 1 delta	RND
CSNK1E	CSNK1E	casein kinase 1 epsilon	ITHACA
CSNK1G1	CSNK1G1	casein kinase 1 gamma 1	ITHACA
CSNK2A1	CSNK2A1	casein kinase 2 alpha 1	ITHACA_RND
CSNK2B	CSNK2B	casein kinase 2 beta	ITHACA_RND
CSPP1	CSPP1	centrosome and spindle pole associated protein 1	ITHACA_RND
CSRP3	CSRP3	cysteine and glycine rich protein 3	NMD
CSTB	CSTB	cystatin B	ITHACA_RND
CSTF2	CSTF2	cleavage stimulation factor subunit 2	ITHACA
CSTF2T	CSTF2T	cleavage stimulation factor subunit 2 tau variant	ITHACA
CTBP1	CTBP1	C-terminal binding protein 1	ITHACA_RND
CTC1	CTC1	CST telomere replication complex component 1	GENTURIS_ITHACA_RND
CTCF	CTCF	CCCTC-binding factor	ITHACA_RND
CTDP1	CTDP1	CTD phosphatase subunit 1	ITHACA_NMD_RND
CTH	CTH	cystathionine gamma-lyase	RND
CTNNA1	CTNNA1	catenin alpha 1	GENTURIS

CTNNA2	CTNNA2	catenin alpha 2	ITHACA_RND
CTNNA3	CTNNA3	catenin alpha 3	NMD
CTNNB1	CTNNB1	catenin beta 1	ITHACA_RND
CTNND1	CTNND1	catenin delta 1	ITHACA
CTNND2	CTNND2	catenin delta 2	ITHACA
CTNS	CTNS	cystinosin, lysosomal cystine transporter	RND
CTR9	CTR9	CTR9 homolog, Paf1/RNA polymerase II complex component	GENTURIS
CTSA	CTSA	cathepsin A	ITHACA_RND
CTSC	CTSC	cathepsin C	RND
CTSD	CTSD	cathepsin D	ITHACA_RND
CTSF	CTSF	cathepsin F	RND
CTSK	CTSK	cathepsin K	RND
CTTNBP2	CTTNBP2	cortactin binding protein 2	ITHACA
CTU2	CTU2	cytosolic thiouridylase subunit 2	ITHACA
CUBN	CUBN	cubilin	RND
CUL3	CUL3	cullin 3	ITHACA
CUL4B	CUL4B	cullin 4B	ITHACA_RND
CUL7	CUL7	cullin 7	ITHACA
CUX1	CUX1	cut like homeobox 1	ITHACA_RND
CUX2	CUX2	cut like homeobox 2	ITHACA_RND
CWC27	CWC27	CWC27 spliceosome associated cyclophilin	ITHACA_RND
CWF19L1	CWF19L1	CWF19 like cell cycle control factor 1	ITHACA_NMD_RND
CX3CR1	CX3CR1	C-X3-C motif chemokine receptor 1	ITHACA
CXorf56	STEEP1	STING1 ER exit protein 1	ITHACA
CYB5R3	CYB5R3	cytochrome b5 reductase 3	ITHACA_RND
CYC1	CYC1	cytochrome c1	ITHACA_RND
CYFIP1	CYFIP1	cytoplasmic FMR1 interacting protein 1	ITHACA
CYFIP2	CYFIP2	cytoplasmic FMR1 interacting protein 2	ITHACA_RND
CYLD	CYLD	CYLD lysine 63 deubiquitinase	GENTURIS
CYP24A1	CYP24A1	cytochrome P450 family 24 subfamily A member 1	ITHACA
CYP27A1	CYP27A1	cytochrome P450 family 27 subfamily A member 1	ITHACA_RND
CYP27B1	CYP27B1	cytochrome P450 family 27 subfamily B member 1	ITHACA
CYP2U1	CYP2U1	cytochrome P450 family 2 subfamily U member 1	ITHACA_NMD_RND
CYP3A4	CYP3A4	cytochrome P450 family 3 subfamily A member 4	ITHACA
CYP7B1	CYP7B1	cytochrome P450 family 7 subfamily B member 1	ITHACA_NMD_RND
D2HGDH	D2HGDH	D-2-hydroxyglutarate dehydrogenase	ITHACA_RND
DAB1	DAB1	DAB adaptor protein 1	RND
DAG1	DAG1	dystroglycan 1	ITHACA_NMD_RND
DAK	TKFC	triokinase and FMN cyclase	ITHACA
DALRD3	DALRD3	DALR anticodon binding domain containing 3	ITHACA
DARS	DARS1	aspartyl-tRNA synthetase 1	ITHACA_RND
DARS2	DARS2	aspartyl-tRNA synthetase 2, mitochondrial	ITHACA_RND
DBH	DBH	dopamine beta-hydroxylase	RND

DBT	DBT	dihydrolipoamide branched chain transacylase E2	ITHACA_RND
DCAF15	DCAF15	DDB1 and CUL4 associated factor 15	ITHACA
DCAF17	DCAF17	DDB1 and CUL4 associated factor 17	ITHACA_RND
DCAF8	DCAF8	DDB1 and CUL4 associated factor 8	NMD
DCC	DCC	DCC netrin 1 receptor	ITHACA_RND
DCHS1	DCHS1	dachsous cadherin-related 1	ITHACA_RND
DCLK1	DCLK1	doublecortin like kinase 1	ITHACA
DCPS	DCPS	decapping enzyme, scavenger	ITHACA_RND
DCTN1	DCTN1	dynactin subunit 1	NMD_RND
DCTN2	DCTN2	dynactin subunit 2	ITHACA
DCTN5	DCTN5	dynactin subunit 5	ITHACA
DCX	DCX	doublecortin	ITHACA_RND
DCXR	DCXR	dicarbonyl and L-xylulose reductase	RND
DDB1	DDB1	damage specific DNA binding protein 1	ITHACA
DDB2	DDB2	damage specific DNA binding protein 2	GENTURIS_RND
DDC	DDC	dopa decarboxylase	ITHACA_RND
DDHD1	DDHD1	DDHD domain containing 1	ITHACA_NMD_RND
DDHD2	DDHD2	DDHD domain containing 2	ITHACA_NMD_RND
DDOST	DDOST	dolichyl-diphosphooligosaccharide--protein glycosyltransferase non-catalytic subunit	ITHACA_RND
DDR2	DDR2	discoidin domain receptor tyrosine kinase 2	ITHACA
DDX1	DDX1	DEAD-box helicase 1	ITHACA
DDX11	DDX11	DEAD/H-box helicase 11	GENTURIS_ITHACA_RND
DDX23	DDX23	DEAD-box helicase 23	ITHACA
DDX24	DDX24	DEAD-box helicase 24	ITHACA
DDX3X	DDX3X	DEAD-box helicase 3 X-linked	ITHACA_RND
DDX47	DDX47	DEAD-box helicase 47	ITHACA
DDX50	DDX50	DExD-box helicase 50	ITHACA
DDX53	DDX53	DEAD-box helicase 53	ITHACA
DDX54	DDX54	DEAD-box helicase 54	ITHACA
DDX59	DDX59	DEAD-box helicase 59	ITHACA_RND
DDX6	DDX6	DEAD-box helicase 6	ITHACA_RND
DEAF1	DEAF1	DEAF1 transcription factor	ITHACA_RND
DEGS1	DEGS1	delta 4-desaturase, sphingolipid 1	ITHACA_RND
DENND2A	DENND2A	DENN domain containing 2A	ITHACA
DENND5A	DENND5A	DENN domain containing 5A	ITHACA_RND
DEPDC5	DEPDC5	DEP domain containing 5, GATOR1 subcomplex subunit	ITHACA_RND
DES	DES	desmin	NMD_RND
DGAT2	DGAT2	diacylglycerol O-acyltransferase 2	NMD
DGCR14	ESS2	ess-2 splicing factor homolog	ITHACA
DGCR2	DGCR2	DiGeorge syndrome critical region gene 2	ITHACA
DGCR6	DGCR6	DiGeorge syndrome critical region gene 6	ITHACA
DGCR8	DGCR8	DGCR8 microprocessor complex subunit	ITHACA
DGKZ	DGKZ	diacylglycerol kinase zeta	ITHACA
DGUOK	DGUOK	deoxyguanosine kinase	NMD_RND
DHCR24	DHCR24	24-dehydrocholesterol reductase	ITHACA_RND

DHCR7	DHCR7	7-dehydrocholesterol reductase	ITHACA_RND
DHDDS	DHDDS	dehydrodolichyl diphosphate synthase subunit	ITHACA_RND
DHFR	DHFR	dihydrofolate reductase	ITHACA_RND
DHODH	DHODH	dihydroorotate dehydrogenase (quinone)	RND
DHPS	DHPS	deoxyhypusine synthase	ITHACA_RND
DHRS3	DHRS3	dehydrogenase/reductase 3	ITHACA
DHTKD1	DHTKD1	dehydrogenase E1 and transketolase domain containing 1	ITHACA_NMD_RND
DHX16	DHX16	DEAH-box helicase 16	ITHACA
DHX30	DHX30	DExH-box helicase 30	ITHACA_RND
DHX32	DHX32	DEAH-box helicase 32 (putative)	ITHACA
DHX34	DHX34	DExH-box helicase 34	ITHACA
DHX37	DHX37	DEAH-box helicase 37	ITHACA
DHX58	DHX58	DExH-box helicase 58	ITHACA
DIAPH1	DIAPH1	diaphanous related formin 1	ITHACA_RND
DIAPH2	DIAPH2	diaphanous related formin 2	ITHACA
DICER1	DICER1	dicer 1, ribonuclease III	GENTURIS_ITHACA
DIP2A	DIP2A	disco interacting protein 2 homolog A	ITHACA
DIP2B	DIP2B	disco interacting protein 2 homolog B	ITHACA
DIP2C	DIP2C	disco interacting protein 2 homolog C	ITHACA
DIS3L2	DIS3L2	DIS3 like 3'-5' exoribonuclease 2	GENTURIS_ITHACA_RND
DISP1	DISP1	dispatched RND transporter family member 1	ITHACA_RND
DKC1	DKC1	dyskerin pseudouridine synthase 1	GENTURIS_ITHACA_RND
DLAT	DLAT	dihydrolipoamide S-acetyltransferase	ITHACA_RND
DLC1	DLC1	DLC1 Rho GTPase activating protein	ITHACA
DLD	DLD	dihydrolipoamide dehydrogenase	ITHACA_RND
DLG1	DLG1	discs large MAGUK scaffold protein 1	ITHACA
DLG2	DLG2	discs large MAGUK scaffold protein 2	ITHACA
DLG3	DLG3	discs large MAGUK scaffold protein 3	ITHACA_RND
DLG4	DLG4	discs large MAGUK scaffold protein 4	ITHACA_RND
DLG5	DLG5	discs large MAGUK scaffold protein 5	ITHACA
DLGAP1	DLGAP1	DLG associated protein 1	ITHACA
DLL1	DLL1	delta like canonical Notch ligand 1	ITHACA
DLL4	DLL4	delta like canonical Notch ligand 4	ITHACA
DLX3	DLX3	distal-less homeobox 3	ITHACA
DLX5	DLX5	distal-less homeobox 5	ITHACA
DLX6	DLX6	distal-less homeobox 6	ITHACA
DMBT1	DMBT1	deleted in malignant brain tumors 1	ITHACA
DMBX1	DMBX1	diencephalon/mesencephalon homeobox 1	ITHACA
DMD	DMD	dystrophin	ITHACA_NMD_RND
DMKN	DMKN	dermokine	ITHACA
DMPK	DMPK	DM1 protein kinase	ITHACA_NMD
DMXL2	DMXL2	Dmx like 2	ITHACA_RND
DNA2	DNA2	DNA replication helicase/nuclease 2	ITHACA_NMD_RND
DNAH3	DNAH3	dynein axonemal heavy chain 3	ITHACA

DNAJA1	DNAJA1	DnaJ heat shock protein family (Hsp40) member A1	ITHACA
DNAJA3	DNAJA3	DnaJ heat shock protein family (Hsp40) member A3	ITHACA
DNAJB2	DNAJB2	DnaJ heat shock protein family (Hsp40) member B2	NMD_RND
DNAJB6	DNAJB6	DnaJ heat shock protein family (Hsp40) member B6	NMD_RND
DNAJC12	DNAJC12	DnaJ heat shock protein family (Hsp40) member C12	ITHACA_RND
DNAJC19	DNAJC19	DnaJ heat shock protein family (Hsp40) member C19	ITHACA_RND
DNAJC21	DNAJC21	DnaJ heat shock protein family (Hsp40) member C21	GENTURIS_ITHACA
DNAJC5	DNAJC5	DnaJ heat shock protein family (Hsp40) member C5	RND
DNAJC6	DNAJC6	DnaJ heat shock protein family (Hsp40) member C6	ITHACA_RND
DNHD1	DNHD1	dynein heavy chain domain 1	ITHACA
DNM1	DNM1	dynamin 1	ITHACA_RND
DNM1L	DNM1L	dynamin 1 like	ITHACA_RND
DNM2	DNM2	dynamin 2	ITHACA_NMD_RND
DNM3	DNM3	dynamin 3	ITHACA
DNMT1	DNMT1	DNA methyltransferase 1	NMD_RND
DNMT3A	DNMT3A	DNA methyltransferase 3 alpha	ITHACA_RND
DNMT3B	DNMT3B	DNA methyltransferase 3 beta	ITHACA_RND
DOCK3	DOCK3	dedicator of cytokinesis 3	ITHACA_RND
DOCK6	DOCK6	dedicator of cytokinesis 6	ITHACA_RND
DOCK7	DOCK7	dedicator of cytokinesis 7	ITHACA_RND
DOCK8	DOCK8	dedicator of cytokinesis 8	ITHACA_RND
DOK7	DOK7	docking protein 7	NMD_RND
DOLK	DOLK	dolichol kinase	ITHACA_NMD_RND
DONSON	DONSON	DNA replication fork stabilization factor DONSON	ITHACA
DOPEY1	DOP1A	DOP1 leucine zipper like protein A	ITHACA
DPAGT1	DPAGT1	dolichyl-phosphate N-acetylglucosaminephosphotransferase 1	ITHACA_NMD_RND
DPF2	DPF2	double PHD fingers 2	ITHACA_RND
DPH1	DPH1	diphthamide biosynthesis 1	ITHACA_RND
DPH2	DPH2	diphthamide biosynthesis 2	ITHACA
DPM1	DPM1	dolichyl-phosphate mannosyltransferase subunit 1, catalytic	ITHACA_NMD_RND
DPM2	DPM2	dolichyl-phosphate mannosyltransferase subunit 2, regulatory	ITHACA_NMD_RND
DPM3	DPM3	dolichyl-phosphate mannosyltransferase subunit 3, regulatory	ITHACA_NMD_RND
DPP6	DPP6	dipeptidyl peptidase like 6	ITHACA_RND
DPYD	DPYD	dihydropyrimidine dehydrogenase	ITHACA_RND
DPYS	DPYS	dihydropyrimidinase	ITHACA_RND
DPYSL2	DPYSL2	dihydropyrimidinase like 2	ITHACA

DPYSL3	DPYSL3	dihydropyrimidinase like 3	ITHACA
DRP2	DRP2	dystrophin related protein 2	ITHACA
DSC2	DSC2	desmocollin 2	NMD
DSCAM	DSCAM	DS cell adhesion molecule	ITHACA
DSE	DSE	dermatan sulfate epimerase	ITHACA
DSG2	DSG2	desmoglein 2	NMD
DSP	DSP	desmoplakin	NMD
DST	DST	dystonin	ITHACA_NMD_RND
DSTYK	DSTYK	dual serine/threonine and tyrosine protein kinase	ITHACA_RND
DTNA	DTNA	dystrobrevin alpha	NMD
DTYMK	DTYMK	deoxythymidylate kinase	ITHACA
DUOX1	DUOX1	dual oxidase 1	ITHACA
DUOX2	DUOX2	dual oxidase 2	ITHACA
DUOXA2	DUOXA2	dual oxidase maturation factor 2	ITHACA
DUX4	DUX4	double homeobox 4	NMD
DVL1	DVL1	dishevelled segment polarity protein 1	ITHACA
DVL2	DVL2	dishevelled segment polarity protein 2	ITHACA
DVL3	DVL3	dishevelled segment polarity protein 3	ITHACA
DYM	DYM	dymeclin	ITHACA_RND
DYNC1H1	DYNC1H1	dynein cytoplasmic 1 heavy chain 1	ITHACA_NMD_RND
DYNC1I2	DYNC1I2	dynein cytoplasmic 1 intermediate chain 2	ITHACA
DYRK1A	DYRK1A	dual specificity tyrosine phosphorylation regulated kinase 1A	ITHACA_RND
DYRK4	DYRK4	dual specificity tyrosine phosphorylation regulated kinase 4	ITHACA
DYSF	DYSF	dysferlin	NMD_RND
EARS2	EARS2	glutamyl-tRNA synthetase 2, mitochondrial	ITHACA_RND
EBF3	EBF3	EBF transcription factor 3	ITHACA_RND
EBP	EBP	EBP cholestenol delta-isomerase	ITHACA_RND
ECE2	ECE2	endothelin converting enzyme 2	ITHACA
ECEL1	ECEL1	endothelin converting enzyme like 1	RND
ECHS1	ECHS1	enoyl-CoA hydratase, short chain 1	ITHACA_RND
ECI1	ECI1	enoyl-CoA delta isomerase 1	ITHACA
ECM1	ECM1	extracellular matrix protein 1	ITHACA
ECM2	ECM2	extracellular matrix protein 2	ITHACA
EDC3	EDC3	enhancer of mRNA decapping 3	ITHACA
EDNRB	EDNRB	endothelin receptor type B	ITHACA
EED	EED	embryonic ectoderm development	ITHACA_RND
EEF1A2	EEF1A2	eukaryotic translation elongation factor 1 alpha 2	ITHACA_RND
EEF1B2	EEF1B2	eukaryotic translation elongation factor 1 beta 2	ITHACA
EEF1D	EEF1D	eukaryotic translation elongation factor 1 delta	ITHACA
EEF2	EEF2	eukaryotic translation elongation factor 2	ITHACA_NMD
EFCAB5	EFCAB5	EF-hand calcium binding domain 5	ITHACA
EFNB1	EFNB1	ephrin B1	ITHACA
EFNB2	EFNB2	ephrin B2	ITHACA
EFTUD1	EFL1	elongation factor like GTPase 1	ITHACA
EFTUD2	EFTUD2	elongation factor Tu GTP binding domain containing 2	ITHACA_RND
EGF	EGF	epidermal growth factor	ITHACA

EGFR	EGFR	epidermal growth factor receptor	GENTURIS
EGLN2	EGLN2	egl-9 family hypoxia inducible factor 2	ITHACA
EGR1	EGR1	early growth response 1	ITHACA
EGR2	EGR2	early growth response 2	ITHACA_NMD_RND
EHMT1	EHMT1	euchromatic histone lysine methyltransferase 1	ITHACA_RND
EI24	EI24	EI24 autophagy associated transmembrane protein	ITHACA
EIF2A	EIF2A	eukaryotic translation initiation factor 2A	ITHACA
EIF2AK1	EIF2AK1	eukaryotic translation initiation factor 2 alpha kinase 1	ITHACA
EIF2AK2	EIF2AK2	eukaryotic translation initiation factor 2 alpha kinase 2	ITHACA
EIF2AK3	EIF2AK3	eukaryotic translation initiation factor 2 alpha kinase 3	ITHACA_RND
EIF2B1	EIF2B1	eukaryotic translation initiation factor 2B subunit alpha	ITHACA_RND
EIF2B2	EIF2B2	eukaryotic translation initiation factor 2B subunit beta	ITHACA_RND
EIF2B3	EIF2B3	eukaryotic translation initiation factor 2B subunit gamma	ITHACA_RND
EIF2B4	EIF2B4	eukaryotic translation initiation factor 2B subunit delta	ITHACA_RND
EIF2B5	EIF2B5	eukaryotic translation initiation factor 2B subunit epsilon	ITHACA_RND
EIF2S3	EIF2S3	eukaryotic translation initiation factor 2 subunit gamma	ITHACA_RND
EIF3F	EIF3F	eukaryotic translation initiation factor 3 subunit F	ITHACA_RND
EIF4A1	EIF4A1	eukaryotic translation initiation factor 4A1	ITHACA
EIF4A2	EIF4A2	eukaryotic translation initiation factor 4A2	ITHACA
EIF4A3	EIF4A3	eukaryotic translation initiation factor 4A3	ITHACA_RND
EIF4G1	EIF4G1	eukaryotic translation initiation factor 4 gamma 1	ITHACA
EIF5A	EIF5A	eukaryotic translation initiation factor 5A	ITHACA
EIF6	EIF6	eukaryotic translation initiation factor 6	ITHACA
ELAC2	ELAC2	elaC ribonuclease Z 2	ITHACA_RND
ELANE	ELANE	elastase, neutrophil expressed	GENTURIS
ELMO2	ELMO2	engulfment and cell motility 2	ITHACA
ELMOD1	ELMOD1	ELMO domain containing 1	ITHACA
ELN	ELN	elastin	ITHACA
ELOVL4	ELOVL4	ELOVL fatty acid elongase 4	ITHACA_NMD_RND
ELOVL5	ELOVL5	ELOVL fatty acid elongase 5	NMD
ELP2	ELP2	elongator acetyltransferase complex subunit 2	ITHACA_RND
EMC1	EMC1	ER membrane protein complex subunit 1	ITHACA_RND
EMC10	EMC10	ER membrane protein complex subunit 10	ITHACA
EMC8	EMC8	ER membrane protein complex subunit 8	ITHACA
EMD	EMD	emerin	NMD_RND
EMG1	EMG1	EMG1 N1-specific pseudouridine methyltransferase	ITHACA
EML1	EML1	EMAP like 1	ITHACA_RND
EML3	EML3	EMAP like 3	ITHACA

EMX2	EMX2	empty spiracles homeobox 2	ITHACA_RND
ENG	ENG	endoglin	GENTURIS
ENO2	ENO2	enolase 2	ITHACA
ENO3	ENO3	enolase 3	ITHACA_NMD_RND
ENTPD1	ENTPD1	ectonucleoside triphosphate diphosphohydrolase 1	ITHACA_NMD_RND
EOGT	EOGT	EGF domain specific O-linked N-acetylglucosamine transferase	ITHACA
EP300	EP300	E1A binding protein p300	ITHACA_RND
EPB41L1	EPB41L1	erythrocyte membrane protein band 4.1 like 1	ITHACA
EPB41L4A	EPB41L4A	erythrocyte membrane protein band 4.1 like 4A	ITHACA
EPCAM	EPCAM	epithelial cell adhesion molecule	GENTURIS
EPG5	EPG5	ectopic P-granules 5 autophagy tethering factor	ITHACA_RND
EPHA5	EPHA5	EPH receptor A5	ITHACA
EPHB2	EPHB2	EPH receptor B2	ITHACA
EPHB6	EPHB6	EPH receptor B6	ITHACA
EPM2A	EPM2A	EPM2A glucan phosphatase, laforin	RND
EPRS	EPRS1	glutamyl-prolyl-tRNA synthetase 1	ITHACA
EPT1	SELENOI	selenoprotein I	ITHACA
ERBB3	ERBB3	erb-b2 receptor tyrosine kinase 3	NMD
ERBB4	ERBB4	erb-b2 receptor tyrosine kinase 4	ITHACA_NMD
ERCC1	ERCC1	ERCC excision repair 1, endonuclease non-catalytic subunit	GENTURIS_ITHACA_RND
ERCC2	ERCC2	ERCC excision repair 2, TFIIH core complex helicase subunit	GENTURIS_ITHACA_RND
ERCC3	ERCC3	ERCC excision repair 3, TFIIH core complex helicase subunit	GENTURIS_ITHACA_RND
ERCC4	ERCC4	ERCC excision repair 4, endonuclease catalytic subunit	GENTURIS_ITHACA_RND
ERCC5	ERCC5	ERCC excision repair 5, endonuclease	GENTURIS_ITHACA_RND
ERCC6	ERCC6	ERCC excision repair 6, chromatin remodeling factor	GENTURIS_ITHACA_RND
ERCC6L2	ERCC6L2	ERCC excision repair 6 like 2	ITHACA_RND
ERCC8	ERCC8	ERCC excision repair 8, CSA ubiquitin ligase complex subunit	ITHACA_RND
ERF	ERF	ETS2 repressor factor	ITHACA_RND
ERLIN1	ERLIN1	ER lipid raft associated 1	ITHACA_NMD_RND
ERLIN2	ERLIN2	ER lipid raft associated 2	ITHACA_NMD_RND
ERMARD	ERMARD	ER membrane associated RNA degradation	ITHACA
ESCO2	ESCO2	establishment of sister chromatid cohesion N-acetyltransferase 2	ITHACA_RND
ESR2	ESR2	estrogen receptor 2	GENTURIS
ETFA	ETFA	electron transfer flavoprotein subunit alpha	ITHACA_NMD_RND
ETFB	ETFB	electron transfer flavoprotein subunit beta	ITHACA_NMD_RND

ETFDH	ETFDH	electron transfer flavoprotein dehydrogenase	ITHACA_NMD_RND
ETHE1	ETHE1	ETHE1 persulfide dioxygenase	ITHACA_RND
ETV6	ETV6	ETS variant transcription factor 6	GENTURIS
EVC	EVC	EvC ciliary complex subunit 1	ITHACA_RND
EVC2	EVC2	EvC ciliary complex subunit 2	ITHACA_RND
EXO1	EXO1	exonuclease 1	GENTURIS
EXOC2	EXOC2	exocyst complex component 2	ITHACA
EXOC3L2	EXOC3L2	exocyst complex component 3 like 2	ITHACA
EXOC7	EXOC7	exocyst complex component 7	ITHACA
EXOC8	EXOC8	exocyst complex component 8	ITHACA
EXOSC2	EXOSC2	exosome component 2	ITHACA
EXOSC3	EXOSC3	exosome component 3	ITHACA_NMD_RND
EXOSC5	EXOSC5	exosome component 5	ITHACA
EXOSC8	EXOSC8	exosome component 8	ITHACA_NMD_RND
EXOSC9	EXOSC9	exosome component 9	ITHACA_RND
EXT1	EXT1	exostosin glycosyltransferase 1	GENTURIS_RND
EXT2	EXT2	exostosin glycosyltransferase 2	GENTURIS_ITHACA_RND
EXTL3	EXTL3	exostosin like glycosyltransferase 3	ITHACA_RND
EYA1	EYA1	EYA transcriptional coactivator and phosphatase 1	ITHACA
EYA4	EYA4	EYA transcriptional coactivator and phosphatase 4	NMD
EZH2	EZH2	enhancer of zeste 2 polycomb repressive complex 2 subunit	GENTURIS_ITHACA_RND
EZR	EZR	ezrin	ITHACA
FA2H	FA2H	fatty acid 2-hydroxylase	ITHACA_NMD_RND
FAAH2	FAAH2	fatty acid amide hydrolase 2	ITHACA
FAH	FAH	fumarylacetoacetate hydrolase	ITHACA_RND
FAM104A	FAM104A	family with sequence similarity 104 member A	ITHACA
FAM111A	FAM111A	FAM111 trypsin like peptidase A	ITHACA_RND
FAM111B	FAM111B	FAM111 trypsin like peptidase B	NMD
FAM120AOS	FAM120AOS	family with sequence similarity 120A opposite strand	ITHACA
FAM120C	FAM120C	family with sequence similarity 120C	ITHACA
FAM126A	HYCC1	hyccin PI4KA lipid kinase complex subunit 1	ITHACA_RND
FAM134B	RETREG1	reticulophagy regulator 1	NMD_RND
FAM149B1	FAM149B1	family with sequence similarity 149 member B1	ITHACA
FAM160B1	FHIP2A	FHF complex subunit HOOK interacting protein 2A	ITHACA
FAM177A1	FAM177A1	family with sequence similarity 177 member A1	ITHACA
FAM179B	TOGARAM1	TOG array regulator of axonemal microtubules 1	ITHACA
FAM183A	FAM183A	family with sequence similarity 183 member A	ITHACA
FAM19A1	TAFA1	TAFA chemokine like family member 1	ITHACA
FAM200A	FAM200A	family with sequence similarity 200 member A	ITHACA
FAM200B	FAM200B	family with sequence similarity 200 member B	ITHACA
FAM20C	FAM20C	FAM20C golgi associated secretory pathway kinase	ITHACA_RND
FAM222A	FAM222A	family with sequence similarity 222 member A	ITHACA

FAM46A	TENT5A	terminal nucleotidyltransferase 5A	ITHACA
FAM50A	FAM50A	family with sequence similarity 50 member A	ITHACA
FAM8A1	FAM8A1	family with sequence similarity 8 member A1	ITHACA
FAM91A1	FAM91A1	family with sequence similarity 91 member A1	ITHACA
FAN1	FAN1	FANCD2 and FANCI associated nuclease 1	GENTURIS
FANCA	FANCA	FA complementation group A	GENTURIS_ITHACA
FANCB	FANCB	FA complementation group B	GENTURIS_ITHACA_RND
FANCC	FANCC	FA complementation group C	GENTURIS_ITHACA
FANCD2	FANCD2	FA complementation group D2	GENTURIS_ITHACA
FANCE	FANCE	FA complementation group E	GENTURIS_ITHACA
FANCF	FANCF	FA complementation group F	GENTURIS_ITHACA
FANCG	FANCG	FA complementation group G	GENTURIS_ITHACA
FANCI	FANCI	FA complementation group I	GENTURIS_ITHACA
FANCL	FANCL	FA complementation group L	GENTURIS_ITHACA
FANCM	FANCM	FA complementation group M	GENTURIS
FAR1	FAR1	fatty acyl-CoA reductase 1	ITHACA_RND
FARS2	FARS2	phenylalanyl-tRNA synthetase 2, mitochondrial	ITHACA_NMD_RND
FARSA	FARSA	phenylalanyl-tRNA synthetase subunit alpha	ITHACA
FARSB	FARSB	phenylalanyl-tRNA synthetase subunit beta	ITHACA
FAS	FAS	Fas cell surface death receptor	GENTURIS
FASN	FASN	fatty acid synthase	ITHACA
FASTKD2	FASTKD2	FAST kinase domains 2	ITHACA_NMD_RND
FAT1	FAT1	FAT atypical cadherin 1	ITHACA
FAT2	FAT2	FAT atypical cadherin 2	NMD
FAT4	FAT4	FAT atypical cadherin 4	ITHACA_RND
FBLN5	FBLN5	fibulin 5	NMD_RND
FBN2	FBN2	fibrillin 2	ITHACA
FBP1	FBP1	fructose-bisphosphatase 1	RND
FBRSL1	FBRSL1	fibrosin like 1	ITHACA
FBXL3	FBXL3	F-box and leucine rich repeat protein 3	ITHACA_RND
FBXL4	FBXL4	F-box and leucine rich repeat protein 4	ITHACA_NMD_RND
FBXL7	FBXL7	F-box and leucine rich repeat protein 7	ITHACA
FBXO10	FBXO10	F-box protein 10	ITHACA
FBXO11	FBXO11	F-box protein 11	ITHACA_RND
FBXO28	FBXO28	F-box protein 28	ITHACA
FBXO31	FBXO31	F-box protein 31	ITHACA
FBXO38	FBXO38	F-box protein 38	NMD
FBXO47	FBXO47	F-box protein 47	ITHACA
FBXO7	FBXO7	F-box protein 7	RND

FBXW11	FBXW11	F-box and WD repeat domain containing 11	ITHACA_RND
FBXW7	FBXW7	F-box and WD repeat domain containing 7	GENTURIS_ITHACA
FCRL6	FCRL6	Fc receptor like 6	ITHACA
FDFT1	FDFT1	farnesyl-diphosphate farnesyltransferase 1	ITHACA
FDPS	FDPS	farnesyl diphosphate synthase	ITHACA
FDX1L	FDX2	ferredoxin 2	ITHACA_NMD_RND
FDXR	FDXR	ferredoxin reductase	ITHACA_RND
FECH	FECH	ferrochelatase	RND
FEM1B	FEM1B	fem-1 homolog B	ITHACA
FEV	FEV	FEV transcription factor, ETS family member	ITHACA
FEZF2	FEZF2	FEZ family zinc finger 2	ITHACA
FGD1	FGD1	FYVE, RhoGEF and PH domain containing 1	ITHACA_RND
FGD4	FGD4	FYVE, RhoGEF and PH domain containing 4	ITHACA_NMD_RND
FGF12	FGF12	fibroblast growth factor 12	ITHACA_RND
FGF13	FGF13	fibroblast growth factor 13	ITHACA
FGF14	FGF14	fibroblast growth factor 14	ITHACA_NMD_RND
FGF3	FGF3	fibroblast growth factor 3	ITHACA
FGF8	FGF8	fibroblast growth factor 8	RND
FGFR1	FGFR1	fibroblast growth factor receptor 1	ITHACA_RND
FGFR2	FGFR2	fibroblast growth factor receptor 2	ITHACA_RND
FGFR3	FGFR3	fibroblast growth factor receptor 3	ITHACA_RND
FGFRL1	FGFRL1	fibroblast growth factor receptor like 1	ITHACA
FH	FH	fumarate hydratase	GENTURIS_ITHACA_RND
FHL1	FHL1	four and a half LIM domains 1	NMD_RND
FIBP	FIBP	FGF1 intracellular binding protein	ITHACA
FIG4	FIG4	FIG4 phosphoinositide 5-phosphatase	ITHACA_NMD_RND
FIGN	FIGN	fidgetin, microtubule severing factor	ITHACA
FITM2	FITM2	fat storage inducing transmembrane protein 2	ITHACA
FKBP14	FKBP14	FKBP prolyl isomerase 14	ITHACA_RND
FKRP	FKRP	fukutin related protein	ITHACA_NMD_RND
FKTN	FKTN	fukutin	ITHACA_NMD_RND
FLAD1	FLAD1	flavin adenine dinucleotide synthetase 1	ITHACA_NMD_RND
FLCN	FLCN	folliculin	GENTURIS_ITHACA
FLG	FLG	filaggrin	ITHACA
FLNA	FLNA	filamin A	ITHACA_NMD_RND
FLNB	FLNB	filamin B	ITHACA
FLNC	FLNC	filamin C	NMD_RND
FLVCR1	FLVCR1	FLVCR heme transporter 1	ITHACA_NMD_RND
FLVCR2	FLVCR2	FLVCR heme transporter 2	ITHACA_RND

FMN2	FMN2	formin 2	ITHACA_RND
FMO3	FMO3	flavin containing dimethylaniline monoxygenase 3	RND
FMO4	FMO4	flavin containing dimethylaniline monoxygenase 4	ITHACA
FMOD	FMOD	fibromodulin	ITHACA
FMR1	FMR1	fragile X messenger ribonucleoprotein 1	ITHACA_RND
FNDC3A	FNDC3A	fibronectin type III domain containing 3A	ITHACA
FOLR1	FOLR1	folate receptor alpha	ITHACA_RND
FOSL2	FOSL2	FOS like 2, AP-1 transcription factor subunit	ITHACA
FOXF1	FOXF1	forkhead box F1	ITHACA
FOXG1	FOXG1	forkhead box G1	ITHACA_RND
FOXI1	FOXI1	forkhead box I1	ITHACA
FOXJ1	FOXJ1	forkhead box J1	ITHACA
FOXO3	FOXO3	forkhead box O3	GENTURIS
FOXP1	FOXP1	forkhead box P1	ITHACA_RND
FOXP2	FOXP2	forkhead box P2	ITHACA_RND
FOXP3	FOXP3	forkhead box P3	ITHACA
FOXP4	FOXP4	forkhead box P4	ITHACA
FOXR2	FOXR2	forkhead box R2	ITHACA
FOXRED1	FOXRED1	FAD dependent oxidoreductase domain containing 1	ITHACA_RND
FRAS1	FRAS1	Fraser extracellular matrix complex subunit 1	ITHACA
FREM1	FREM1	FRAS1 related extracellular matrix 1	ITHACA
FREM2	FREM2	FRAS1 related extracellular matrix 2	ITHACA
FREM3	FREM3	FRAS1 related extracellular matrix 3	ITHACA
FRG1	FRG1	FSHD region gene 1	ITHACA
FRMD4A	FRMD4A	FERM domain containing 4A	ITHACA
FRMPD4	FRMPD4	FERM and PDZ domain containing 4	ITHACA_RND
FRRS1L	FRRS1L	ferric chelate reductase 1 like	ITHACA_RND
FRY	FRY	FRY microtubule binding protein	ITHACA
FSCN1	FSCN1	fascin actin-bundling protein 1	ITHACA
FTCD	FTCD	formimidoyltransferase cyclodeaminase	ITHACA_RND
FTL	FTL	ferritin light chain	RND
FTO	FTO	FTO alpha-ketoglutarate dependent dioxygenase	ITHACA
FTSJ1	FTSJ1	FtsJ RNA 2'-O-methyltransferase 1	ITHACA_RND
FTSJ2	MRM2	mitochondrial rRNA methyltransferase 2	ITHACA
FUCA1	FUCA1	alpha-L-fucosidase 1	ITHACA_RND
FUK	FCSK	fucose kinase	ITHACA
FUS	FUS	FUS RNA binding protein	NMD_RND
FUT8	FUT8	fucosyltransferase 8	ITHACA_RND
FXN	FXN	frataxin	NMD_RND
FXR1	FXR1	FMR1 autosomal homolog 1	ITHACA_NMD_RND
FZD2	FZD2	frizzled class receptor 2	ITHACA
FZD3	FZD3	frizzled class receptor 3	ITHACA
G6PC	G6PC1	glucose-6-phosphatase catalytic subunit 1	RND
G6PC3	G6PC3	glucose-6-phosphatase catalytic subunit 3	GENTURIS_ITHACA_RND
G6PD	G6PD	glucose-6-phosphate dehydrogenase	ITHACA
GAA	GAA	alpha glucosidase	NMD_RND
GABBR2	GABBR2	gamma-aminobutyric acid type B receptor subunit 2	ITHACA_RND

GABRA1	GABRA1	gamma-aminobutyric acid type A receptor subunit alpha1	ITHACA_RND
GABRA2	GABRA2	gamma-aminobutyric acid type A receptor subunit alpha2	ITHACA_RND
GABRA3	GABRA3	gamma-aminobutyric acid type A receptor subunit alpha3	ITHACA
GABRA5	GABRA5	gamma-aminobutyric acid type A receptor subunit alpha5	ITHACA_RND
GABRB1	GABRB1	gamma-aminobutyric acid type A receptor subunit beta1	ITHACA
GABRB2	GABRB2	gamma-aminobutyric acid type A receptor subunit beta2	ITHACA_RND
GABRB3	GABRB3	gamma-aminobutyric acid type A receptor subunit beta3	ITHACA_RND
GABRE	GABRE	gamma-aminobutyric acid type A receptor subunit epsilon	ITHACA
GABRG2	GABRG2	gamma-aminobutyric acid type A receptor subunit gamma2	ITHACA_RND
GAD1	GAD1	glutamate decarboxylase 1	ITHACA_RND
GALC	GALC	galactosylceramidase	ITHACA_RND
GALE	GALE	UDP-galactose-4-epimerase	ITHACA_RND
GALK1	GALK1	galactokinase 1	RND
GALNS	GALNS	galactosamine (N-acetyl)-6-sulfatase	ITHACA_RND
GALNT12	GALNT12	polypeptide N-acetylgalactosaminyltransferase 12	GENTURIS
GALNT18	GALNT18	polypeptide N-acetylgalactosaminyltransferase 18	ITHACA
GALNT2	GALNT2	polypeptide N-acetylgalactosaminyltransferase 2	ITHACA
GALNT3	GALNT3	polypeptide N-acetylgalactosaminyltransferase 3	RND
GALT	GALT	galactose-1-phosphate uridylyltransferase	ITHACA_RND
GAMT	GAMT	guanidinoacetate N-methyltransferase	ITHACA_RND
GAN	GAN	gigaxonin	ITHACA_NMD_RND
GARNL3	GARNL3	GTPase activating Rap/RanGAP domain like 3	ITHACA
GARS	GARS1	glycyl-tRNA synthetase 1	ITHACA_NMD_RND
GATA1	GATA1	GATA binding protein 1	ITHACA
GATA2	GATA2	GATA binding protein 2	GENTURIS
GATA6	GATA6	GATA binding protein 6	ITHACA
GATAD1	GATAD1	GATA zinc finger domain containing 1	NMD
GATAD2B	GATAD2B	GATA zinc finger domain containing 2B	ITHACA_RND
GATM	GATM	glycine amidinotransferase	ITHACA_RND
GBA	GBA1	glucosylceramidase beta 1	ITHACA_NMD_RND
GBA2	GBA2	glucosylceramidase beta 2	ITHACA_NMD_RND
GBE1	GBE1	1,4-alpha-glucan branching enzyme 1	NMD_RND
GBF1	GBF1	golgi brefeldin A resistant guanine nucleotide exchange factor 1	NMD
GCC2	GCC2	GRIP and coiled-coil domain containing 2	ITHACA
GCDH	GCDH	glutaryl-CoA dehydrogenase	ITHACA_RND
GCH1	GCH1	GTP cyclohydrolase 1	ITHACA_RND
GCK	GCK	glucokinase	ITHACA

GCLC	GCLC	glutamate-cysteine ligase catalytic subunit	RND
GCN1L1	GCN1	GCN1 activator of EIF2AK4	ITHACA
GCSH	GCSH	glycine cleavage system protein H	ITHACA
GDAP1	GDAP1	ganglioside induced differentiation associated protein 1	ITHACA_NMD_RND
GDAP2	GDAP2	ganglioside induced differentiation associated protein 2	NMD_RND
GDF1	GDF1	growth differentiation factor 1	ITHACA
GDI1	GDI1	GDP dissociation inhibitor 1	ITHACA_RND
GDNF	GDNF	glial cell derived neurotrophic factor	GENTURIS
GEMIN4	GEMIN4	gem nuclear organelle associated protein 4	ITHACA
GEMIN7	GEMIN7	gem nuclear organelle associated protein 7	ITHACA
GET4	GET4	guided entry of tail-anchored proteins factor 4	ITHACA
GFAP	GFAP	glial fibrillary acidic protein	ITHACA_RND
GFER	GFER	growth factor, augmenter of liver regeneration	ITHACA_RND
GFI1	GFI1	growth factor independent 1 transcriptional repressor	GENTURIS
GFM1	GFM1	G elongation factor mitochondrial 1	ITHACA_RND
GFM2	GFM2	GTP dependent ribosome recycling factor mitochondrial 2	ITHACA_RND
GFPT1	GFPT1	glutamine--fructose-6-phosphate transaminase 1	ITHACA_NMD_RND
GFPT2	GFPT2	glutamine-fructose-6-phosphate transaminase 2	ITHACA
GGN	GGN	gametogenetin	ITHACA
GGT1	GGT1	gamma-glutamyltransferase 1	ITHACA
GIF	CBLIF	cobalamin binding intrinsic factor	RND
GIGYF1	GIGYF1	GRB10 interacting GYF protein 1	ITHACA
GIGYF2	GIGYF2	GRB10 interacting GYF protein 2	ITHACA
GIMAP8	GIMAP8	GTPase, IMAP family member 8	ITHACA
GIPC1	GIPC1	GIPC PDZ domain containing family member 1	NMD
GJA1	GJA1	gap junction protein alpha 1	ITHACA_RND
GJA5	GJA5	gap junction protein alpha 5	ITHACA_NMD
GJA8	GJA8	gap junction protein alpha 8	ITHACA
GJB1	GJB1	gap junction protein beta 1	ITHACA_NMD_RND
GJB2	GJB2	gap junction protein beta 2	ITHACA
GJB3	GJB3	gap junction protein beta 3	ITHACA_NMD
GJC2	GJC2	gap junction protein gamma 2	ITHACA_NMD_RND
GK	GK	glycerol kinase	ITHACA_RND
GLA	GLA	galactosidase alpha	RND
GLB1	GLB1	galactosidase beta 1	ITHACA_RND
GLDC	GLDC	glycine decarboxylase	ITHACA_RND
GLDN	GLDN	gliomedin	NMD
GLE1	GLE1	GLE1 RNA export mediator	ITHACA_NMD
GLI2	GLI2	GLI family zinc finger 2	ITHACA_RND
GLI3	GLI3	GLI family zinc finger 3	ITHACA_RND
GLIS3	GLIS3	GLIS family zinc finger 3	ITHACA_RND
GLRA1	GLRA1	glycine receptor alpha 1	ITHACA_RND
GLRA2	GLRA2	glycine receptor alpha 2	ITHACA
GLRB	GLRB	glycine receptor beta	RND

GLRX5	GLRX5	glutaredoxin 5	RND
GLS	GLS	glutaminase	ITHACA_RND
GLTSCR1	BICRA	BRD4 interacting chromatin remodeling complex associated protein	ITHACA
GLUD1	GLUD1	glutamate dehydrogenase 1	ITHACA_RND
GLUL	GLUL	glutamate-ammonia ligase	ITHACA_RND
GLYCTK	GLYCTK	glycerate kinase	ITHACA_RND
GM2A	GM2A	ganglioside GM2 activator	ITHACA_RND
GMNN	GMNN	geminin DNA replication inhibitor	ITHACA
GMPPA	GMPPA	GDP-mannose pyrophosphorylase A	ITHACA_RND
GMPPB	GMPPB	GDP-mannose pyrophosphorylase B	ITHACA_NMD_RND
GNA11	GNA11	G protein subunit alpha 11	ITHACA
GNAI1	GNAI1	G protein subunit alpha i1	ITHACA_RND
GNAI2	GNAI2	G protein subunit alpha i2	ITHACA
GNAL	GNAL	G protein subunit alpha L	RND
GNAO1	GNAO1	G protein subunit alpha o1	ITHACA_RND
GNAQ	GNAQ	G protein subunit alpha q	ITHACA_RND
GNAS	GNAS	GNAS complex locus	ITHACA_RND
GNB1	GNB1	G protein subunit beta 1	ITHACA_RND
GNB2	GNB2	G protein subunit beta 2	ITHACA
GNB4	GNB4	G protein subunit beta 4	NMD_RND
GNB5	GNB5	G protein subunit beta 5	ITHACA_RND
GNE	GNE	glucosamine (UDP-N-acetyl)-2-epimerase/N-acetylmannosamine kinase	ITHACA_NMD_RND
GNMT	GNMT	glycine N-methyltransferase	RND
GNPAT	GNPAT	glyceronephosphate O-acyltransferase	ITHACA_RND
GNPTAB	GNPTAB	N-acetylglucosamine-1-phosphate transferase subunits alpha and beta	ITHACA_RND
GNPTG	GNPTG	N-acetylglucosamine-1-phosphate transferase subunit gamma	ITHACA_RND
GNS	GNS	glucosamine (N-acetyl)-6-sulfatase	ITHACA_RND
GOLGA2	GOLGA2	golgin A2	ITHACA_NMD
GON4L	GON4L	gon-4 like	ITHACA
GORAB	GORAB	golgin, RAB6 interacting	ITHACA
GOSR2	GOSR2	golgi SNAP receptor complex member 2	NMD_RND
GOT2	GOT2	glutamic-oxaloacetic transaminase 2	ITHACA_RND
GPAA1	GPAA1	glycosylphosphatidylinositol anchor attachment 1	ITHACA_RND
GPC3	GPC3	glypican 3	GENTURIS_ITHACA_RND
GPC4	GPC4	glypican 4	ITHACA
GPD1	GPD1	glycerol-3-phosphate dehydrogenase 1	RND
GPD1L	GPD1L	glycerol-3-phosphate dehydrogenase 1 like	NMD
GPHN	GPHN	gephyrin	ITHACA_RND
GPI	GPI	glucose-6-phosphate isomerase	ITHACA
GPM6A	GPM6A	glycoprotein M6A	ITHACA
GPR126	ADGRG6	adhesion G protein-coupled receptor G6	ITHACA_NMD
GPR139	GPR139	G protein-coupled receptor 139	ITHACA
GPR37	GPR37	G protein-coupled receptor 37	ITHACA
GPR52	GPR52	G protein-coupled receptor 52	ITHACA
GPR56	ADGRG1	adhesion G protein-coupled receptor G1	ITHACA_RND

GPR64	ADGRG2	adhesion G protein-coupled receptor G2	ITHACA
GPR88	GPR88	G protein-coupled receptor 88	ITHACA
GPS1	GPS1	G protein pathway suppressor 1	ITHACA
GPSM2	GPSM2	G protein signaling modulator 2	ITHACA_RND
GPT2	GPT2	glutamic--pyruvic transaminase 2	ITHACA_RND
GPX4	GPX4	glutathione peroxidase 4	ITHACA
GRAMD1B	GRAMD1B	GRAM domain containing 1B	ITHACA
GREM1	GREM1	gremlin 1, DAN family BMP antagonist	GENTURIS
GRHL2	GRHL2	grainyhead like transcription factor 2	GENTURIS
GRHPR	GRHPR	glyoxylate and hydroxypyruvate reductase	RND
GRIA1	GRIA1	glutamate ionotropic receptor AMPA type subunit 1	ITHACA
GRIA2	GRIA2	glutamate ionotropic receptor AMPA type subunit 2	ITHACA_RND
GRIA3	GRIA3	glutamate ionotropic receptor AMPA type subunit 3	ITHACA_RND
GRIA4	GRIA4	glutamate ionotropic receptor AMPA type subunit 4	ITHACA_RND
GRID2	GRID2	glutamate ionotropic receptor delta type subunit 2	ITHACA_NMD_RND
GRIK2	GRIK2	glutamate ionotropic receptor kainate type subunit 2	ITHACA_RND
GRIK4	GRIK4	glutamate ionotropic receptor kainate type subunit 4	ITHACA
GRIN1	GRIN1	glutamate ionotropic receptor NMDA type subunit 1	ITHACA_RND
GRIN2A	GRIN2A	glutamate ionotropic receptor NMDA type subunit 2A	ITHACA_RND
GRIN2B	GRIN2B	glutamate ionotropic receptor NMDA type subunit 2B	ITHACA_RND
GRIN2D	GRIN2D	glutamate ionotropic receptor NMDA type subunit 2D	ITHACA_RND
GRIP1	GRIP1	glutamate receptor interacting protein 1	ITHACA
GRM1	GRM1	glutamate metabotropic receptor 1	ITHACA_NMD_RND
GRM7	GRM7	glutamate metabotropic receptor 7	ITHACA
GRN	GRN	granulin precursor	ITHACA_RND
GSE1	GSE1	Gse1 coiled-coil protein	ITHACA
GSPT2	GSPT2	G1 to S phase transition 2	ITHACA
GSS	GSS	glutathione synthetase	ITHACA_RND
GSX2	GSX2	GS homeobox 2	ITHACA_RND
GTF2E2	GTF2E2	general transcription factor IIE subunit 2	ITHACA
GTF2H5	GTF2H5	general transcription factor IIH subunit 5	ITHACA_RND
GTF3C1	GTF3C1	general transcription factor IIIC subunit 1	ITHACA
GTF3C3	GTF3C3	general transcription factor IIIC subunit 3	ITHACA
GTPBP2	GTPBP2	GTP binding protein 2	ITHACA_RND
GTPBP3	GTPBP3	GTP binding protein 3, mitochondrial	ITHACA_RND
GUCY2D	GUCY2D	guanylate cyclase 2D, retinal	ITHACA
GUF1	GUF1	GTP binding elongation factor GUF1	ITHACA
GUSB	GUSB	glucuronidase beta	ITHACA_RND
GYG1	GYG1	glycogenin 1	NMD_RND

GYS1	GYS1	glycogen synthase 1	NMD_RND
GYS2	GYS2	glycogen synthase 2	RND
H3F3A	H3-3A	H3.3 histone A	ITHACA
H3F3B	H3-3B	H3.3 histone B	ITHACA
HAAO	HAAO	3-hydroxyanthranilate 3,4-dioxygenase	ITHACA_RND
HABP2	HABP2	hyaluronan binding protein 2	GENTURIS
HACE1	HACE1	HECT domain and ankyrin repeat containing E3 ubiquitin protein ligase 1	ITHACA_NMD_RND
HACL1	HACL1	2-hydroxyacyl-CoA lyase 1	ITHACA
HADH	HADH	hydroxyacyl-CoA dehydrogenase	ITHACA_RND
HADHA	HADHA	hydroxyacyl-CoA dehydrogenase trifunctional multienzyme complex subunit alpha	ITHACA_RND
HADHB	HADHB	hydroxyacyl-CoA dehydrogenase trifunctional multienzyme complex subunit beta	ITHACA_RND
HAMP	HAMP	hepcidin antimicrobial peptide	RND
HARS	HARS1	histidyl-tRNA synthetase 1	ITHACA_NMD_RND
HARS2	HARS2	histidyl-tRNA synthetase 2, mitochondrial	RND
HAX1	HAX1	HCLS1 associated protein X-1	GENTURIS_ITHACA_RND
HCCS	HCCS	holocytochrome c synthase	ITHACA_RND
HCFC1	HCFC1	host cell factor C1	ITHACA_RND
HCN1	HCN1	hyperpolarization activated cyclic nucleotide gated potassium channel 1	ITHACA_RND
HCN2	HCN2	hyperpolarization activated cyclic nucleotide gated potassium and sodium channel 2	ITHACA_RND
HCN4	HCN4	hyperpolarization activated cyclic nucleotide gated potassium channel 4	ITHACA_NMD
HCRT	HCRT	hypocretin neuropeptide precursor	ITHACA
HDAC1	HDAC1	histone deacetylase 1	ITHACA
HDAC3	HDAC3	histone deacetylase 3	ITHACA
HDAC4	HDAC4	histone deacetylase 4	ITHACA_RND
HDAC6	HDAC6	histone deacetylase 6	ITHACA
HDAC8	HDAC8	histone deacetylase 8	ITHACA_RND
HEATR5B	HEATR5B	HEAT repeat containing 5B	ITHACA
HECW1	HECW1	HECT, C2 and WW domain containing E3 ubiquitin protein ligase 1	ITHACA
HECW2	HECW2	HECT, C2 and WW domain containing E3 ubiquitin protein ligase 2	ITHACA_RND
HELLS	HELLS	helicase, lymphoid specific	ITHACA
HELZ	HELZ	helicase with zinc finger	ITHACA
HEMK1	HEMK1	HemK methyltransferase family member 1	ITHACA
HEPACAM	HEPACAM	hepatic and glial cell adhesion molecule	ITHACA_RND
HEPHL1	HEPHL1	hephaestin like 1	ITHACA
HERC1	HERC1	HECT and RLD domain containing E3 ubiquitin protein ligase family member 1	ITHACA_RND
HERC2	HERC2	HECT and RLD domain containing E3 ubiquitin protein ligase 2	ITHACA
HESX1	HESX1	HESX homeobox 1	ITHACA_RND
HEXA	HEXA	hexosaminidase subunit alpha	ITHACA_RND

HEXB	HEXB	hexosaminidase subunit beta	ITHACA_NMD_RND
HFE	HFE	homeostatic iron regulator	RND
HFE2	HJV	hemojuvelin BMP co-receptor	RND
HGD	HGD	homogentisate 1,2-dioxygenase	RND
HGSNAT	HGSNAT	heparan-alpha-glucosaminide N-acetyltransferase	ITHACA_RND
HHAT	HHAT	hedgehog acyltransferase	ITHACA
HIBCH	HIBCH	3-hydroxyisobutyryl-CoA hydrolase	ITHACA_RND
HID1	HID1	HID1 domain containing	ITHACA
HINT1	HINT1	histidine triad nucleotide binding protein 1	ITHACA_NMD_RND
HIRA	HIRA	histone cell cycle regulator	ITHACA
HIST1H1C	H1-2	H1.2 linker histone, cluster member	ITHACA
HIST1H1E	H1-4	H1.4 linker histone, cluster member	ITHACA_RND
HIST1H2AC	H2AC6	H2A clustered histone 6	ITHACA
HIST3H3	H3-4	H3.4 histone, cluster member	ITHACA
HIST4H4	H4C16	H4 histone 16	ITHACA
HIVEP2	HIVEP2	HIVEP zinc finger 2	ITHACA_RND
HIVEP3	HIVEP3	HIVEP zinc finger 3	ITHACA
HK1	HK1	hexokinase 1	ITHACA_NMD_RND
HLCS	HLCS	holocarboxylase synthetase	ITHACA_RND
HMBS	HMBS	hydroxymethylbilane synthase	RND
HMCN2	HMCN2	hemicentin 2	ITHACA
HMG20A	HMG20A	high mobility group 20A	ITHACA
HMGB3	HMGB3	high mobility group box 3	ITHACA
HMGCL	HMGCL	3-hydroxy-3-methylglutaryl-CoA lyase	ITHACA_RND
HMGCLL1	HMGCLL1	3-hydroxymethyl-3-methylglutaryl-CoA lyase like 1	ITHACA
HMGCS2	HMGCS2	3-hydroxy-3-methylglutaryl-CoA synthase 2	RND
HMGXB3	HMGXB3	HMG-box containing 3	ITHACA
HNF1A	HNF1A	HNF1 homeobox A	GENTURIS
HNF1B	HNF1B	HNF1 homeobox B	ITHACA
HNMT	HNMT	histamine N-methyltransferase	ITHACA
HNRNPA1	HNRNPA1	heterogeneous nuclear ribonucleoprotein A1	NMD_RND
HNRNPA2B1	HNRNPA2B1	heterogeneous nuclear ribonucleoprotein A2/B1	NMD
HNRNPD	HNRNPD	heterogeneous nuclear ribonucleoprotein D	ITHACA
HNRNPDL	HNRNPDL	heterogeneous nuclear ribonucleoprotein D like	NMD_RND
HNRNPH1	HNRNPH1	heterogeneous nuclear ribonucleoprotein H1	ITHACA
HNRNPH2	HNRNPH2	heterogeneous nuclear ribonucleoprotein H2	ITHACA_RND
HNRNPK	HNRNPK	heterogeneous nuclear ribonucleoprotein K	ITHACA_RND
HNRNPL	HNRNPL	heterogeneous nuclear ribonucleoprotein L	ITHACA
HNRNPR	HNRNPR	heterogeneous nuclear ribonucleoprotein R	ITHACA_RND
HNRNPU	HNRNPU	heterogeneous nuclear ribonucleoprotein U	ITHACA_RND
HOGA1	HOGA1	4-hydroxy-2-oxoglutarate aldolase 1	RND
HOXA1	HOXA1	homeobox A1	ITHACA_RND
HOXB1	HOXB1	homeobox B1	ITHACA
HOXB13	HOXB13	homeobox B13	GENTURIS
HOXD10	HOXD10	homeobox D10	NMD
HPCA	HPCA	hippocalcin	RND
HPD	HPD	4-hydroxyphenylpyruvate dioxygenase	ITHACA_RND

HPDL	HPDL	4-hydroxyphenylpyruvate dioxygenase like	ITHACA_RND
HPRT1	HPRT1	hypoxanthine phosphoribosyltransferase 1	ITHACA_RND
HPS1	HPS1	HPS1 biogenesis of lysosomal organelles complex 3 subunit 1	RND
HPS6	HPS6	HPS6 biogenesis of lysosomal organelles complex 2 subunit 3	ITHACA
HRAS	HRAS	HRas proto-oncogene, GTPase	ITHACA_NMD_RND
HS2ST1	HS2ST1	heparan sulfate 2-O-sulfotransferase 1	ITHACA
HS6ST2	HS6ST2	heparan sulfate 6-O-sulfotransferase 2	ITHACA
HSD17B10	HSD17B10	hydroxysteroid 17-beta dehydrogenase 10	ITHACA_RND
HSD17B4	HSD17B4	hydroxysteroid 17-beta dehydrogenase 4	ITHACA_RND
HSD3B7	HSD3B7	hydroxy-delta-5-steroid dehydrogenase, 3 beta- and steroid delta-isomerase 7	RND
HSPA9	HSPA9	heat shock protein family A (Hsp70) member 9	ITHACA
HSPB1	HSPB1	heat shock protein family B (small) member 1	NMD_RND
HSPB3	HSPB3	heat shock protein family B (small) member 3	NMD
HSPB8	HSPB8	heat shock protein family B (small) member 8	NMD_RND
HSPD1	HSPD1	heat shock protein family D (Hsp60) member 1	ITHACA_NMD_RND
HSPG2	HSPG2	heparan sulfate proteoglycan 2	ITHACA_NMD
HTR2A	HTR2A	5-hydroxytryptamine receptor 2A	ITHACA
HTR7	HTR7	5-hydroxytryptamine receptor 7	ITHACA
HTRA1	HTRA1	HtrA serine peptidase 1	RND
HTRA2	HTRA2	HtrA serine peptidase 2	ITHACA_RND
HTT	HTT	huntingtin	ITHACA
HUWE1	HUWE1	HECT, UBA and WWE domain containing E3 ubiquitin protein ligase 1	ITHACA_RND
HYAL1	HYAL1	hyaluronidase 1	RND
HYLS1	HYLS1	HYLS1 centriolar and ciliogenesis associated	ITHACA_RND
IARS	IARS1	isoleucyl-tRNA synthetase 1	ITHACA_RND
IARS2	IARS2	isoleucyl-tRNA synthetase 2, mitochondrial	ITHACA_RND
IBA57	IBA57	iron-sulfur cluster assembly factor IBA57	ITHACA_NMD_RND
ICK	CILK1	ciliogenesis associated kinase 1	RND
IDH2	IDH2	isocitrate dehydrogenase (NADP(+)) 2	ITHACA_RND
IDH3A	IDH3A	isocitrate dehydrogenase (NAD(+)) 3 catalytic subunit alpha	ITHACA
IDS	IDS	iduronate 2-sulfatase	ITHACA_RND
IDUA	IDUA	alpha-L-iduronidase	ITHACA_RND
IER3IP1	IER3IP1	immediate early response 3 interacting protein 1	ITHACA_RND
IFIH1	IFIH1	interferon induced with helicase C domain 1	ITHACA_RND
IFNG	IFNG	interferon gamma	ITHACA
IFRD1	IFRD1	interferon related developmental regulator 1	NMD
IFT140	IFT140	intraflagellar transport 140	ITHACA
IFT172	IFT172	intraflagellar transport 172	ITHACA_RND
IFT27	IFT27	intraflagellar transport 27	ITHACA
IFT43	IFT43	intraflagellar transport 43	ITHACA
IFT52	IFT52	intraflagellar transport 52	ITHACA
IFT57	IFT57	intraflagellar transport 57	ITHACA
IFT74	IFT74	intraflagellar transport 74	ITHACA

IFT81	IFT81	intraflagellar transport 81	ITHACA
IGBP1	IGBP1	immunoglobulin binding protein 1	ITHACA
IGF1	IGF1	insulin like growth factor 1	ITHACA_RND
IGF1R	IGF1R	insulin like growth factor 1 receptor	ITHACA_RND
IGF2	IGF2	insulin like growth factor 2	ITHACA
IGFBP4	IGFBP4	insulin like growth factor binding protein 4	ITHACA
IGHMBP2	IGHMBP2	immunoglobulin mu DNA binding protein 2	NMD_RND
IKBKAP	ELP1	elongator acetyltransferase complex subunit 1	ITHACA_NMD_RND
IKBKG	IKBKG	inhibitor of nuclear factor kappa B kinase regulatory subunit gamma	ITHACA_RND
IL1R2	IL1R2	interleukin 1 receptor type 2	ITHACA
IL1RAPL1	IL1RAPL1	interleukin 1 receptor accessory protein like 1	ITHACA_RND
IL1RAPL2	IL1RAPL2	interleukin 1 receptor accessory protein like 2	ITHACA
IL1RN	IL1RN	interleukin 1 receptor antagonist	ITHACA
IL6ST	IL6ST	interleukin 6 cytokine family signal transducer	ITHACA
ILF2	ILF2	interleukin enhancer binding factor 2	ITHACA
ILF3	ILF3	interleukin enhancer binding factor 3	ITHACA
ILK	ILK	integrin linked kinase	NMD
IMMP2L	IMMP2L	inner mitochondrial membrane peptidase subunit 2	ITHACA
IMPA1	IMPA1	inositol monophosphatase 1	ITHACA
IMPDH2	IMPDH2	inosine monophosphate dehydrogenase 2	ITHACA
INA	INA	internexin neuronal intermediate filament protein alpha	ITHACA
INF2	INF2	inverted formin 2	NMD_RND
INIP	INIP	INTS3 and NABP interacting protein	ITHACA
INO80	INO80	INO80 complex ATPase subunit	ITHACA
INPP4A	INPP4A	inositol polyphosphate-4-phosphatase type I A	ITHACA
INPP5E	INPP5E	inositol polyphosphate-5-phosphatase E	ITHACA_RND
INPP5K	INPP5K	inositol polyphosphate-5-phosphatase K	ITHACA_NMD_RND
INSR	INSR	insulin receptor	ITHACA
INTS1	INTS1	integrator complex subunit 1	ITHACA_RND
INTS8	INTS8	integrator complex subunit 8	ITHACA
INTU	INTU	inturned planar cell polarity protein	ITHACA
IPMK	IPMK	inositol polyphosphate multikinase	GENTURIS
IPP	IPP	intracisternal A particle-promoted polypeptide	ITHACA
IQSEC1	IQSEC1	IQ motif and Sec7 domain ArfGEF 1	ITHACA
IQSEC2	IQSEC2	IQ motif and Sec7 domain ArfGEF 2	ITHACA_RND
IQSEC3	IQSEC3	IQ motif and Sec7 domain ArfGEF 3	ITHACA
IREB2	IREB2	iron responsive element binding protein 2	ITHACA
IRF2BPL	IRF2BPL	interferon regulatory factor 2 binding protein like	ITHACA_RND
IRX5	IRX5	iroquois homeobox 5	ITHACA_RND
ISCA1	ISCA1	iron-sulfur cluster assembly 1	ITHACA_RND
ISCA2	ISCA2	iron-sulfur cluster assembly 2	ITHACA_RND
ISCU	ISCU	iron-sulfur cluster assembly enzyme	NMD_RND
ISLR2	ISLR2	immunoglobulin superfamily containing leucine rich repeat 2	ITHACA
ISPD	CRPPA	CDP-L-ribitol pyrophosphorylase A	ITHACA_NMD_RND

ITCH	ITCH	itchy E3 ubiquitin protein ligase	ITHACA
ITFG2	ITFG2	integrin alpha FG-GAP repeat containing 2	ITHACA
ITGA11	ITGA11	integrin subunit alpha 11	ITHACA
ITGA7	ITGA7	integrin subunit alpha 7	ITHACA_NMD_RND
ITGAV	ITGAV	integrin subunit alpha V	ITHACA
ITIH6	ITIH6	inter-alpha-trypsin inhibitor heavy chain family member 6	ITHACA
ITM2B	ITM2B	integral membrane protein 2B	RND
ITPA	ITPA	inosine triphosphatase	ITHACA_RND
ITPR1	ITPR1	inositol 1,4,5-trisphosphate receptor type 1	ITHACA_NMD_RND
ITPR3	ITPR3	inositol 1,4,5-trisphosphate receptor type 3	NMD
ITSN1	ITSN1	intersectin 1	ITHACA
IVD	IVD	isovaleryl-CoA dehydrogenase	ITHACA_RND
IYD	IYD	iodotyrosine deiodinase	ITHACA
JAG1	JAG1	jagged canonical Notch ligand 1	ITHACA_NMD
JAM2	JAM2	junctional adhesion molecule 2	ITHACA
JAM3	JAM3	junctional adhesion molecule 3	ITHACA_RND
JARID2	JARID2	jumonji and AT-rich interaction domain containing 2	ITHACA
JKAMP	JKAMP	JNK1/MAPK8 associated membrane protein	ITHACA
JMJD1C	JMJD1C	jumonji domain containing 1C	ITHACA
JPH2	JPH2	junctophilin 2	NMD
JPH3	JPH3	junctophilin 3	ITHACA
JUP	JUP	junction plakoglobin	NMD
KALRN	KALRN	kalirin RhoGEF kinase	ITHACA
KANK1	KANK1	KN motif and ankyrin repeat domains 1	ITHACA
KANSL1	KANSL1	KAT8 regulatory NSL complex subunit 1	ITHACA_RND
KARS	KARS1	lysyl-tRNA synthetase 1	ITHACA_NMD_RND
KAT5	KAT5	lysine acetyltransferase 5	ITHACA
KAT6A	KAT6A	lysine acetyltransferase 6A	ITHACA_RND
KAT6B	KAT6B	lysine acetyltransferase 6B	ITHACA_RND
KAT8	KAT8	lysine acetyltransferase 8	ITHACA
KATNAL2	KATNAL2	katanin catalytic subunit A1 like 2	ITHACA
KATNB1	KATNB1	katanin regulatory subunit B1	ITHACA_RND
KBTBD13	KBTBD13	kelch repeat and BTB domain containing 13	NMD_RND
KCNA1	KCNA1	potassium voltage-gated channel subfamily A member 1	ITHACA_NMD_RND
KCNA2	KCNA2	potassium voltage-gated channel subfamily A member 2	ITHACA_RND
KCNA4	KCNA4	potassium voltage-gated channel subfamily A member 4	ITHACA
KCNA5	KCNA5	potassium voltage-gated channel subfamily A member 5	NMD
KCNB1	KCNB1	potassium voltage-gated channel subfamily B member 1	ITHACA_RND
KCNC1	KCNC1	potassium voltage-gated channel subfamily C member 1	ITHACA_RND

KCNC2	KCNC2	potassium voltage-gated channel subfamily C member 2	ITHACA
KCNC3	KCNC3	potassium voltage-gated channel subfamily C member 3	ITHACA_NMD_RND
KCND3	KCND3	potassium voltage-gated channel subfamily D member 3	ITHACA_NMD_RND
KCNE1	KCNE1	potassium voltage-gated channel subfamily E regulatory subunit 1	NMD
KCNE2	KCNE2	potassium voltage-gated channel subfamily E regulatory subunit 2	NMD
KCNE3	KCNE3	potassium voltage-gated channel subfamily E regulatory subunit 3	NMD
KCNH1	KCNH1	potassium voltage-gated channel subfamily H member 1	ITHACA_RND
KCNH2	KCNH2	potassium voltage-gated channel subfamily H member 2	NMD
KCNJ1	KCNJ1	potassium inwardly rectifying channel subfamily J member 1	ITHACA
KCNJ10	KCNJ10	potassium inwardly rectifying channel subfamily J member 10	ITHACA_RND
KCNJ11	KCNJ11	potassium inwardly rectifying channel subfamily J member 11	ITHACA_RND
KCNJ12	KCNJ12	potassium inwardly rectifying channel subfamily J member 12	NMD
KCNJ2	KCNJ2	potassium inwardly rectifying channel subfamily J member 2	NMD
KCNJ5	KCNJ5	potassium inwardly rectifying channel subfamily J member 5	NMD
KCNJ6	KCNJ6	potassium inwardly rectifying channel subfamily J member 6	ITHACA_RND
KCNJ8	KCNJ8	potassium inwardly rectifying channel subfamily J member 8	ITHACA
KCNK18	KCNK18	potassium two pore domain channel subfamily K member 18	ITHACA
KCNK3	KCNK3	potassium two pore domain channel subfamily K member 3	ITHACA
KCNK4	KCNK4	potassium two pore domain channel subfamily K member 4	ITHACA_RND
KCNK6	KCNK6	potassium two pore domain channel subfamily K member 6	ITHACA
KCNK9	KCNK9	potassium two pore domain channel subfamily K member 9	ITHACA_RND
KCNMA1	KCNMA1	potassium calcium-activated channel subfamily M alpha 1	ITHACA_RND
KCNN2	KCNN2	potassium calcium-activated channel subfamily N member 2	ITHACA
KCNN3	KCNN3	potassium calcium-activated channel subfamily N member 3	ITHACA
KCNQ1	KCNQ1	potassium voltage-gated channel subfamily Q member 1	NMD
KCNQ2	KCNQ2	potassium voltage-gated channel subfamily Q member 2	ITHACA_RND

KCNQ3	KCNQ3	potassium voltage-gated channel subfamily Q member 3	ITHACA_RND
KCNQ5	KCNQ5	potassium voltage-gated channel subfamily Q member 5	ITHACA_RND
KCNS3	KCNS3	potassium voltage-gated channel modifier subfamily S member 3	ITHACA
KCNT1	KCNT1	potassium sodium-activated channel subfamily T member 1	ITHACA_RND
KCNT2	KCNT2	potassium sodium-activated channel subfamily T member 2	ITHACA_RND
KCNV1	KCNV1	potassium voltage-gated channel modifier subfamily V member 1	ITHACA
KCTD17	KCTD17	potassium channel tetramerization domain containing 17	RND
KCTD18	KCTD18	potassium channel tetramerization domain containing 18	ITHACA
KCTD3	KCTD3	potassium channel tetramerization domain containing 3	ITHACA_RND
KCTD7	KCTD7	potassium channel tetramerization domain containing 7	ITHACA_RND
KDELR2	KDELR2	KDEL endoplasmic reticulum protein retention receptor 2	ITHACA
KDM1A	KDM1A	lysine demethylase 1A	ITHACA_RND
KDM2B	KDM2B	lysine demethylase 2B	ITHACA
KDM3B	KDM3B	lysine demethylase 3B	ITHACA
KDM4B	KDM4B	lysine demethylase 4B	ITHACA
KDM4C	KDM4C	lysine demethylase 4C	ITHACA
KDM5A	KDM5A	lysine demethylase 5A	ITHACA
KDM5B	KDM5B	lysine demethylase 5B	ITHACA_RND
KDM5C	KDM5C	lysine demethylase 5C	ITHACA_RND
KDM6A	KDM6A	lysine demethylase 6A	ITHACA_RND
KDM6B	KDM6B	lysine demethylase 6B	ITHACA
KIAA0195	TMEM94	transmembrane protein 94	ITHACA_RND
KIAA0196	WASHC5	WASH complex subunit 5	ITHACA_NMD_RND
KIAA0226	RUBCN	rubicon autophagy regulator	ITHACA_NMD
KIAA0232	KIAA0232	KIAA0232	ITHACA
KIAA0556	KATNIP	katanin interacting protein	ITHACA
KIAA0586	KIAA0586	KIAA0586	ITHACA_RND
KIAA0753	KIAA0753	KIAA0753	ITHACA_RND
KIAA0947	ICE1	interactor of little elongation complex ELL subunit 1	ITHACA
KIAA1033	WASHC4	WASH complex subunit 4	ITHACA
KIAA1109	BLTP1	bridge-like lipid transfer protein family member 1	ITHACA_RND
KIAA1161	MYORG	myogenesis regulating glycosidase (putative)	RND
KIAA1244	ARFGEF3	ARFGEF family member 3	ITHACA
KIAA1279	KIFBP	kinesin family binding protein	ITHACA_RND
KIAA1432	RIC1	RIC1 homolog, RAB6A GEF complex partner 1	ITHACA
KIAA1467	FAM234B	family with sequence similarity 234 member B	ITHACA
KIAA1524	CIP2A	cellular inhibitor of PP2A	ITHACA
KIAA1715	LNPK	lunapark, ER junction formation factor	ITHACA

KIAA2022	NEXMIF	neurite extension and migration factor	ITHACA_RND
KIDINS220	KIDINS220	kinase D interacting substrate 220	ITHACA_NMD_RND
KIF11	KIF11	kinesin family member 11	ITHACA_RND
KIF13A	KIF13A	kinesin family member 13A	ITHACA
KIF14	KIF14	kinesin family member 14	ITHACA_RND
KIF15	KIF15	kinesin family member 15	ITHACA
KIF16B	KIF16B	kinesin family member 16B	ITHACA
KIF1A	KIF1A	kinesin family member 1A	ITHACA_NMD_RND
KIF1B	KIF1B	kinesin family member 1B	GENTURIS_NMD
KIF1C	KIF1C	kinesin family member 1C	NMD_RND
KIF21A	KIF21A	kinesin family member 21A	NMD
KIF21B	KIF21B	kinesin family member 21B	ITHACA
KIF23	KIF23	kinesin family member 23	ITHACA
KIF26A	KIF26A	kinesin family member 26A	ITHACA
KIF26B	KIF26B	kinesin family member 26B	NMD
KIF2A	KIF2A	kinesin family member 2A	ITHACA_RND
KIF3B	KIF3B	kinesin family member 3B	ITHACA
KIF4A	KIF4A	kinesin family member 4A	ITHACA
KIF5A	KIF5A	kinesin family member 5A	ITHACA_NMD_RND
KIF5B	KIF5B	kinesin family member 5B	ITHACA
KIF5C	KIF5C	kinesin family member 5C	ITHACA_RND
KIF7	KIF7	kinesin family member 7	ITHACA_RND
KIRREL3	KIRREL3	kirre like nephrin family adhesion molecule 3	ITHACA
KIT	KIT	KIT proto-oncogene, receptor tyrosine kinase	GENTURIS
KLC1	KLC1	kinesin light chain 1	ITHACA
KLC2	KLC2	kinesin light chain 2	ITHACA_NMD
KLF13	KLF13	KLF transcription factor 13	ITHACA
KLF7	KLF7	KLF transcription factor 7	ITHACA
KLHL15	KLHL15	kelch like family member 15	ITHACA
KLHL24	KLHL24	kelch like family member 24	ITHACA
KLHL33	KLHL33	kelch like family member 33	ITHACA
KLHL40	KLHL40	kelch like family member 40	ITHACA_NMD_RND
KLHL41	KLHL41	kelch like family member 41	ITHACA_NMD_RND
KLHL7	KLHL7	kelch like family member 7	ITHACA_RND
KLHL9	KLHL9	kelch like family member 9	NMD
KLLN	KLLN	killin, p53 regulated DNA replication inhibitor	GENTURIS
KMT2A	KMT2A	lysine methyltransferase 2A	ITHACA_RND
KMT2B	KMT2B	lysine methyltransferase 2B	ITHACA_RND
KMT2C	KMT2C	lysine methyltransferase 2C	ITHACA_RND
KMT2D	KMT2D	lysine methyltransferase 2D	ITHACA_RND
KMT2E	KMT2E	lysine methyltransferase 2E (inactive)	ITHACA_RND
KPNA7	KPNA7	karyopherin subunit alpha 7	ITHACA
KPTN	KPTN	kaptin, actin binding protein	ITHACA_RND
KRAS	KRAS	KRAS proto-oncogene, GTPase	GENTURIS_ITHACA_RND

KRBOX4	KRBOX4	KRAB box domain containing 4	ITHACA
KY	KY	kyphoscoliosis peptidase	ITHACA_NMD
KYNU	KYNU	kynureninase	ITHACA_RND
L1CAM	L1CAM	L1 cell adhesion molecule	ITHACA_NMD_RND
L2HGDH	L2HGDH	L-2-hydroxyglutarate dehydrogenase	ITHACA_RND
LAGE3	LAGE3	L antigen family member 3	ITHACA
LAMA1	LAMA1	laminin subunit alpha 1	ITHACA_RND
LAMA2	LAMA2	laminin subunit alpha 2	ITHACA_NMD_RND
LAMA4	LAMA4	laminin subunit alpha 4	NMD
LAMA5	LAMA5	laminin subunit alpha 5	ITHACA_NMD
LAMB1	LAMB1	laminin subunit beta 1	ITHACA_RND
LAMB2	LAMB2	laminin subunit beta 2	ITHACA_NMD
LAMC1	LAMC1	laminin subunit gamma 1	ITHACA
LAMC3	LAMC3	laminin subunit gamma 3	ITHACA_RND
LAMP2	LAMP2	lysosomal associated membrane protein 2	ITHACA_NMD_RND
LARGE	LARGE1	LARGE xylosyl- and glucuronyltransferase 1	ITHACA_NMD_RND
LARP4B	LARP4B	La ribonucleoprotein 4B	ITHACA
LARP7	LARP7	La ribonucleoprotein 7, transcriptional regulator	ITHACA_RND
LARS	LARS1	leucyl-tRNA synthetase 1	ITHACA_RND
LARS2	LARS2	leucyl-tRNA synthetase 2, mitochondrial	ITHACA_RND
LAS1L	LAS1L	LAS1 like ribosome biogenesis factor	ITHACA
LBR	LBR	lamin B receptor	ITHACA_RND
LCAT	LCAT	lecithin-cholesterol acyltransferase	RND
LCT	LCT	lactase	RND
LDB3	LDB3	LIM domain binding 3	ITHACA_NMD_RND
LDHA	LDHA	lactate dehydrogenase A	NMD_RND
LDHD	LDHD	lactate dehydrogenase D	ITHACA
LDLR	LDLR	low density lipoprotein receptor	RND
LDLRAP1	LDLRAP1	low density lipoprotein receptor adaptor protein 1	RND
LEMD2	LEMD2	LEM domain nuclear envelope protein 2	ITHACA
LENG8	LENG8	leukocyte receptor cluster member 8	ITHACA
LEO1	LEO1	LEO1 homolog, Paf1/RNA polymerase II complex component	ITHACA
LEPRE1	P3H1	prolyl 3-hydroxylase 1	ITHACA
LETM1	LETM1	leucine zipper and EF-hand containing transmembrane protein 1	ITHACA
LGI1	LGI1	leucine rich glioma inactivated 1	RND
LGI3	LGI3	leucine rich repeat LGI family member 3	ITHACA
LGI4	LGI4	leucine rich repeat LGI family member 4	ITHACA_RND
LHX3	LHX3	LIM homeobox 3	ITHACA
LIAS	LIAS	lipoic acid synthetase	ITHACA_RND
LIG4	LIG4	DNA ligase 4	GENTURIS_ITHACA_RND
LIMS2	LIMS2	LIM zinc finger domain containing 2	NMD
LINGO1	LINGO1	leucine rich repeat and Ig domain containing 1	ITHACA

LINGO4	LINGO4	leucine rich repeat and Ig domain containing 4	ITHACA
LINS	LINS1	lines homolog 1	ITHACA_RND
LIPA	LIPA	lipase A, lysosomal acid type	RND
LIPT1	LIPT1	lipoyltransferase 1	ITHACA_RND
LIPT2	LIPT2	lipoyl(octanoyl) transferase 2	ITHACA_RND
LITAF	LITAF	lipopolysaccharide induced TNF factor	NMD_RND
LMAN2L	LMAN2L	lectin, mannose binding 2 like	ITHACA
LMBRD1	LMBRD1	LMBR1 domain containing 1	ITHACA_RND
LMBRD2	LMBRD2	LMBR1 domain containing 2	ITHACA
LMNA	LMNA	lamin A/C	ITHACA_NMD_RND
LMNB1	LMNB1	lamin B1	ITHACA_RND
LMNB2	LMNB2	lamin B2	ITHACA
LMOD3	LMOD3	leiomodin 3	NMD_RND
LMTK3	LMTK3	lemur tyrosine kinase 3	ITHACA
LONP1	LONP1	lon peptidase 1, mitochondrial	ITHACA_RND
LPHN1	ADGRL1	adhesion G protein-coupled receptor L1	ITHACA
LPHN2	ADGRL2	adhesion G protein-coupled receptor L2	ITHACA
LPIN1	LPIN1	lipin 1	NMD_RND
LPIN3	LPIN3	lipin 3	ITHACA
LPL	LPL	lipoprotein lipase	RND
LPPR4	PLPPR4	phospholipid phosphatase related 4	ITHACA
LRAT	LRAT	lecithin retinol acyltransferase	ITHACA
LRCH3	LRCH3	leucine rich repeats and calponin homology domain containing 3	ITHACA
LRFN5	LRFN5	leucine rich repeat and fibronectin type III domain containing 5	ITHACA
LRP1	LRP1	LDL receptor related protein 1	ITHACA
LRP12	LRP12	LDL receptor related protein 12	NMD
LRP1B	LRP1B	LDL receptor related protein 1B	ITHACA
LRP2	LRP2	LDL receptor related protein 2	ITHACA_RND
LRP4	LRP4	LDL receptor related protein 4	NMD_RND
LRP5	LRP5	LDL receptor related protein 5	ITHACA
LRPPRC	LRPPRC	leucine rich pentatricopeptide repeat containing	ITHACA_RND
LRRC32	LRRC32	leucine rich repeat containing 32	ITHACA
LRRC40	LRRC40	leucine rich repeat containing 40	ITHACA
LRRC7	LRRC7	leucine rich repeat containing 7	ITHACA
LRRIQ3	LRRIQ3	leucine rich repeats and IQ motif containing 3	ITHACA
LRRK1	LRRK1	leucine rich repeat kinase 1	ITHACA
LRRK2	LRRK2	leucine rich repeat kinase 2	RND
LRSAM1	LRSAM1	leucine rich repeat and sterile alpha motif containing 1	NMD_RND
LSM1	LSM1	LSM1 homolog, mRNA degradation associated	ITHACA
LSS	LSS	lanosterol synthase	ITHACA
LTC4S	LTC4S	leukotriene C4 synthase	ITHACA
LTN1	LTN1	listerin E3 ubiquitin protein ligase 1	ITHACA
LYRM4	LYRM4	LYR motif containing 4	ITHACA
LYRM7	LYRM7	LYR motif containing 7	ITHACA_RND
LYST	LYST	lysosomal trafficking regulator	ITHACA_RND
LZTFL1	LZTFL1	leucine zipper transcription factor like 1	ITHACA

LZTR1	LZTR1	leucine zipper like transcription regulator 1	GENTURIS_ITHACA_RND
MAB21L1	MAB21L1	mab-21 like 1	ITHACA_RND
MAB21L2	MAB21L2	mab-21 like 2	ITHACA_RND
MACF1	MACF1	microtubule actin crosslinking factor 1	ITHACA_RND
MACROD2	MACROD2	mono-ADP ribosylhydrolase 2	ITHACA
MADD	MADD	MAP kinase activating death domain	ITHACA_RND
MAF	MAF	MAF bZIP transcription factor	ITHACA_RND
MAG	MAG	myelin associated glycoprotein	ITHACA_NMD_RND
MAGEA11	MAGEA11	MAGE family member A11	ITHACA
MAGED2	MAGED2	MAGE family member D2	ITHACA
MAGEL2	MAGEL2	MAGE family member L2	ITHACA_RND
MAGI1	MAGI1	membrane associated guanylate kinase, WW and PDZ domain containing 1	ITHACA
MAGI2	MAGI2	membrane associated guanylate kinase, WW and PDZ domain containing 2	ITHACA_RND
MAGT1	MAGT1	magnesium transporter 1	ITHACA_RND
MAMDC2	MAMDC2	MAM domain containing 2	ITHACA
MAN1B1	MAN1B1	mannosidase alpha class 1B member 1	ITHACA_RND
MAN2B1	MAN2B1	mannosidase alpha class 2B member 1	ITHACA_RND
MAN2B2	MAN2B2	mannosidase alpha class 2B member 2	ITHACA
MANBA	MANBA	mannosidase beta	ITHACA_RND
MAOA	MAOA	monoamine oxidase A	ITHACA_RND
MAP1B	MAP1B	microtubule associated protein 1B	ITHACA
MAP2	MAP2	microtubule associated protein 2	ITHACA
MAP2K1	MAP2K1	mitogen-activated protein kinase kinase 1	GENTURIS_ITHACA_RND
MAP2K2	MAP2K2	mitogen-activated protein kinase kinase 2	GENTURIS_ITHACA_RND
MAP3K7	MAP3K7	mitogen-activated protein kinase kinase kinase 7	ITHACA
MAP4K4	MAP4K4	mitogen-activated protein kinase kinase kinase kinase 4	ITHACA
MAPK1	MAPK1	mitogen-activated protein kinase 1	ITHACA
MAPK10	MAPK10	mitogen-activated protein kinase 10	ITHACA
MAPK3	MAPK3	mitogen-activated protein kinase 3	ITHACA
MAPK8	MAPK8	mitogen-activated protein kinase 8	ITHACA
MAPK8IP3	MAPK8IP3	mitogen-activated protein kinase 8 interacting protein 3	ITHACA_RND
MAPKAPK5	MAPKAPK5	MAPK activated protein kinase 5	ITHACA
MAPRE2	MAPRE2	microtubule associated protein RP/EB family member 2	ITHACA
MAPT	MAPT	microtubule associated protein tau	NMD_RND
MARS	MARS1	methionyl-tRNA synthetase 1	NMD_RND
MARS2	MARS2	methionyl-tRNA synthetase 2, mitochondrial	ITHACA_NMD_RND
MASP1	MASP1	MBL associated serine protease 1	ITHACA_RND
MAST1	MAST1	microtubule associated serine/threonine kinase 1	ITHACA_RND
MAT1A	MAT1A	methionine adenosyltransferase 1A	ITHACA_RND
MATN4	MATN4	matrilin 4	ITHACA
MATR3	MATR3	matrin 3	NMD_RND

MAX	MAX	MYC associated factor X	GENTURIS
MB	MB	myoglobin	NMD
MBD4	MBD4	methyl-CpG binding domain 4, DNA glycosylase	GENTURIS
MBD5	MBD5	methyl-CpG binding domain protein 5	ITHACA_RND
MBNL3	MBNL3	muscleblind like splicing regulator 3	ITHACA
MBOAT7	MBOAT7	membrane bound O-acyltransferase domain containing 7	ITHACA_RND
MBTPS2	MBTPS2	membrane bound transcription factor peptidase, site 2	ITHACA_RND
MCCC1	MCCC1	methylcrotonyl-CoA carboxylase subunit 1	ITHACA_RND
MCCC2	MCCC2	methylcrotonyl-CoA carboxylase subunit 2	ITHACA_RND
MCEE	MCEE	methylmalonyl-CoA epimerase	ITHACA_RND
MCM3AP	MCM3AP	minichromosome maintenance complex component 3 associated protein	ITHACA_NMD_RND
MCM4	MCM4	minichromosome maintenance complex component 4	ITHACA
MCOLN1	MCOLN1	mucolipin TRP cation channel 1	ITHACA_RND
MCPH1	MCPH1	microcephalin 1	ITHACA_RND
MCTP2	MCTP2	multiple C2 and transmembrane domain containing 2	ITHACA
MDGA2	MDGA2	MAM domain containing glycosylphosphatidylinositol anchor 2	ITHACA
MDH1	MDH1	malate dehydrogenase 1	ITHACA
MDH2	MDH2	malate dehydrogenase 2	GENTURIS_ITHACA_RND
MDM2	MDM2	MDM2 proto-oncogene	ITHACA
MECOM	MECOM	MDS1 and EVI1 complex locus	ITHACA
MECP2	MECP2	methyl-CpG binding protein 2	ITHACA_RND
MECR	MECR	mitochondrial trans-2-enoyl-CoA reductase	ITHACA_RND
MED12	MED12	mediator complex subunit 12	ITHACA_RND
MED12L	MED12L	mediator complex subunit 12L	ITHACA
MED13	MED13	mediator complex subunit 13	ITHACA
MED13L	MED13L	mediator complex subunit 13L	ITHACA_RND
MED17	MED17	mediator complex subunit 17	ITHACA_RND
MED23	MED23	mediator complex subunit 23	ITHACA_RND
MED25	MED25	mediator complex subunit 25	ITHACA_NMD_RND
MED27	MED27	mediator complex subunit 27	ITHACA
MEF2C	MEF2C	myocyte enhancer factor 2C	ITHACA_RND
MEF2D	MEF2D	myocyte enhancer factor 2D	ITHACA
MEGF10	MEGF10	multiple EGF like domains 10	ITHACA_NMD_RND
MEGF8	MEGF8	multiple EGF like domains 8	ITHACA
MEGF9	MEGF9	multiple EGF like domains 9	ITHACA
MEIS2	MEIS2	Meis homeobox 2	ITHACA_RND
MELK	MELK	maternal embryonic leucine zipper kinase	ITHACA
MEN1	MEN1	menin 1	GENTURIS
MEPCE	MEPCE	methylphosphate capping enzyme	ITHACA
MESDC2	MESD	mesoderm development LRP chaperone	ITHACA
MET	MET	MET proto-oncogene, receptor tyrosine kinase	GENTURIS_NMD

METAP1	METAP1	methionyl aminopeptidase 1	ITHACA
METTL23	METTL23	methyltransferase like 23	ITHACA_RND
METTL4	METTL4	methyltransferase 4, N6-adenosine	ITHACA
METTL5	METTL5	methyltransferase 5, N6-adenosine	ITHACA
MFF	MFF	mitochondrial fission factor	ITHACA_RND
MFN2	MFN2	mitofusin 2	ITHACA_NMD_RND
MFRP	MFRP	membrane frizzled-related protein	ITHACA
MFSD11	MFSD11	major facilitator superfamily domain containing 11	ITHACA
MFSD2A	MFSD2A	major facilitator superfamily domain containing 2A	ITHACA
MFSD8	MFSD8	major facilitator superfamily domain containing 8	ITHACA_RND
MGAT2	MGAT2	alpha-1,6-mannosyl-glycoprotein 2-beta-N-acetylglucosaminyltransferase	ITHACA_RND
MGME1	MGME1	mitochondrial genome maintenance exonuclease 1	ITHACA_NMD_RND
MGP	MGP	matrix Gla protein	ITHACA_RND
MIA3	MIA3	MIA SH3 domain ER export factor 3	ITHACA
MIB1	MIB1	MIB E3 ubiquitin protein ligase 1	ITHACA_NMD
MICU1	MICU1	mitochondrial calcium uptake 1	ITHACA_RND
MICU2	MICU2	mitochondrial calcium uptake 2	ITHACA
MID1	MID1	midline 1	ITHACA_RND
MID2	MID2	midline 2	ITHACA
MINPP1	MINPP1	multiple inositol-polyphosphate phosphatase 1	ITHACA
MIPEP	MIPEP	mitochondrial intermediate peptidase	ITHACA_RND
MIR17HG	MIR17HG	miR-17-92a-1 cluster host gene	ITHACA
MITF	MITF	melanocyte inducing transcription factor	GENTURIS
MKKS	MKKS	MKKS centrosomal shuttling protein	ITHACA_RND
MKL2	MRTFB	myocardin related transcription factor B	ITHACA
MKRN3	MKRN3	makorin ring finger protein 3	ITHACA
MKS1	MKS1	MKS transition zone complex subunit 1	ITHACA_RND
MLC1	MLC1	modulator of VRAC current 1	ITHACA_RND
MLH1	MLH1	mutL homolog 1	GENTURIS
MLH3	MLH3	mutL homolog 3	GENTURIS
MLLT1	MLLT1	MLLT1 super elongation complex subunit	ITHACA
MLTK	MAP3K20	mitogen-activated protein kinase kinase kinase 20	ITHACA_NMD
MLXIPL	MLXIPL	MLX interacting protein like	ITHACA
MLYCD	MLYCD	malonyl-CoA decarboxylase	ITHACA_RND
MMAA	MMAA	metabolism of cobalamin associated A	ITHACA_RND
MMAB	MMAB	metabolism of cobalamin associated B	ITHACA_RND
MMACHC	MMACHC	metabolism of cobalamin associated C	ITHACA_RND
MMADHC	MMADHC	metabolism of cobalamin associated D	ITHACA_RND
MME	MME	membrane metalloendopeptidase	NMD_RND
MMGT1	MMGT1	membrane magnesium transporter 1	ITHACA
MMP13	MMP13	matrix metallopeptidase 13	ITHACA
MMP21	MMP21	matrix metallopeptidase 21	ITHACA
MN1	MN1	MN1 proto-oncogene, transcriptional regulator	ITHACA_RND
MNX1	MNX1	motor neuron and pancreas homeobox 1	ITHACA
MOCS1	MOCS1	molybdenum cofactor synthesis 1	ITHACA_RND
MOCS2	MOCS2	molybdenum cofactor synthesis 2	ITHACA_RND

MOGS	MOGS	mannosyl-oligosaccharide glucosidase	ITHACA_RND
MORC2	MORC2	MORC family CW-type zinc finger 2	ITHACA_NMD_RND
MPC1	MPC1	mitochondrial pyruvate carrier 1	ITHACA_RND
MPDU1	MPDU1	mannose-P-dolichol utilization defect 1	ITHACA_NMD_RND
MPDZ	MPDZ	multiple PDZ domain crumbs cell polarity complex component	ITHACA
MPHOSPH8	MPHOSPH8	M-phase phosphoprotein 8	ITHACA
MPI	MPI	mannose phosphate isomerase	ITHACA_RND
MPL	MPL	MPL proto-oncogene, thrombopoietin receptor	GENTURIS
MPLKIP	MPLKIP	M-phase specific PLK1 interacting protein	ITHACA_RND
MPP5	PALS1	protein associated with LIN7 1, MAGUK p55 family member	ITHACA
MPV17	MPV17	mitochondrial inner membrane protein MPV17	ITHACA_NMD_RND
MPZ	MPZ	myelin protein zero	ITHACA_NMD_RND
MRAS	MRAS	muscle RAS oncogene homolog	ITHACA
MRE11A	MRE11	MRE11 homolog, double strand break repair nuclease	GENTURIS_ITHACA_NMD_RND
MROH5	MROH5	maestro heat like repeat family member 5 (gene/pseudogene)	ITHACA
MRPL10	MRPL10	mitochondrial ribosomal protein L10	ITHACA
MRPL12	MRPL12	mitochondrial ribosomal protein L12	ITHACA
MRPL24	MRPL24	mitochondrial ribosomal protein L24	ITHACA
MRPL3	MRPL3	mitochondrial ribosomal protein L3	ITHACA_NMD_RND
MRPL44	MRPL44	mitochondrial ribosomal protein L44	NMD_RND
MRPS14	MRPS14	mitochondrial ribosomal protein S14	ITHACA
MRPS16	MRPS16	mitochondrial ribosomal protein S16	ITHACA
MRPS2	MRPS2	mitochondrial ribosomal protein S2	ITHACA_RND
MRPS22	MRPS22	mitochondrial ribosomal protein S22	ITHACA_RND
MRPS25	MRPS25	mitochondrial ribosomal protein S25	ITHACA_NMD
MRPS34	MRPS34	mitochondrial ribosomal protein S34	ITHACA_RND
MRPS35	MRPS35	mitochondrial ribosomal protein S35	ITHACA
MRPS7	MRPS7	mitochondrial ribosomal protein S7	ITHACA
MSH2	MSH2	mutS homolog 2	GENTURIS
MSH3	MSH3	mutS homolog 3	GENTURIS
MSH6	MSH6	mutS homolog 6	GENTURIS
MSL2	MSL2	MSL complex subunit 2	ITHACA
MSL3	MSL3	MSL complex subunit 3	ITHACA_RND
MSMO1	MSMO1	methylsterol monooxygenase 1	ITHACA_RND
MSS51	MSS51	MSS51 mitochondrial translational activator	ITHACA
MSTN	MSTN	myostatin	NMD
MSTO1	MSTO1	misato mitochondrial distribution and morphology regulator 1	ITHACA_NMD_RND
MT-ATP6	MT-ATP6	mitochondrially encoded ATP synthase membrane subunit 6	ITHACA_NMD_RND

MT-ATP8	MT-ATP8	mitochondrially encoded ATP synthase membrane subunit 8	RND
MT-CO1	MT-CO1	mitochondrially encoded cytochrome c oxidase I	ITHACA_RND
MT-CO2	MT-CO2	mitochondrially encoded cytochrome c oxidase II	ITHACA_RND
MT-CO3	MT-CO3	mitochondrially encoded cytochrome c oxidase III	ITHACA_RND
MT-CYB	MT-CYB	mitochondrially encoded cytochrome b	RND
MT-ND1	MT-ND1	mitochondrially encoded NADH:ubiquinone oxidoreductase core subunit 1	RND
MT-ND2	MT-ND2	mitochondrially encoded NADH:ubiquinone oxidoreductase core subunit 2	RND
MT-ND3	MT-ND3	mitochondrially encoded NADH:ubiquinone oxidoreductase core subunit 3	NMD_RND
MT-ND4	MT-ND4	mitochondrially encoded NADH:ubiquinone oxidoreductase core subunit 4	RND
MT-ND4L	MT-ND4L	mitochondrially encoded NADH:ubiquinone oxidoreductase core subunit 4L	RND
MT-ND5	MT-ND5	mitochondrially encoded NADH:ubiquinone oxidoreductase core subunit 5	ITHACA_RND
MT-ND6	MT-ND6	mitochondrially encoded NADH:ubiquinone oxidoreductase core subunit 6	RND
MT-RNR1	MT-RNR1	mitochondrially encoded 12S rRNA	RND
MT-TA	MT-TA	mitochondrially encoded tRNA-Ala (GCN)	RND
MT-TC	MT-TC	mitochondrially encoded tRNA-Cys (UGU/C)	RND
MT-TD	MT-TD	mitochondrially encoded tRNA-Asp (GAU/C)	RND
MT-TE	MT-TE	mitochondrially encoded tRNA-Glu (GAA/G)	ITHACA_RND
MT-TF	MT-TF	mitochondrially encoded tRNA-Phe (UUU/C)	ITHACA_RND
MT-TG	MT-TG	mitochondrially encoded tRNA-Gly (GGN)	RND
MT-TH	MT-TH	mitochondrially encoded tRNA-His (CAU/C)	RND
MT-TI	MT-TI	mitochondrially encoded tRNA-Ile (AUU/C)	RND
MT-TK	MT-TK	mitochondrially encoded tRNA-Lys (AAA/G)	ITHACA_RND
MT-TL1	MT-TL1	mitochondrially encoded tRNA-Leu (UUA/G) 1	ITHACA_NMD_RND
MT-TL2	MT-TL2	mitochondrially encoded tRNA-Leu (CUN) 2	RND
MT-TM	MT-TM	mitochondrially encoded tRNA-Met (AUA/G)	NMD_RND
MT-TN	MT-TN	mitochondrially encoded tRNA-Asn (AAU/C)	ITHACA_NMD_RND
MT-TP	MT-TP	mitochondrially encoded tRNA-Pro (CCN)	RND
MT-TQ	MT-TQ	mitochondrially encoded tRNA-Gln (CAA/G)	RND
MT-TR	MT-TR	mitochondrially encoded tRNA-Arg (CGN)	RND
MT-TS1	MT-TS1	mitochondrially encoded tRNA-Ser (UCN) 1	ITHACA_RND
MT-TS2	MT-TS2	mitochondrially encoded tRNA-Ser (AGU/C) 2	RND
MT-TT	MT-TT	mitochondrially encoded tRNA-Thr (ACN)	NMD
MT-TV	MT-TV	mitochondrially encoded tRNA-Val (GUN)	ITHACA_RND
MT-TW	MT-TW	mitochondrially encoded tRNA-Trp (UGA/G)	RND
MT-TY	MT-TY	mitochondrially encoded tRNA-Tyr (UAU/C)	RND
MTDH	MTDH	metadherin	ITHACA
MTF1	MTF1	metal regulatory transcription factor 1	ITHACA
MTF2	MTF2	metal response element binding transcription factor 2	ITHACA
MTFMT	MTFMT	mitochondrial methionyl-tRNA formyltransferase	ITHACA_RND

MTHFD1	MTHFD1	methylenetetrahydrofolate dehydrogenase, cyclohydrolase and formyltetrahydrofolate synthetase 1	ITHACA
MTHFR	MTHFR	methylenetetrahydrofolate reductase	ITHACA_RND
MTHFS	MTHFS	methenyltetrahydrofolate synthetase	ITHACA
MTIF2	MTIF2	mitochondrial translational initiation factor 2	ITHACA
MTM1	MTM1	myotubularin 1	NMD_RND
MTMR14	MTMR14	myotubularin related protein 14	ITHACA
MTMR2	MTMR2	myotubularin related protein 2	ITHACA_NMD_RND
MTMR9	MTMR9	myotubularin related protein 9	ITHACA
MTO1	MTO1	mitochondrial tRNA translation optimization 1	ITHACA_NMD_RND
MTOR	MTOR	mechanistic target of rapamycin kinase	ITHACA_RND
MTPAP	MTPAP	mitochondrial poly(A) polymerase	ITHACA_NMD_RND
MTR	MTR	5-methyltetrahydrofolate-homocysteine methyltransferase	ITHACA_RND
MTRR	MTRR	5-methyltetrahydrofolate-homocysteine methyltransferase reductase	ITHACA_RND
MTSS1L	MTSS2	MTSS I-BAR domain containing 2	ITHACA
MTTP	MTTP	microsomal triglyceride transfer protein	ITHACA_RND
MUC3A	MUC3A	mucin 3A, cell surface associated	ITHACA
MUC5B	MUC5B	mucin 5B, oligomeric mucus/gel-forming	GENTURIS
MURC	CAVIN4	caveolae associated protein 4	NMD
MUSK	MUSK	muscle associated receptor tyrosine kinase	NMD_RND
MUT	MMUT	methylmalonyl-CoA mutase	ITHACA_RND
MUTYH	MUTYH	mutY DNA glycosylase	GENTURIS
MVK	MVK	mevalonate kinase	ITHACA_RND
MXRA8	MXRA8	matrix remodeling associated 8	ITHACA
MYBPC1	MYBPC1	myosin binding protein C1	NMD_RND
MYBPC3	MYBPC3	myosin binding protein C3	NMD
MYCN	MYCN	MYCN proto-oncogene, bHLH transcription factor	ITHACA_RND
MYH10	MYH10	myosin heavy chain 10	ITHACA
MYH13	MYH13	myosin heavy chain 13	ITHACA
MYH14	MYH14	myosin heavy chain 14	NMD
MYH2	MYH2	myosin heavy chain 2	NMD_RND
MYH3	MYH3	myosin heavy chain 3	ITHACA_NMD_RND
MYH6	MYH6	myosin heavy chain 6	NMD
MYH7	MYH7	myosin heavy chain 7	NMD_RND
MYH8	MYH8	myosin heavy chain 8	NMD_RND
MYH9	MYH9	myosin heavy chain 9	ITHACA
MYL1	MYL1	myosin light chain 1	ITHACA_NMD_RND
MYL2	MYL2	myosin light chain 2	NMD
MYL3	MYL3	myosin light chain 3	NMD
MYL4	MYL4	myosin light chain 4	NMD
MYLK2	MYLK2	myosin light chain kinase 2	NMD
MYO18B	MYO18B	myosin XVIIIB	ITHACA_NMD_RND

MYO1A	MYO1A	myosin IA	ITHACA
MYO1E	MYO1E	myosin IE	ITHACA
MYO1H	MYO1H	myosin IH	ITHACA
MYO5A	MYO5A	myosin VA	ITHACA_RND
MYO7A	MYO7A	myosin VIIA	ITHACA
MYO9A	MYO9A	myosin IXA	ITHACA_NMD_RND
MYOCD	MYOCD	myocardin	ITHACA
MYOD1	MYOD1	myogenic differentiation 1	ITHACA
MYOT	MYOT	myotilin	NMD_RND
MYOZ2	MYOZ2	myozenin 2	NMD
MYPN	MYPN	myopalladin	NMD_RND
MYRF	MYRF	myelin regulatory factor	ITHACA
MYSM1	MYSM1	Myb like, SWIRM and MPN domains 1	ITHACA
MYT1L	MYT1L	myelin transcription factor 1 like	ITHACA_RND
NAA10	NAA10	N-alpha-acetyltransferase 10, NatA catalytic subunit	ITHACA_RND
NAA15	NAA15	N-alpha-acetyltransferase 15, NatA auxiliary subunit	ITHACA_RND
NAA35	NAA35	N-alpha-acetyltransferase 35, NatC auxiliary subunit	ITHACA
NACC1	NACC1	nucleus accumbens associated 1	ITHACA_RND
NADK2	NADK2	NAD kinase 2, mitochondrial	ITHACA_RND
NADSYN1	NADSYN1	NAD synthetase 1	ITHACA
NAGA	NAGA	alpha-N-acetylgalactosaminidase	ITHACA_RND
NAGLU	NAGLU	N-acetyl-alpha-glucosaminidase	ITHACA_NMD_RND
NAGS	NAGS	N-acetylglutamate synthase	ITHACA_RND
NALCN	NALCN	sodium leak channel, non-selective	ITHACA_RND
NANS	NANS	N-acetylneuraminate synthase	ITHACA_RND
NAPB	NAPB	NSF attachment protein beta	ITHACA
NAPRT1	NAPRT	nicotinate phosphoribosyltransferase	ITHACA
NARG2	ICE2	interactor of little elongation complex ELL subunit 2	ITHACA
NARS	NARS1	asparaginyl-tRNA synthetase 1	ITHACA
NARS2	NARS2	asparaginyl-tRNA synthetase 2, mitochondrial	ITHACA_NMD_RND
NAT10	NAT10	N-acetyltransferase 10	ITHACA
NAT8L	NAT8L	N-acetyltransferase 8 like	ITHACA
NBAS	NBAS	NBAS subunit of NRZ tethering complex	ITHACA
NBEA	NBEA	neurobeachin	ITHACA_RND
NBN	NBN	nibrin	GENTURIS_ITHACA
NCAPD2	NCAPD2	non-SMC condensin I complex subunit D2	ITHACA
NCAPD3	NCAPD3	non-SMC condensin II complex subunit D3	ITHACA
NCAPG2	NCAPG2	non-SMC condensin II complex subunit G2	ITHACA
NCAPH	NCAPH	non-SMC condensin I complex subunit H	ITHACA
NCDN	NCDN	neurochondrin	ITHACA
NCKAP1	NCKAP1	NCK associated protein 1	ITHACA
NCKAP5	NCKAP5	NCK associated protein 5	ITHACA
NCOA1	NCOA1	nuclear receptor coactivator 1	ITHACA

NCOR1	NCOR1	nuclear receptor corepressor 1	ITHACA
NCOR2	NCOR2	nuclear receptor corepressor 2	ITHACA
NDE1	NDE1	nudE neurodevelopment protein 1	ITHACA_RND
NDN	NDN	necdin, MAGE family member	ITHACA
NDNL2	NSMCE3	NSE3 homolog, SMC5-SMC6 complex component	ITHACA
NDP	NDP	norrin cystine knot growth factor NDP	ITHACA_RND
NDRG1	NDRG1	N-myc downstream regulated 1	NMD_RND
NDST1	NDST1	N-deacetylase and N-sulfotransferase 1	ITHACA_RND
NDUFA1	NDUFA1	NADH:ubiquinone oxidoreductase subunit A1	ITHACA_RND
NDUFA10	NDUFA10	NADH:ubiquinone oxidoreductase subunit A10	ITHACA_RND
NDUFA11	NDUFA11	NADH:ubiquinone oxidoreductase subunit A11	ITHACA_RND
NDUFA12	NDUFA12	NADH:ubiquinone oxidoreductase subunit A12	ITHACA
NDUFA13	NDUFA13	NADH:ubiquinone oxidoreductase subunit A13	ITHACA
NDUFA2	NDUFA2	NADH:ubiquinone oxidoreductase subunit A2	ITHACA_RND
NDUFA4	NDUFA4	NDUFA4 mitochondrial complex associated	ITHACA_RND
NDUFA6	NDUFA6	NADH:ubiquinone oxidoreductase subunit A6	ITHACA_RND
NDUFA8	NDUFA8	NADH:ubiquinone oxidoreductase subunit A8	ITHACA
NDUFA9	NDUFA9	NADH:ubiquinone oxidoreductase subunit A9	ITHACA_RND
NDUFAF1	NDUFAF1	NADH:ubiquinone oxidoreductase complex assembly factor 1	ITHACA_NMD_RND
NDUFAF2	NDUFAF2	NADH:ubiquinone oxidoreductase complex assembly factor 2	ITHACA_RND
NDUFAF3	NDUFAF3	NADH:ubiquinone oxidoreductase complex assembly factor 3	ITHACA_RND
NDUFAF4	NDUFAF4	NADH:ubiquinone oxidoreductase complex assembly factor 4	ITHACA_RND
NDUFAF5	NDUFAF5	NADH:ubiquinone oxidoreductase complex assembly factor 5	ITHACA_RND
NDUFAF6	NDUFAF6	NADH:ubiquinone oxidoreductase complex assembly factor 6	ITHACA_RND
NDUFB10	NDUFB10	NADH:ubiquinone oxidoreductase subunit B10	ITHACA
NDUFB11	NDUFB11	NADH:ubiquinone oxidoreductase subunit B11	ITHACA_RND
NDUFB3	NDUFB3	NADH:ubiquinone oxidoreductase subunit B3	ITHACA_RND
NDUFB8	NDUFB8	NADH:ubiquinone oxidoreductase subunit B8	ITHACA_RND
NDUFB9	NDUFB9	NADH:ubiquinone oxidoreductase subunit B9	ITHACA
NDUFC2	NDUFC2	NADH:ubiquinone oxidoreductase subunit C2	ITHACA
NDUFS1	NDUFS1	NADH:ubiquinone oxidoreductase core subunit S1	ITHACA_RND
NDUFS2	NDUFS2	NADH:ubiquinone oxidoreductase core subunit S2	ITHACA_RND
NDUFS3	NDUFS3	NADH:ubiquinone oxidoreductase core subunit S3	ITHACA_RND
NDUFS4	NDUFS4	NADH:ubiquinone oxidoreductase subunit S4	ITHACA_RND
NDUFS6	NDUFS6	NADH:ubiquinone oxidoreductase subunit S6	ITHACA_RND
NDUFS7	NDUFS7	NADH:ubiquinone oxidoreductase core subunit S7	ITHACA_RND
NDUFS8	NDUFS8	NADH:ubiquinone oxidoreductase core subunit S8	ITHACA_RND
NDUFV1	NDUFV1	NADH:ubiquinone oxidoreductase core subunit V1	ITHACA_RND
NDUFV2	NDUFV2	NADH:ubiquinone oxidoreductase core subunit V2	ITHACA_RND
NEB	NEB	nebulin	ITHACA_NMD_RND
NECAP1	NECAP1	NECAP endocytosis associated 1	ITHACA
NECAP2	NECAP2	NECAP endocytosis associated 2	ITHACA

NEDD4L	NEDD4L	NEDD4 like E3 ubiquitin protein ligase	ITHACA_RND
NEFH	NEFH	neurofilament heavy chain	NMD_RND
NEFL	NEFL	neurofilament light chain	ITHACA_NMD_RND
NEK1	NEK1	NIMA related kinase 1	ITHACA_NMD
NEK9	NEK9	NIMA related kinase 9	NMD
NEMF	NEMF	nuclear export mediator factor	ITHACA
NEO1	NEO1	neogenin 1	ITHACA
NEU1	NEU1	neuraminidase 1	ITHACA_RND
NEURL4	NEURL4	neuralized E3 ubiquitin protein ligase 4	ITHACA
NEUROD2	NEUROD2	neuronal differentiation 2	ITHACA
NEUROG1	NEUROG1	neurogenin 1	ITHACA
NEXN	NEXN	nexilin F-actin binding protein	NMD
NF1	NF1	neurofibromin 1	GENTURIS_ITHACA_RND
NF2	NF2	NF2, moesin-ezrin-radixin like (MERLIN) tumor suppressor	GENTURIS
NFASC	NFASC	neurofascin	ITHACA_RND
NFE2L2	NFE2L2	NFE2 like bZIP transcription factor 2	ITHACA
NFE2L3	NFE2L3	NFE2 like bZIP transcription factor 3	ITHACA
NFIA	NFIA	nuclear factor I A	ITHACA_RND
NFIB	NFIB	nuclear factor I B	ITHACA
NFIX	NFIX	nuclear factor I X	ITHACA_RND
NFKB2	NFKB2	nuclear factor kappa B subunit 2	ITHACA
NFU1	NFU1	NFU1 iron-sulfur cluster scaffold	ITHACA_RND
NFXL1	NFXL1	nuclear transcription factor, X-box binding like 1	ITHACA
NGF	NGF	nerve growth factor	ITHACA_NMD_RND
NGFR	NGFR	nerve growth factor receptor	ITHACA
NGLY1	NGLY1	N-glycanase 1	ITHACA_RND
NHLRC1	NHLRC1	NHL repeat containing E3 ubiquitin protein ligase 1	RND
NHLRC2	NHLRC2	NHL repeat containing 2	ITHACA
NHP2	NHP2	NHP2 ribonucleoprotein	GENTURIS_ITHACA
NHS	NHS	NHS actin remodeling regulator	ITHACA_RND
NID1	NID1	nidogen 1	ITHACA
NIN	NIN	ninein	ITHACA
NIPA1	NIPA1	NIPA magnesium transporter 1	NMD_RND
NIPBL	NIPBL	NIPBL cohesin loading factor	ITHACA_RND
NKAP	NKAP	NFKB activating protein	ITHACA_RND
NKX2-1	NKX2-1	NK2 homeobox 1	ITHACA_RND
NKX2-5	NKX2-5	NK2 homeobox 5	ITHACA
NKX6-2	NKX6-2	NK6 homeobox 2	ITHACA_NMD_RND
NLGN1	NLGN1	neuroligin 1	ITHACA
NLGN2	NLGN2	neuroligin 2	ITHACA
NLGN3	NLGN3	neuroligin 3	ITHACA_RND
NLGN4X	NLGN4X	neuroligin 4 X-linked	ITHACA
NLGN4Y	NLGN4Y	neuroligin 4 Y-linked	ITHACA
NLRP3	NLRP3	NLR family pyrin domain containing 3	ITHACA

NLRP4	NLRP4	NLR family pyrin domain containing 4	ITHACA
NMNAT1	NMNAT1	nicotinamide nucleotide adenylyltransferase 1	ITHACA
NMNAT2	NMNAT2	nicotinamide nucleotide adenylyltransferase 2	ITHACA_NMD
NNT	NNT	nicotinamide nucleotide transhydrogenase	RND
NONO	NONO	non-POU domain containing octamer binding	ITHACA_RND
NOP10	NOP10	NOP10 ribonucleoprotein	GENTURIS_ITHACA
NOP56	NOP56	NOP56 ribonucleoprotein	NMD
NOTCH1	NOTCH1	notch receptor 1	ITHACA
NOTCH3	NOTCH3	notch receptor 3	ITHACA_RND
NOVA2	NOVA2	NOVA alternative splicing regulator 2	ITHACA
NPAP1	NPAP1	nuclear pore associated protein 1	ITHACA
NPAS3	NPAS3	neuronal PAS domain protein 3	ITHACA
NPAS4	NPAS4	neuronal PAS domain protein 4	ITHACA
NPC1	NPC1	NPC intracellular cholesterol transporter 1	ITHACA_RND
NPC2	NPC2	NPC intracellular cholesterol transporter 2	ITHACA_RND
NPHP1	NPHP1	nephrocystin 1	ITHACA_RND
NPHP3	NPHP3	nephrocystin 3	ITHACA_RND
NPHS1	NPHS1	NPHS1 adhesion molecule, nephrin	ITHACA
NPM1	NPM1	nucleophosmin 1	GENTURIS_ITHACA
NPPA	NPPA	natriuretic peptide A	NMD
NPRL2	NPRL2	NPR2 like, GATOR1 complex subunit	ITHACA_RND
NPRL3	NPRL3	NPR3 like, GATOR1 complex subunit	ITHACA_RND
NR1I3	NR1I3	nuclear receptor subfamily 1 group I member 3	ITHACA
NR2F1	NR2F1	nuclear receptor subfamily 2 group F member 1	ITHACA_RND
NR3C2	NR3C2	nuclear receptor subfamily 3 group C member 2	ITHACA
NR4A2	NR4A2	nuclear receptor subfamily 4 group A member 2	ITHACA
NRAS	NRAS	NRAS proto-oncogene, GTPase	GENTURIS_ITHACA_RND
NRG3	NRG3	neuregulin 3	ITHACA
NRROS	NRROS	negative regulator of reactive oxygen species	ITHACA
NRXN1	NRXN1	neurexin 1	ITHACA_RND
NRXN2	NRXN2	neurexin 2	ITHACA
NRXN3	NRXN3	neurexin 3	ITHACA
NSD1	NSD1	nuclear receptor binding SET domain protein 1	GENTURIS_ITHACA_RND
NSDHL	NSDHL	NAD(P) dependent steroid dehydrogenase-like	ITHACA_RND
NSUN2	NSUN2	NOP2/Sun RNA methyltransferase 2	ITHACA_RND
NT5C2	NT5C2	5'-nucleotidase, cytosolic II	ITHACA_NMD_RND
NT5C3A	NT5C3A	5'-nucleotidase, cytosolic IIIA	ITHACA_RND
NTHL1	NTHL1	nth like DNA glycosylase 1	GENTURIS
NTN1	NTN1	netrin 1	ITHACA
NTNG1	NTNG1	netrin G1	ITHACA
NTNG2	NTNG2	netrin G2	ITHACA_RND
NTRK1	NTRK1	neurotrophic receptor tyrosine kinase 1	ITHACA_NMD_RND
NTRK2	NTRK2	neurotrophic receptor tyrosine kinase 2	ITHACA_RND
NUAK1	NUAK1	NUAK family kinase 1	ITHACA

NUBPL	NUBPL	NUBP iron-sulfur cluster assembly factor, mitochondrial	ITHACA_RND
NUDT2	NUDT2	nudix hydrolase 2	ITHACA
NUP107	NUP107	nucleoporin 107	ITHACA
NUP133	NUP133	nucleoporin 133	ITHACA
NUP155	NUP155	nucleoporin 155	NMD
NUP188	NUP188	nucleoporin 188	ITHACA
NUP205	NUP205	nucleoporin 205	ITHACA
NUP214	NUP214	nucleoporin 214	ITHACA
NUP37	NUP37	nucleoporin 37	ITHACA
NUP62	NUP62	nucleoporin 62	ITHACA
NUP88	NUP88	nucleoporin 88	NMD
NUS1	NUS1	NUS1 dehydrodolichyl diphosphate synthase subunit	ITHACA_RND
NXF5	NXF5	nuclear RNA export factor 5	ITHACA
NXN	NXN	nucleoredoxin	ITHACA
NYX	NYX	nyctalopin	ITHACA
OAT	OAT	ornithine aminotransferase	ITHACA_RND
OBFC1	STN1	STN1 subunit of CST complex	ITHACA
OBSCN	OBSCN	obscurin, cytoskeletal calmodulin and titin-interacting RhoGEF	ITHACA
OBSL1	OBSL1	obscurin like cytoskeletal adaptor 1	ITHACA
OCLN	OCLN	occludin	ITHACA_RND
OCM2	OCM2	oncomodulin 2	ITHACA
OCRL	OCRL	OCRL inositol polyphosphate-5-phosphatase	ITHACA_RND
ODC1	ODC1	ornithine decarboxylase 1	ITHACA_RND
OFD1	OFD1	OFD1 centriole and centriolar satellite protein	ITHACA_RND
OGDH	OGDH	oxoglutarate dehydrogenase	ITHACA
OGDHL	OGDHL	oxoglutarate dehydrogenase L	ITHACA
OGG1	OGG1	8-oxoguanine DNA glycosylase	GENTURIS
OGT	OGT	O-linked N-acetylglucosamine (GlcNAc) transferase	ITHACA_RND
OPA1	OPA1	OPA1 mitochondrial dynamin like GTPase	ITHACA_NMD_RND
OPA3	OPA3	outer mitochondrial membrane lipid metabolism regulator OPA3	ITHACA_RND
OPHN1	OPHN1	oligophrenin 1	ITHACA_RND
OPTN	OPTN	optineurin	NMD_RND
OR2A12	OR2A12	olfactory receptor family 2 subfamily A member 12	ITHACA
ORAI1	ORAI1	ORAI calcium release-activated calcium modulator 1	NMD_RND
ORC1	ORC1	origin recognition complex subunit 1	ITHACA
ORC4	ORC4	origin recognition complex subunit 4	ITHACA
ORC6	ORC6	origin recognition complex subunit 6	ITHACA
OSGEP	OSGEP	O-sialoglycoprotein endopeptidase	ITHACA_RND
OSTC	OSTC	oligosaccharyltransferase complex non-catalytic subunit	ITHACA
OSTM1	OSTM1	osteoclastogenesis associated transmembrane protein 1	RND
OTC	OTC	ornithine transcarbamylase	ITHACA_RND

OTUD5	OTUD5	OTU deubiquitinase 5	ITHACA
OTUD6B	OTUD6B	OTU deubiquitinase 6B	ITHACA_RND
OTUD7A	OTUD7A	OTU deubiquitinase 7A	ITHACA
OTX2	OTX2	orthodenticle homeobox 2	ITHACA_RND
OXCT1	OXCT1	3-oxoacid CoA-transferase 1	RND
OXR1	OXR1	oxidation resistance 1	ITHACA_RND
P4HA3	P4HA3	prolyl 4-hydroxylase subunit alpha 3	ITHACA
P4HB	P4HB	prolyl 4-hydroxylase subunit beta	ITHACA
P4HTM	P4HTM	prolyl 4-hydroxylase, transmembrane	ITHACA_RND
PABPN1	PABPN1	poly(A) binding protein nuclear 1	NMD
PACS1	PACS1	phosphofurin acidic cluster sorting protein 1	ITHACA_RND
PACS2	PACS2	phosphofurin acidic cluster sorting protein 2	ITHACA_RND
PAFAH1B1	PAFAH1B1	platelet activating factor acetylhydrolase 1b regulatory subunit 1	ITHACA_RND
PAH	PAH	phenylalanine hydroxylase	ITHACA_RND
PAICS	PAICS	phosphoribosylaminoimidazole carboxylase and phosphoribosylaminoimidazolesuccinocarboxamide synthase	ITHACA
PAK1	PAK1	p21 (RAC1) activated kinase 1	ITHACA_RND
PAK2	PAK2	p21 (RAC1) activated kinase 2	ITHACA
PAK3	PAK3	p21 (RAC1) activated kinase 3	ITHACA_RND
PALB2	PALB2	partner and localizer of BRCA2	GENTURIS
PAM16	PAM16	presequence translocase associated motor 16	ITHACA
PANK2	PANK2	pantothenate kinase 2	ITHACA_RND
PANX1	PANX1	pannexin 1	ITHACA
PAPOLG	PAPOLG	poly(A) polymerase gamma	ITHACA
PARD3B	PARD3B	par-3 family cell polarity regulator beta	ITHACA
PARK2	PRKN	parkin RBR E3 ubiquitin protein ligase	GENTURIS_ITHACA_RND
PARK7	PARK7	Parkinsonism associated deglycase	RND
PARN	PARN	poly(A)-specific ribonuclease	GENTURIS_ITHACA_RND
PARP1	PARP1	poly(ADP-ribose) polymerase 1	ITHACA
PARS2	PARS2	prolyl-tRNA synthetase 2, mitochondrial	ITHACA_RND
PASK	PASK	PAS domain containing serine/threonine kinase	ITHACA
PAX1	PAX1	paired box 1	ITHACA
PAX2	PAX2	paired box 2	ITHACA
PAX3	PAX3	paired box 3	ITHACA
PAX5	PAX5	paired box 5	GENTURIS_ITHACA
PAX6	PAX6	paired box 6	ITHACA_RND
PAX7	PAX7	paired box 7	ITHACA_NMD_RND
PAX8	PAX8	paired box 8	ITHACA_RND
PAX9	PAX9	paired box 9	ITHACA
PBX1	PBX1	PBX homeobox 1	ITHACA_RND
PC	PC	pyruvate carboxylase	ITHACA_RND
PCBD1	PCBD1	pterin-4 alpha-carbinolamine dehydratase 1	ITHACA_RND
PCBP1	PCBP1	poly(rC) binding protein 1	ITHACA
PCCA	PCCA	propionyl-CoA carboxylase subunit alpha	ITHACA_RND
PCCB	PCCB	propionyl-CoA carboxylase subunit beta	ITHACA_RND

PCDH12	PCDH12	protocadherin 12	ITHACA_RND
PCDH15	PCDH15	protocadherin related 15	ITHACA
PCDH19	PCDH19	protocadherin 19	ITHACA_RND
PCDHA13	PCDHA13	protocadherin alpha 13	ITHACA
PCDHB4	PCDHB4	protocadherin beta 4	ITHACA
PCDHGA10	PCDHGA10	protocadherin gamma subfamily A, 10	ITHACA
PCGF2	PCGF2	polycomb group ring finger 2	ITHACA_RND
PCK1	PCK1	phosphoenolpyruvate carboxykinase 1	ITHACA_RND
PCLO	PCLO	piccolo presynaptic cytomatrix protein	ITHACA
PCNA	PCNA	proliferating cell nuclear antigen	ITHACA_NMD
PCNT	PCNT	pericentrin	ITHACA_RND
PCOLCE	PCOLCE	procollagen C-endopeptidase enhancer	ITHACA
PCSK9	PCSK9	proprotein convertase subtilisin/kexin type 9	RND
PCYT2	PCYT2	phosphate cytidylyltransferase 2, ethanolamine	ITHACA_RND
PDCD1	PDCD1	programmed cell death 1	ITHACA
PDCD6IP	PDCD6IP	programmed cell death 6 interacting protein	ITHACA
PDE10A	PDE10A	phosphodiesterase 10A	ITHACA_RND
PDE2A	PDE2A	phosphodiesterase 2A	ITHACA_RND
PDE4D	PDE4D	phosphodiesterase 4D	ITHACA_RND
PDE4DIP	PDE4DIP	phosphodiesterase 4D interacting protein	ITHACA
PDE6D	PDE6D	phosphodiesterase 6D	ITHACA
PDGFB	PDGFB	platelet derived growth factor subunit B	GENTURIS_RND
PDGFRA	PDGFRA	platelet derived growth factor receptor alpha	GENTURIS
PDGFRB	PDGFRB	platelet derived growth factor receptor beta	ITHACA_RND
PDHA1	PDHA1	pyruvate dehydrogenase E1 subunit alpha 1	ITHACA_RND
PDHB	PDHB	pyruvate dehydrogenase E1 subunit beta	ITHACA_RND
PDHX	PDHX	pyruvate dehydrogenase complex component X	ITHACA_RND
PDK2	PDK2	pyruvate dehydrogenase kinase 2	ITHACA
PDK3	PDK3	pyruvate dehydrogenase kinase 3	NMD
PDP1	PDP1	pyruvate dehydrogenase phosphatase catalytic subunit 1	ITHACA_RND
PDPR	PDPR	pyruvate dehydrogenase phosphatase regulatory subunit	ITHACA
PDSS1	PDSS1	decaprenyl diphosphate synthase subunit 1	ITHACA_RND
PDSS2	PDSS2	decaprenyl diphosphate synthase subunit 2	ITHACA_RND
PDYN	PDYN	prodynorphin	NMD_RND
PECR	PECR	peroxisomal trans-2-enoyl-CoA reductase	ITHACA
PEPD	PEPD	peptidase D	ITHACA_RND
PET100	PET100	PET100 cytochrome c oxidase chaperone	ITHACA_RND
PET117	PET117	PET117 cytochrome c oxidase chaperone	ITHACA
PEX1	PEX1	peroxisomal biogenesis factor 1	ITHACA_RND
PEX10	PEX10	peroxisomal biogenesis factor 10	ITHACA_RND
PEX11B	PEX11B	peroxisomal biogenesis factor 11 beta	ITHACA_RND
PEX12	PEX12	peroxisomal biogenesis factor 12	ITHACA_RND
PEX13	PEX13	peroxisomal biogenesis factor 13	ITHACA_RND
PEX14	PEX14	peroxisomal biogenesis factor 14	ITHACA_RND
PEX16	PEX16	peroxisomal biogenesis factor 16	ITHACA_RND
PEX19	PEX19	peroxisomal biogenesis factor 19	ITHACA_RND
PEX2	PEX2	peroxisomal biogenesis factor 2	ITHACA_RND
PEX26	PEX26	peroxisomal biogenesis factor 26	ITHACA_RND

PEX3	PEX3	peroxisomal biogenesis factor 3	ITHACA_RND
PEX5	PEX5	peroxisomal biogenesis factor 5	ITHACA_RND
PEX6	PEX6	peroxisomal biogenesis factor 6	ITHACA_RND
PEX7	PEX7	peroxisomal biogenesis factor 7	ITHACA_NMD_RND
PFKM	PFKM	phosphofructokinase, muscle	NMD_RND
PFN1	PFN1	profilin 1	NMD_RND
PGA5	PGA5	pepsinogen A5	ITHACA
PGAM2	PGAM2	phosphoglycerate mutase 2	NMD_RND
PGAM5	PGAM5	PGAM family member 5, mitochondrial serine/threonine protein phosphatase	ITHACA
PGAP1	PGAP1	post-GPI attachment to proteins inositol deacylase 1	ITHACA_RND
PGAP2	PGAP2	post-GPI attachment to proteins 2	ITHACA_RND
PGAP3	PGAP3	post-GPI attachment to proteins phospholipase 3	ITHACA_RND
PGK1	PGK1	phosphoglycerate kinase 1	ITHACA_NMD_RND
PGM1	PGM1	phosphoglucomutase 1	ITHACA_NMD_RND
PGM3	PGM3	phosphoglucomutase 3	ITHACA_RND
PHACTR1	PHACTR1	phosphatase and actin regulator 1	ITHACA_RND
PHC1	PHC1	polyhomeotic homolog 1	ITHACA
PHF12	PHF12	PHD finger protein 12	ITHACA
PHF2	PHF2	PHD finger protein 2	ITHACA
PHF21A	PHF21A	PHD finger protein 21A	ITHACA_RND
PHF3	PHF3	PHD finger protein 3	ITHACA
PHF6	PHF6	PHD finger protein 6	ITHACA_RND
PHF7	PHF7	PHD finger protein 7	ITHACA
PHF8	PHF8	PHD finger protein 8	ITHACA_RND
PHGDH	PHGDH	phosphoglycerate dehydrogenase	ITHACA_RND
PHIP	PHIP	pleckstrin homology domain interacting protein	ITHACA_RND
PHKA1	PHKA1	phosphorylase kinase regulatory subunit alpha 1	NMD_RND
PHKA2	PHKA2	phosphorylase kinase regulatory subunit alpha 2	ITHACA_RND
PHKB	PHKB	phosphorylase kinase regulatory subunit beta	RND
PHKG2	PHKG2	phosphorylase kinase catalytic subunit gamma 2	ITHACA_RND
PHLPP1	PHLPP1	PH domain and leucine rich repeat protein phosphatase 1	ITHACA
PHOX2A	PHOX2A	paired like homeobox 2A	NMD
PHOX2B	PHOX2B	paired like homeobox 2B	GENTURIS_ITHACA
PHYH	PHYH	phytanoyl-CoA 2-hydroxylase	NMD_RND
PI4K2A	PI4K2A	phosphatidylinositol 4-kinase type 2 alpha	ITHACA
PI4KA	PI4KA	phosphatidylinositol 4-kinase alpha	ITHACA
PIANP	PIANP	PILR alpha associated neural protein	ITHACA
PIAS4	PIAS4	protein inhibitor of activated STAT 4	ITHACA
PIBF1	PIBF1	progesterone immunomodulatory binding factor 1	ITHACA
PIDD	PIDD1	p53-induced death domain protein 1	ITHACA
PIEZO1	PIEZO1	piezo type mechanosensitive ion channel component 1	ITHACA

PIEZO2	PIEZO2	piezo type mechanosensitive ion channel component 2	ITHACA_NMD_RND
PIGA	PIGA	phosphatidylinositol glycan anchor biosynthesis class A	ITHACA_RND
PIGB	PIGB	phosphatidylinositol glycan anchor biosynthesis class B	ITHACA_RND
PIGC	PIGC	phosphatidylinositol glycan anchor biosynthesis class C	ITHACA_RND
PIGF	PIGF	phosphatidylinositol glycan anchor biosynthesis class F	ITHACA
PIGG	PIGG	phosphatidylinositol glycan anchor biosynthesis class G	ITHACA_RND
PIGH	PIGH	phosphatidylinositol glycan anchor biosynthesis class H	ITHACA_RND
PIGK	PIGK	phosphatidylinositol glycan anchor biosynthesis class K	ITHACA
PIGL	PIGL	phosphatidylinositol glycan anchor biosynthesis class L	ITHACA_RND
PIGM	PIGM	phosphatidylinositol glycan anchor biosynthesis class M	ITHACA
PIGN	PIGN	phosphatidylinositol glycan anchor biosynthesis class N	ITHACA_RND
PIGO	PIGO	phosphatidylinositol glycan anchor biosynthesis class O	ITHACA_RND
PIGP	PIGP	phosphatidylinositol glycan anchor biosynthesis class P	ITHACA_RND
PIGQ	PIGQ	phosphatidylinositol glycan anchor biosynthesis class Q	ITHACA_RND
PIGS	PIGS	phosphatidylinositol glycan anchor biosynthesis class S	ITHACA_RND
PIGT	PIGT	phosphatidylinositol glycan anchor biosynthesis class T	ITHACA_RND
PIGU	PIGU	phosphatidylinositol glycan anchor biosynthesis class U	ITHACA_RND
PIGV	PIGV	phosphatidylinositol glycan anchor biosynthesis class V	ITHACA_RND
PIGW	PIGW	phosphatidylinositol glycan anchor biosynthesis class W	ITHACA_RND
PIGY	PIGY	phosphatidylinositol glycan anchor biosynthesis class Y	ITHACA
PIK3AP1	PIK3AP1	phosphoinositide-3-kinase adaptor protein 1	ITHACA
PIK3C2A	PIK3C2A	phosphatidylinositol-4-phosphate 3-kinase catalytic subunit type 2 alpha	ITHACA
PIK3C3	PIK3C3	phosphatidylinositol 3-kinase catalytic subunit type 3	ITHACA
PIK3CA	PIK3CA	phosphatidylinositol-4,5-bisphosphate 3-kinase catalytic subunit alpha	GENTURIS_ITHACA_RND
PIK3R1	PIK3R1	phosphoinositide-3-kinase regulatory subunit 1	ITHACA
PIK3R2	PIK3R2	phosphoinositide-3-kinase regulatory subunit 2	ITHACA_RND
PINK1	PINK1	PTEN induced kinase 1	RND
PIP5K1A	PIP5K1A	phosphatidylinositol-4-phosphate 5-kinase type 1 alpha	ITHACA

PIP5K1C	PIP5K1C	phosphatidylinositol-4-phosphate 5-kinase type 1 gamma	ITHACA_NMD
PISD	PISD	phosphatidylserine decarboxylase	ITHACA
PITRM1	PITRM1	pitrilysin metallopeptidase 1	ITHACA_RND
PITX3	PITX3	paired like homeodomain 3	ITHACA
PIWIL1	PIWIL1	piwi like RNA-mediated gene silencing 1	ITHACA
PJA1	PJA1	praja ring finger ubiquitin ligase 1	ITHACA
PKN2	PKN2	protein kinase N2	ITHACA
PKP2	PKP2	plakophilin 2	NMD
PLA2G6	PLA2G6	phospholipase A2 group VI	ITHACA_RND
PLAA	PLAA	phospholipase A2 activating protein	ITHACA_RND
PLAC8L1	PLAC8L1	PLAC8 like 1	ITHACA
PLCB1	PLCB1	phospholipase C beta 1	ITHACA_RND
PLCB3	PLCB3	phospholipase C beta 3	ITHACA
PLCD1	PLCD1	phospholipase C delta 1	ITHACA
PLCH1	PLCH1	phospholipase C eta 1	ITHACA
PLCH2	PLCH2	phospholipase C eta 2	ITHACA
PLD3	PLD3	phospholipase D family member 3	NMD
PLEC	PLEC	plectin	ITHACA_NMD_RND
PLEKHG2	PLEKHG2	pleckstrin homology and RhoGEF domain containing G2	ITHACA
PLEKHG5	PLEKHG5	pleckstrin homology and RhoGEF domain containing G5	NMD_RND
PLG	PLG	plasminogen	RND
PLIN1	PLIN1	perilipin 1	ITHACA
PLK2	PLK2	polo like kinase 2	ITHACA
PLK4	PLK4	polo like kinase 4	ITHACA_RND
PLK5	PLK5	polo like kinase 5 (inactive)	ITHACA
PLN	PLN	phospholamban	NMD
PLOD1	PLOD1	procollagen-lysine,2-oxoglutarate 5-dioxygenase 1	ITHACA
PLOD3	PLOD3	procollagen-lysine,2-oxoglutarate 5-dioxygenase 3	ITHACA
PLP1	PLP1	proteolipid protein 1	ITHACA_NMD_RND
PLXNA1	PLXNA1	plexin A1	ITHACA
PLXNA2	PLXNA2	plexin A2	ITHACA
PLXNA3	PLXNA3	plexin A3	ITHACA
PLXNB1	PLXNB1	plexin B1	ITHACA
PLXND1	PLXND1	plexin D1	ITHACA
PMM2	PMM2	phosphomannomutase 2	ITHACA_RND
PMP2	PMP2	peripheral myelin protein 2	NMD_RND
PMP22	PMP22	peripheral myelin protein 22	ITHACA_NMD_RND
PMPCA	PMPCA	peptidase, mitochondrial processing subunit alpha	ITHACA_RND
PMPCB	PMPCB	peptidase, mitochondrial processing subunit beta	ITHACA_RND
PMS1	PMS1	PMS1 homolog 1, mismatch repair system component	GENTURIS

PMS2	PMS2	PMS1 homolog 2, mismatch repair system component	GENTURIS
PMS2CL	PMS2CL	PMS2 C-terminal like pseudogene	GENTURIS
PNKD	PNKD	PNKD metallo-beta-lactamase domain containing	RND
PNKP	PNKP	polynucleotide kinase 3'-phosphatase	ITHACA_NMD_RND
PNP	PNP	purine nucleoside phosphorylase	ITHACA_RND
PNPLA2	PNPLA2	patatin like phospholipase domain containing 2	ITHACA_NMD
PNPLA6	PNPLA6	patatin like phospholipase domain containing 6	ITHACA_NMD_RND
PNPLA8	PNPLA8	patatin like phospholipase domain containing 8	NMD_RND
PNPO	PNPO	pyridoxamine 5'-phosphate oxidase	ITHACA_RND
PNPT1	PNPT1	polyribonucleotide nucleotidyltransferase 1	ITHACA_RND
POC1A	POC1A	POC1 centriolar protein A	ITHACA
POFUT1	POFUT1	protein O-fucosyltransferase 1	ITHACA
POGLUT1	POGLUT1	protein O-glucosyltransferase 1	NMD
POGZ	POGZ	pogo transposable element derived with ZNF domain	ITHACA_RND
POLA1	POLA1	DNA polymerase alpha 1, catalytic subunit	ITHACA_RND
POLD1	POLD1	DNA polymerase delta 1, catalytic subunit	GENTURIS
POLD2	POLD2	DNA polymerase delta 2, accessory subunit	GENTURIS
POLD3	POLD3	DNA polymerase delta 3, accessory subunit	GENTURIS
POLD4	POLD4	DNA polymerase delta 4, accessory subunit	GENTURIS
POLE	POLE	DNA polymerase epsilon, catalytic subunit	GENTURIS
POLE2	POLE2	DNA polymerase epsilon 2, accessory subunit	GENTURIS
POLE3	POLE3	DNA polymerase epsilon 3, accessory subunit	GENTURIS
POLE4	POLE4	DNA polymerase epsilon 4, accessory subunit	GENTURIS
POLG	POLG	DNA polymerase gamma, catalytic subunit	ITHACA_NMD_RND
POLG2	POLG2	DNA polymerase gamma 2, accessory subunit	ITHACA_NMD_RND
POLH	POLH	DNA polymerase eta	GENTURIS_RND
POLR1C	POLR1C	RNA polymerase I and III subunit C	ITHACA_RND
POLR2A	POLR2A	RNA polymerase II subunit A	ITHACA_RND
POLR3A	POLR3A	RNA polymerase III subunit A	ITHACA_RND
POLR3B	POLR3B	RNA polymerase III subunit B	ITHACA_RND
POLR3GL	POLR3GL	RNA polymerase III subunit GL	ITHACA
POLRMT	POLRMT	RNA polymerase mitochondrial	ITHACA
POMGNT1	POMGNT1	protein O-linked mannose N-acetylglucosaminyltransferase 1 (beta 1,2-)	ITHACA_NMD_RND
POMGNT2	POMGNT2	protein O-linked mannose N-acetylglucosaminyltransferase 2 (beta 1,4-)	ITHACA_NMD_RND
POMK	POMK	protein O-mannose kinase	ITHACA_NMD_RND
POMT1	POMT1	protein O-mannosyltransferase 1	ITHACA_NMD_RND
POMT2	POMT2	protein O-mannosyltransferase 2	ITHACA_NMD_RND
POP1	POP1	POP1 homolog, ribonuclease P/MRP subunit	ITHACA
POPDC3	POPDC3	popeye domain containing 3	NMD

POR	POR	cytochrome p450 oxidoreductase	ITHACA_RND
PORCN	PORCN	porcupine O-acyltransferase	ITHACA_RND
POT1	POT1	protection of telomeres 1	GENTURIS
POU1F1	POU1F1	POU class 1 homeobox 1	ITHACA
POU3F2	POU3F2	POU class 3 homeobox 2	ITHACA
POU3F3	POU3F3	POU class 3 homeobox 3	ITHACA_RND
POU6F2	POU6F2	POU class 6 homeobox 2	GENTURIS
PPA2	PPA2	inorganic pyrophosphatase 2	RND
PPARGC1A	PPARGC1A	PPARG coactivator 1 alpha	ITHACA
PPFIA1	PPFIA1	PTPRF interacting protein alpha 1	ITHACA
PPFIBP1	PPFIBP1	PPFIA binding protein 1	ITHACA
PPIL1	PPIL1	peptidylprolyl isomerase like 1	ITHACA
PPM1D	PPM1D	protein phosphatase, Mg2+/Mn2+ dependent 1D	GENTURIS_ITHACA_RND
PPOX	PPOX	protoporphyrinogen oxidase	ITHACA_RND
PPP1CB	PPP1CB	protein phosphatase 1 catalytic subunit beta	ITHACA_RND
PPP1R12A	PPP1R12A	protein phosphatase 1 regulatory subunit 12A	ITHACA
PPP1R15B	PPP1R15B	protein phosphatase 1 regulatory subunit 15B	ITHACA_RND
PPP1R21	PPP1R21	protein phosphatase 1 regulatory subunit 21	ITHACA_RND
PPP1R35	PPP1R35	protein phosphatase 1 regulatory subunit 35	ITHACA
PPP2CA	PPP2CA	protein phosphatase 2 catalytic subunit alpha	ITHACA_RND
PPP2R1A	PPP2R1A	protein phosphatase 2 scaffold subunit Aalpha	ITHACA_RND
PPP2R2B	PPP2R2B	protein phosphatase 2 regulatory subunit Bbeta	NMD
PPP2R2C	PPP2R2C	protein phosphatase 2 regulatory subunit Bgamma	ITHACA
PPP2R5A	PPP2R5A	protein phosphatase 2 regulatory subunit B'alpha	ITHACA
PPP2R5B	PPP2R5B	protein phosphatase 2 regulatory subunit B'beta	ITHACA
PPP2R5C	PPP2R5C	protein phosphatase 2 regulatory subunit B'gamma	ITHACA
PPP2R5D	PPP2R5D	protein phosphatase 2 regulatory subunit B'delta	ITHACA_RND
PPP3CA	PPP3CA	protein phosphatase 3 catalytic subunit alpha	ITHACA_RND
PPRC1	PPRC1	PPARG related coactivator 1	ITHACA
PPT1	PPT1	palmitoyl-protein thioesterase 1	ITHACA_RND
PQBP1	PQBP1	polyglutamine binding protein 1	ITHACA_RND
PRDM12	PRDM12	PR/SET domain 12	NMD_RND
PRDM16	PRDM16	PR/SET domain 16	NMD
PRDX1	PRDX1	peroxiredoxin 1	ITHACA
PREP	PREP	prolyl endopeptidase	ITHACA
PREPL	PREPL	prolyl endopeptidase like	ITHACA_NMD_RND
PRF1	PRF1	perforin 1	GENTURIS_ITHACA
PRICKLE1	PRICKLE1	prickle planar cell polarity protein 1	ITHACA_RND
PRICKLE2	PRICKLE2	prickle planar cell polarity protein 2	ITHACA
PRIM1	PRIM1	DNA primase subunit 1	ITHACA
PRIMA1	PRIMA1	proline rich membrane anchor 1	ITHACA
PRKACA	PRKACA	protein kinase cAMP-activated catalytic subunit alpha	ITHACA
PRKACB	PRKACB	protein kinase cAMP-activated catalytic subunit beta	ITHACA

PRKAG2	PRKAG2	protein kinase AMP-activated non-catalytic subunit gamma 2	NMD_RND
PRKAR1A	PRKAR1A	protein kinase cAMP-dependent type I regulatory subunit alpha	GENTURIS_ITHACA
PRKAR1B	PRKAR1B	protein kinase cAMP-dependent type I regulatory subunit beta	ITHACA
PRKAR2B	PRKAR2B	protein kinase cAMP-dependent type II regulatory subunit beta	ITHACA
PRKCA	PRKCA	protein kinase C alpha	ITHACA
PRKCG	PRKCG	protein kinase C gamma	ITHACA_NMD_RND
PRKD1	PRKD1	protein kinase D1	ITHACA_RND
PRKDC	PRKDC	protein kinase, DNA-activated, catalytic subunit	ITHACA
PRKRA	PRKRA	protein activator of interferon induced protein kinase EIF2AK2	ITHACA_RND
PRMT10	PRMT9	protein arginine methyltransferase 9	ITHACA
PRMT7	PRMT7	protein arginine methyltransferase 7	ITHACA_RND
PRNP	PRNP	prion protein	RND
PROC	PROC	protein C, inactivator of coagulation factors Va and VIIIa	ITHACA
PROCA1	PROCA1	protein interacting with cyclin A1	ITHACA
PRODH	PRODH	proline dehydrogenase 1	ITHACA_RND
PROSC	PLPBP	pyridoxal phosphate binding protein	ITHACA_RND
PROX2	PROX2	prospero homeobox 2	ITHACA
PRPF18	PRPF18	pre-mRNA processing factor 18	ITHACA
PRPF8	PRPF8	pre-mRNA processing factor 8	ITHACA
PRPH	PRPH	peripherin	NMD
PRPS1	PRPS1	phosphoribosyl pyrophosphate synthetase 1	ITHACA_NMD_RND
PRR12	PRR12	proline rich 12	ITHACA_RND
PRRT2	PRRT2	proline rich transmembrane protein 2	ITHACA_RND
PRRX1	PRRX1	paired related homeobox 1	ITHACA
PRSS1	PRSS1	serine protease 1	GENTURIS
PRSS12	PRSS12	serine protease 12	ITHACA_RND
PRUNE	PRUNE1	prune exopolyphosphatase 1	ITHACA_NMD_RND
PRX	PRX	periaxin	ITHACA_NMD_RND
PSAP	PSAP	prosaposin	ITHACA_RND
PSAT1	PSAT1	phosphoserine aminotransferase 1	ITHACA_RND
PSEN1	PSEN1	presenilin 1	NMD_RND
PSEN2	PSEN2	presenilin 2	NMD_RND
PSMA7	PSMA7	proteasome 20S subunit alpha 7	ITHACA
PSMB1	PSMB1	proteasome 20S subunit beta 1	ITHACA
PSMB8	PSMB8	proteasome 20S subunit beta 8	ITHACA
PSMC5	PSMC5	proteasome 26S subunit, ATPase 5	ITHACA
PSMD12	PSMD12	proteasome 26S subunit, non-ATPase 12	ITHACA_RND
PSMG2	PSMG2	proteasome assembly chaperone 2	ITHACA
PSMG4	PSMG4	proteasome assembly chaperone 4	ITHACA
PSPH	PSPH	phosphoserine phosphatase	ITHACA_RND
PTBP1	PTBP1	polypyrimidine tract binding protein 1	ITHACA

PTCH1	PTCH1	patched 1	GENTURIS_ITHACA_RND
PTCH2	PTCH2	patched 2	GENTURIS_ITHACA
PTCHD1	PTCHD1	patched domain containing 1	ITHACA_RND
PTCHD2	DISP3	dispatched RND transporter family member 3	ITHACA
PTDSS1	PTDSS1	phosphatidylserine synthase 1	ITHACA_RND
PTEN	PTEN	phosphatase and tensin homolog	GENTURIS_ITHACA_RND
PTF1A	PTF1A	pancreas associated transcription factor 1a	ITHACA_RND
PTH1R	PTH1R	parathyroid hormone 1 receptor	ITHACA
PTH2R	PTH2R	parathyroid hormone 2 receptor	ITHACA
PTHLH	PTHLH	parathyroid hormone like hormone	ITHACA
PTK7	PTK7	protein tyrosine kinase 7 (inactive)	ITHACA
PTPLA	HACD1	3-hydroxyacyl-CoA dehydratase 1	NMD
PTPN11	PTPN11	protein tyrosine phosphatase non-receptor type 11	GENTURIS_ITHACA_RND
PTPN12	PTPN12	protein tyrosine phosphatase non-receptor type 12	GENTURIS
PTPN23	PTPN23	protein tyrosine phosphatase non-receptor type 23	ITHACA_RND
PTPN4	PTPN4	protein tyrosine phosphatase non-receptor type 4	ITHACA
PTPRD	PTPRD	protein tyrosine phosphatase receptor type D	ITHACA
PTPRJ	PTPRJ	protein tyrosine phosphatase receptor type J	GENTURIS
PTPRK	PTPRK	protein tyrosine phosphatase receptor type K	ITHACA
PTPRQ	PTPRQ	protein tyrosine phosphatase receptor type Q	ITHACA
PTPRT	PTPRT	protein tyrosine phosphatase receptor type T	ITHACA
PTRF	CAVIN1	caveolae associated protein 1	NMD
PTRH2	PTRH2	peptidyl-tRNA hydrolase 2	ITHACA_NMD_RND
PTRHD1	PTRHD1	peptidyl-tRNA hydrolase domain containing 1	ITHACA
PTS	PTS	6-pyruvoyltetrahydropterin synthase	ITHACA_RND
PUF60	PUF60	poly(U) binding splicing factor 60	ITHACA_RND
PUM1	PUM1	pumilio RNA binding family member 1	ITHACA_NMD_RND
PUM2	PUM2	pumilio RNA binding family member 2	ITHACA
PURA	PURA	purine rich element binding protein A	ITHACA_NMD_RND
PUS1	PUS1	pseudouridine synthase 1	ITHACA_NMD_RND
PUS3	PUS3	pseudouridine synthase 3	ITHACA_RND
PUS7	PUS7	pseudouridine synthase 7	ITHACA_RND
PVRL1	NECTIN1	nectin cell adhesion molecule 1	ITHACA
PWRN1	PWRN1	Prader-Willi region non-protein coding RNA 1	ITHACA
PYCR1	PYCR1	pyrroline-5-carboxylate reductase 1	ITHACA_RND
PYCR2	PYCR2	pyrroline-5-carboxylate reductase 2	ITHACA_RND
PYGL	PYGL	glycogen phosphorylase L	RND
PYGM	PYGM	glycogen phosphorylase, muscle associated	NMD_RND
PYROXD1	PYROXD1	pyridine nucleotide-disulphide oxidoreductase domain 1	NMD_RND
QARS	QARS1	glutaminyl-tRNA synthetase 1	ITHACA_RND

QDPR	QDPR	quinoid dihydropteridine reductase	ITHACA_RND
QRFPR	QRFPR	pyroglutamylated RFamide peptide receptor	ITHACA
QRICH1	QRICH1	glutamine rich 1	ITHACA_RND
QRSL1	QRSL1	glutaminyl-tRNA amidotransferase subunit QRSL1	ITHACA_RND
R3HDM1	R3HDM1	R3H domain containing 1	ITHACA
RAB11A	RAB11A	RAB11A, member RAS oncogene family	ITHACA
RAB11B	RAB11B	RAB11B, member RAS oncogene family	ITHACA_RND
RAB14	RAB14	RAB14, member RAS oncogene family	ITHACA
RAB18	RAB18	RAB18, member RAS oncogene family	ITHACA_RND
RAB23	RAB23	RAB23, member RAS oncogene family	ITHACA_RND
RAB27A	RAB27A	RAB27A, member RAS oncogene family	ITHACA
RAB2A	RAB2A	RAB2A, member RAS oncogene family	ITHACA
RAB39B	RAB39B	RAB39B, member RAS oncogene family	ITHACA_RND
RAB3GAP1	RAB3GAP1	RAB3 GTPase activating protein catalytic subunit 1	ITHACA_RND
RAB3GAP2	RAB3GAP2	RAB3 GTPase activating non-catalytic protein subunit 2	ITHACA_RND
RAB40AL	RAB40AL	RAB40A like	ITHACA
RAB7A	RAB7A	RAB7A, member RAS oncogene family	NMD_RND
RABGGTA	RABGGTA	Rab geranylgeranyltransferase subunit alpha	ITHACA
RABL6	RABL6	RAB, member RAS oncogene family like 6	ITHACA
RAC1	RAC1	Rac family small GTPase 1	ITHACA_RND
RAC3	RAC3	Rac family small GTPase 3	ITHACA_RND
RAD21	RAD21	RAD21 cohesin complex component	ITHACA_RND
RAD21L1	RAD21L1	RAD21 cohesin complex component like 1	ITHACA
RAD50	RAD50	RAD50 double strand break repair protein	GENTURIS_ITHACA
RAD51	RAD51	RAD51 recombinase	ITHACA
RAD51C	RAD51C	RAD51 paralog C	GENTURIS_ITHACA
RAD51D	RAD51D	RAD51 paralog D	GENTURIS
RAF1	RAF1	Raf-1 proto-oncogene, serine/threonine kinase	GENTURIS_ITHACA_NMD_RND
RAI1	RAI1	retinoic acid induced 1	ITHACA_RND
RALA	RALA	RAS like proto-oncogene A	ITHACA_RND
RALGAPA1	RALGAPA1	Ral GTPase activating protein catalytic subunit alpha 1	ITHACA
RALGAPB	RALGAPB	Ral GTPase activating protein non-catalytic subunit beta	ITHACA
RALGDS	RALGDS	ral guanine nucleotide dissociation stimulator	ITHACA
RANBP2	RANBP2	RAN binding protein 2	ITHACA
RAP1B	RAP1B	RAP1B, member of RAS oncogene family	ITHACA
RAP1GDS1	RAP1GDS1	Rap1 GTPase-GDP dissociation stimulator 1	ITHACA
RAPGEF1	RAPGEF1	Rap guanine nucleotide exchange factor 1	ITHACA
RAPSN	RAPSN	receptor associated protein of the synapse	ITHACA_NMD_RND
RARB	RARB	retinoic acid receptor beta	ITHACA_RND
RARS	RARS1	arginyl-tRNA synthetase 1	ITHACA_RND
RARS2	RARS2	arginyl-tRNA synthetase 2, mitochondrial	ITHACA_RND
RAX	RAX	retina and anterior neural fold homeobox	ITHACA
RB1	RB1	RB transcriptional corepressor 1	GENTURIS

RBBP8	RBBP8	RB binding protein 8, endonuclease	ITHACA_RND
RBCK1	RBCK1	RANBP2-type and C3HC4-type zinc finger containing 1	NMD_RND
RBFOX1	RBFOX1	RNA binding fox-1 homolog 1	ITHACA
RBM10	RBM10	RNA binding motif protein 10	ITHACA_RND
RBM20	RBM20	RNA binding motif protein 20	NMD
RBM26	RBM26	RNA binding motif protein 26	ITHACA
RBM27	RBM27	RNA binding motif protein 27	ITHACA
RBM28	RBM28	RNA binding motif protein 28	ITHACA
RBM7	RBM7	RNA binding motif protein 7	NMD
RBM8A	RBM8A	RNA binding motif protein 8A	ITHACA
RBMS3	RBMS3	RNA binding motif single stranded interacting protein 3	ITHACA
RBMX	RBMX	RNA binding motif protein X-linked	ITHACA
RBP4	RBP4	retinol binding protein 4	RND
RBPJ	RBPJ	recombination signal binding protein for immunoglobulin kappa J region	ITHACA
RCAN1	RCAN1	regulator of calcineurin 1	ITHACA
RCBTB1	RCBTB1	RCC1 and BTB domain containing protein 1	ITHACA
RCL1	RCL1	RNA terminal phosphate cyclase like 1	ITHACA
RDH11	RDH11	retinol dehydrogenase 11	ITHACA
RECQL	RECQL	RecQ like helicase	GENTURIS
RECQL4	RECQL4	RecQ like helicase 4	GENTURIS_ITHACA
REEP1	REEP1	receptor accessory protein 1	NMD_RND
REEP2	REEP2	receptor accessory protein 2	NMD_RND
RELN	RELN	reelin	ITHACA_RND
REPS1	REPS1	RALBP1 associated Eps domain containing 1	ITHACA
RERE	RERE	arginine-glutamic acid dipeptide repeats	ITHACA_RND
REST	REST	RE1 silencing transcription factor	GENTURIS
RET	RET	ret proto-oncogene	GENTURIS
REV3L	REV3L	REV3 like, DNA directed polymerase zeta catalytic subunit	ITHACA
RFC1	RFC1	replication factor C subunit 1	NMD
RFT1	RFT1	RFT1 homolog	ITHACA_RND
RFX3	RFX3	regulatory factor X3	ITHACA
RFX4	RFX4	regulatory factor X4	ITHACA
RFX7	RFX7	regulatory factor X7	ITHACA
RFX8	RFX8	regulatory factor X8	ITHACA
RGL1	RGL1	ral guanine nucleotide dissociation stimulator like 1	ITHACA
RGMA	RGMA	repulsive guidance molecule BMP co-receptor a	ITHACA
RGR	RGR	retinal G protein coupled receptor	ITHACA
RGS6	RGS6	regulator of G protein signaling 6	ITHACA
RGS7	RGS7	regulator of G protein signaling 7	ITHACA
RHBDF2	RHBDF2	rhomboid 5 homolog 2	GENTURIS
RHEB	RHEB	Ras homolog, mTORC1 binding	ITHACA
RHOA	RHOA	ras homolog family member A	ITHACA
RHOBTB2	RHOBTB2	Rho related BTB domain containing 2	ITHACA_RND
RILPL2	RILPL2	Rab interacting lysosomal protein like 2	ITHACA
RIMS1	RIMS1	regulating synaptic membrane exocytosis 1	ITHACA

RIMS2	RIMS2	regulating synaptic membrane exocytosis 2	ITHACA
RIN2	RIN2	Ras and Rab interactor 2	ITHACA
RINT1	RINT1	RAD50 interactor 1	GENTURIS_ITHACA
RIPK1	RIPK1	receptor interacting serine/threonine kinase 1	ITHACA
RIPPLY1	RIPPLY1	ripply transcriptional repressor 1	ITHACA
RIT1	RIT1	Ras like without CAAX 1	GENTURIS_ITHACA_RND
RLF	RLF	RLF zinc finger	ITHACA
RLIM	RLIM	ring finger protein, LIM domain interacting	ITHACA_RND
RMND1	RMND1	required for meiotic nuclear division 1 homolog	ITHACA_RND
RMRP	RMRP	RNA component of mitochondrial RNA processing endoribonuclease	ITHACA
RNASEH1	RNASEH1	ribonuclease H1	NMD_RND
RNASEH2A	RNASEH2A	ribonuclease H2 subunit A	ITHACA_RND
RNASEH2B	RNASEH2B	ribonuclease H2 subunit B	ITHACA_RND
RNASEH2C	RNASEH2C	ribonuclease H2 subunit C	ITHACA_RND
RNASET2	RNASET2	ribonuclease T2	ITHACA_RND
RNF113A	RNF113A	ring finger protein 113A	ITHACA
RNF125	RNF125	ring finger protein 125	ITHACA_RND
RNF13	RNF13	ring finger protein 13	ITHACA
RNF135	RNF135	ring finger protein 135	ITHACA
RNF168	RNF168	ring finger protein 168	ITHACA
RNF170	RNF170	ring finger protein 170	RND
RNF216	RNF216	ring finger protein 216	NMD_RND
RNF38	RNF38	ring finger protein 38	ITHACA
RNF43	RNF43	ring finger protein 43	GENTURIS
RNF5	RNF5	ring finger protein 5	ITHACA
RNFT2	RNFT2	ring finger protein, transmembrane 2	ITHACA
RNPC3	RNPC3	RNA binding region (RNP1, RRM) containing 3	ITHACA
RNU12	RNU12	RNA, U12 small nuclear	ITHACA
RNU4ATAC	RNU4ATAC	RNA, U4atac small nuclear	ITHACA
RNU7-1	RNU7-1	RNA, U7 small nuclear 1	ITHACA
ROBO1	ROBO1	roundabout guidance receptor 1	ITHACA
ROBO3	ROBO3	roundabout guidance receptor 3	RND
ROCK2	ROCK2	Rho associated coiled-coil containing protein kinase 2	ITHACA
ROGDI	ROGDI	rogdi atypical leucine zipper	ITHACA_RND
ROR2	ROR2	receptor tyrosine kinase like orphan receptor 2	ITHACA_RND
RORA	RORA	RAR related orphan receptor A	ITHACA_RND
RORB	RORB	RAR related orphan receptor B	ITHACA_RND
ROS1	ROS1	ROS proto-oncogene 1, receptor tyrosine kinase	ITHACA
RPGRIP1	RPGRIP1	RPGR interacting protein 1	ITHACA
RPGRIP1L	RPGRIP1L	RPGRIP1 like	ITHACA_RND
RPH3A	RPH3A	rabphilin 3A	ITHACA_NMD
RPIA	RPIA	ribose 5-phosphate isomerase A	ITHACA_RND
RPL10	RPL10	ribosomal protein L10	ITHACA_RND
RPL11	RPL11	ribosomal protein L11	GENTURIS
RPL26	RPL26	ribosomal protein L26	ITHACA
RPL35A	RPL35A	ribosomal protein L35a	GENTURIS_ITHACA

RPL5	RPL5	ribosomal protein L5	GENTURIS
RPLP1	RPLP1	ribosomal protein lateral stalk subunit P1	ITHACA
RPS10	RPS10	ribosomal protein S10	GENTURIS
RPS17	RPS17	ribosomal protein S17	GENTURIS
RPS19	RPS19	ribosomal protein S19	GENTURIS_ITHACA
RPS20	RPS20	ribosomal protein S20	GENTURIS
RPS23	RPS23	ribosomal protein S23	ITHACA
RPS24	RPS24	ribosomal protein S24	GENTURIS
RPS26	RPS26	ribosomal protein S26	GENTURIS_ITHACA
RPS28	RPS28	ribosomal protein S28	ITHACA
RPS6KA3	RPS6KA3	ribosomal protein S6 kinase A3	ITHACA_RND
RPS7	RPS7	ribosomal protein S7	GENTURIS
RRAS	RRAS	RAS related	ITHACA
RRAS2	RRAS2	RAS related 2	ITHACA
RREB1	RREB1	ras responsive element binding protein 1	ITHACA
RRM2B	RRM2B	ribonucleotide reductase regulatory TP53 inducible subunit M2B	ITHACA_NMD_RND
RRP8	RRP8	ribosomal RNA processing 8	ITHACA
RSPRY1	RSPRY1	ring finger and SPRY domain containing 1	ITHACA
RSRC1	RSRC1	arginine and serine rich coiled-coil 1	ITHACA
RTEL1	RTEL1	regulator of telomere elongation helicase 1	GENTURIS_ITHACA_RND
RTN2	RTN2	reticulon 2	NMD_RND
RTN4IP1	RTN4IP1	reticulon 4 interacting protein 1	ITHACA_RND
RTTN	RTTN	rotatin	ITHACA_RND
RUNX1	RUNX1	RUNX family transcription factor 1	GENTURIS
RUSC2	RUSC2	RUN and SH3 domain containing 2	ITHACA
RXRB	RXRB	retinoid X receptor beta	ITHACA
RYR1	RYR1	ryanodine receptor 1	NMD_RND
RYR2	RYR2	ryanodine receptor 2	NMD
RYR3	RYR3	ryanodine receptor 3	ITHACA_NMD_RND
SACS	SACS	sacsin molecular chaperone	ITHACA_NMD_RND
SALL1	SALL1	spalt like transcription factor 1	ITHACA
SAMD12	SAMD12	sterile alpha motif domain containing 12	ITHACA
SAMD9	SAMD9	sterile alpha motif domain containing 9	GENTURIS_ITHACA_RND
SAMD9L	SAMD9L	sterile alpha motif domain containing 9 like	GENTURIS_RND
SAMHD1	SAMHD1	SAM and HD domain containing deoxynucleoside triphosphate triphosphohydrolase 1	ITHACA_RND
SAR1B	SAR1B	secretion associated Ras related GTPase 1B	ITHACA_RND
SARS	SARS1	seryl-tRNA synthetase 1	ITHACA
SARS2	SARS2	seryl-tRNA synthetase 2, mitochondrial	ITHACA_RND
SASS6	SASS6	SAS-6 centriolar assembly protein	ITHACA
SATB1	SATB1	SATB homeobox 1	ITHACA
SATB2	SATB2	SATB homeobox 2	ITHACA_RND

SBDS	SBDS	SBDS ribosome maturation factor	GENTURIS_ITHACA
SBF1	SBF1	SET binding factor 1	ITHACA_NMD_RND
SBF2	SBF2	SET binding factor 2	NMD_RND
SC5D	SC5D	sterol-C5-desaturase	ITHACA_RND
SCAF4	SCAF4	SR-related CTD associated factor 4	ITHACA
SCAMP5	SCAMP5	secretory carrier membrane protein 5	ITHACA
SCAPER	SCAPER	S-phase cyclin A associated protein in the ER	ITHACA_RND
SCARB2	SCARB2	scavenger receptor class B member 2	RND
SCG2	SCG2	secretogranin II	ITHACA
SCGN	SCGN	secretagogin, EF-hand calcium binding protein	ITHACA
SCHIP1	SCHIP1	schwannomin interacting protein 1	ITHACA
SCLT1	SCLT1	sodium channel and clathrin linker 1	RND
SCN10A	SCN10A	sodium voltage-gated channel alpha subunit 10	ITHACA_RND
SCN11A	SCN11A	sodium voltage-gated channel alpha subunit 11	ITHACA_NMD_RND
SCN1A	SCN1A	sodium voltage-gated channel alpha subunit 1	ITHACA_RND
SCN1B	SCN1B	sodium voltage-gated channel beta subunit 1	ITHACA_NMD_RND
SCN2A	SCN2A	sodium voltage-gated channel alpha subunit 2	ITHACA_RND
SCN2B	SCN2B	sodium voltage-gated channel beta subunit 2	NMD
SCN3A	SCN3A	sodium voltage-gated channel alpha subunit 3	ITHACA_RND
SCN3B	SCN3B	sodium voltage-gated channel beta subunit 3	NMD
SCN4A	SCN4A	sodium voltage-gated channel alpha subunit 4	ITHACA_NMD_RND
SCN4B	SCN4B	sodium voltage-gated channel beta subunit 4	NMD
SCN5A	SCN5A	sodium voltage-gated channel alpha subunit 5	NMD
SCN8A	SCN8A	sodium voltage-gated channel alpha subunit 8	ITHACA_RND
SCN9A	SCN9A	sodium voltage-gated channel alpha subunit 9	NMD_RND
SCO1	SCO1	synthesis of cytochrome C oxidase 1	ITHACA_RND
SCO2	SCO2	synthesis of cytochrome C oxidase 2	ITHACA_NMD_RND
SCP2	SCP2	sterol carrier protein 2	ITHACA_RND
SCUBE2	SCUBE2	signal peptide, CUB domain and EGF like domain containing 2	ITHACA
SCUBE3	SCUBE3	signal peptide, CUB domain and EGF like domain containing 3	ITHACA
SCYL1	SCYL1	SCY1 like pseudokinase 1	ITHACA_NMD_RND
SCYL2	SCYL2	SCY1 like pseudokinase 2	ITHACA
SDCCAG8	SDCCAG8	SHH signaling and ciliogenesis regulator SDCCAG8	ITHACA_RND
SDHA	SDHA	succinate dehydrogenase complex flavoprotein subunit A	GENTURIS_ITHACA_NMD_RND
SDHAF1	SDHAF1	succinate dehydrogenase complex assembly factor 1	ITHACA_RND
SDHAF2	SDHAF2	succinate dehydrogenase complex assembly factor 2	GENTURIS
SDHB	SDHB	succinate dehydrogenase complex iron sulfur subunit B	GENTURIS_ITHACA_RND

SDHC	SDHC	succinate dehydrogenase complex subunit C	GENTURIS
SDHD	SDHD	succinate dehydrogenase complex subunit D	GENTURIS_ITHACA_RND
SDK2	SDK2	sidekick cell adhesion molecule 2	ITHACA
SEC16A	SEC16A	SEC16 homolog A, endoplasmic reticulum export factor	ITHACA
SEC23B	SEC23B	SEC23 homolog B, COPII coat complex component	GENTURIS_RND
SEC23IP	SEC23IP	SEC23 interacting protein	ITHACA
SEC24D	SEC24D	SEC24 homolog D, COPII coat complex component	ITHACA
SEC31A	SEC31A	SEC31 homolog A, COPII coat complex component	ITHACA
SEC61A1	SEC61A1	SEC61 translocon subunit alpha 1	ITHACA
SEMA3A	SEMA3A	semaphorin 3A	ITHACA
SEMA3E	SEMA3E	semaphorin 3E	ITHACA
SEMA4A	SEMA4A	semaphorin 4A	GENTURIS
SEMA5A	SEMA5A	semaphorin 5A	ITHACA
SEMA6A	SEMA6A	semaphorin 6A	ITHACA
SEMA6B	SEMA6B	semaphorin 6B	ITHACA
SEPN1	SELENON	selenoprotein N	NMD_RND
SEPSECS	SEPSECS	Sep (O-phosphoserine) tRNA:Sec (selenocysteine) tRNA synthase	ITHACA_RND
SEPT10	SEPTIN10	septin 10	ITHACA
SEPT6	SEPTIN6	septin 6	ITHACA
SEPT9	SEPTIN9	septin 9	NMD
SERAC1	SERAC1	serine active site containing 1	ITHACA_RND
SET	SET	SET nuclear proto-oncogene	ITHACA_RND
SETBP1	SETBP1	SET binding protein 1	ITHACA_RND
SETD1A	SETD1A	SET domain containing 1A, histone lysine methyltransferase	ITHACA
SETD1B	SETD1B	SET domain containing 1B, histone lysine methyltransferase	ITHACA_RND
SETD2	SETD2	SET domain containing 2, histone lysine methyltransferase	ITHACA_RND
SETD5	SETD5	SET domain containing 5	ITHACA_RND
SETX	SETX	senataxin	NMD_RND
SF1	SF1	splicing factor 1	ITHACA
SF3B1	SF3B1	splicing factor 3b subunit 1	ITHACA
SF3B4	SF3B4	splicing factor 3b subunit 4	ITHACA
SFTPA1	SFTPA1	surfactant protein A1	GENTURIS
SFTPA2	SFTPA2	surfactant protein A2	GENTURIS
SFXN4	SFXN4	sideroflexin 4	ITHACA_RND
SGCA	SGCA	sarcoglycan alpha	NMD_RND
SGCB	SGCB	sarcoglycan beta	NMD_RND
SGCD	SGCD	sarcoglycan delta	NMD_RND
SGCE	SGCE	sarcoglycan epsilon	ITHACA_NMD_RND
SGCG	SGCG	sarcoglycan gamma	NMD_RND
SGMS1	SGMS1	sphingomyelin synthase 1	ITHACA
SGMS2	SGMS2	sphingomyelin synthase 2	ITHACA
SGPL1	SGPL1	sphingosine-1-phosphate lyase 1	ITHACA_NMD_RND

SGSH	SGSH	N-sulfoglucosamine sulfohydrolase	ITHACA_RND
SGSM3	SGSM3	small G protein signaling modulator 3	ITHACA
SH2B3	SH2B3	SH2B adaptor protein 3	GENTURIS
SH3PXD2B	SH3PXD2B	SH3 and PX domains 2B	ITHACA
SH3TC2	SH3TC2	SH3 domain and tetratricopeptide repeats 2	ITHACA_NMD_RND
SHANK1	SHANK1	SH3 and multiple ankyrin repeat domains 1	ITHACA
SHANK2	SHANK2	SH3 and multiple ankyrin repeat domains 2	ITHACA_RND
SHANK3	SHANK3	SH3 and multiple ankyrin repeat domains 3	ITHACA_RND
SHH	SHH	sonic hedgehog signaling molecule	ITHACA_RND
SHISA6	SHISA6	shisa family member 6	ITHACA
SHMT2	SHMT2	serine hydroxymethyltransferase 2	ITHACA
SHOC2	SHOC2	SHOC2 leucine rich repeat scaffold protein	GENTURIS_ITHACA_RND
SHROOM4	SHROOM4	shroom family member 4	ITHACA
SI	SI	sucrase-isomaltase	RND
SIAH1	SIAH1	siah E3 ubiquitin protein ligase 1	ITHACA
SIGMAR1	SIGMAR1	sigma non-opioid intracellular receptor 1	NMD_RND
SIK1	SIK1	salt inducible kinase 1	ITHACA_RND
SIK3	SIK3	SIK family kinase 3	ITHACA
SIL1	SIL1	SIL1 nucleotide exchange factor	ITHACA_NMD_RND
SIM1	SIM1	SIM bHLH transcription factor 1	ITHACA
SIN3A	SIN3A	SIN3 transcription regulator family member A	ITHACA_RND
SIN3B	SIN3B	SIN3 transcription regulator family member B	ITHACA
SIRT2	SIRT2	sirtuin 2	ITHACA
SIX3	SIX3	SIX homeobox 3	ITHACA_RND
SIX4	SIX4	SIX homeobox 4	ITHACA
SIX6	SIX6	SIX homeobox 6	ITHACA
SKA1	SKA1	spindle and kinetochore associated complex subunit 1	ITHACA
SKI	SKI	SKI proto-oncogene	ITHACA_RND
SKIDA1	SKIDA1	SKI/DACH domain containing 1	ITHACA
SKIV2L	SKIC2	SKI2 subunit of superkiller complex	RND
SLAIN1	SLAIN1	SLAIN motif family member 1	ITHACA
SLC10A7	SLC10A7	solute carrier family 10 member 7	ITHACA
SLC12A1	SLC12A1	solute carrier family 12 member 1	ITHACA
SLC12A2	SLC12A2	solute carrier family 12 member 2	ITHACA
SLC12A3	SLC12A3	solute carrier family 12 member 3	RND
SLC12A5	SLC12A5	solute carrier family 12 member 5	ITHACA_RND
SLC12A6	SLC12A6	solute carrier family 12 member 6	ITHACA_NMD_RND
SLC13A5	SLC13A5	solute carrier family 13 member 5	ITHACA_RND
SLC16A1	SLC16A1	solute carrier family 16 member 1	ITHACA_NMD_RND
SLC16A2	SLC16A2	solute carrier family 16 member 2	ITHACA_RND
SLC17A5	SLC17A5	solute carrier family 17 member 5	ITHACA_RND
SLC18A2	SLC18A2	solute carrier family 18 member A2	ITHACA_RND
SLC18A3	SLC18A3	solute carrier family 18 member A3	NMD_RND
SLC19A2	SLC19A2	solute carrier family 19 member 2	ITHACA_RND
SLC19A3	SLC19A3	solute carrier family 19 member 3	ITHACA_RND

SLC1A1	SLC1A1	solute carrier family 1 member 1	ITHACA
SLC1A2	SLC1A2	solute carrier family 1 member 2	ITHACA_RND
SLC1A3	SLC1A3	solute carrier family 1 member 3	ITHACA_NMD_RND
SLC1A4	SLC1A4	solute carrier family 1 member 4	ITHACA_RND
SLC20A2	SLC20A2	solute carrier family 20 member 2	RND
SLC22A20	SLC22A20P	solute carrier family 22 member 20, pseudogene	ITHACA
SLC22A5	SLC22A5	solute carrier family 22 member 5	ITHACA_NMD_RND
SLC25A1	SLC25A1	solute carrier family 25 member 1	ITHACA_NMD_RND
SLC25A12	SLC25A12	solute carrier family 25 member 12	ITHACA_RND
SLC25A13	SLC25A13	solute carrier family 25 member 13	RND
SLC25A15	SLC25A15	solute carrier family 25 member 15	ITHACA_RND
SLC25A19	SLC25A19	solute carrier family 25 member 19	ITHACA_NMD_RND
SLC25A20	SLC25A20	solute carrier family 25 member 20	ITHACA_NMD_RND
SLC25A22	SLC25A22	solute carrier family 25 member 22	ITHACA_RND
SLC25A23	SLC25A23	solute carrier family 25 member 23	ITHACA
SLC25A24	SLC25A24	solute carrier family 25 member 24	ITHACA
SLC25A26	SLC25A26	solute carrier family 25 member 26	ITHACA_RND
SLC25A3	SLC25A3	solute carrier family 25 member 3	RND
SLC25A32	SLC25A32	solute carrier family 25 member 32	RND
SLC25A38	SLC25A38	solute carrier family 25 member 38	ITHACA_RND
SLC25A39	SLC25A39	solute carrier family 25 member 39	ITHACA
SLC25A4	SLC25A4	solute carrier family 25 member 4	ITHACA_NMD_RND
SLC25A42	SLC25A42	solute carrier family 25 member 42	ITHACA_NMD_RND
SLC25A46	SLC25A46	solute carrier family 25 member 46	ITHACA_NMD_RND
SLC26A2	SLC26A2	solute carrier family 26 member 2	ITHACA
SLC26A3	SLC26A3	solute carrier family 26 member 3	GENTURIS
SLC26A4	SLC26A4	solute carrier family 26 member 4	ITHACA
SLC27A4	SLC27A4	solute carrier family 27 member 4	ITHACA
SLC2A1	SLC2A1	solute carrier family 2 member 1	ITHACA_RND
SLC2A10	SLC2A10	solute carrier family 2 member 10	ITHACA
SLC2A2	SLC2A2	solute carrier family 2 member 2	ITHACA_RND
SLC30A10	SLC30A10	solute carrier family 30 member 10	RND
SLC30A5	SLC30A5	solute carrier family 30 member 5	ITHACA
SLC30A9	SLC30A9	solute carrier family 30 member 9	ITHACA
SLC31A1	SLC31A1	solute carrier family 31 member 1	ITHACA
SLC33A1	SLC33A1	solute carrier family 33 member 1	ITHACA_NMD_RND
SLC35A1	SLC35A1	solute carrier family 35 member A1	ITHACA_RND
SLC35A2	SLC35A2	solute carrier family 35 member A2	ITHACA_RND
SLC35A3	SLC35A3	solute carrier family 35 member A3	ITHACA
SLC35C1	SLC35C1	solute carrier family 35 member C1	ITHACA_RND
SLC35D1	SLC35D1	solute carrier family 35 member D1	ITHACA_RND
SLC35F1	SLC35F1	solute carrier family 35 member F1	ITHACA

SLC36A2	SLC36A2	solute carrier family 36 member 2	ITHACA
SLC37A4	SLC37A4	solute carrier family 37 member 4	RND
SLC39A13	SLC39A13	solute carrier family 39 member 13	ITHACA
SLC39A14	SLC39A14	solute carrier family 39 member 14	ITHACA_RND
SLC39A4	SLC39A4	solute carrier family 39 member 4	RND
SLC39A8	SLC39A8	solute carrier family 39 member 8	ITHACA_RND
SLC3A1	SLC3A1	solute carrier family 3 member 1	RND
SLC3A2	SLC3A2	solute carrier family 3 member 2	ITHACA
SLC40A1	SLC40A1	solute carrier family 40 member 1	RND
SLC44A1	SLC44A1	solute carrier family 44 member 1	ITHACA
SLC45A1	SLC45A1	solute carrier family 45 member 1	ITHACA
SLC46A1	SLC46A1	solute carrier family 46 member 1	ITHACA_RND
SLC4A1	SLC4A1	solute carrier family 4 member 1 (Diego blood group)	ITHACA
SLC4A11	SLC4A11	solute carrier family 4 member 11	ITHACA
SLC4A4	SLC4A4	solute carrier family 4 member 4	ITHACA_RND
SLC52A2	SLC52A2	solute carrier family 52 member 2	ITHACA_NMD_RND
SLC52A3	SLC52A3	solute carrier family 52 member 3	ITHACA_NMD_RND
SLC5A1	SLC5A1	solute carrier family 5 member 1	RND
SLC5A5	SLC5A5	solute carrier family 5 member 5	ITHACA
SLC5A6	SLC5A6	solute carrier family 5 member 6	ITHACA
SLC5A7	SLC5A7	solute carrier family 5 member 7	ITHACA_NMD_RND
SLC6A1	SLC6A1	solute carrier family 6 member 1	ITHACA_RND
SLC6A17	SLC6A17	solute carrier family 6 member 17	ITHACA_RND
SLC6A19	SLC6A19	solute carrier family 6 member 19	ITHACA_RND
SLC6A20	SLC6A20	solute carrier family 6 member 20	ITHACA_RND
SLC6A3	SLC6A3	solute carrier family 6 member 3	ITHACA_RND
SLC6A5	SLC6A5	solute carrier family 6 member 5	ITHACA_RND
SLC6A8	SLC6A8	solute carrier family 6 member 8	ITHACA_RND
SLC6A9	SLC6A9	solute carrier family 6 member 9	ITHACA_RND
SLC7A10	SLC7A10	solute carrier family 7 member 10	ITHACA
SLC7A5	SLC7A5	solute carrier family 7 member 5	ITHACA
SLC7A7	SLC7A7	solute carrier family 7 member 7	ITHACA_RND
SLC7A8	SLC7A8	solute carrier family 7 member 8	ITHACA
SLC7A9	SLC7A9	solute carrier family 7 member 9	RND
SLC9A1	SLC9A1	solute carrier family 9 member A1	ITHACA_NMD_RND
SLC9A3R1	SLC9A3R1	SLC9A3 regulator 1	NMD
SLC9A6	SLC9A6	solute carrier family 9 member A6	ITHACA_RND
SLC9A7	SLC9A7	solute carrier family 9 member A7	ITHACA
SLC9A9	SLC9A9	solute carrier family 9 member A9	ITHACA
SLCO1C1	SLCO1C1	solute carrier organic anion transporter family member 1C1	ITHACA
SLITRK5	SLITRK5	SLIT and NTRK like family member 5	ITHACA
SLITRK6	SLITRK6	SLIT and NTRK like family member 6	ITHACA
SLK	SLK	STE20 like kinase	ITHACA
SLX4	SLX4	SLX4 structure-specific endonuclease subunit	GENTURIS_ITHACA_RND

SMAD4	SMAD4	SMAD family member 4	GENTURIS_ITHACA_RND
SMAD6	SMAD6	SMAD family member 6	ITHACA
SMAD9	SMAD9	SMAD family member 9	GENTURIS
SMARCA1	SMARCA1	SWI/SNF related, matrix associated, actin dependent regulator of chromatin, subfamily a, member 1	ITHACA
SMARCA2	SMARCA2	SWI/SNF related, matrix associated, actin dependent regulator of chromatin, subfamily a, member 2	ITHACA_RND
SMARCA4	SMARCA4	SWI/SNF related, matrix associated, actin dependent regulator of chromatin, subfamily a, member 4	GENTURIS_ITHACA_RND
SMARCAL1	SMARCAL1	SWI/SNF related, matrix associated, actin dependent regulator of chromatin, subfamily a like 1	ITHACA
SMARCB1	SMARCB1	SWI/SNF related, matrix associated, actin dependent regulator of chromatin, subfamily b, member 1	GENTURIS_ITHACA_RND
SMARCC2	SMARCC2	SWI/SNF related, matrix associated, actin dependent regulator of chromatin subfamily c member 2	ITHACA_RND
SMARCD1	SMARCD1	SWI/SNF related, matrix associated, actin dependent regulator of chromatin, subfamily d, member 1	ITHACA_RND
SMARCD2	SMARCD2	SWI/SNF related, matrix associated, actin dependent regulator of chromatin, subfamily d, member 2	ITHACA
SMARCE1	SMARCE1	SWI/SNF related, matrix associated, actin dependent regulator of chromatin, subfamily e, member 1	GENTURIS_ITHACA_RND
SMC1A	SMC1A	structural maintenance of chromosomes 1A	ITHACA_RND
SMC3	SMC3	structural maintenance of chromosomes 3	ITHACA_RND
SMCHD1	SMCHD1	structural maintenance of chromosomes flexible hinge domain containing 1	ITHACA_NMD
SMG8	SMG8	SMG8 nonsense mediated mRNA decay factor	ITHACA
SMG9	SMG9	SMG9 nonsense mediated mRNA decay factor	ITHACA
SMN1	SMN1	survival of motor neuron 1, telomeric	NMD_RND
SMO	SMO	smoothened, frizzled class receptor	ITHACA_RND
SMOC1	SMOC1	SPARC related modular calcium binding 1	ITHACA_RND
SMPD1	SMPD1	sphingomyelin phosphodiesterase 1	ITHACA_RND
SMPD4	SMPD4	sphingomyelin phosphodiesterase 4	ITHACA_NMD_RND
SMS	SMS	spermine synthase	ITHACA_RND
SMURF2	SMURF2	SMAD specific E3 ubiquitin protein ligase 2	ITHACA
SMYD5	SMYD5	SMYD family member 5	ITHACA
SNAP25	SNAP25	synaptosome associated protein 25	ITHACA_NMD_RND
SNAP29	SNAP29	synaptosome associated protein 29	ITHACA_RND
SNAPC5	SNAPC5	small nuclear RNA activating complex polypeptide 5	ITHACA
SNAPIN	SNAPIN	SNAP associated protein	ITHACA

SNCA	SNCA	synuclein alpha	RND
SNIP1	SNIP1	Smad nuclear interacting protein 1	ITHACA
SNORD115-1	SNORD115-1	small nucleolar RNA, C/D box 115-1	ITHACA
SNORD116-1	SNORD116-1	small nucleolar RNA, C/D box 116-1	ITHACA
SNORD118	SNORD118	small nucleolar RNA, C/D box 118	ITHACA_RND
SNRPB	SNRPB	small nuclear ribonucleoprotein polypeptides B and B1	ITHACA_RND
SNRPD1	SNRPD1	small nuclear ribonucleoprotein D1 polypeptide	ITHACA
SNRPE	SNRPE	small nuclear ribonucleoprotein polypeptide E	ITHACA
SNRPN	SNRPN	small nuclear ribonucleoprotein polypeptide N	ITHACA
SNTA1	SNTA1	syntrophin alpha 1	NMD
SNTG1	SNTG1	syntrophin gamma 1	ITHACA
SNX10	SNX10	sorting nexin 10	RND
SNX14	SNX14	sorting nexin 14	ITHACA_NMD_RND
SNX27	SNX27	sorting nexin 27	ITHACA
SNX3	SNX3	sorting nexin 3	ITHACA
SNX5	SNX5	sorting nexin 5	ITHACA
SOBP	SOBP	sine oculis binding protein homolog	ITHACA
SOD1	SOD1	superoxide dismutase 1	ITHACA_NMD_RND
SON	SON	SON DNA and RNA binding protein	ITHACA_RND
SORCS3	SORCS3	sortilin related VPS10 domain containing receptor 3	ITHACA
SORD	SORD	sorbitol dehydrogenase	NMD
SOS1	SOS1	SOS Ras/Rac guanine nucleotide exchange factor 1	GENTURIS_ITHACA_RND
SOS2	SOS2	SOS Ras/Rho guanine nucleotide exchange factor 2	ITHACA_RND
SOX10	SOX10	SRY-box transcription factor 10	ITHACA_RND
SOX11	SOX11	SRY-box transcription factor 11	ITHACA_RND
SOX18	SOX18	SRY-box transcription factor 18	ITHACA
SOX2	SOX2	SRY-box transcription factor 2	ITHACA_RND
SOX3	SOX3	SRY-box transcription factor 3	ITHACA_RND
SOX4	SOX4	SRY-box transcription factor 4	ITHACA_RND
SOX5	SOX5	SRY-box transcription factor 5	ITHACA_RND
SOX6	SOX6	SRY-box transcription factor 6	ITHACA
SOX9	SOX9	SRY-box transcription factor 9	ITHACA_RND
SP2	SP2	Sp2 transcription factor	ITHACA
SP7	SP7	Sp7 transcription factor	ITHACA
SPAG17	SPAG17	sperm associated antigen 17	ITHACA
SPAG5	SPAG5	sperm associated antigen 5	ITHACA
SPARC	SPARC	secreted protein acidic and cysteine rich	ITHACA
SPAST	SPAST	spastin	ITHACA_NMD_RND
SPATA13	SPATA13	spermatogenesis associated 13	ITHACA
SPATA5	SPATA5	spermatogenesis associated 5	ITHACA_RND
SPDL1	SPDL1	spindle apparatus coiled-coil protein 1	ITHACA
SPECC1L	SPECC1L	sperm antigen with calponin homology and coiled-coil domains 1 like	ITHACA_RND

SPEG	SPEG	striated muscle enriched protein kinase	ITHACA_NMD_RND
SPEN	SPEN	spen family transcriptional repressor	ITHACA
SPG11	SPG11	SPG11 vesicle trafficking associated, spatacsin	ITHACA_NMD_RND
SPG20	SPART	spartin	ITHACA_NMD_RND
SPG21	SPG21	SPG21 abhydrolase domain containing, maspardin	ITHACA_NMD_RND
SPG7	SPG7	SPG7 matrix AAA peptidase subunit, paraplegin	NMD_RND
SPINK1	SPINK1	serine peptidase inhibitor Kazal type 1	GENTURIS
SPINK5	SPINK5	serine peptidase inhibitor Kazal type 5	ITHACA
SPOCK1	SPOCK1	SPARC (osteonectin), cwcv and kazal like domains proteoglycan 1	ITHACA
SPOP	SPOP	speckle type BTB/POZ protein	ITHACA
SPR	SPR	sepiapterin reductase	ITHACA_RND
SPRED1	SPRED1	sprouty related EVH1 domain containing 1	GENTURIS_ITHACA_RND
SPRY2	SPRY2	sprouty RTK signaling antagonist 2	ITHACA
SPTAN1	SPTAN1	spectrin alpha, non-erythrocytic 1	ITHACA_NMD_RND
SPTBN1	SPTBN1	spectrin beta, non-erythrocytic 1	ITHACA
SPTBN2	SPTBN2	spectrin beta, non-erythrocytic 2	ITHACA_NMD_RND
SPTBN4	SPTBN4	spectrin beta, non-erythrocytic 4	ITHACA_NMD
SPTLC1	SPTLC1	serine palmitoyltransferase long chain base subunit 1	NMD_RND
SPTLC2	SPTLC2	serine palmitoyltransferase long chain base subunit 2	NMD_RND
SQSTM1	SQSTM1	sequestosome 1	GENTURIS_ITHACA_NMD_RND
SRC	SRC	SRC proto-oncogene, non-receptor tyrosine kinase	GENTURIS
SRCAP	SRCAP	Snf2 related CREBBP activator protein	ITHACA_RND
SRD5A3	SRD5A3	steroid 5 alpha-reductase 3	ITHACA_RND
SRGAP3	SRGAP3	SLIT-ROBO Rho GTPase activating protein 3	ITHACA
SRP54	SRP54	signal recognition particle 54	ITHACA
SRPX2	SRPX2	sushi repeat containing protein X-linked 2	ITHACA
SRRM2	SRRM2	serine/arginine repetitive matrix 2	ITHACA
SRSF1	SRSF1	serine and arginine rich splicing factor 1	ITHACA
SRSF11	SRSF11	serine and arginine rich splicing factor 11	ITHACA
SSBP1	SSBP1	single stranded DNA binding protein 1	RND
SSR4	SSR4	signal sequence receptor subunit 4	ITHACA_RND
SSTR1	SSTR1	somatostatin receptor 1	ITHACA
ST3GAL3	ST3GAL3	ST3 beta-galactoside alpha-2,3-sialyltransferase 3	ITHACA_RND
ST3GAL5	ST3GAL5	ST3 beta-galactoside alpha-2,3-sialyltransferase 5	ITHACA_RND
ST7	ST7	suppression of tumorigenicity 7	ITHACA
STAC3	STAC3	SH3 and cysteine rich domain 3	ITHACA_NMD_RND
STAG1	STAG1	stromal antigen 1	ITHACA_RND

STAG2	STAG2	stromal antigen 2	ITHACA_RND
STAMBP	STAMBP	STAM binding protein	ITHACA_RND
STARD7	STARD7	StAR related lipid transfer domain containing 7	ITHACA
STARD9	STARD9	StAR related lipid transfer domain containing 9	ITHACA
STAT2	STAT2	signal transducer and activator of transcription 2	ITHACA
STC1	STC1	stanniocalcin 1	ITHACA
STIL	STIL	STIL centriolar assembly protein	ITHACA_RND
STIM1	STIM1	stromal interaction molecule 1	ITHACA_NMD_RND
STK11	STK11	serine/threonine kinase 11	GENTURIS
STK32C	STK32C	serine/threonine kinase 32C	ITHACA
STRA6	STRA6	signaling receptor and transporter of retinol STRA6	ITHACA_RND
STRADA	STRADA	STE20 related adaptor alpha	ITHACA_RND
STS	STS	steroid sulfatase	RND
STT3A	STT3A	STT3 oligosaccharyltransferase complex catalytic subunit A	ITHACA
STT3B	STT3B	STT3 oligosaccharyltransferase complex catalytic subunit B	ITHACA
STUB1	STUB1	STIP1 homology and U-box containing protein 1	ITHACA_NMD_RND
STX11	STX11	syntaxin 11	ITHACA
STX1A	STX1A	syntaxin 1A	ITHACA
STX1B	STX1B	syntaxin 1B	ITHACA_RND
STX3	STX3	syntaxin 3	ITHACA
STXBP1	STXBP1	syntaxin binding protein 1	ITHACA_RND
STXBP5	STXBP5	syntaxin binding protein 5	ITHACA
STXBP5L	STXBP5L	syntaxin binding protein 5L	ITHACA
STYXL1	STYXL1	serine/threonine/tyrosine interacting like 1	ITHACA
SUCLA2	SUCLA2	succinate-CoA ligase ADP-forming subunit beta	ITHACA_NMD_RND
SUCLG1	SUCLG1	succinate-CoA ligase GDP/ADP-forming subunit alpha	ITHACA_NMD_RND
SUFU	SUFU	SUFU negative regulator of hedgehog signaling	GENTURIS_ITHACA_RND
SUMF1	SUMF1	sulfatase modifying factor 1	ITHACA_RND
SUMF2	SUMF2	sulfatase modifying factor 2	ITHACA
SUOX	SUOX	sulfite oxidase	ITHACA_RND
SUPT16H	SUPT16H	SPT16 homolog, facilitates chromatin remodeling subunit	ITHACA
SURF1	SURF1	SURF1 cytochrome c oxidase assembly factor	ITHACA_NMD_RND
SUSD4	SUSD4	sushi domain containing 4	ITHACA
SUV420H1	KMT5B	lysine methyltransferase 5B	ITHACA_RND
SUZ12	SUZ12	SUZ12 polycomb repressive complex 2 subunit	ITHACA
SV2A	SV2A	synaptic vesicle glycoprotein 2A	ITHACA
SV2C	SV2C	synaptic vesicle glycoprotein 2C	ITHACA
SVEP1	SVEP1	sushi, von Willebrand factor type A, EGF and pentraxin domain containing 1	ITHACA
SVIL	SVIL	supervillin	ITHACA_NMD
SYCN	SYCN	syncollin	ITHACA

SYDE2	SYDE2	synapse defective Rho GTPase homolog 2	ITHACA
SYN1	SYN1	synapsin I	ITHACA_RND
SYNCRIP	SYNCRIP	synaptotagmin binding cytoplasmic RNA interacting protein	ITHACA
SYNE1	SYNE1	spectrin repeat containing nuclear envelope protein 1	ITHACA_NMD_RND
SYNE2	SYNE2	spectrin repeat containing nuclear envelope protein 2	NMD_RND
SYNGAP1	SYNGAP1	synaptic Ras GTPase activating protein 1	ITHACA_RND
SYNJ1	SYNJ1	synaptojanin 1	ITHACA_RND
SYNRG	SYNRG	synergin gamma	ITHACA
SYP	SYP	synaptophysin	ITHACA_RND
SYT1	SYT1	synaptotagmin 1	ITHACA_RND
SYT14	SYT14	synaptotagmin 14	ITHACA_NMD
SYT15	SYT15	synaptotagmin 15	ITHACA
SYT2	SYT2	synaptotagmin 2	NMD_RND
SZT2	SZT2	SZT2 subunit of KICSTOR complex	ITHACA_RND
TAB2	TAB2	TGF-beta activated kinase 1 (MAP3K7) binding protein 2	ITHACA
TAC3	TAC3	tachykinin precursor 3	ITHACA
TACO1	TACO1	translational activator of cytochrome c oxidase I	ITHACA_RND
TADA1	TADA1	transcriptional adaptor 1	ITHACA
TAF1	TAF1	TATA-box binding protein associated factor 1	ITHACA_RND
TAF13	TAF13	TATA-box binding protein associated factor 13	ITHACA
TAF1C	TAF1C	TATA-box binding protein associated factor, RNA polymerase I subunit C	ITHACA
TAF1L	TAF1L	TATA-box binding protein associated factor 1 like	ITHACA
TAF2	TAF2	TATA-box binding protein associated factor 2	ITHACA
TAF6	TAF6	TATA-box binding protein associated factor 6	ITHACA_RND
TALDO1	TALDO1	transaldolase 1	RND
TANC2	TANC2	tetratricopeptide repeat, ankyrin repeat and coiled-coil containing 2	ITHACA
TANGO2	TANGO2	transport and golgi organization 2 homolog	ITHACA_RND
TANK	TANK	TRAF family member associated NFKB activator	ITHACA
TAOK1	TAOK1	TAO kinase 1	ITHACA_RND
TARDBP	TARDBP	TAR DNA binding protein	NMD_RND
TARS	TARS1	threonyl-tRNA synthetase 1	ITHACA
TARS2	TARS2	threonyl-tRNA synthetase 2, mitochondrial	ITHACA
TASP1	TASP1	taspase 1	ITHACA
TAT	TAT	tyrosine aminotransferase	ITHACA_RND
TAZ	TAFAZZIN	tafazzin, phospholipid-lysophospholipid transacylase	ITHACA_NMD_RND
TBC1D20	TBC1D20	TBC1 domain family member 20	ITHACA_RND
TBC1D23	TBC1D23	TBC1 domain family member 23	ITHACA_RND
TBC1D24	TBC1D24	TBC1 domain family member 24	ITHACA_RND
TBC1D2B	TBC1D2B	TBC1 domain family member 2B	ITHACA
TBC1D32	TBC1D32	TBC1 domain family member 32	ITHACA
TBC1D7	TBC1D7	TBC1 domain family member 7	ITHACA
TBC1D8	TBC1D8	TBC1 domain family member 8	ITHACA
TBCD	TBCD	tubulin folding cofactor D	ITHACA_NMD_RND

TBCE	TBCE	tubulin folding cofactor E	ITHACA_RND
TBCK	TBCK	TBC1 domain containing kinase	ITHACA_RND
TBK1	TBK1	TANK binding kinase 1	NMD_RND
TBL1XR1	TBL1XR1	TBL1X/Y related 1	ITHACA_RND
TBP	TBP	TATA-box binding protein	ITHACA_NMD
TBR1	TBR1	T-box brain transcription factor 1	ITHACA_RND
TBRG1	TBRG1	transforming growth factor beta regulator 1	ITHACA
TBX1	TBX1	T-box transcription factor 1	ITHACA
TBX2	TBX2	T-box transcription factor 2	ITHACA
TBX6	TBX6	T-box transcription factor 6	ITHACA
TCAP	TCAP	titin-cap	NMD_RND
TCF12	TCF12	transcription factor 12	ITHACA_RND
TCF20	TCF20	transcription factor 20	ITHACA_RND
TCF4	TCF4	transcription factor 4	ITHACA_RND
TCF7L2	TCF7L2	transcription factor 7 like 2	ITHACA
TCN2	TCN2	transcobalamin 2	ITHACA_RND
TCOF1	TCOF1	treacle ribosome biogenesis factor 1	ITHACA
TCTN1	TCTN1	tectonic family member 1	ITHACA_RND
TCTN2	TCTN2	tectonic family member 2	ITHACA_RND
TCTN3	TCTN3	tectonic family member 3	ITHACA_RND
TDGF1	TDGF1	teratocarcinoma-derived growth factor 1	ITHACA
TDP1	TDP1	tyrosyl-DNA phosphodiesterase 1	ITHACA_NMD
TDP2	TDP2	tyrosyl-DNA phosphodiesterase 2	ITHACA_NMD_RND
TECPR2	TECPR2	tectonin beta-propeller repeat containing 2	ITHACA_NMD_RND
TECR	TECR	trans-2,3-enoyl-CoA reductase	ITHACA
TECRL	TECRL	trans-2,3-enoyl-CoA reductase like	NMD
TELO2	TELO2	telomere maintenance 2	ITHACA_RND
TENM2	TENM2	teneurin transmembrane protein 2	ITHACA
TENM3	TENM3	teneurin transmembrane protein 3	ITHACA
TERC	TERC	telomerase RNA component	GENTURIS
TERF2IP	TERF2IP	TERF2 interacting protein	GENTURIS
TERT	TERT	telomerase reverse transcriptase	GENTURIS_ITHACA_RND
TET1	TET1	tet methylcytosine dioxygenase 1	ITHACA
TET2	TET2	tet methylcytosine dioxygenase 2	ITHACA
TET3	TET3	tet methylcytosine dioxygenase 3	ITHACA
TFAP2A	TFAP2A	transcription factor AP-2 alpha	ITHACA
TFAP2B	TFAP2B	transcription factor AP-2 beta	ITHACA
TFCP2L1	TFCP2L1	transcription factor CP2 like 1	ITHACA
TFE3	TFE3	transcription factor binding to IGHM enhancer 3	ITHACA
TFG	TFG	trafficking from ER to golgi regulator	ITHACA_NMD_RND
TFR2	TFR2	transferrin receptor 2	RND
TG	TG	thyroglobulin	ITHACA
TGDS	TGDS	TDP-glucose 4,6-dehydratase	ITHACA
TGFB1	TGFB1	transforming growth factor beta 1	ITHACA
TGFB3	TGFB3	transforming growth factor beta 3	ITHACA_NMD
TGFBR1	TGFBR1	transforming growth factor beta receptor 1	ITHACA
TGFBR2	TGFBR2	transforming growth factor beta receptor 2	ITHACA

TGIF1	TGIF1	TGFB induced factor homeobox 1	ITHACA_RND
TGM3	TGM3	transglutaminase 3	ITHACA
TGM6	TGM6	transglutaminase 6	NMD_RND
TH	TH	tyrosine hydroxylase	ITHACA_RND
THAP1	THAP1	THAP domain containing 1	RND
THAP11	THAP11	THAP domain containing 11	ITHACA
THG1L	THG1L	tRNA-histidine guanylyltransferase 1 like	ITHACA
THOC2	THOC2	THO complex 2	ITHACA_RND
THOC6	THOC6	THO complex 6	ITHACA_RND
THRA	THRA	thyroid hormone receptor alpha	ITHACA_RND
THRB	THRB	thyroid hormone receptor beta	ITHACA
THSD7A	THSD7A	thrombospondin type 1 domain containing 7A	ITHACA
THUMPD1	THUMPD1	THUMP domain containing 1	ITHACA
TIA1	TIA1	TIA1 cytotoxic granule associated RNA binding protein	NMD_RND
TIMM22	TIMM22	translocase of inner mitochondrial membrane 22	NMD
TIMM50	TIMM50	translocase of inner mitochondrial membrane 50	ITHACA_RND
TIMM8A	TIMM8A	translocase of inner mitochondrial membrane 8A	ITHACA_RND
TIMMDC1	TIMMDC1	translocase of inner mitochondrial membrane domain containing 1	ITHACA
TINF2	TINF2	TERF1 interacting nuclear factor 2	GENTURIS_ITHACA_RND
TK2	TK2	thymidine kinase 2	ITHACA_NMD_RND
TKT	TKT	transketolase	ITHACA
TLE1	TLE1	TLE family member 1, transcriptional corepressor	ITHACA
TLK2	TLK2	tousled like kinase 2	ITHACA_RND
TLR3	TLR3	toll like receptor 3	ITHACA
TM2D3	TM2D3	TM2 domain containing 3	ITHACA
TM4SF19	TM4SF19	transmembrane 4 L six family member 19	ITHACA
TM4SF20	TM4SF20	transmembrane 4 L six family member 20	ITHACA
TMCO1	TMCO1	transmembrane and coiled-coil domains 1	ITHACA_RND
TMED7-TICAM2	TMED7-TICAM2	TMED7-TICAM2 readthrough	ITHACA
TMEM106B	TMEM106B	transmembrane protein 106B	ITHACA_RND
TMEM107	TMEM107	transmembrane protein 107	ITHACA_RND
TMEM126A	TMEM126A	transmembrane protein 126A	RND
TMEM126B	TMEM126B	transmembrane protein 126B	RND
TMEM127	TMEM127	transmembrane protein 127	GENTURIS
TMEM132D	TMEM132D	transmembrane protein 132D	ITHACA
TMEM135	TMEM135	transmembrane protein 135	ITHACA
TMEM138	TMEM138	transmembrane protein 138	ITHACA_RND
TMEM147	TMEM147	transmembrane protein 147	ITHACA
TMEM165	TMEM165	transmembrane protein 165	ITHACA_RND
TMEM178A	TMEM178A	transmembrane protein 178A	ITHACA
TMEM199	TMEM199	transmembrane protein 199	ITHACA_RND
TMEM216	TMEM216	transmembrane protein 216	ITHACA_RND
TMEM222	TMEM222	transmembrane protein 222	ITHACA
TMEM231	TMEM231	transmembrane protein 231	ITHACA_RND
TMEM237	TMEM237	transmembrane protein 237	ITHACA_RND

TMEM240	TMEM240	transmembrane protein 240	ITHACA_NMD_RND
TMEM251	TMEM251	transmembrane protein 251	ITHACA
TMEM260	TMEM260	transmembrane protein 260	ITHACA
TMEM27	CLTRN	collectrin, amino acid transport regulator	ITHACA
TMEM38B	TMEM38B	transmembrane protein 38B	ITHACA
TMEM42	TMEM42	transmembrane protein 42	ITHACA
TMEM43	TMEM43	transmembrane protein 43	NMD
TMEM5	RXYLT1	ribitol xylosyltransferase 1	ITHACA_NMD_RND
TMEM63A	TMEM63A	transmembrane protein 63A	ITHACA
TMEM65	TMEM65	transmembrane protein 65	NMD
TMEM67	TMEM67	transmembrane protein 67	ITHACA_RND
TMEM70	TMEM70	transmembrane protein 70	ITHACA_RND
TMEM87B	TMEM87B	transmembrane protein 87B	ITHACA
TMEM8C	MYMK	myomaker, myoblast fusion factor	ITHACA_NMD_RND
TMEM92	TMEM92	transmembrane protein 92	ITHACA
TMLHE	TMLHE	trimethyllysine hydroxylase, epsilon	ITHACA
TMPO	TMPO	thymopoietin	NMD
TMPRSS9	TMPRSS9	transmembrane serine protease 9	ITHACA
TMTC3	TMTC3	transmembrane O-mannosyltransferase targeting cadherins 3	ITHACA_RND
TMX2	TMX2	thioredoxin related transmembrane protein 2	ITHACA_RND
TNFRSF11A	TNFRSF11A	TNF receptor superfamily member 11a	GENTURIS_ITHACA
TNFRSF11B	TNFRSF11B	TNF receptor superfamily member 11b	ITHACA
TNIK	TNIK	TRAF2 and NCK interacting kinase	ITHACA
TNKS	TNKS	tankyrase	ITHACA
TNN	TNN	tenascin N	ITHACA
TNNC1	TNNC1	troponin C1, slow skeletal and cardiac type	NMD
TNNI2	TNNI2	troponin I2, fast skeletal type	NMD_RND
TNNI3	TNNI3	troponin I3, cardiac type	NMD
TNNT1	TNNT1	troponin T1, slow skeletal type	ITHACA_NMD_RND
TNNT2	TNNT2	troponin T2, cardiac type	NMD
TNNT3	TNNT3	troponin T3, fast skeletal type	NMD_RND
TNPO2	TNPO2	transportin 2	ITHACA
TNPO3	TNPO3	transportin 3	ITHACA_NMD_RND
TNR	TNR	tenascin R	ITHACA
TNRC6B	TNRC6B	trinucleotide repeat containing adaptor 6B	ITHACA
TNS3	TNS3	tensin 3	ITHACA
TOE1	TOE1	target of EGR1, exonuclease	ITHACA_RND
TOMM70A	TOMM70	translocase of outer mitochondrial membrane 70	ITHACA
TONSL	TONSL	tonsoku like, DNA repair protein	ITHACA
TOP2B	TOP2B	DNA topoisomerase II beta	ITHACA
TOP3A	TOP3A	DNA topoisomerase III alpha	ITHACA_NMD_RND
TOP3B	TOP3B	DNA topoisomerase III beta	ITHACA

TOR1A	TOR1A	torsin family 1 member A	ITHACA_NMD_RND
TOR1AIP1	TOR1AIP1	torsin 1A interacting protein 1	ITHACA_NMD
TP53	TP53	tumor protein p53	GENTURIS
TP53RK	TP53RK	TP53 regulating kinase	ITHACA
TP53TG5	TP53TG5	TP53 target 5	ITHACA
TP63	TP63	tumor protein p63	ITHACA
TP73	TP73	tumor protein p73	ITHACA
TPI1	TPI1	triosephosphate isomerase 1	ITHACA
TPK1	TPK1	thiamin pyrophosphokinase 1	ITHACA_RND
TPM1	TPM1	tropomyosin 1	NMD
TPM2	TPM2	tropomyosin 2	ITHACA_NMD_RND
TPM3	TPM3	tropomyosin 3	ITHACA_NMD_RND
TPO	TPO	thyroid peroxidase	ITHACA
TPP1	TPP1	tripeptidyl peptidase 1	ITHACA_RND
TPP2	TPP2	tripeptidyl peptidase 2	ITHACA
TPRKB	TPRKB	TP53RK binding protein	ITHACA
TPRN	TPRN	taperin	ITHACA
TRA2B	TRA2B	transformer 2 beta homolog	ITHACA
TRAF3IP1	TRAF3IP1	TRAF3 interacting protein 1	ITHACA
TRAF7	TRAF7	TNF receptor associated factor 7	ITHACA_RND
TRAIP	TRAIP	TRAF interacting protein	ITHACA_RND
TRAK1	TRAK1	trafficking kinesin protein 1	ITHACA_RND
TRAP1	TRAP1	TNF receptor associated protein 1	ITHACA_RND
TRAPPC10	TRAPPC10	trafficking protein particle complex subunit 10	ITHACA
TRAPPC11	TRAPPC11	trafficking protein particle complex subunit 11	ITHACA_NMD_RND
TRAPPC12	TRAPPC12	trafficking protein particle complex subunit 12	ITHACA_RND
TRAPPC2L	TRAPPC2L	trafficking protein particle complex subunit 2L	ITHACA
TRAPPC4	TRAPPC4	trafficking protein particle complex subunit 4	ITHACA
TRAPPC6B	TRAPPC6B	trafficking protein particle complex subunit 6B	ITHACA_RND
TRAPPC9	TRAPPC9	trafficking protein particle complex subunit 9	ITHACA_RND
TRDN	TRDN	triadin	NMD
TREM2	TREM2	triggering receptor expressed on myeloid cells 2	RND
TRERF1	TRERF1	transcriptional regulating factor 1	ITHACA
TREX1	TREX1	three prime repair exonuclease 1	ITHACA_RND
TRH	TRH	thyrotropin releasing hormone	ITHACA
TRIM17	TRIM17	tripartite motif containing 17	ITHACA
TRIM2	TRIM2	tripartite motif containing 2	ITHACA_NMD_RND
TRIM32	TRIM32	tripartite motif containing 32	ITHACA_NMD_RND
TRIM36	TRIM36	tripartite motif containing 36	ITHACA
TRIM37	TRIM37	tripartite motif containing 37	ITHACA_RND
TRIM47	TRIM47	tripartite motif containing 47	ITHACA
TRIM54	TRIM54	tripartite motif containing 54	NMD
TRIM63	TRIM63	tripartite motif containing 63	NMD
TRIM71	TRIM71	tripartite motif containing 71	ITHACA
TRIM8	TRIM8	tripartite motif containing 8	ITHACA_RND

TRIO	TRIO	trio Rho guanine nucleotide exchange factor	ITHACA_RND
TRIP11	TRIP11	thyroid hormone receptor interactor 11	ITHACA
TRIP12	TRIP12	thyroid hormone receptor interactor 12	ITHACA_RND
TRIP13	TRIP13	thyroid hormone receptor interactor 13	GENTURIS_ITHACA
TRIP4	TRIP4	thyroid hormone receptor interactor 4	ITHACA_NMD_RND
TRIT1	TRIT1	tRNA isopentenyltransferase 1	ITHACA_RND
TRMT1	TRMT1	tRNA methyltransferase 1	ITHACA_RND
TRMT10A	TRMT10A	tRNA methyltransferase 10A	ITHACA_RND
TRMT10C	TRMT10C	tRNA methyltransferase 10C, mitochondrial RNase P subunit	ITHACA_RND
TRMT5	TRMT5	tRNA methyltransferase 5	ITHACA_RND
TRMU	TRMU	tRNA mitochondrial 2-thiouridylase	RND
TRNT1	TRNT1	tRNA nucleotidyl transferase 1	ITHACA_RND
TROVE2	RO60	Ro60, Y RNA binding protein	ITHACA
TRPC3	TRPC3	transient receptor potential cation channel subfamily C member 3	NMD
TRPM3	TRPM3	transient receptor potential cation channel subfamily M member 3	ITHACA
TRPM6	TRPM6	transient receptor potential cation channel subfamily M member 6	ITHACA_RND
TRPS1	TRPS1	transcriptional repressor GATA binding 1	ITHACA
TRPV4	TRPV4	transient receptor potential cation channel subfamily V member 4	ITHACA_NMD_RND
TRPV6	TRPV6	transient receptor potential cation channel subfamily V member 6	ITHACA
TRRAP	TRRAP	transformation/transcription domain associated protein	ITHACA_RND
TSC1	TSC1	TSC complex subunit 1	GENTURIS_ITHACA_RND
TSC2	TSC2	TSC complex subunit 2	GENTURIS_ITHACA_RND
TSEN15	TSEN15	tRNA splicing endonuclease subunit 15	ITHACA_RND
TSEN2	TSEN2	tRNA splicing endonuclease subunit 2	ITHACA_RND
TSEN34	TSEN34	tRNA splicing endonuclease subunit 34	ITHACA_RND
TSEN54	TSEN54	tRNA splicing endonuclease subunit 54	ITHACA_RND
TSFM	TSFM	Ts translation elongation factor, mitochondrial	ITHACA_NMD_RND
TSHB	TSHB	thyroid stimulating hormone subunit beta	ITHACA_RND
TSHR	TSHR	thyroid stimulating hormone receptor	ITHACA
TSHZ3	TSHZ3	teashirt zinc finger homeobox 3	ITHACA
TSPAN18	TSPAN18	tetraspanin 18	ITHACA
TSPAN7	TSPAN7	tetraspanin 7	ITHACA_RND
TTBK2	TTBK2	tau tubulin kinase 2	NMD_RND
TTC1	TTC1	tetratricopeptide repeat domain 1	ITHACA
TTC19	TTC19	tetratricopeptide repeat domain 19	ITHACA_RND
TTC28	TTC28	tetratricopeptide repeat domain 28	ITHACA
TTC37	SKIC3	SKI3 subunit of superkiller complex	ITHACA_RND
TTC5	TTC5	tetratricopeptide repeat domain 5	ITHACA
TTC8	TTC8	tetratricopeptide repeat domain 8	ITHACA_RND

TTI1	TTI1	TELO2 interacting protein 1	ITHACA
TTI2	TTI2	TELO2 interacting protein 2	ITHACA_RND
TTN	TTN	titin	NMD_RND
TTPA	TTPA	alpha tocopherol transfer protein	NMD_RND
TTR	TTR	transthyretin	NMD_RND
TUB	TUB	TUB bipartite transcription factor	ITHACA
TUBA1A	TUBA1A	tubulin alpha 1a	ITHACA_RND
TUBA3E	TUBA3E	tubulin alpha 3e	ITHACA
TUBA4A	TUBA4A	tubulin alpha 4a	NMD
TUBA8	TUBA8	tubulin alpha 8	ITHACA
TUBAL3	TUBAL3	tubulin alpha like 3	ITHACA
TUBB	TUBB	tubulin beta class I	ITHACA_RND
TUBB2A	TUBB2A	tubulin beta 2A class IIa	ITHACA_RND
TUBB2B	TUBB2B	tubulin beta 2B class IIb	ITHACA_RND
TUBB3	TUBB3	tubulin beta 3 class III	ITHACA_NMD_RND
TUBB4A	TUBB4A	tubulin beta 4A class IVa	ITHACA_RND
TUBG1	TUBG1	tubulin gamma 1	ITHACA_RND
TUBGCP2	TUBGCP2	tubulin gamma complex associated protein 2	ITHACA
TUBGCP4	TUBGCP4	tubulin gamma complex associated protein 4	ITHACA
TUBGCP5	TUBGCP5	tubulin gamma complex associated protein 5	ITHACA
TUBGCP6	TUBGCP6	tubulin gamma complex associated protein 6	ITHACA_RND
TUFM	TUFM	Tu translation elongation factor, mitochondrial	ITHACA_RND
TUSC3	TUSC3	tumor suppressor candidate 3	ITHACA_RND
TUT1	TUT1	terminal uridylyl transferase 1, U6 snRNA-specific	ITHACA
TWIST1	TWIST1	twist family bHLH transcription factor 1	ITHACA_RND
TWIST2	TWIST2	twist family bHLH transcription factor 2	ITHACA
TXN2	TXN2	thioredoxin 2	ITHACA
TXNDC15	TXNDC15	thioredoxin domain containing 15	RND
TXNRD2	TXNRD2	thioredoxin reductase 2	ITHACA
TYMP	TYMP	thymidine phosphorylase	NMD_RND
TYROBP	TYROBP	transmembrane immune signaling adaptor TYROBP	RND
U2AF2	U2AF2	U2 small nuclear RNA auxiliary factor 2	ITHACA
UAP1	UAP1	UDP-N-acetylglucosamine pyrophosphorylase 1	ITHACA
UBA1	UBA1	ubiquitin like modifier activating enzyme 1	NMD_RND
UBA5	UBA5	ubiquitin like modifier activating enzyme 5	ITHACA_RND
UBA7	UBA7	ubiquitin like modifier activating enzyme 7	ITHACA
UBAP1	UBAP1	ubiquitin associated protein 1	ITHACA_NMD_RND
UBE2A	UBE2A	ubiquitin conjugating enzyme E2 A	ITHACA_RND
UBE2H	UBE2H	ubiquitin conjugating enzyme E2 H	ITHACA
UBE2J2	UBE2J2	ubiquitin conjugating enzyme E2 J2	ITHACA
UBE3A	UBE3A	ubiquitin protein ligase E3A	ITHACA_RND
UBE3B	UBE3B	ubiquitin protein ligase E3B	ITHACA_RND
UBE3C	UBE3C	ubiquitin protein ligase E3C	ITHACA
UBE4A	UBE4A	ubiquitination factor E4A	ITHACA
UBN2	UBN2	ubinuclein 2	ITHACA
UBQLN1	UBQLN1	ubiquilin 1	ITHACA
UBQLN2	UBQLN2	ubiquilin 2	NMD_RND

UBR1	UBR1	ubiquitin protein ligase E3 component n-recognin 1	ITHACA_RND
UBR4	UBR4	ubiquitin protein ligase E3 component n-recognin 4	ITHACA
UBR7	UBR7	ubiquitin protein ligase E3 component n-recognin 7	ITHACA
UBTD2	UBTD2	ubiquitin domain containing 2	ITHACA
UBTF	UBTF	upstream binding transcription factor	ITHACA_RND
UCHL1	UCHL1	ubiquitin C-terminal hydrolase L1	ITHACA_NMD_RND
UFC1	UFC1	ubiquitin-fold modifier conjugating enzyme 1	ITHACA
UFM1	UFM1	ubiquitin fold modifier 1	ITHACA_RND
UFSP2	UFSP2	UFM1 specific peptidase 2	ITHACA
UGDH	UGDH	UDP-glucose 6-dehydrogenase	ITHACA_RND
UGP2	UGP2	UDP-glucose pyrophosphorylase 2	ITHACA
UGT1A1	UGT1A1	UDP glucuronosyltransferase family 1 member A1	RND
UIMC1	UIMC1	ubiquitin interaction motif containing 1	ITHACA
ULK2	ULK2	unc-51 like autophagy activating kinase 2	ITHACA
UMOD	UMOD	uromodulin	RND
UMPS	UMPS	uridine monophosphate synthetase	ITHACA_RND
UNC13A	UNC13A	unc-13 homolog A	ITHACA
UNC13B	UNC13B	unc-13 homolog B	NMD
UNC45A	UNC45A	unc-45 myosin chaperone A	ITHACA
UNC45B	UNC45B	unc-45 myosin chaperone B	ITHACA_NMD
UNC5A	UNC5A	unc-5 netrin receptor A	ITHACA
UNC79	UNC79	unc-79 homolog, NALCN channel complex subunit	ITHACA
UNC80	UNC80	unc-80 homolog, NALCN channel complex subunit	ITHACA_RND
UPB1	UPB1	beta-ureidopropionase 1	ITHACA_RND
UPF1	UPF1	UPF1 RNA helicase and ATPase	ITHACA
UPF2	UPF2	UPF2 regulator of nonsense mediated mRNA decay	ITHACA
UPF3B	UPF3B	UPF3B regulator of nonsense mediated mRNA decay	ITHACA_RND
UQCC2	UQCC2	ubiquinol-cytochrome c reductase complex assembly factor 2	ITHACA_RND
UQCRB	UQCRB	ubiquinol-cytochrome c reductase binding protein	ITHACA_RND
UQCRC2	UQCRC2	ubiquinol-cytochrome c reductase core protein 2	ITHACA
UQCRFS1	UQCRFS1	ubiquinol-cytochrome c reductase, Rieske iron-sulfur polypeptide 1	ITHACA
UQCRQ	UQCRQ	ubiquinol-cytochrome c reductase complex III subunit VII	ITHACA
UROC1	UROC1	urocanate hydratase 1	ITHACA_RND
UROD	UROD	uroporphyrinogen decarboxylase	RND
UROS	UROS	uroporphyrinogen III synthase	RND
USB1	USB1	U6 snRNA biogenesis phosphodiesterase 1	GENTURIS
USH1C	USH1C	USH1 protein network component harmonin	ITHACA
USMG5	ATP5MK	ATP synthase membrane subunit k	ITHACA
USP15	USP15	ubiquitin specific peptidase 15	ITHACA
USP18	USP18	ubiquitin specific peptidase 18	ITHACA_RND
USP2	USP2	ubiquitin specific peptidase 2	ITHACA
USP24	USP24	ubiquitin specific peptidase 24	ITHACA

USP27X	USP27X	ubiquitin specific peptidase 27 X-linked	ITHACA
USP44	USP44	ubiquitin specific peptidase 44	ITHACA
USP7	USP7	ubiquitin specific peptidase 7	ITHACA
USP9X	USP9X	ubiquitin specific peptidase 9 X-linked	ITHACA_RND
VAC14	VAC14	VAC14 component of PIKFYVE complex	ITHACA_RND
VAMP1	VAMP1	vesicle associated membrane protein 1	ITHACA_NMD_RND
VAMP2	VAMP2	vesicle associated membrane protein 2	ITHACA_RND
VAMP4	VAMP4	vesicle associated membrane protein 4	ITHACA
VAPB	VAPB	VAMP associated protein B and C	NMD_RND
VARS	VARS1	valyl-tRNA synthetase 1	ITHACA_RND
VARS2	VARS2	valyl-tRNA synthetase 2, mitochondrial	ITHACA_RND
VAX1	VAX1	ventral anterior homeobox 1	ITHACA
VCL	VCL	vinculin	NMD
VCP	VCP	valosin containing protein	ITHACA_NMD_RND
VDR	VDR	vitamin D receptor	ITHACA
VEZF1	VEZF1	vascular endothelial zinc finger 1	ITHACA
VHL	VHL	von Hippel-Lindau tumor suppressor	GENTURIS
VIPAS39	VIPAS39	VPS33B interacting protein, apical-basolateral polarity regulator, spe-39 homolog	ITHACA_RND
VKORC1	VKORC1	vitamin K epoxide reductase complex subunit 1	RND
VLDLR	VLDLR	very low density lipoprotein receptor	ITHACA_RND
VMA21	VMA21	vacuolar ATPase assembly factor VMA21	ITHACA_NMD_RND
VPS11	VPS11	VPS11 core subunit of CORVET and HOPS complexes	ITHACA_RND
VPS13A	VPS13A	vacuolar protein sorting 13 homolog A	RND
VPS13B	VPS13B	vacuolar protein sorting 13 homolog B	ITHACA_RND
VPS13D	VPS13D	vacuolar protein sorting 13 homolog D	ITHACA_NMD_RND
VPS16	VPS16	VPS16 core subunit of CORVET and HOPS complexes	ITHACA_RND
VPS26A	VPS26A	VPS26 retromer complex component A	ITHACA
VPS33A	VPS33A	VPS33A core subunit of CORVET and HOPS complexes	ITHACA
VPS33B	VPS33B	VPS33B late endosome and lysosome associated	ITHACA_RND
VPS35	VPS35	VPS35 retromer complex component	ITHACA_RND
VPS36	VPS36	vacuolar protein sorting 36 homolog	ITHACA
VPS37A	VPS37A	VPS37A subunit of ESCRT-I	ITHACA_NMD_RND
VPS41	VPS41	VPS41 subunit of HOPS complex	ITHACA
VPS45	VPS45	vacuolar protein sorting 45 homolog	ITHACA
VPS4A	VPS4A	vacuolar protein sorting 4 homolog A	ITHACA
VPS51	VPS51	VPS51 subunit of GARP complex	ITHACA
VPS53	VPS53	VPS53 subunit of GARP complex	ITHACA_RND
VRK1	VRK1	VRK serine/threonine kinase 1	ITHACA_NMD_RND
VWA1	VWA1	von Willebrand factor A domain containing 1	RND
VWA3B	VWA3B	von Willebrand factor A domain containing 3B	ITHACA_NMD
WAC	WAC	WW domain containing adaptor with coiled-coil	ITHACA_RND

WARS	WARS1	tryptophanyl-tRNA synthetase 1	NMD_RND
WARS2	WARS2	tryptophanyl tRNA synthetase 2, mitochondrial	ITHACA_RND
WAS	WAS	WASP actin nucleation promoting factor	GENTURIS
WASF1	WASF1	WASP family member 1	ITHACA_RND
WDFY3	WDFY3	WD repeat and FYVE domain containing 3	ITHACA
WDFY4	WDFY4	WDFY family member 4	ITHACA
WDPCP	WDPCP	WD repeat containing planar cell polarity effector	ITHACA_RND
WDR13	WDR13	WD repeat domain 13	ITHACA
WDR26	WDR26	WD repeat domain 26	ITHACA_RND
WDR34	DYNC2I2	dynein 2 intermediate chain 2	ITHACA
WDR35	WDR35	WD repeat domain 35	ITHACA
WDR37	WDR37	WD repeat domain 37	ITHACA_RND
WDR4	WDR4	WD repeat domain 4	ITHACA
WDR45	WDR45	WD repeat domain 45	ITHACA_RND
WDR45B	WDR45B	WD repeat domain 45B	ITHACA_RND
WDR48	WDR48	WD repeat domain 48	RND
WDR5	WDR5	WD repeat domain 5	ITHACA
WDR52	CFAP44	cilia and flagella associated protein 44	ITHACA
WDR60	DYNC2I1	dynein 2 intermediate chain 1	ITHACA
WDR62	WDR62	WD repeat domain 62	ITHACA_RND
WDR73	WDR73	WD repeat domain 73	ITHACA_RND
WDR81	WDR81	WD repeat domain 81	ITHACA_RND
WDR87	WDR87	WD repeat domain 87	ITHACA
WDR93	WDR93	WD repeat domain 93	ITHACA
WDR96	CFAP43	cilia and flagella associated protein 43	ITHACA
WFDC1	WFDC1	WAP four-disulfide core domain 1	ITHACA
WFS1	WFS1	wolframin ER transmembrane glycoprotein	ITHACA_RND
WHSC1	NSD2	nuclear receptor binding SET domain protein 2	ITHACA_RND
WIPI2	WIPI2	WD repeat domain, phosphoinositide interacting 2	ITHACA
WNK1	WNK1	WNK lysine deficient protein kinase 1	NMD_RND
WNT1	WNT1	Wnt family member 1	ITHACA
WNT3	WNT3	Wnt family member 3	ITHACA
WNT4	WNT4	Wnt family member 4	ITHACA
WNT5A	WNT5A	Wnt family member 5A	ITHACA
WRAP53	WRAP53	WD repeat containing antisense to TP53	GENTURIS_ITHACA
WRN	WRN	WRN RecQ like helicase	GENTURIS
WT1	WT1	WT1 transcription factor	GENTURIS
WWOX	WWOX	WW domain containing oxidoreductase	ITHACA_NMD_RND
WWP2	WWP2	WW domain containing E3 ubiquitin protein ligase 2	ITHACA
XDH	XDH	xanthine dehydrogenase	RND
XIRP1	XIRP1	xin actin binding repeat containing 1	ITHACA
XPA	XPA	XPA, DNA damage recognition and repair factor	GENTURIS_ITHACA_RND
XPC	XPC	XPC complex subunit, DNA damage recognition and repair factor	GENTURIS_RND
XPNPEP2	XPNPEP2	X-prolyl aminopeptidase 2	ITHACA
XPNPEP3	XPNPEP3	X-prolyl aminopeptidase 3	ITHACA

XPO7	XPO7	exportin 7	ITHACA
XPOT	XPOT	exportin for tRNA	ITHACA
XPR1	XPR1	xenotropic and polytropic retrovirus receptor 1	RND
XRCC1	XRCC1	X-ray repair cross complementing 1	NMD
XRCC4	XRCC4	X-ray repair cross complementing 4	ITHACA_RND
XRCC6	XRCC6	X-ray repair cross complementing 6	ITHACA
XRRA1	XRRA1	X-ray radiation resistance associated 1	ITHACA
XYLT1	XYLT1	xylosyltransferase 1	ITHACA_RND
XYLT2	XYLT2	xylosyltransferase 2	ITHACA_RND
YAP1	YAP1	Yes1 associated transcriptional regulator	ITHACA
YARS	YARS1	tyrosyl-tRNA synthetase 1	ITHACA_NMD_RND
YARS2	YARS2	tyrosyl-tRNA synthetase 2	NMD_RND
YIF1B	YIF1B	Yip1 interacting factor homolog B, membrane trafficking protein	ITHACA
YIPF5	YIPF5	Yip1 domain family member 5	ITHACA
YME1L1	YME1L1	YME1 like 1 ATPase	ITHACA
YTHDF1	YTHDF1	YTH N6-methyladenosine RNA binding protein 1	ITHACA
YTHDF3	YTHDF3	YTH N6-methyladenosine RNA binding protein 3	ITHACA
YWHAE	YWHAE	tyrosine 3-monooxygenase/tryptophan 5-monooxygenase activation protein epsilon	ITHACA
YWHAG	YWHAG	tyrosine 3-monooxygenase/tryptophan 5-monooxygenase activation protein gamma	ITHACA_RND
YWHAZ	YWHAZ	tyrosine 3-monooxygenase/tryptophan 5-monooxygenase activation protein zeta	ITHACA
YY1	YY1	YY1 transcription factor	ITHACA_RND
YY1AP1	YY1AP1	YY1 associated protein 1	ITHACA
ZBTB11	ZBTB11	zinc finger and BTB domain containing 11	ITHACA
ZBTB16	ZBTB16	zinc finger and BTB domain containing 16	ITHACA
ZBTB18	ZBTB18	zinc finger and BTB domain containing 18	ITHACA_RND
ZBTB20	ZBTB20	zinc finger and BTB domain containing 20	ITHACA_RND
ZBTB24	ZBTB24	zinc finger and BTB domain containing 24	ITHACA_RND
ZBTB40	ZBTB40	zinc finger and BTB domain containing 40	ITHACA
ZBTB42	ZBTB42	zinc finger and BTB domain containing 42	NMD
ZBTB7A	ZBTB7A	zinc finger and BTB domain containing 7A	ITHACA
ZC3H14	ZC3H14	zinc finger CCCH-type containing 14	ITHACA
ZC3H4	ZC3H4	zinc finger CCCH-type containing 4	ITHACA
ZC4H2	ZC4H2	zinc finger C4H2-type containing	ITHACA_RND
ZCCHC8	ZCCHC8	zinc finger CCHC-type containing 8	ITHACA
ZDHHC15	ZDHHC15	zinc finger DHHC-type palmitoyltransferase 15	ITHACA
ZDHHC9	ZDHHC9	zinc finger DHHC-type palmitoyltransferase 9	ITHACA_RND
ZEB2	ZEB2	zinc finger E-box binding homeobox 2	ITHACA_RND
ZFAND2B	ZFAND2B	zinc finger AN1-type containing 2B	ITHACA
ZFAT	ZFAT	zinc finger and AT-hook domain containing	ITHACA
ZFHX2	ZFHX2	zinc finger homeobox 2	NMD
ZFHX4	ZFHX4	zinc finger homeobox 4	ITHACA
ZFP57	ZFP57	ZFP57 zinc finger protein	ITHACA
ZFX	ZFX	zinc finger protein X-linked	ITHACA
ZFYVE26	ZFYVE26	zinc finger FYVE-type containing 26	ITHACA_NMD_RND
ZFYVE27	ZFYVE27	zinc finger FYVE-type containing 27	NMD

ZIC1	ZIC1	Zic family member 1	ITHACA
ZIC2	ZIC2	Zic family member 2	ITHACA_RND
ZIC3	ZIC3	Zic family member 3	RND
ZMIZ1	ZMIZ1	zinc finger MIZ-type containing 1	ITHACA_RND
ZMYM2	ZMYM2	zinc finger MYM-type containing 2	ITHACA
ZMYM3	ZMYM3	zinc finger MYM-type containing 3	ITHACA
ZMYM5	ZMYM5	zinc finger MYM-type containing 5	ITHACA
ZMYM6	ZMYM6	zinc finger MYM-type containing 6	ITHACA
ZMYND11	ZMYND11	zinc finger MYND-type containing 11	ITHACA_RND
ZNF142	ZNF142	zinc finger protein 142	ITHACA_RND
ZNF148	ZNF148	zinc finger protein 148	ITHACA
ZNF259	ZPR1	ZPR1 zinc finger	ITHACA
ZNF292	ZNF292	zinc finger protein 292	ITHACA
ZNF335	ZNF335	zinc finger protein 335	ITHACA
ZNF407	ZNF407	zinc finger protein 407	ITHACA
ZNF41	ZNF41	zinc finger protein 41	ITHACA
ZNF420	ZNF420	zinc finger protein 420	ITHACA
ZNF423	ZNF423	zinc finger protein 423	ITHACA
ZNF462	ZNF462	zinc finger protein 462	ITHACA_RND
ZNF526	ZNF526	zinc finger protein 526	ITHACA
ZNF528	ZNF528	zinc finger protein 528	ITHACA
ZNF589	ZNF589	zinc finger protein 589	ITHACA
ZNF592	ZNF592	zinc finger protein 592	ITHACA
ZNF599	ZNF599	zinc finger protein 599	ITHACA
ZNF668	ZNF668	zinc finger protein 668	ITHACA
ZNF711	ZNF711	zinc finger protein 711	ITHACA_RND
ZNF713	ZNF713	zinc finger protein 713	ITHACA
ZNF804A	ZNF804A	zinc finger protein 804A	ITHACA
ZNF81	ZNF81	zinc finger protein 81	ITHACA
ZNHIT3	ZNHIT3	zinc finger HIT-type containing 3	ITHACA
ZNRF3	ZNRF3	zinc and ring finger 3	ITHACA
ZSCAN25	ZSCAN25	zinc finger and SCAN domain containing 25	ITHACA
ZSWIM6	ZSWIM6	zinc finger SWIM-type containing 6	ITHACA_RND
ZSWIM8	ZSWIM8	zinc finger SWIM-type containing 8	ITHACA

Supplementary Table 2

Source	Annotation	Description
AnnotSV	ACMG_class	SV ranking class
AnnotSV	AnnotSV_ranking_criteria	Decision criteria explaining the AnnotSV ranking score
AnnotSV	AnnotSV_ranking_score	SV ranking score following the 2019 joint consensus recommendation of ACMG and ClinGen. Scoring: pathogenic ≥0.99 likely pathogenic [0.90;0.98] variant of uncertain significance [0.89;-0.89] likely benign [-0.90;-0.98] benign ≤-0.99.
AnnotSV	B_gain_coord	Coordinates of the benign gain genomic regions
AnnotSV	B_gain_source	Origin of the benign gain genomic regions completely overlapping the CNV
AnnotSV	B_loss_coord	Coordinates of the benign loss genomic regions
AnnotSV	B_loss_source	Origin of the benign loss genomic regions completely overlapping the CNV
AnnotSV	DDD_consequence	DDD mutation consequence: e.g. "loss of function" uncertain
AnnotSV	DDD_disease	DDD disease name
AnnotSV	DDD_HI_percent	Haploinsufficiency ranks from Deciphering Developmental Disorders (DDD)
AnnotSV	DDD_mode	DDD allelic requirement: e.g. biallelic hemizygous
AnnotSV	DDD_status	DDD status: e.g. confirmed probable
AnnotSV	ENCODE_blacklist_left	The human ENCODE blacklist is a set of regions that have anomalous unstructured or high signal in NGS experiments - if there is an annotation here highly likely CNV is false positive
AnnotSV	ENCODE_blacklist_right	See above
AnnotSV	ENCODE_blacklist_characteristics_left	See above
AnnotSV	ENCODE_blacklist_characteristics_right	See above
AnnotSV	ExAC_cnvZ	cnvZ_ExAC (Z score) from ExAC indicate gene intolerance to CNV. Higher positive values indicate greater intolerance.
AnnotSV	ExAC_delZ	delZ_ExAC (Z score) from ExAC indicate gene intolerance to deletion. Higher positive values indicate greater intolerance.
AnnotSV	ExAC_dupZ	dupZ_ExAC (Z score) from ExAC indicate gene intolerance to duplication. Higher positive values indicate greater intolerance.
AnnotSV	ExAC_misZ	“yes” if the SV overlaps an OMIM morbid gene candidate
AnnotSV	ExAC_pLI	Score computed by ExAC indicating the probability that a gene is intolerant to a loss of function variation.
AnnotSV	ExAC_synZ	“yes” if the SV overlaps an OMIM morbid gene
AnnotSV	Gene_count	Total of all overlapping genes
AnnotSV	GnomAD_pLI	Score computed by gnomAD indicating the probability that a gene is intolerant to a loss of function variation.
AnnotSV	HI	ClinGen Haploinsufficiency Score (1 is confirmed 2 is likely 3 is dubious)

AnnotSV	LOEUF_bin	Loss-of-function observed/expected upper bound fraction. Low LOEUF scores (e.g. 0) indicate strong selection against predicted loss-of-function (pLoF) variation.
AnnotSV	OMIM_ID	OMIM Gene ID
AnnotSV	OMIM_morbid_candidate	OMIM ID candidate gene associated with disease
AnnotSV	OMIM_morbid_candidate	“yes” if the SV overlaps an OMIM morbid gene candidate
AnnotSV	OMIM_morbid	OMIM ID gene associated with disease
AnnotSV	OMIM_morbid	“yes” if the SV overlaps an OMIM morbid gene
AnnotSV	P_gain_coord	Coordinates of the pathogenic gain genomic regions
AnnotSV	P_gain_hpo	HPO terms describing the pathogenic gain genomic regions
AnnotSV	P_gain_phen	Phenotype of the pathogenic gain genomic regions completely overlapped with the SV
AnnotSV	P_gain_source	Origin of the pathogenic gain genomic regions
AnnotSV	P_loss_coord	Coordinates of the pathogenic loss genomic regions
AnnotSV	P_loss_hpo	HPO terms describing the pathogenic loss genomic regions
AnnotSV	P_loss_phen	Phenotype of the pathogenic loss genomic regions completely overlapped with the SV
AnnotSV	P_loss_source	Origin of the pathogenic loss genomic regions
AnnotSV	P_snvindel_nb	Number of pathogenic SNVs/InDels from public databases completely overlapped with the SV
AnnotSV	P_snvindel_phen	Phenotype of pathogenic SNVs/InDels from public databases completely overlapped with the SV
AnnotSV	RE_gene	Name of the genes regulated by a regulatory element overlapped with the SV to annotate
AnnotSV	SegDup_left	Co-ordinates of large segmental duplication regions - if there is an annotation here highly likely CNV is false positive
AnnotSV	SegDup_right	Co-ordinates of large segmental duplication regions - if there is an annotation here highly likely CNV is false positive
AnnotSV	SV_chrom	Chromosome on which CNV occurs
AnnotSV	SV_end	CNV start co-ordinates
AnnotSV	SV_start	CNV end co-ordinates
AnnotSV	SV.type	DELetion or DUPlication
AnnotSV	TS	ClinGen Triplosensitivity Score (1 is confirmed 2 is likely 3 is dubious)
AnnotSV	Type	DELetion or DUPlication
ClinCNV	ClinC_AF	AF for the CNV within Solve-RD
ClinCNV	ClinC_father	Copy Number in Father (if available)
ClinCNV	ClinC_inh	Inherited or de novo CNV (if parents were available)
ClinCNV	ClinC_LogL	The higher the better - anything over 50 should be real, anything over 200 very robust
ClinCNV	ClinC_mother	Copy Number in Mother (if available)
ClinCNV	ClinC_Prio	ClinCNV prioritisation - 2 is more interesting than 1
ClinCNV	ClinC_targets	Number of exome kit targets affected
ClinCNV	CN_change	Ploidy if CNV is real

ClinCNV	loglikelihood	The higher the better - anything over 50 should be real anything over 200 very robust
ClinCNV	no_of_regions	Number of exome kit targets affected
ClinCNV	qvalue	Lower the better
ClinCNV	Suggested_status	Inherited or de novo CNV (if parents were available)
CNV_End	CNV_End	Self-explanatory
CNV_Len	CNV_Len	Self-explanatory
CNV_Sta	CNV_Sta	Self-explanatory
Conifer	Con_geneOverlap	Overlapping Gene
Conifer	Con_Z-score	Z-score - further from 0 more likely true
ExomeDepth	ED_BF	Bayes Factor
ExomeDepth	ED_Conrad	If overlapping CNV was seen in Conrad et al (2010) - If there is a "CNVR" here then more likely that CNV is benign
ExomeDepth	ED_exp	Expected number of reads in diploid individual across this region
ExomeDepth	ED_obs	Observed number of reads across this region
ExomeDepth	ED_QC	Indication as to whether the call is likely to be LowQuality (more likely false positive) and reason
ExomeDepth	ED_ratio	Ratio of observed/expected number of reads - used to determine ploidy reported in the "CN" field for ExomeDepth CNVs
ExomeDepth	ED_targets	Number of exome kit targets affected
Solve-RD	Gene_Chr	ERN gene chromosome
Solve-RD	Gene_End	ERN gene end coordinate
Solve-RD	Gene	Gene name
Solve-RD	Gene_inh	ERN gene mode of inheritance when disease causing (OMIM and/or Tubingen internal definition)
Solve-RD	Gene_Len	ERN gene length
Solve-RD	Gene_name	HGNC gene symbol
Solve-RD	Gene_Sta	ERN gene start coordinate
Solve-RD	Overlap	Number of nucleotides of CNV overlapping with ERN gene
Solve-RD	Sex	Sex of individual
Solve-RD	CN	New ploidy if CNV is real e.g. 0 for homozygous deletion 1 for heterozygous deletion
Solve-RD	CNV_Class	"Long", "Homozygous deletion" etc.
Solve-RD	Consanguineous	Experimentally determined consanguinity status
Solve-RD	Tool	One of ClinCNV Conifer ExomeDepth. CNVs may have been identified by more than one tool but will be shown on different rows

Supplementary Table 3

ERN	Families Analysed	Total individuals	Affected Individuals	Families with at least one CNV call	Affected individuals with at least one CNV call
**GENTURIS**	**340**	**369**	**357**	**113**	**116**
**ITHACA***	**1.788**	**4.140**	**1.859**	**1.239**	**1.273**
**NMD**	**1.461**	**2.002**	**1.608**	**778**	**833**
**RND**	**2.168**	**2.660**	**2.319**	**1.170**	**1.214**
**Total**	**5.757**	**9.171**	**6.143**	**3.300**	**3.436**

Supplementary Table 4

Tool	Deletions	Duplications	All CNVs	Proportion of all CNV calls by tool	Proportion of duplications calls by each tool
ClinCNV	1.561	1.221	2.782	0,35	0,44
Conifer	233	629	862	0,11	0,73
ExomeDepth	1.693	2.512	4.205	0,54	0,60
Total	3.487	4.362	7.849	1	0,56

Supplementary Table 5

Tool	Length of Duplications (n=3,487)				Length of Deletions (n=4,362)			
	Min	Max	Median	Mean	Min	Max	Median	Mean
ClinCNV	129	5.338.196	3.655	139.028	124	14.803.639	583	110.786
Conifer	771	23.550.077	599.167	1.235.880	2.440	13.012.339	732.642	1.166.805
Exome Depth	23	16.302.705	79.763	309.262	16	9.719.821	25.590	122.824
